# Evaluation of Protein Dihedral Angle Prediction Methods

**DOI:** 10.1371/journal.pone.0105667

**Published:** 2014-08-28

**Authors:** Harinder Singh, Sandeep Singh, Gajendra P. S. Raghava

**Affiliations:** Bioinformatics Center, Institute of Microbial Technology, Chandigarh, India; UMR-S665, INSERM, Université Paris Diderot, INTS, France

## Abstract

Tertiary structure prediction of a protein from its amino acid sequence is one of the major challenges in the field of bioinformatics. Hierarchical approach is one of the persuasive techniques used for predicting protein tertiary structure, especially in the absence of homologous protein structures. In hierarchical approach, intermediate states are predicted like secondary structure, dihedral angles, C^α^-C^α^ distance bounds, etc. These intermediate states are used to restraint the protein backbone and assist its correct folding. In the recent years, several methods have been developed for predicting dihedral angles of a protein, but it is difficult to conclude which method is better than others. In this study, we benchmarked the performance of dihedral prediction methods ANGLOR and SPINE X on various datasets, including independent datasets. TANGLE dihedral prediction method was not benchmarked (due to unavailability of its standalone) and was compared with SPINE X and ANGLOR on only ANGLOR dataset on which TANGLE has reported its results. It was observed that SPINE X performed better than ANGLOR and TANGLE, especially in case of prediction of dihedral angles of glycine and proline residues. The analysis suggested that angle shifting was the foremost reason of better performance of SPINE X. We further evaluated the performance of the methods on independent ccPDB30 dataset and observed that SPINE X performed better than ANGLOR.

## Introduction

One of the ultimate goals of bioinformatics is the prediction of protein tertiary structure from its primary sequence. In the past, several techniques were developed for predicting tertiary structure of a protein that includes homology and threading based approaches [1], [2], [3], [4], [5]. The performance of these methods depends on the homology between query and target sequences. Therefore, these techniques work best when homologous templates are available and are not designed to work in the absence of homologous protein sequence/structure. Hierarchical approach provides an alternate to predict the structure of a protein when it is difficult to detect homologous protein sequences from protein databank (PDB). In this approach, intermediate states such as secondary structure states [6], [7], [8], super-secondary structures [9], [10], [11], turns [12], [13], [14], [15], [16], [17], C^α^-C^α^ distance bounds, backbone dihedral angle of proteins, etc. are used as restrains to assist the correct folding of protein backbone [18], [19], [20]. Recently, Kurgan *et al.* reviewed the progress in the field of intermediate state or one-dimension prediction [21]. It was observed that predicted secondary structure is useful in the prediction of disorder, flexible region, fold recognition and function prediction. It was also observed that dihedral angle (or backbone torsion angle) and secondary structures of a protein are highly correlated. In Ramachandran plot, phi-psi angles generally cluster around phi = −60°, psi = −40° for helix, phi = −120°, psi = 120° for beta-strand, and around phi = 60°, psi = 40° for L-helix [22]. Dihedral angle omega is almost fixed at 180°and 0° due to planarity of partial di-peptide bond [23]. Apart from Helix and Sheet, which have defined phi-psi region, coil residues are distributed in most of the Ramachandran plot. Strong correlations exist between the dihedral state of a residue and the immediate sequence neighbor [24]. This correlation helps in accurately defining the local ordering/confirmation in proteins. On the other hand, secondary structure predictions do not distinguish one loop conformation to another, but backbone dihedral angles accurately provide the local structural information that is useful in defining highly variable loop regions in a primary sequence. Backbone torsion angles significantly reduce the conformational search space for tertiary structure prediction. Thus, prediction of dihedral angle is especially useful for predicting tertiary structure of proteins.

Dihedral angle prediction has many applications in protein structure prediction that includes: (i) supplement for better secondary structure prediction [25], [26], [27], (ii) generation of multiple sequence alignment [28], [29], (iii) identification of protein folds [30], [31], [32] and (iv) fragment-free tertiary structure prediction [19]. Initially, dihedral prediction methods were developed for predicting few discrete states based on their distribution in Ramachandran plot [33], [34], [35], [36], [37], [38]. Wood *et al.* first developed a method for prediction of real values of dihedral angle psi and used this information for prediction of the protein secondary structure with high accuracy [26]. Later, Real-SPINE (1.0, 2.0 and 3.0), ANGLOR and TANGLE were developed to predict the real value of both phi and psi dihedral angle [39], [40], [41], [42], [43]. Real-SPINE was developed on a dataset of 2640 proteins with MAE of 54° for psi angle. The prediction was further improved in successive methods Real-SPINE 2.0 (38°/25°) for psi/phi angle respectively, Real-SPINE 3.0 (36°/22°), SPINE X (35° for psi) and SPINE XI (33.4° for psi) [44]. The new version of SPINE X incorporated the SPINE XI algorithm and it has MAE 33.4° equivalent to SPINE XI. In our study we have used the new version of SPINE X. ANGLOR and TANGLE were developed on a dataset of 1989 proteins and achieved an MAE of 46°/28° (ANGLOR), 44.6°/27.8° (TANGLE).

Presently, it is difficult to conclude which method among SPINE X, ANGLOR and TANGLE performs better than other, as these methods have been tested on different datasets. In this study, we have performed a benchmarking for principal prediction methods SPINE X and ANGLOR. These methods were evaluated on three different datasets; (i) SPINE X (2479 protein chains), (ii) ANGLOR (1989 protein chains), and (iii) a latest dataset from ccPDB (4682 protein chains) [40], [42], [45]. As the standalone of TANGLE method was not available, we were unable to benchmark TANGLE method on all datasets. Instead, we compared it with SPINE X and ANGLOR methods, only on the ANGLOR dataset on which TANGLE has reported its results. We have also analyzed why different algorithms perform differently just for few amino acids with respect to their secondary structure. We have also provided the raw data (prediction results of methods on different datasets) in an easily understandable text format, which can be downloaded from (http://crdd.osdd.net/raghava/download/rawdata.tgz).

## Materials and Methods

### Datasets Used for Evaluation

In this study, we evaluated the performance of different methods on datasets used in previous studies. In addition, we have also created new dataset from PDB using ccPDB server.

Following is the description of these datasets: -.

#### SPINE X dataset

This dataset contains 2479 protein chains that were obtained from SPINE X server (http://sparks.informatics.iupui.edu/SPINE-X/list.spinex.tgz). [40].

#### ANGLOR dataset

We obtained this dataset from ANGLOR web site available at URL http://zhanglab.ccmb.med.umich.edu/ANGLOR/benchmark.html. Out of the total chains, 500 chains were used as training data, 460 as validation data and 1029 as testing data [42].

#### ccPDB Dataset

We created new dataset using the database cum web server ccPDB “compilation and creation of datasets from PDB” (http://crdd.osdd.net/raghava/ccpdb) [45]. We extracted those protein chains from ccPDB that satisfy following three criteria’s i) protein chains having resolution better than 2A°, ii) Rfree less than 0.25 and iii) number of residues in each chain between 50 to 3000. We created a non-redundant dataset having sequence identity cut-off 30% with 4682 protein chains. This dataset was named accordingly to its sequence identity level *i.e.* ccPDB30 dataset, which consists of chains having sequence identity less than 30%. The list of PDB IDs used in ccPDB30 dataset is provided in Table S1 of File S2. For more information on PDB chains sequence identity level, please refer to (ftp://resources.rcsb.org/sequence/clusters). We obtained the dihedral angle of all PDB chains using DSSP software [46].

### Dihedral Angle Prediction Methods

#### SPINE X

The method utilizes a guided-learning artificial neural network for prediction of dihedral angle. In the first step, sequence profile, seven representative physical parameters and secondary structure were used as input to predict the normalized solvent accessibility value of a residue. The normalized solvent accessibility value was combined with the above stated input features to predict the real value dihedral angles. This method is then combined with a discrete state classifier to improve the accuracy of predicted angles. The resulting predicted angles were further refined with a conditional random field model to give the final predicted angles. The method is available at http://sparks.informatics.iupui.edu/SPINE-X/index.html.

#### ANGLOR

The method is a composite machine-learning algorithm using neural network for phi angle prediction and Support Vector Machine (SVM) for psi angle prediction. In the first step, sequence profile is used to predict secondary structure and solvent accessibility value of a residue. In the next step, three features: sequence profile, secondary structure and solvent accessibility were used as input vector to predict dihedral angles. The method is available at http://zhanglab.ccmb.med.umich.edu/ANGLOR/.

#### TANGLE

This method is based on two level prediction using SVM based regression approach. In the first level, features derived from sequence (PSSM profiles, secondary structure, solvent accessibility, native disorder, sequence length and sequence weight) are used as input to predict initial dihedral angles. The predicted dihedral angles from first level are used as input in the second level to predict the final refined dihedral angles. TANGLE is available at http://sunflower.kuicr.kyoto-u.ac.jp/~sjn/TANGLE/webserver.html.

#### Performance Evaluation

We used Mean Absolute Error (MAE) as described by Wu *et al.* [42], for assessing the prediction of phi/psi angles throughout the study. According to Wu *et al.* the MAE is defined as the average difference in degrees between the predicted (P) and the experimental values (E) of all residues. MAE measures the accuracy for continuous variables e.g. dihedral angles and is the standard practice of evaluation of dihedral angle prediction methods. [39], [40], [41], [42], [43]. MAE is defined by the following formulae:
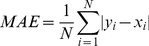
(1)where, *x_i_* and *y_i_* are the actual (observed) and predicted dihedral angles of the *i^th^* residue and *N* is the total number of residues.

To test whether the obtained MAE difference while comparing the methods is statistically significant, we applied Wilcoxon signed rank test using coin package [47] in R statistical programming language [48] to calculate the *p*-value for the comparison. We also reported Root Mean Square Error (RMSE) and Pearson correlation coefficient (PCC) achieved by all the methods on all the datasets. However, it should be kept in mind that in assessing the quality of prediction of dihedral angles, PCC appears to be a less robust measure [40], [41], [42]. RMSE and PCC are defined by the following formula:
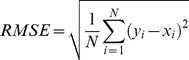
(2)

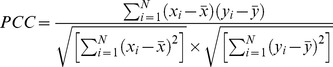
(3)where *x_i_* and *y_i_* are the actual (observed) and predicted dihedral angles of the *i^th^* residue; 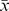 and 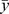 are the mean values of *x* and *y*, and *N* is the total number of residues.

As the nature of the data is circular, we calculated the difference between actual and predicted/mean dihedral angle as per Wu *et al.* [42] for calculating both RMSE and PCC.

## Results

### Evaluation of Existing Methods

We evaluated the performance of existing methods on different datasets used in the past for developing prediction method. In addition, the performance of existing methods was also evaluated on new or independent dataset generated in this study. We also performed amino acid specific random based prediction as described by Wu *et al.* [42] and Song *et al.* [39] to perform the base line comparison of the methods with a random method. Wu *et al.* took the dihedral angles randomly from amino acid specific pool obtained using training dataset of 500 proteins and repeated this random process 10,000 times to get a stable distribution. We also adopted the same process for random prediction. On SPINE X and ccPDB30 datasets, the whole respective dataset was used for amino acid specific pool generation to obtain random prediction. The performance of these methods on various datasets is described below:

#### ANGLOR dataset

First, we evaluated the performance of methods on ANGLOR dataset. As shown in Table 1, for dihedral angle phi, ANGLOR, TANGLE and SPINE X achieved MAE of 28.20°, 27.80° and 24.83°, respectively between actual and predicted phi. These results show that SPINE X performs better than other methods. SPINE X achieved MAE of 56.70° and 9.63° for glycine and proline, which is much better than ANGLOR (75.1° and 15.2°) and TANGLE (84.1° and 13.6°). Both SPINE X and TANGLE performed better than ANGLOR in case of serine and threonine residues. TANGLE performed relatively better than other two methods for helix forming residues. The result shows that SPINE X performs better among all methods, but the difference between all three methods is less than 4° (Table 1). In case of prediction of psi angle, SPINE X performed better for almost all residues, especially for glycine and proline residues. ANGLOR, TANGLE and SPINE X have MAE 46.40°, 44.64° and 38.80° respectively (Table 2). Again, TANGLE performed better than other methods for helix forming residues. The above results clearly indicate that SPINE X is outperforming other two methods by a margin of around 6°. The MAE of SPINE X for phi and psi angles on this dataset is significantly smaller than ANGLOR with a *p*-value of <<0.001 and <<0.001 respectively, using Wilcoxon signed rank test. With respect to random prediction (Table 3), both ANGLOR and SPINE X performed significantly better with MAE difference 16.5° (*p*-value<<0.001) and 19.9° (*p*-value<<0.001) for phi and 40.8° (*p*-value<<0.001) and 48.0° (*p*-value<<0.001) for psi respectively. For both phi and psi dihedral angles, SPINE X has high PCC than ANGLOR, TANGLE (as reported) and random prediction. SPINE X has least RMSE in predicting phi dihedral angle (Table S2, S3 in File S2).

**Table 1:** Comparison of performance of SPINE X, ANGLOR and TANGLE, in terms of MAE, on different datasets for the prediction of phi dihedral angle.

Datasets	ANGLOR			SPINE X		ccPDB30	
Residue	ANGLOR	TANGLE	SPINE X	ANGLOR	SPINE X	ANGLOR	SPINE X
**ALA**	22.5	21.9	20.7	18.3	16.4	18.2	16.6
**CYS**	27.7	25.5	25.7	24.9	22.9	24.9	22.6
**ASP**	30.8	29.7	27.6	26.0	22.9	26.2	23.2
**GLU**	23.3	22.3	21.3	18.7	16.9	18.7	17.0
**PHE**	24.4	23.6	22.6	22.4	20.2	22.6	20.6
**GLY**	75.1	84.1	56.7	69.5	48.9	69.9	50.6
**HIS**	31.8	29.6	28.7	28.2	25.4	28.5	26.0
**ILE**	18.1	17.5	17.0	15.8	14.4	15.7	14.4
**LYS**	25.6	24.8	23.4	21.1	18.9	21.4	19.2
**LEU**	18.3	17.8	17.3	15.5	14.3	15.4	14.2
**MET**	22.4	22.0	25.7	18.1	20.6	18.6	20.2
**ASN**	37.6	37.1	33.6	33.7	29.6	34.0	30.5
**PRO**	15.2	13.6	9.6	14.4	8.6	14.0	8.2
**GLN**	25.1	23.9	22.8	20.6	18.5	20.7	18.8
**ARG**	25.0	23.5	22.5	21.6	19.3	21.6	19.5
**SER**	32.3	30.6	29.1	26.0	23.4	25.6	23.5
**THR**	26.0	23.9	22.9	22.6	19.4	22.5	19.4
**VAL**	20.1	19.1	18.6	17.6	15.7	17.6	15.7
**TRP**	23.1	22.8	22.3	21.5	19.9	21.8	20.7
**TYR**	25.3	23.7	23.4	23.3	20.9	23.5	21.2
**ALL**	28.2	27.8	24.8	24.3	20.8	24.5	21.2
**Helix (H)**	11.0	9.9	11.0	9.5	9.3	9.5	9.6
**Sheet (E)**	27.9	26.1	23.4	27.4	22.4	27.4	22.6
**Coil (C)**	41.8	40.8	36.4	36.9	31.2	36.5	31.1

First row show the name of dataset and second row show the name of methods.

**Table 2:** Comparison of performance of SPINE X, ANGLOR and TANGLE, in terms of MAE, on different datasets for the prediction of psi dihedral angle.

Datasets	ANGLOR			SPINE X		ccPDB30	
Residue	ANGLOR	TANGLE	SPINE X	ANGLOR	SPINE X	ANGLOR	SPINE X
**ALA**	42.7	38.2	34.0	39.2	28.0	40.4	29.9
**CYS**	48.7	45.0	45.4	44.6	38.4	44.4	39.0
**ASP**	48.9	48.7	45.1	46.0	40.1	46.7	42.4
**GLU**	43.1	39.1	35.1	39.4	29.3	41.1	32.1
**PHE**	40.8	39.4	35.7	40.0	33.0	40.4	34.3
**GLY**	66.9	76.7	52.7	65.2	46.8	65.3	48.0
**HIS**	48.2	46.4	45.5	44.3	37.9	45.1	41.1
**ILE**	35.3	32.1	28.9	33.7	25.1	34.6	26.6
**LYS**	45.6	41.8	38.6	42.0	32.5	43.3	34.6
**LEU**	38.1	35.2	31.6	35.2	27.1	36.3	28.9
**MET**	40.9	36.5	36.2	38.0	29.9	39.3	33.0
**ASN**	45.9	45.2	46.4	43.4	42.4	44.2	45.2
**PRO**	61.3	59.3	45.7	58.6	38.2	59.1	40.6
**GLN**	43.0	39.4	36.0	40.1	31.0	41.2	33.6
**ARG**	44.1	40.9	36.9	40.9	31.5	41.6	33.5
**SER**	55.4	53.5	46.2	52.6	39.5	53.6	42.3
**THR**	51.1	50.4	40.4	49.5	37.2	50.1	39.3
**VAL**	37.6	34.8	30.3	35.3	26.6	36.0	27.9
**TRP**	43.5	41.6	36.3	41.8	33.0	43.0	35.9
**TYR**	42.3	40.1	36.4	40.9	33.1	41.8	35.3
**ALL**	46.0	44.6	38.8	43.5	33.5	44.5	35.7
**Helix (H)**	28.2	18.7	19.2	26.9	16.4	28.0	18.0
**Sheet (E)**	39.9	38.9	29.7	40.4	28.3	40.9	30.1
**Coil (C)**	63.9	66.0	58.8	61.6	53.4	61.8	55.3

First row show the name of dataset and second row show the name of methods.

**Table 3:** Performance of random prediction method, in terms of MAE, on ANGLOR, SPINE X and ccPDB30 datasets for the prediction of phi and psi dihedral angle.

	Random PHI Prediction			Random PSI prediction		
Residue/Dataset	ANGLOR	SPINE X	ccPDB30	ANGLOR	SPINE X	ccPDB30
**ALA**	40.4	34.3	33.7	83.6	82.3	83.0
**CYS**	44.6	42.7	42.2	88.5	88.7	88.3
**ASP**	47.8	42.2	41.5	84.8	83.2	83.6
**GLU**	40.3	33.2	33.3	83.6	78.9	80.8
**PHE**	43.9	40.6	40.5	88.0	89.5	89.4
**GLY**	88.5	87.8	88.2	87.3	88.1	88.2
**HIS**	49.2	46.7	46.7	89.6	88.7	87.1
**ILE**	34.9	32.9	32.5	88.1	88.5	88.1
**LYS**	44.0	38.1	38.4	85.9	84.4	85.6
**LEU**	34.3	30.5	30.2	87.9	85.4	86.2
**MET**	46.8	40.7	39.2	88.5	86.7	87.7
**ASN**	59.5	55.6	56.4	83.8	81.8	81.0
**PRO**	14.0	13.2	12.4	87.7	87.7	87.4
**GLN**	42.2	37.3	37.7	84.8	81.2	84.3
**ARG**	42.9	39.2	39.4	86.0	85.2	86.3
**SER**	49.7	42.8	42.1	89.7	89.9	89.7
**THR**	41.4	36.8	35.7	89.0	89.8	88.6
**VAL**	37.7	34.5	34.0	86.7	86.9	86.0
**TRP**	40.4	38.2	38.8	90.1	88.7	89.0
**TYR**	42.2	41.4	40.6	89.6	89.3	89.2
**ALL**	44.7	40.4	40.2	86.8	85.8	86.1
**Helix (H)**	36.3	32.0	32.0	82.7	78.4	80.5
**Sheet (E)**	44.9	44.2	43.2	90.7	93.7	92.2
**Coil (C)**	51.1	46.4	45.9	87.9	88.2	87.5

#### SPINE X dataset

Next, we evaluated the performance of methods on SPINE X dataset. SPINE X achieved MAE of 20.8° and performed better than ANGLOR with MAE 24.31° for phi angle. The results were more pronounced for glycine, proline, serine and threonine residues. (Table 2). The same trend follows in case of psi angle; SPINE X performed better for glycine, proline, serine and threonine having MAE 46.8°, 38.17°, 39.49° and 37.18° as compared to ANGLOR with MAE 65.17°, 58.59°, 52.6°, and 49.46° respectively. Overall ANGLOR achieved MAE of 43.52° and SPINE X achieved 33.5° (Table 2). It is evident from the results that SPINE X performs better than ANGLOR, especially in case of psi angle. The difference of MAE between SPINE X and ANGLOR for phi (3.5°) and psi (10°) angles on this dataset, corresponds to a *p*-value of <<0.001 using Wilcoxon signed rank test. Both ANGLOR and SPINE X performed significantly better than amino acid specific random prediction method (Table 3) with MAE difference of 16.1° (*p*-value<<0.001) and 19.6° (*p*-value<<0.001) for phi and 42.3° (*p*-value<<0.001) and 52.3° (*p*-value<<0.001) for psi, respectively. SPINE X has highest PCC as compared to ANGLOR and random prediction for phi and psi dihedral angles (Table S2, S3 in File S2).

#### ccPDB30 Dataset

We also evaluated the performance of SPINE X and ANGLOR on independent ccPDB30 dataset. For dihedral angle phi, SPINE X achieved MAE of 21.23° and ANGLOR achieved 24.46°. SPINE X performed much better for glycine and proline having MAE 19.33° and 5.8°, which is lower than ANGLOR. Similarly, in case of psi angle, SPINE X achieved MAE 17.29° and 18.45°, which is lower than ANGLOR for glycine and proline residues respectively. SPINE X having MAE of 35.70° performed much better than ANGLOR with MAE of 44.48°. The results clearly demonstrate the superior performance of SPINE X over ANGLOR (Table 1, 2). Using Wilcoxon signed rank test, the MAE difference between SPINE X and ANGLOR for phi angle (3.3°) corresponds to a *p*-value<<0.001 and for psi angle (8.8°) *p*-value<<0.001. Both SPINE X and ANGLOR performed significantly better than random prediction (Table 3) with *p*-values (phi<<0.001; psi<<0.001) and (phi<<0.001; psi<<0.001) respectively. SPINE X has least RMSE and highest PCC for phi dihedral angle on this dataset (Table S2, S3 in File S2).

### Effect of Angle Shifting in SPINE X

**Figure 1:**
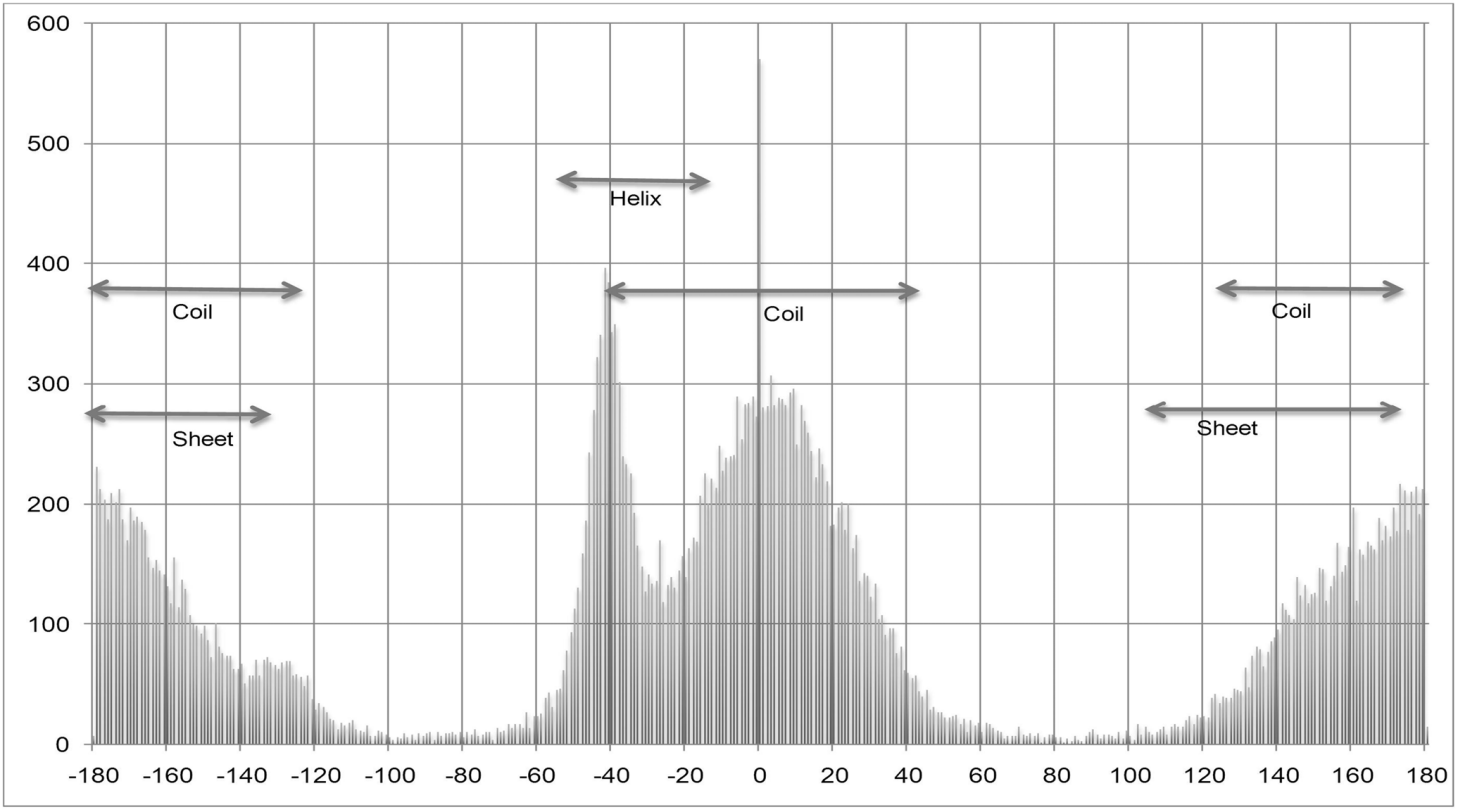
Normal psi angle distribution of glycine.

**Figure 2:**
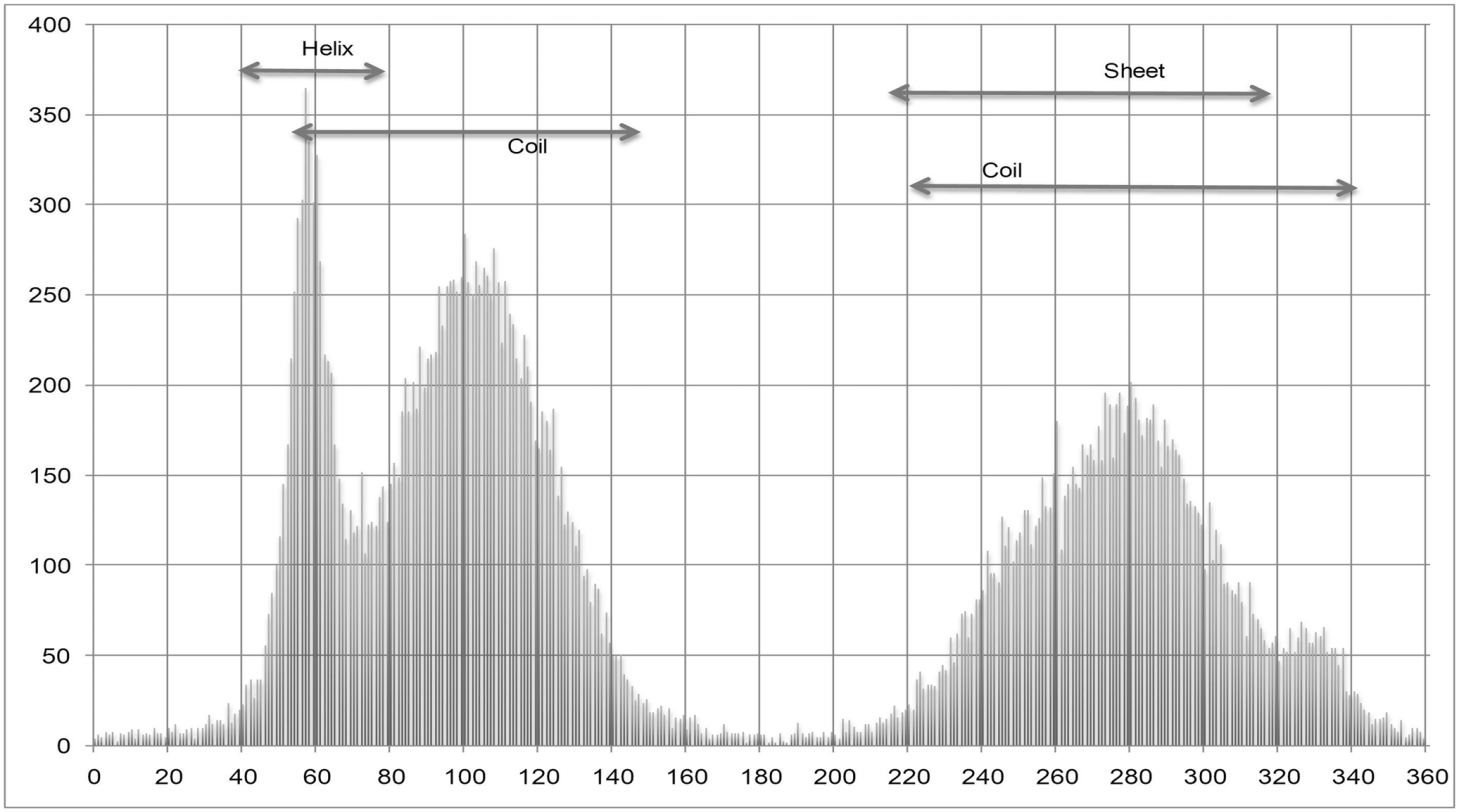
Psi angle distribution of glycine after shifting the angles.

The results suggest that SPINE X performs better than ANGLOR and TANGLE for the prediction of psi angle. Amino acid wise comparison reveals that SPINE X performs better than ANGLOR and TANGLE especially in glycine, proline, serine and threonine amino acids. Interestingly, both glycine and proline do not follow the standard Ramachandran plot. In case of glycine of ccPDB30 dataset, ANGLOR achieved MAE of 65.32° and SPINE X has 48.03° for psi angle. It has been observed that distribution of psi angle for glycine in helix region has a range between −55° to −10°, sheet ranges from −180° to −130°, 110° to 180° and coil occurs mainly in −180° to −120°, −45° to 45° and 130° to 180° as shown in Figure 1. SPINE X shifted the angles by adding 100° to the angles between −100° and 180° and adding 460° to the angles between −180° and −100°, thus shifting the angles from −180° –180° to 0° −360° (Figure 2). SPINE X authors have suggested that this shifting ensures that a minimum number of angles occur at the end of the sigmoidal function, making the data more linear and continuous, which ultimately improves the learning by machine learning algorithms. To prove that shifting the angles actually work or not, we developed two models, one without angle shifting and other with angle shifting using SPINE X dataset. It was observed that the model developed with shifted angles has 10° lower MAE as in case of glycine (data not shown). We also observed that shifting the phi dihedral angle improved the MAE in case of glycine.

**Figure 3:**
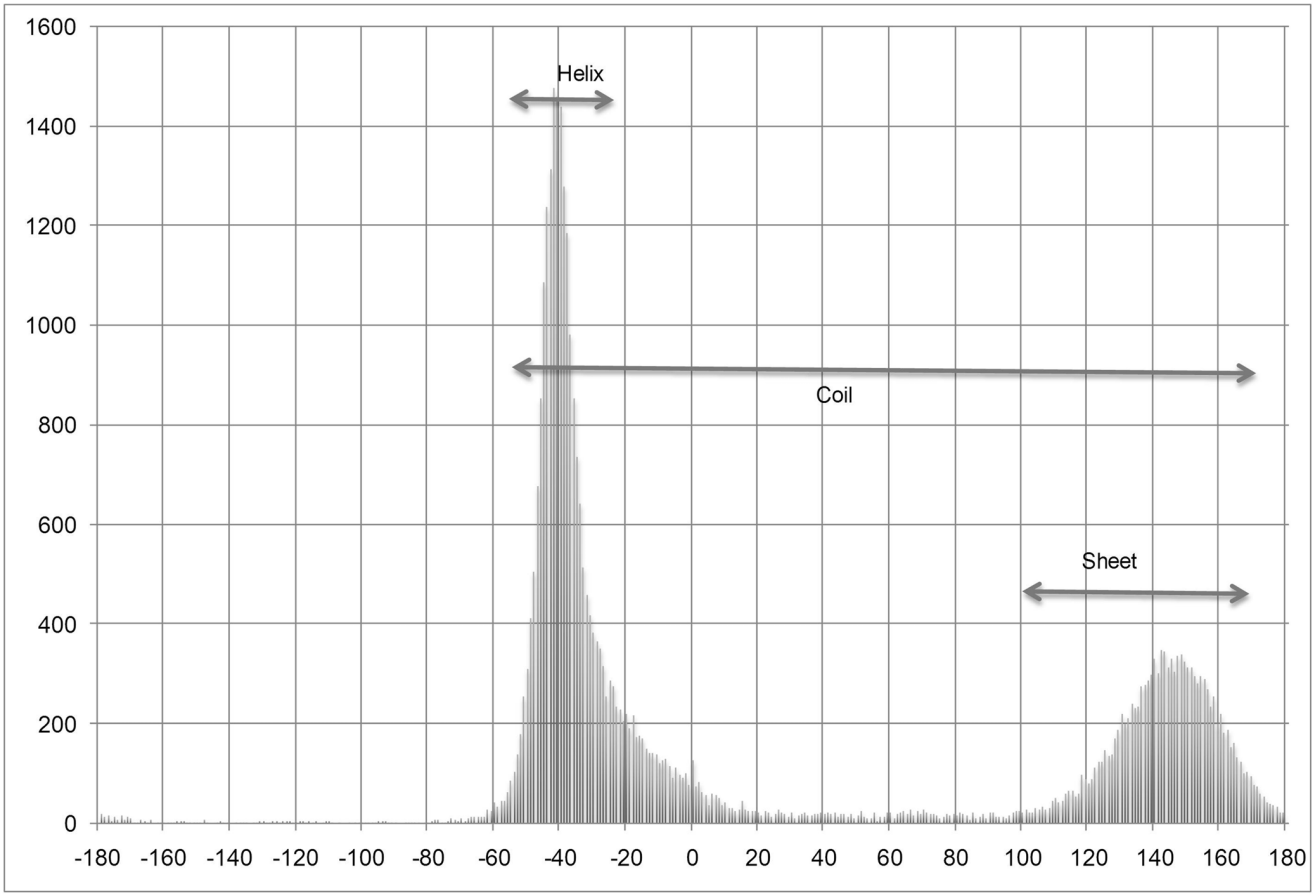
Normal psi angle distribution of Alanine.

There are amino acids in which angle shifting does not increase the performance because they have minimal residues in the −100° to −180° ranges. Thus shifting of angles makes no difference as in the case of alanine (Figure 3). For graphs showing the dihedral angles distribution of all 20 amino acids, please refer to File S1 and complete details are found in (Table S4, S5 in File S2). We have also observed in the developed models on SPINE X dataset that angle shifting produce negligible difference for alanine (data not shown).

## Discussion

One of the advantages of prediction of dihedral angles of residues over secondary structure state is that they can be effectively used as restraints for building tertiary structure of proteins. In the past, methods were developed to predict real value of dihedral angles of residues in a protein. The assessment of the performance of a method/technique plays a vital role in the development of any field of science. It is important for users as well as developers, since it allow users to find the best method for their work and for the developers to compare their method with existing methods. In this study, an attempt has been made to assess the performance of existing methods in the field of dihedral prediction. We benchmarked the performance of SPINE X and ANGLOR in this study. The performance of these methods was evaluated on datasets used in the past as well as on new dataset called independent dataset generated using ccPDB server. TANGLE method was compared with ANGLOR and SPINE X on only ANGLOR dataset because of its reported results on this dataset and its unavailability as standalone for benchmarking on other datasets. Among various performance measures like PCC, MAE and RMSE, MAE is the most widely used measure for accessing the performance of dihedral angle prediction. The reason behind this is the circular nature of dihedral angles while PCC is used to measure linear dependence between observed and predicted values. Therefore, angles predicted near the border (e.g. observed angle 175° and predicted angle −175°) are actually close to each other (MAE 10°) but will lead to irregular correlation coefficient. It was observed that SPINE X performed better than rest of the methods, especially for psi angle. The angle shifting performed by SPINE X for training, improves the psi dihedral angle prediction considerably. The angle shifting improves results only for those amino acids, which have considerable number of residues in −100° to −180° range. We also observed that angle shifting of phi angle, especially for glycine, improves the prediction performance. The dihedral angle prediction performance can be improved if amino acid specific dihedral angle shifting is done based upon the amino acid dihedral angle distribution to make the training data linear and continuous.

## Supporting Information

File S1 (PDF)

File S2 Contains the following files: **Table S1.** PDB IDs of 4682 PDB chains used in ccPDB30 dataset. **Table S2.** Pearson correlation coefficient (PCC) of methods on different datasets. Methods are represented by rows and datasets are represented by columns respectively. **Table S3.** Root-mean-square-error (RMSE) of methods on different datasets. Methods are represented by rows and datasets are represented by columns respectively. **Table S4.** Distribution of phi angle for 20 amino acids. **Table S5.** Distribution of psi angle for 20 amino acids.
(DOC)
