# Supplementary material for: Evaluation of Protein Dihedral Angle Prediction Methods
Source: PLoS One. 2014 Aug 28;9(8):e105667. doi: 10.1371/journal.pone.0105667 (PMC4148315; doi:10.1371/journal.pone.0105667)
Supplement: File S1 — (PDF) [file pone.0105667.s001.pdf]

Phi Angle Distribution

X-axis shows angle distribution

Y-axis shows the number of residues

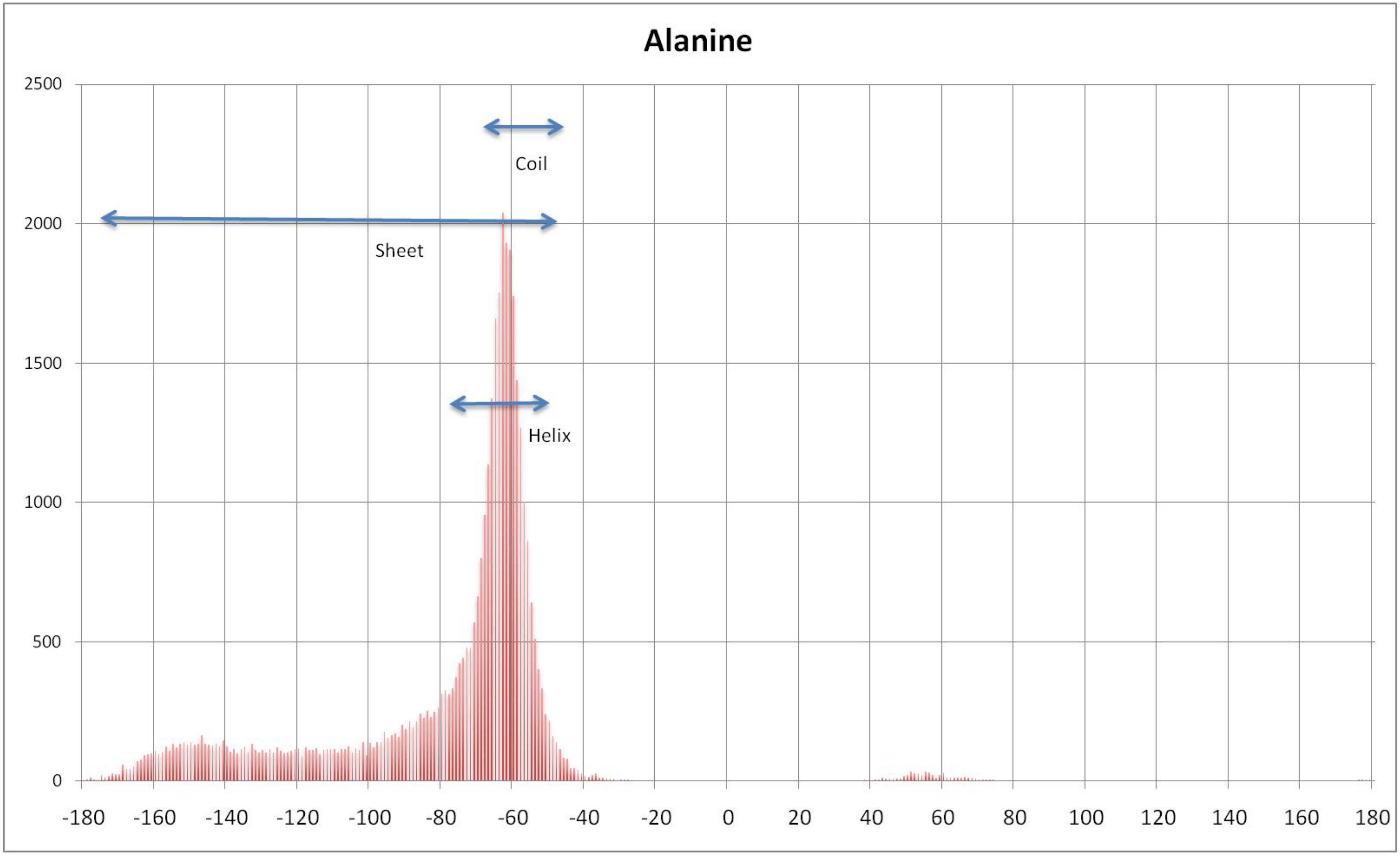

Alanine
Coil
Sheet
Helix
2500
2000
1500
1000
500
0
-180
-160
-140
-120
-100
-80
-60
-40
-20
0
20
40
60
80
100
120
140
160
180

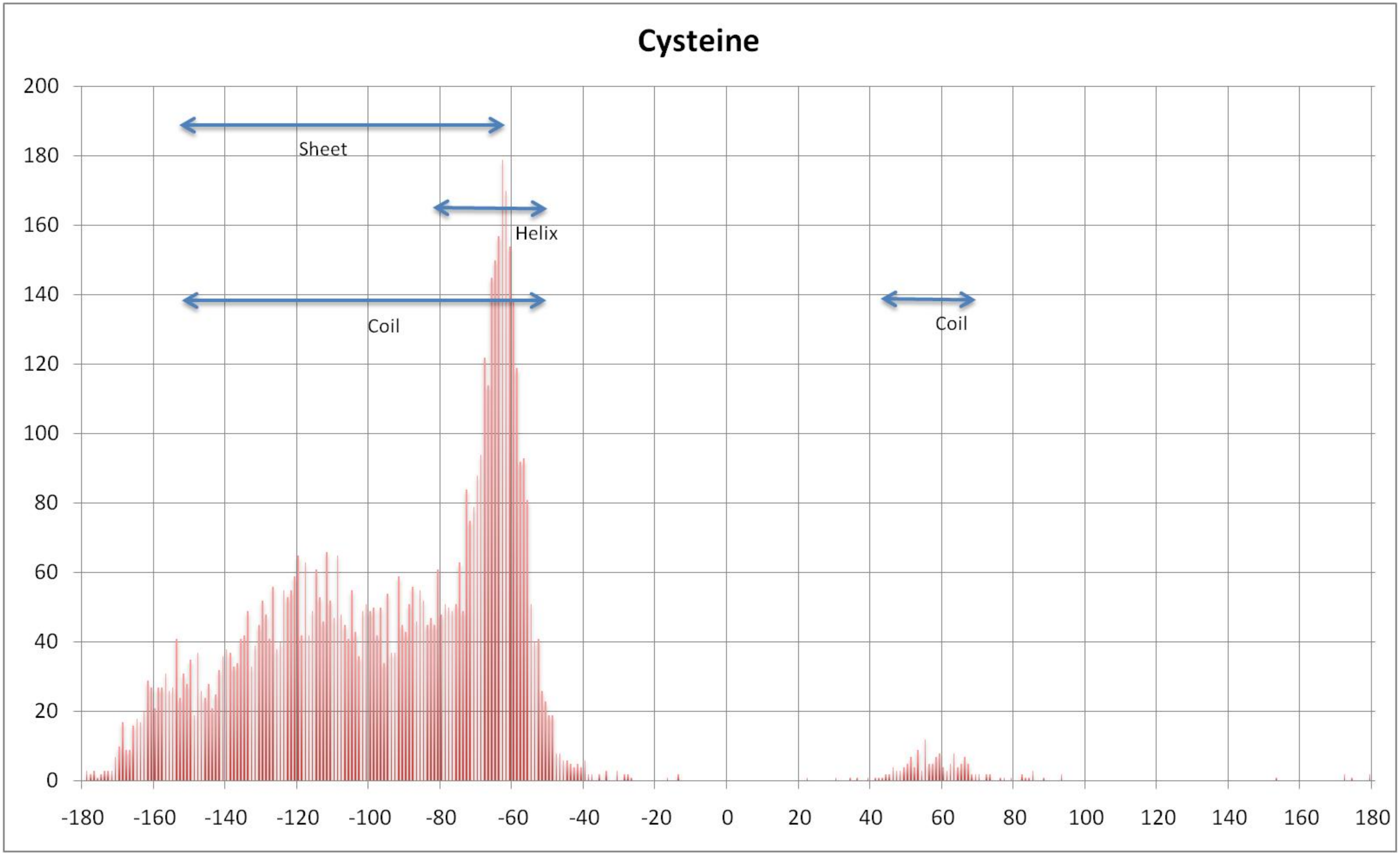

Cysteine
200
180
160
140
120
100
80
60
40
20
0
Sheet
Helix
Coil
Coil
-180
-160
-140
-120
-100
-80
-60
-40
-20
0
20
40
60
80
100
120
140
160
180

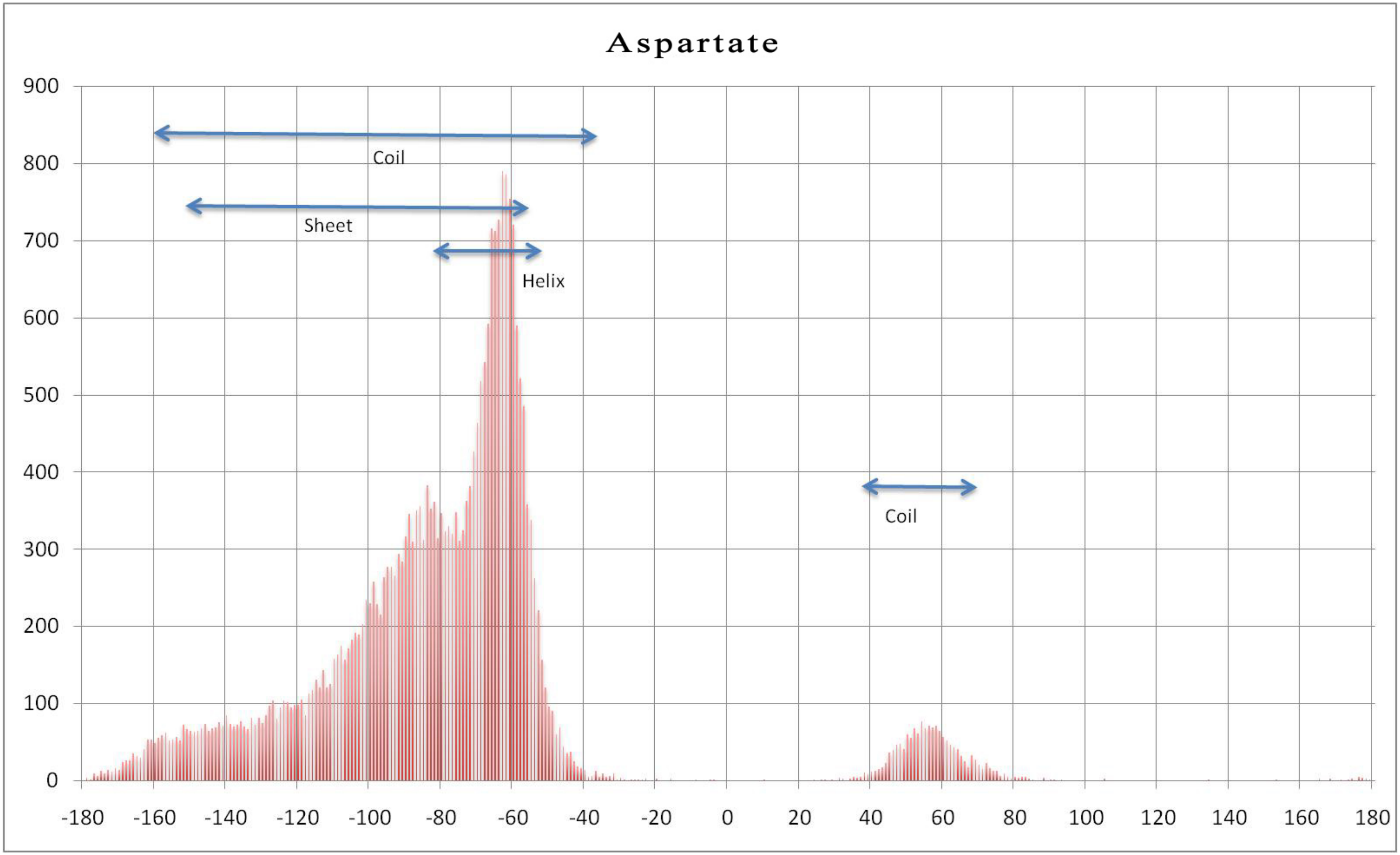
Aspartate
900
800
700
600
500
400
300
200
100
0
Coil
Sheet
Helix
Coil
-180
-160
-140
-120
-100
-80
-60
-40
-20
0
20
40
60
80
100
120
140
160
180

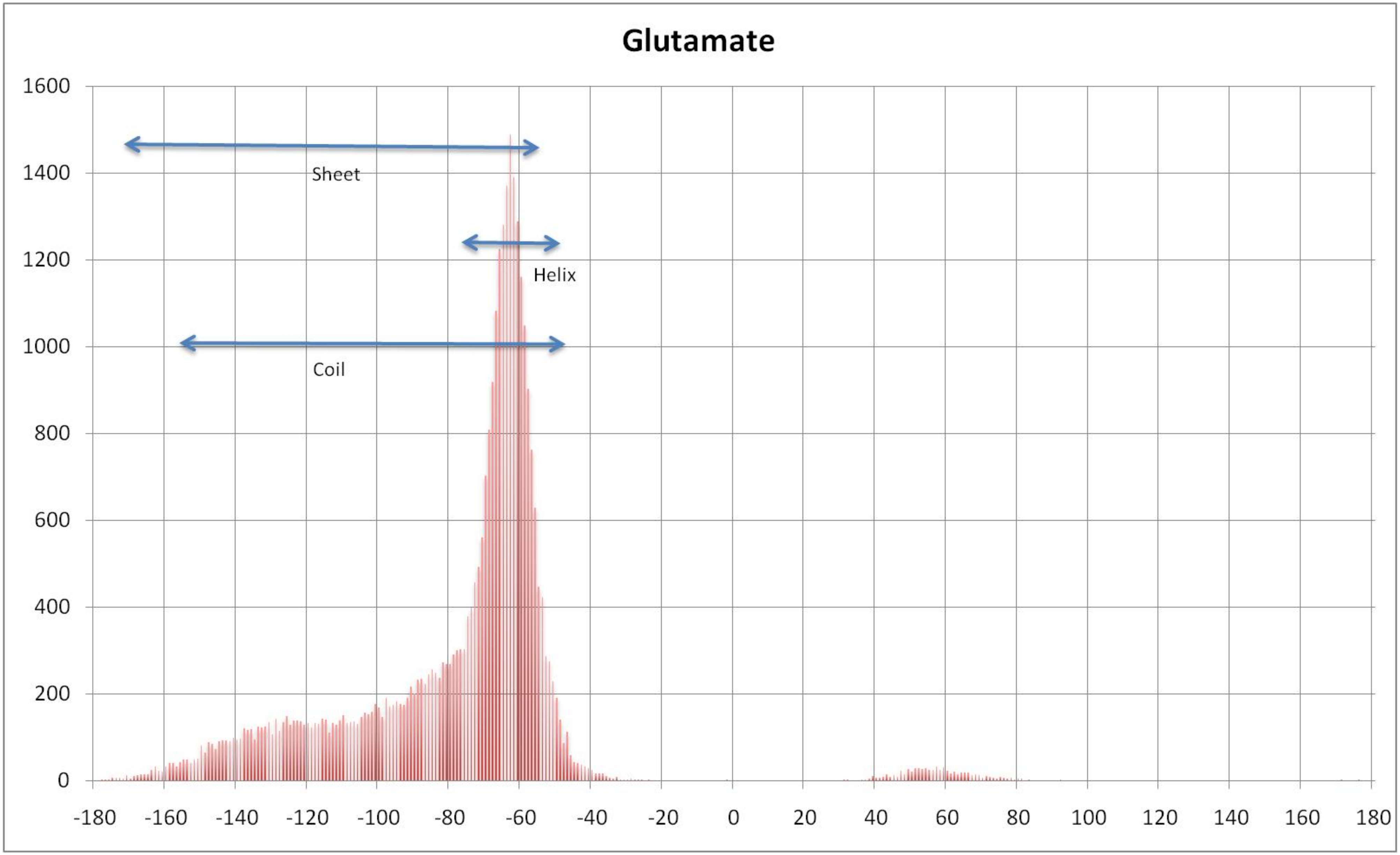
Glutamate
Sheet
Helix
Coil
1600
1400
1200
1000
800
600
400
200
0
-180
-160
-140
-120
-100
-80
-60
-40
-20
0
20
40
60
80
100
120
140
160
180

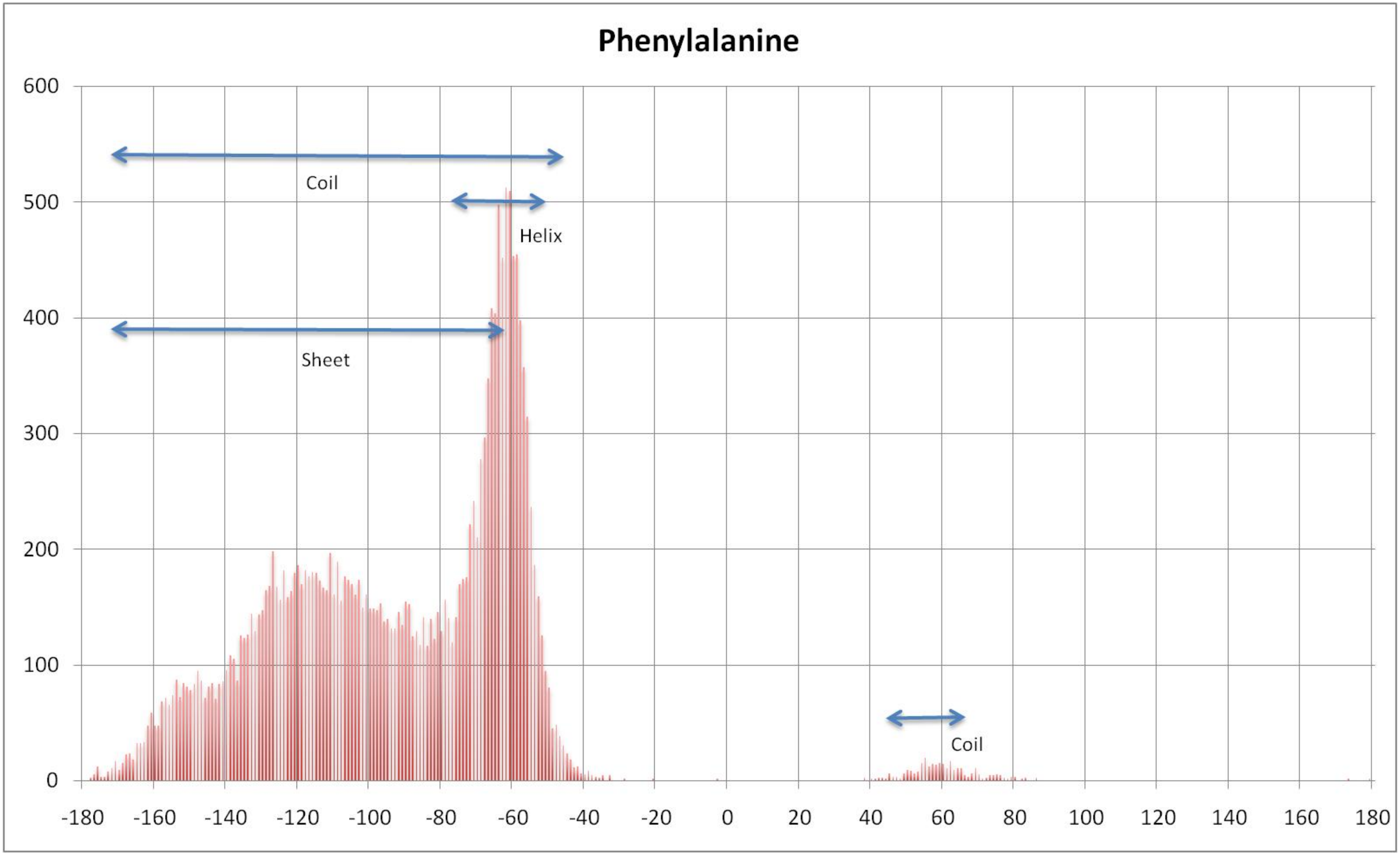
Phenylalanine
Coil
Helix
Sheet
Coil
600
500
400
300
200
100
0
-180 -160 -140 -120 -100 -80 -60 -40 -20 0 20 40 60 80 100 120 140 160 180

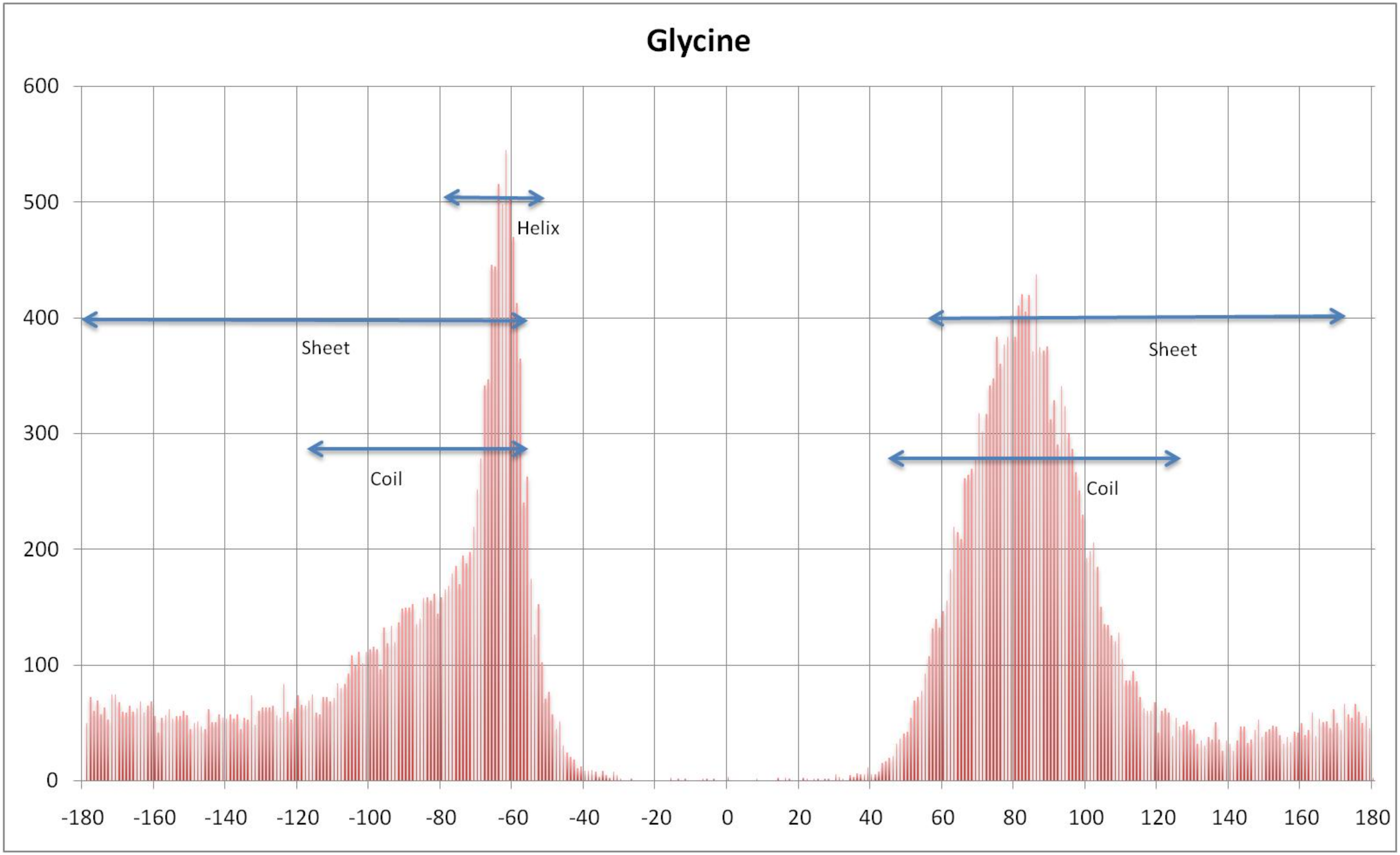
Glycine
Helix
Sheet
Coil
Sheet
Coil
600
500
400
300
200
100
0
-180
-160
-140
-120
-100
-80
-60
-40
-20
0
20
40
60
80
100
120
140
160
180

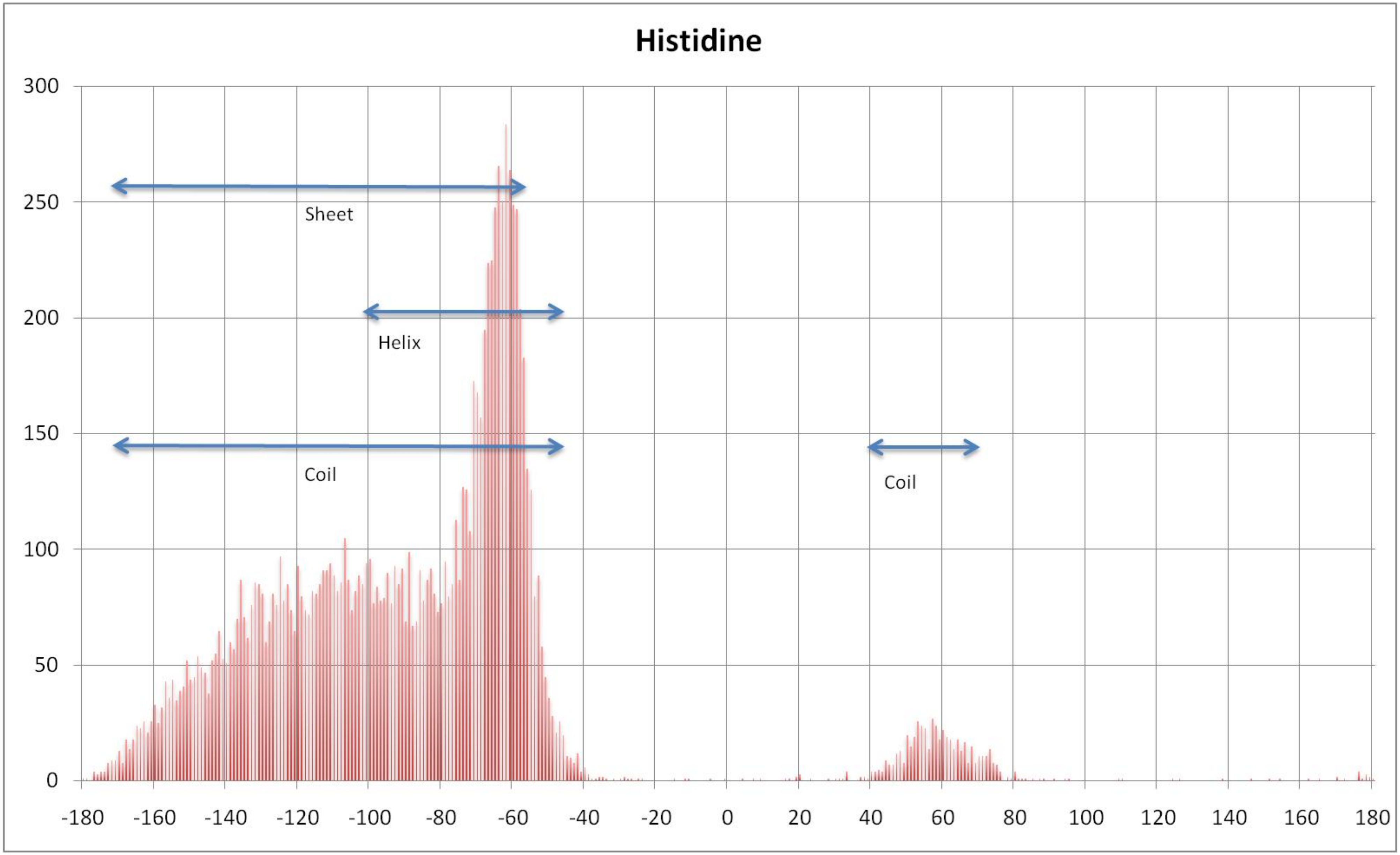
Histidine
Sheet
Helix
Coil
Coil
300
250
200
150
100
50
0
-180
-160
-140
-120
-100
-80
-60
-40
-20
0
20
40
60
80
100
120
140
160
180

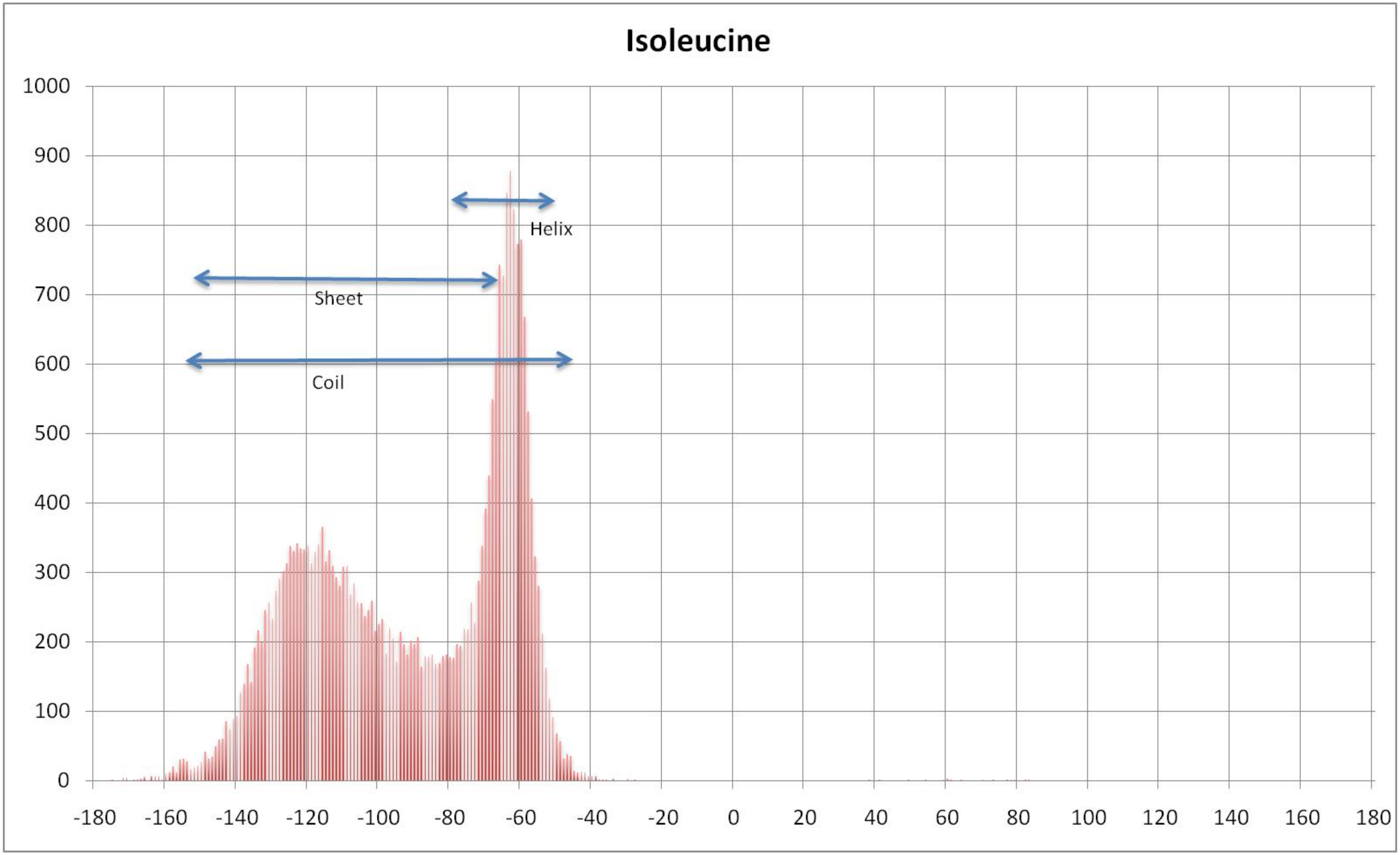
Isoleucine
Helix
Sheet
Coil
1000
900
800
700
600
500
400
300
200
100
0
-180
-160
-140
-120
-100
-80
-60
-40
-20
0
20
40
60
80
100
120
140
160
180

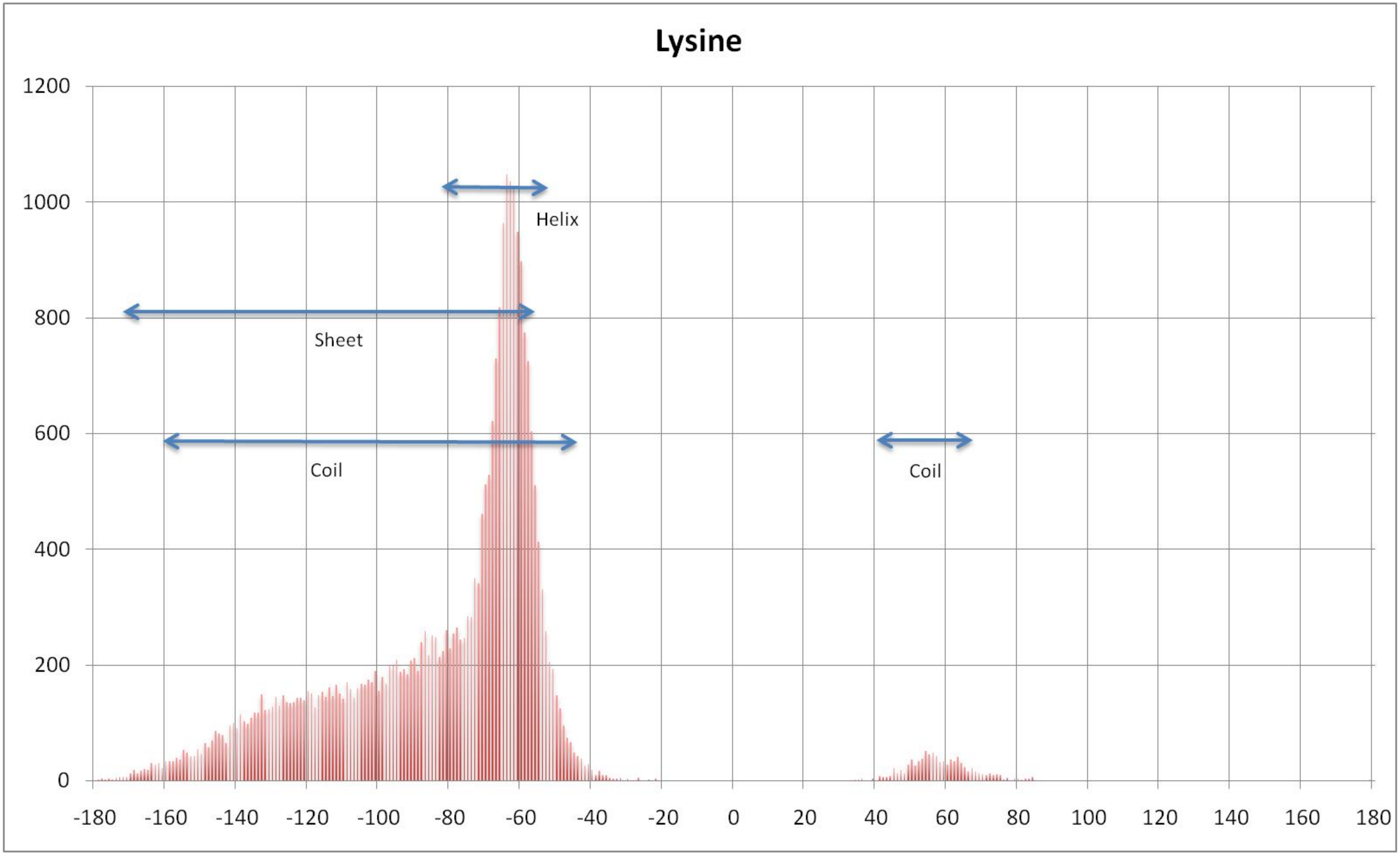
Lysine
Helix
Sheet
Coil
Coil
1200
1000
800
600
400
200
0
-180
-160
-140
-120
-100
-80
-60
-40
-20
0
20
40
60
80
100
120
140
160
180

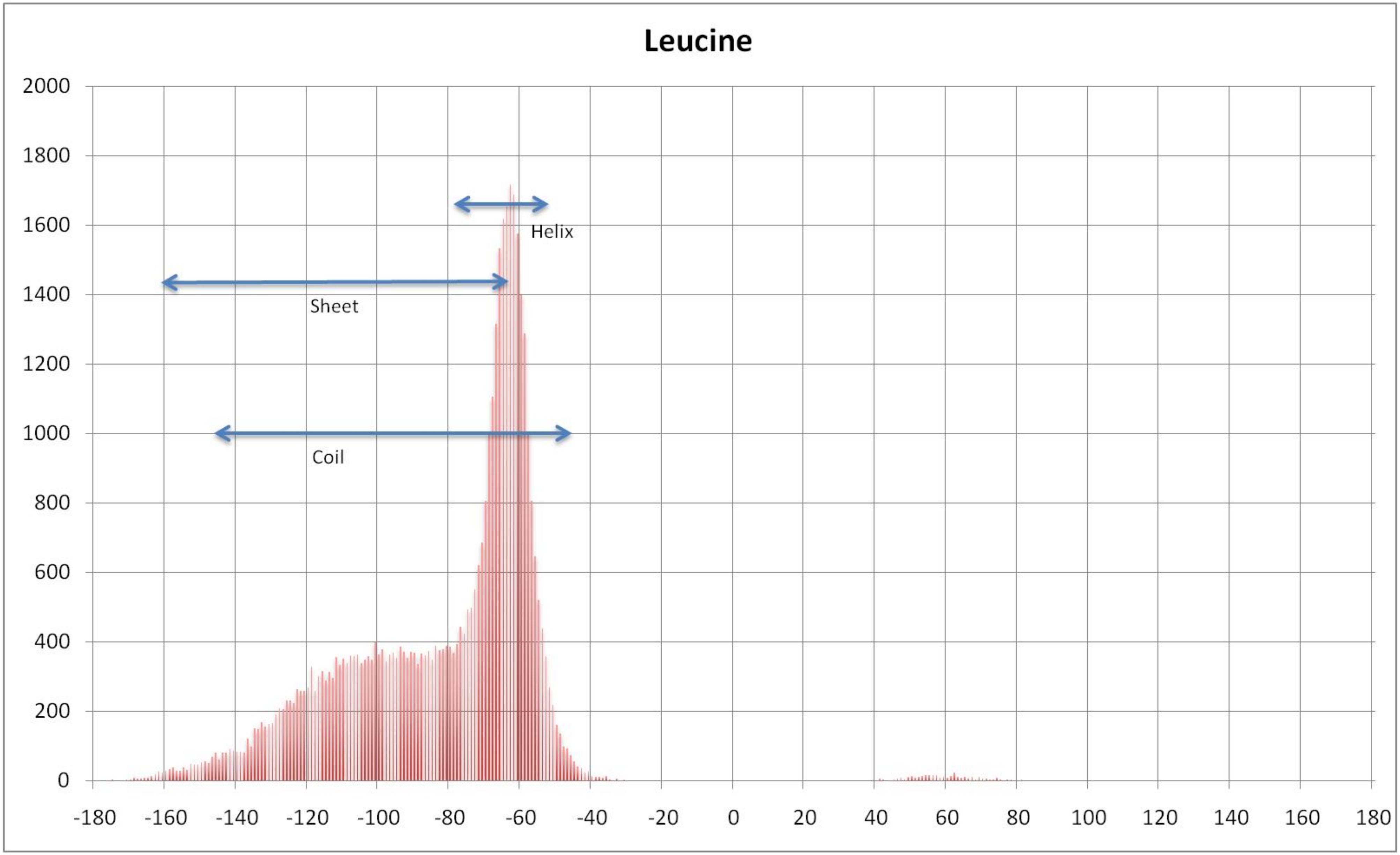

Leucine
Helix
Sheet
Coil
2000
1800
1600
1400
1200
1000
800
600
400
200
0
-180
-160
-140
-120
-100
-80
-60
-40
-20
0
20
40
60
80
100
120
140
160
180

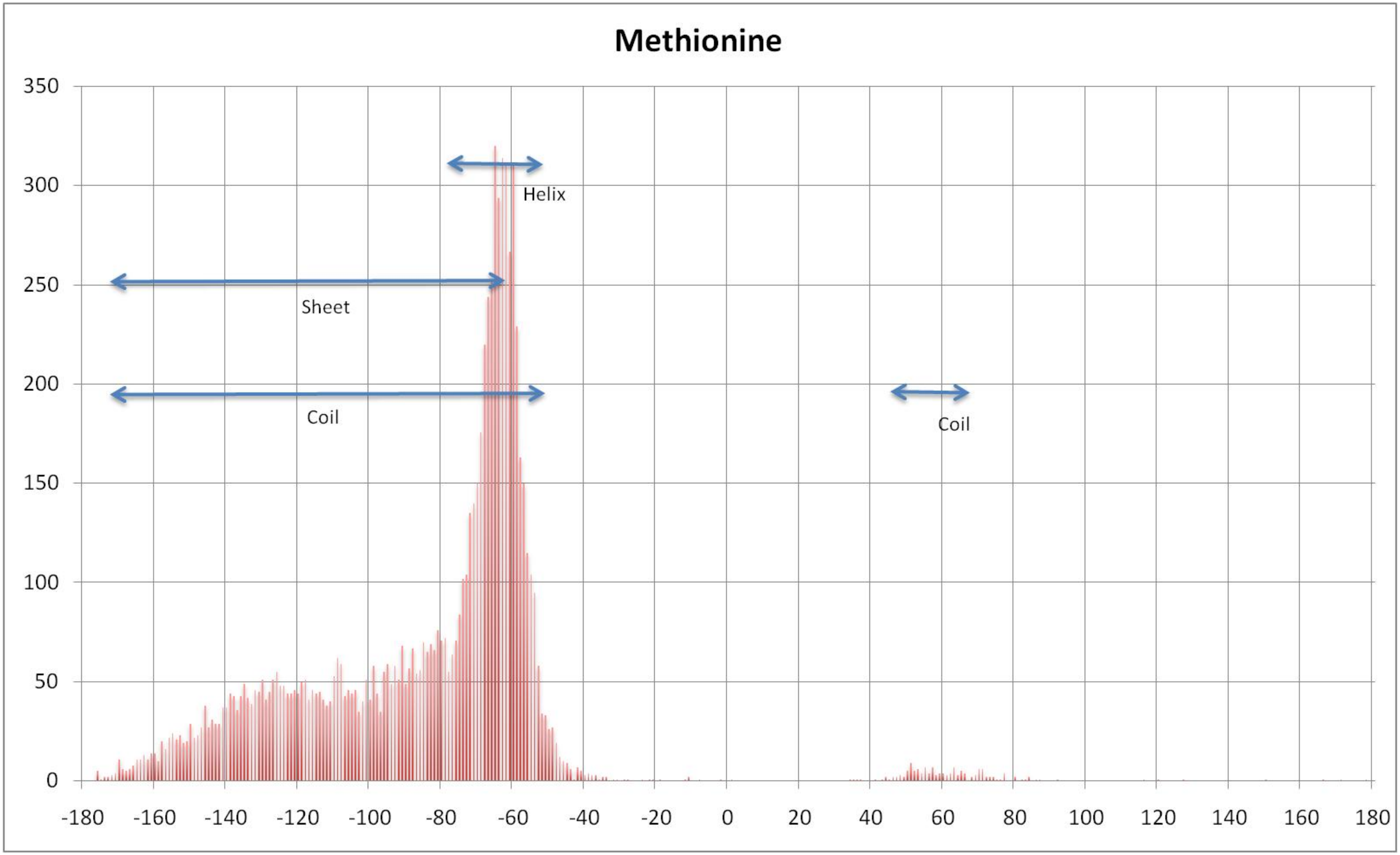
Methionine
350
300
250
200
150
100
50
0
-180
-160
-140
-120
-100
-80
-60
-40
-20
0
20
40
60
80
100
120
140
160
180
Helix
Sheet
Coil
Coil

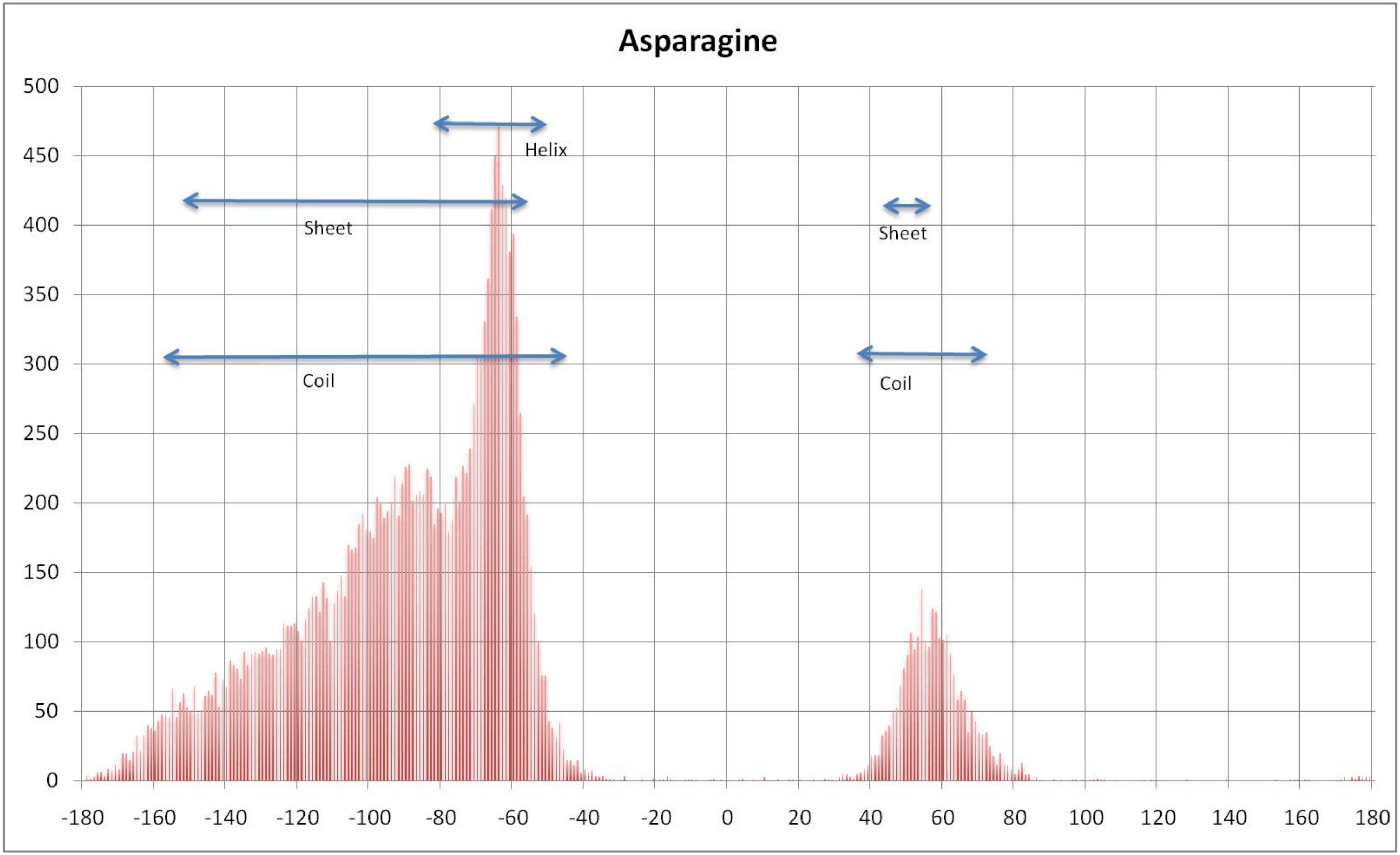

Asparagine
Helix
Sheet
Sheet
Coil
Coil
500
450
400
350
300
250
200
150
100
50
0
-180
-160
-140
-120
-100
-80
-60
-40
-20
0
20
40
60
80
100
120
140
160
180

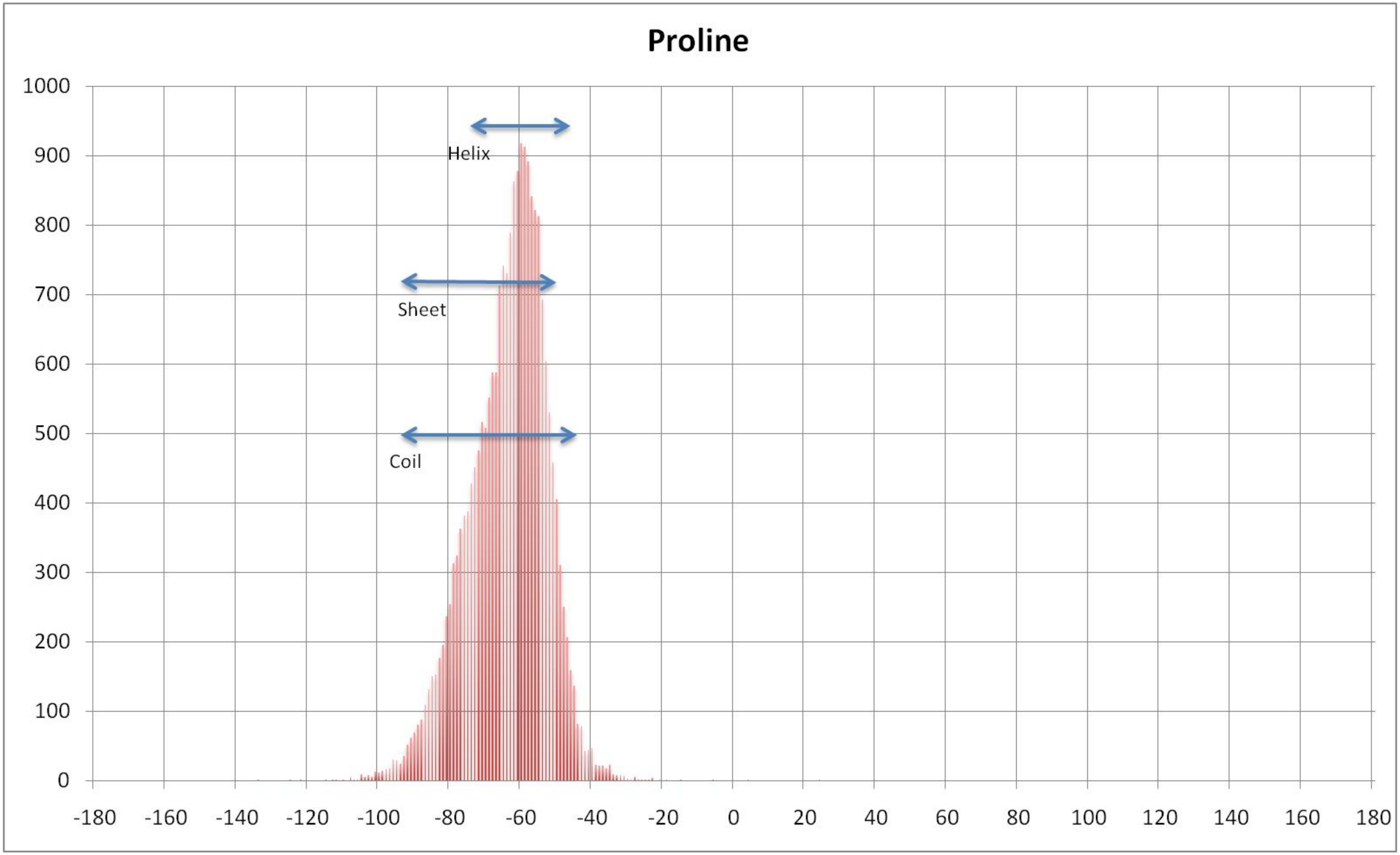

Proline
Helix
Sheet
Coil
1000
900
800
700
600
500
400
300
200
100
0
-180
-160
-140
-120
-100
-80
-60
-40
-20
0
20
40
60
80
100
120
140
160
180

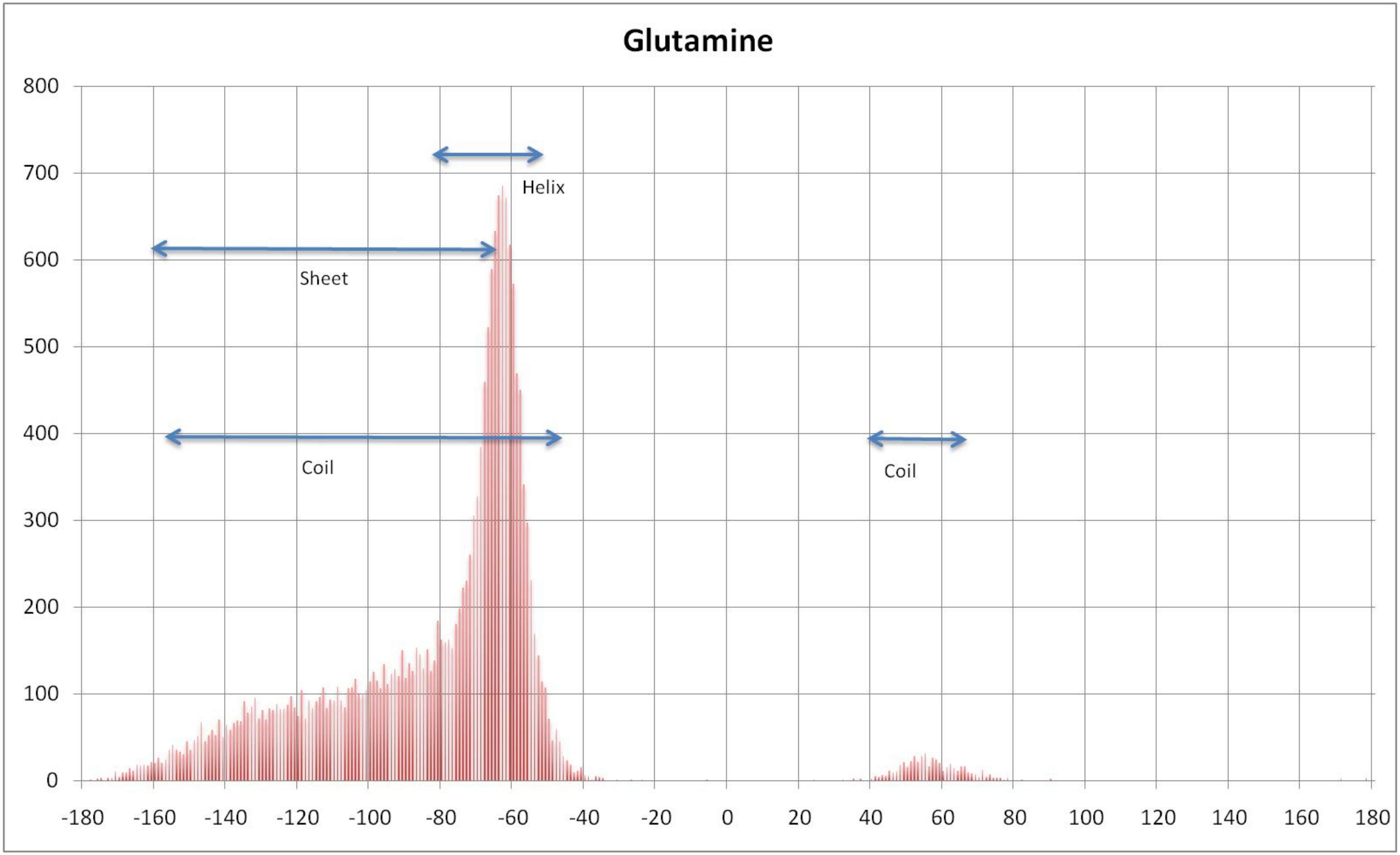

Glutamine
Helix
Sheet
Coil
Coil
800
700
600
500
400
300
200
100
0
-180
-160
-140
-120
-100
-80
-60
-40
-20
0
20
40
60
80
100
120
140
160
180

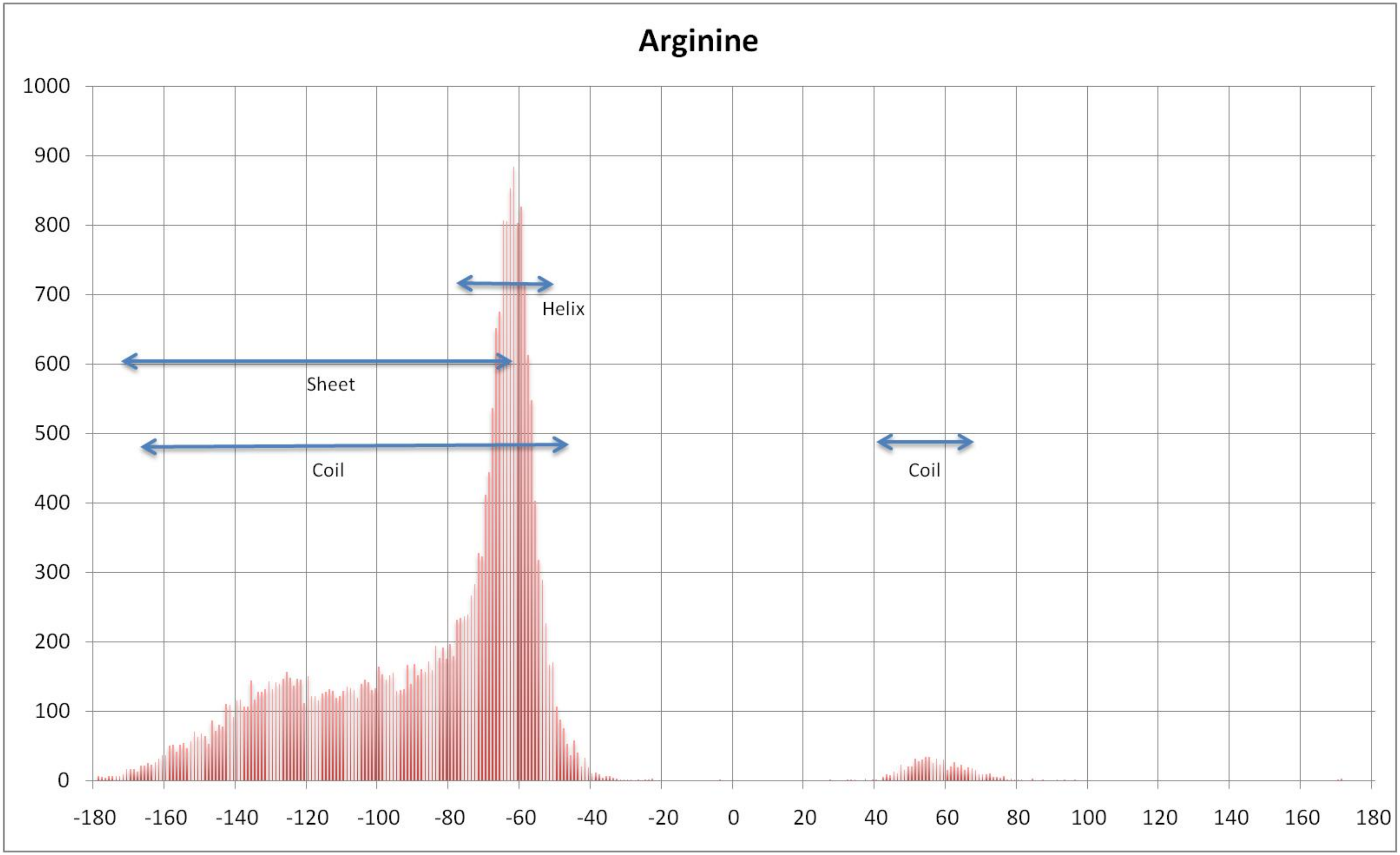
Arginine
Helix
Sheet
Coil
Coil
1000
900
800
700
600
500
400
300
200
100
0
-180
-160
-140
-120
-100
-80
-60
-40
-20
0
20
40
60
80
100
120
140
160
180

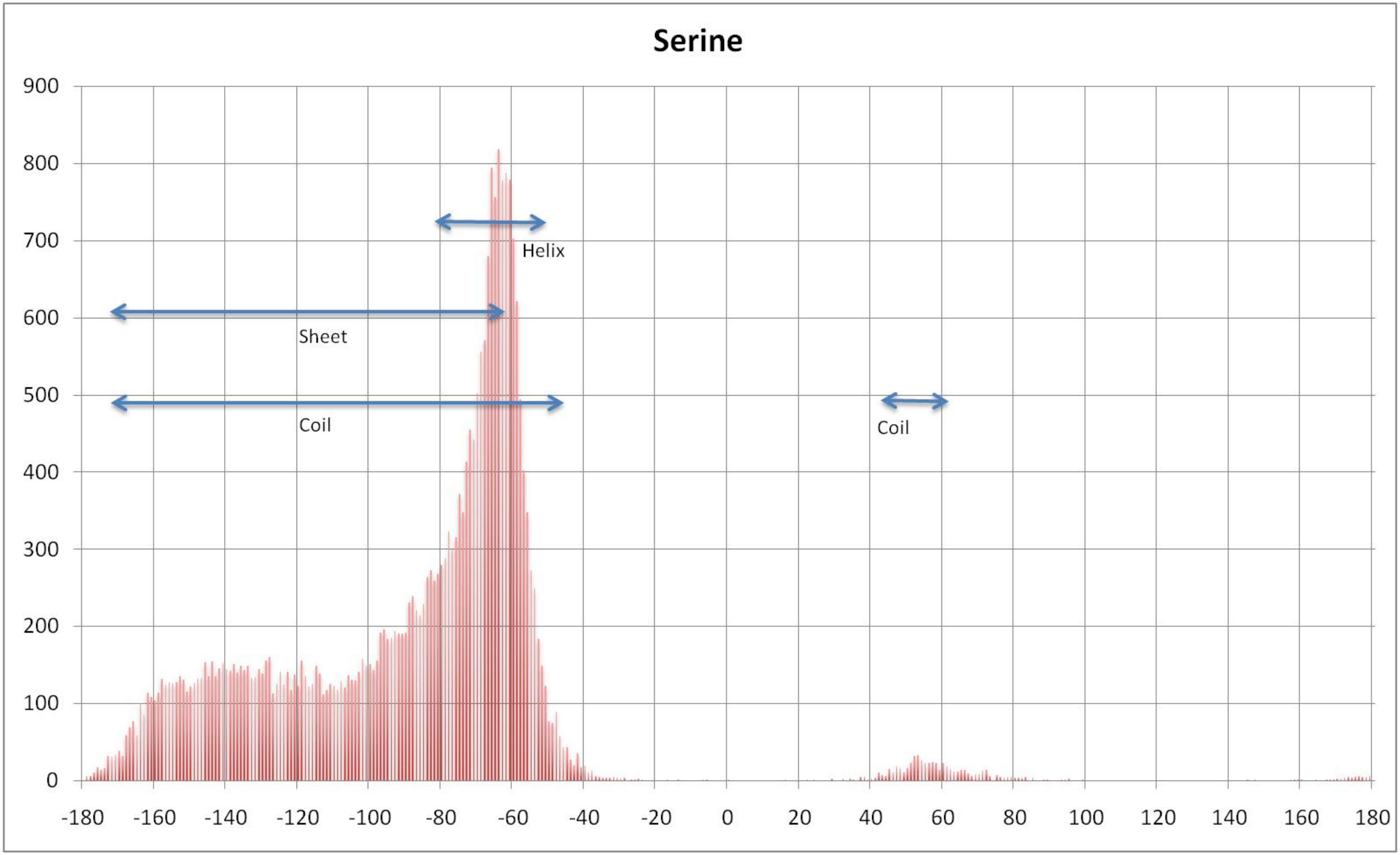

Serine
Helix
Sheet
Coil
Coil
900
800
700
600
500
400
300
200
100
0
-180
-160
-140
-120
-100
-80
-60
-40
-20
0
20
40
60
80
100
120
140
160
180

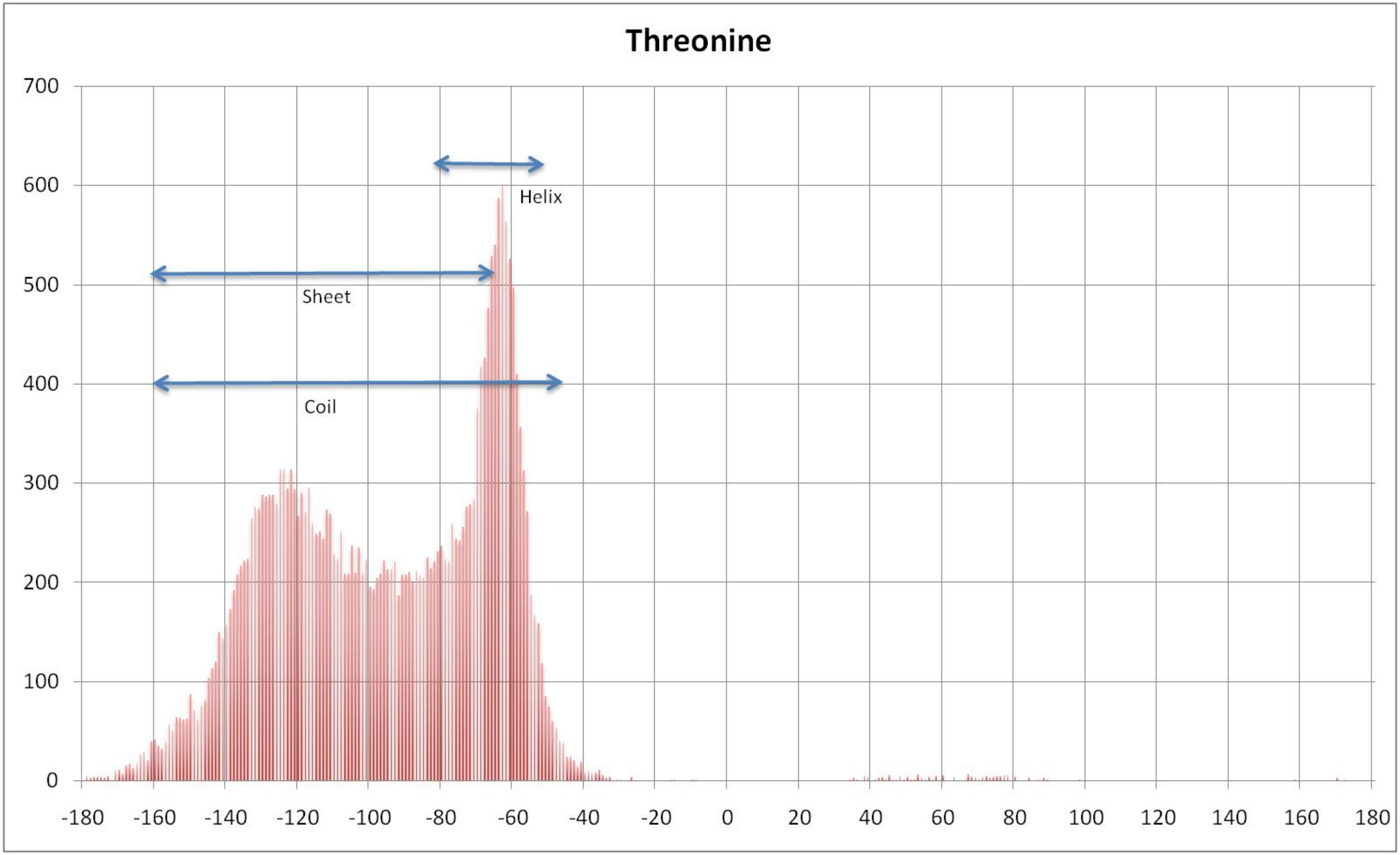
Threonine
700
600
500
400
300
200
100
0
-180
-160
-140
-120
-100
-80
-60
-40
-20
0
20
40
60
80
100
120
140
160
180
Helix
Sheet
Coil

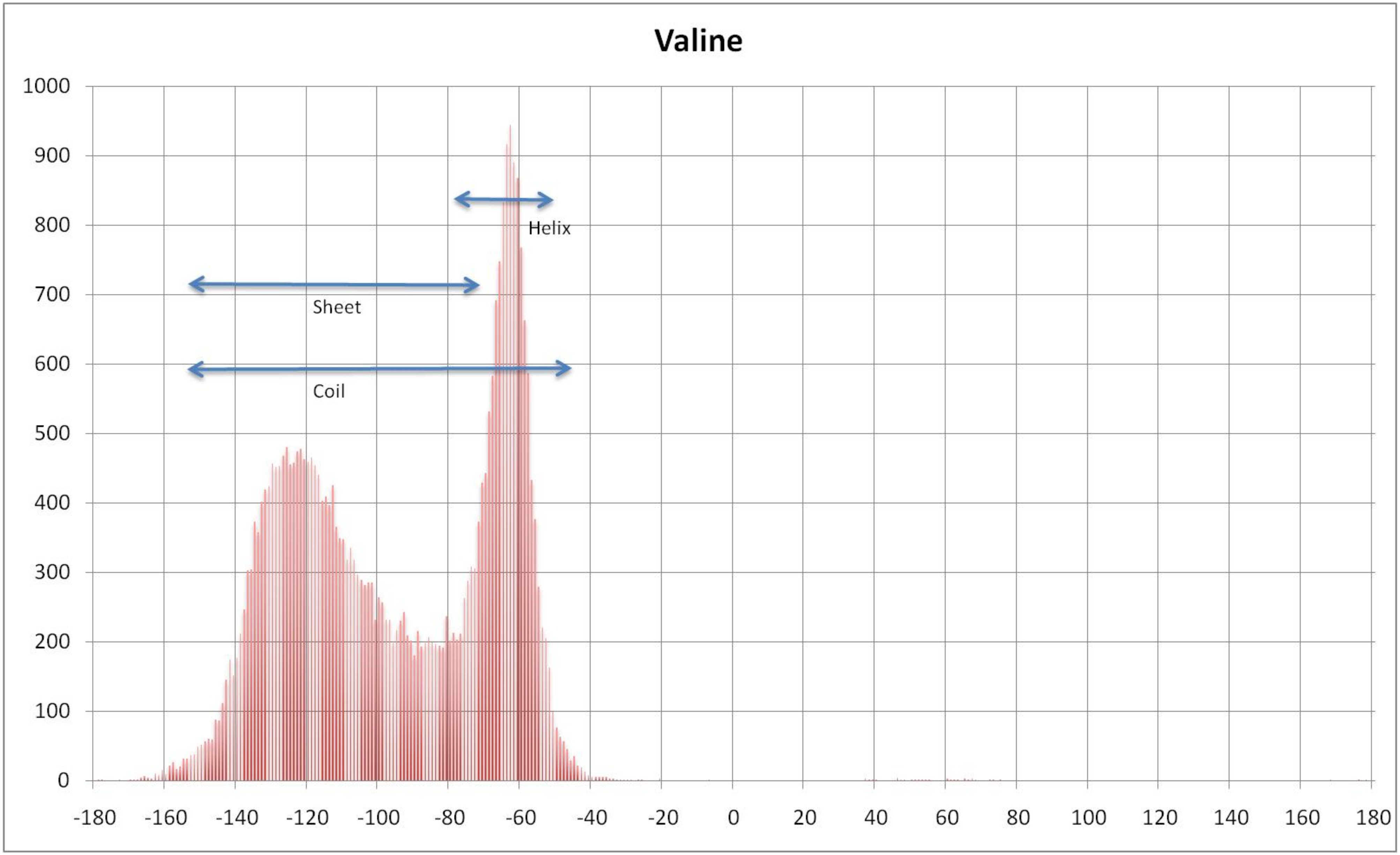
Valine
1000
900
800
700
600
500
400
300
200
100
0
-180
-160
-140
-120
-100
-80
-60
-40
-20
0
20
40
60
80
100
120
140
160
180
Helix
Sheet
Coil

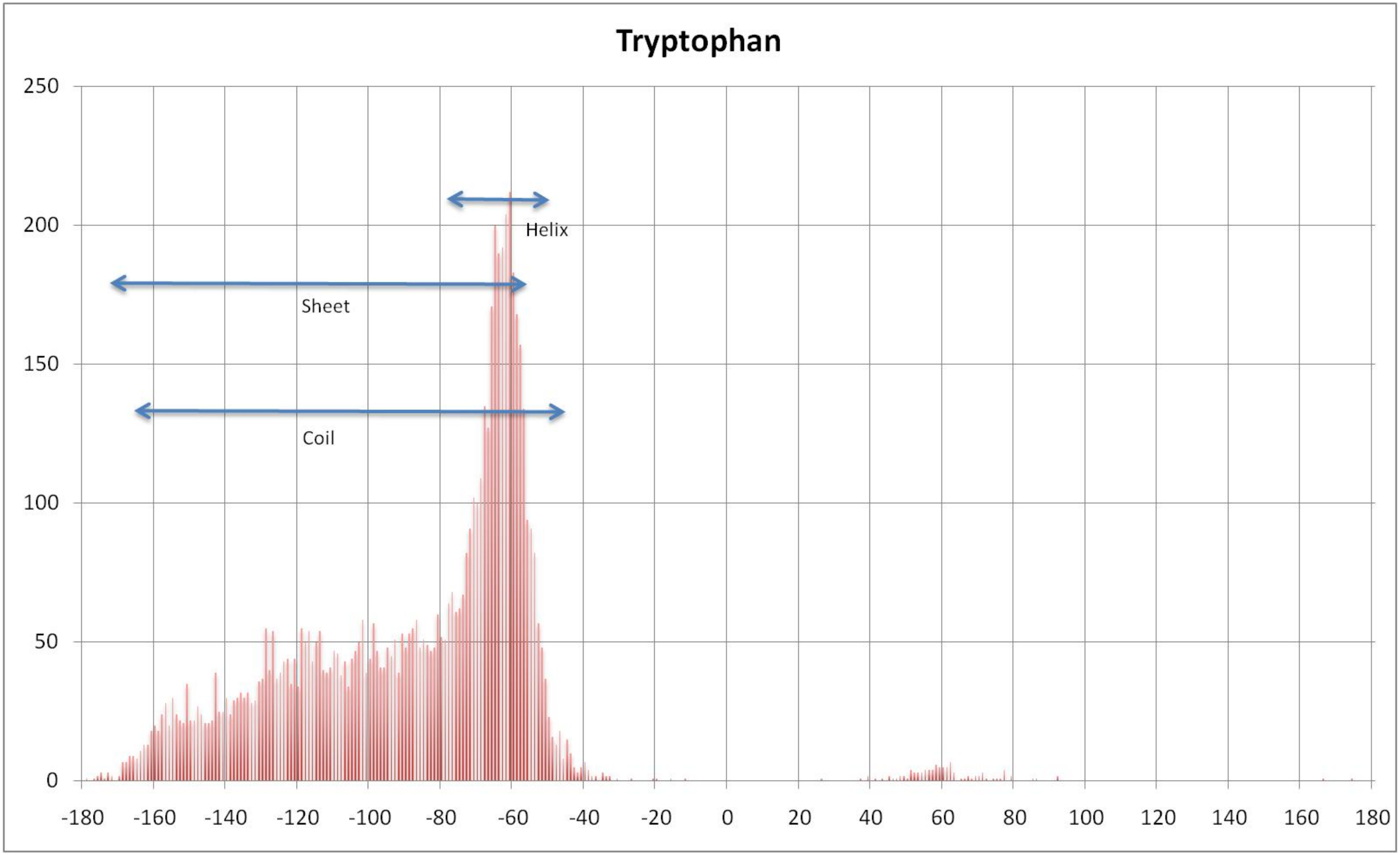
Tryptophan
250
200
150
100
50
0
-180
-160
-140
-120
-100
-80
-60
-40
-20
0
20
40
60
80
100
120
140
160
180
Helix
Sheet
Coil

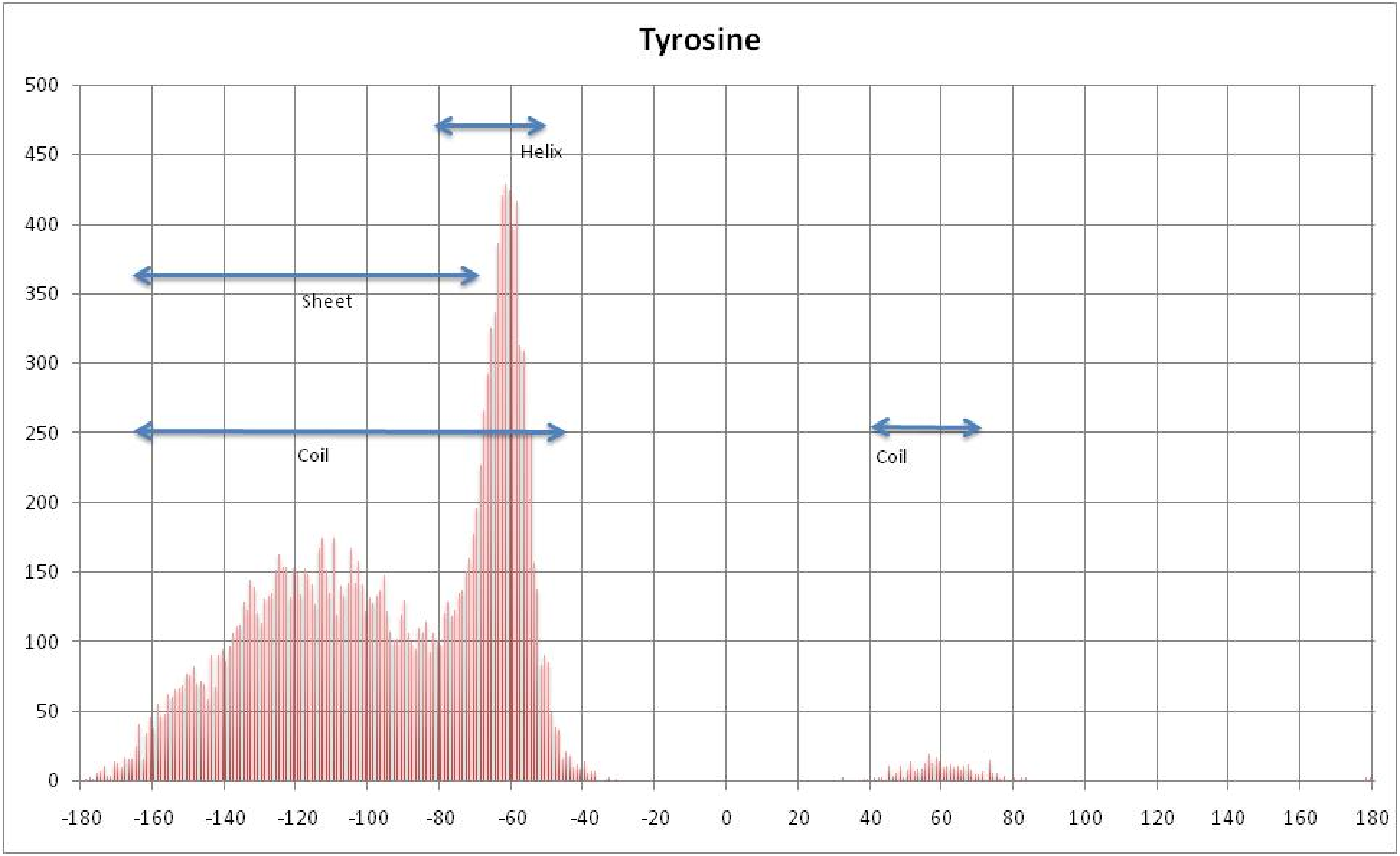

Tyrosine
Helix
Sheet
Coil
Coil
500
450
400
350
300
250
200
150
100
50
0
-180
-160
-140
-120
-100
-80
-60
-40
-20
0
20
40
60
80
100
120
140
160
180

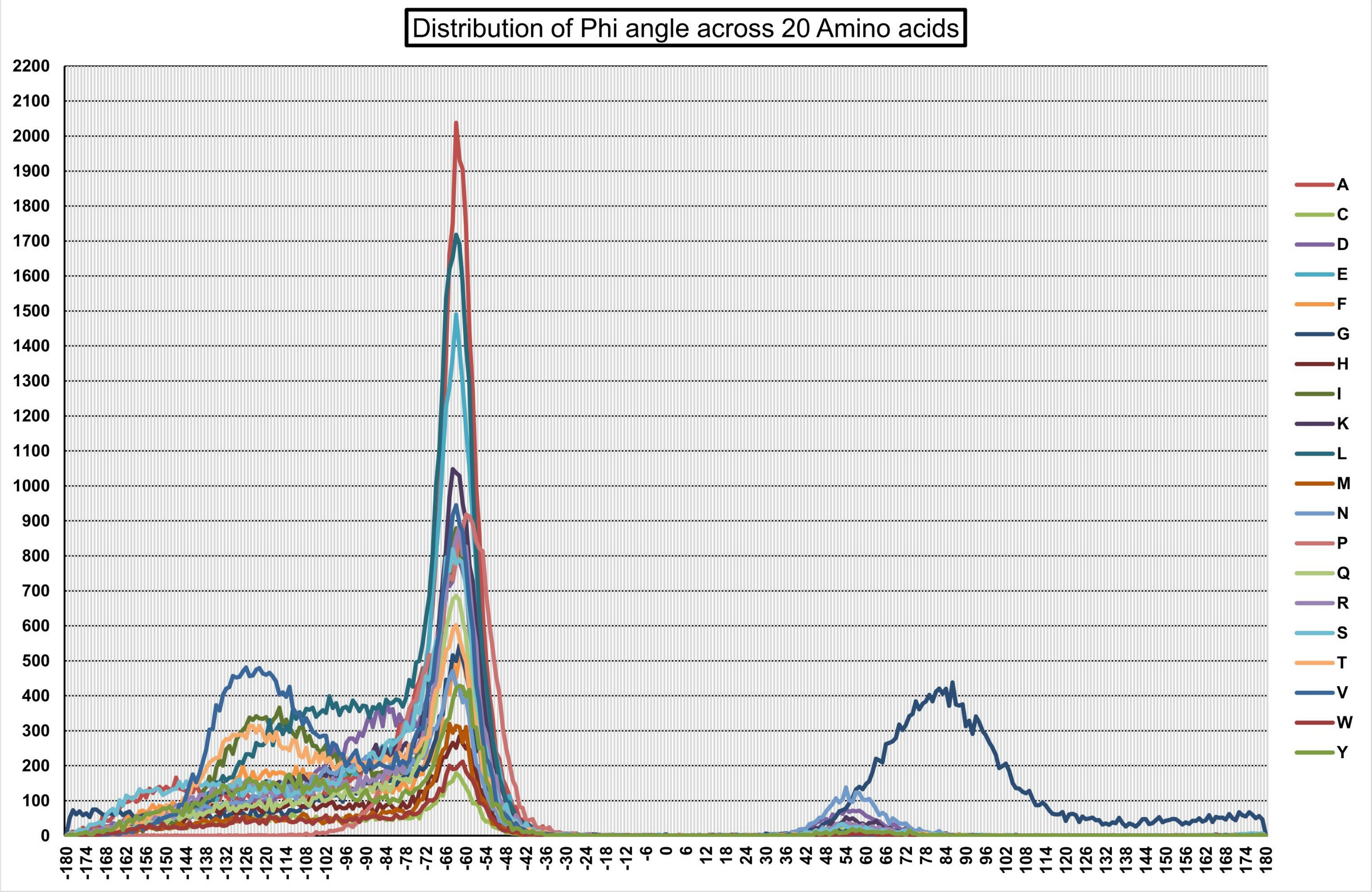
Distribution of Phi angle across 20 Amino acids
A
C
D
E
F
G
H
I
K
L
M
N
P
Q
R
S
T
V
W
Y

Psi Angle Distribution

X-axis shows angle distribution

Y-axis shows the number of residues

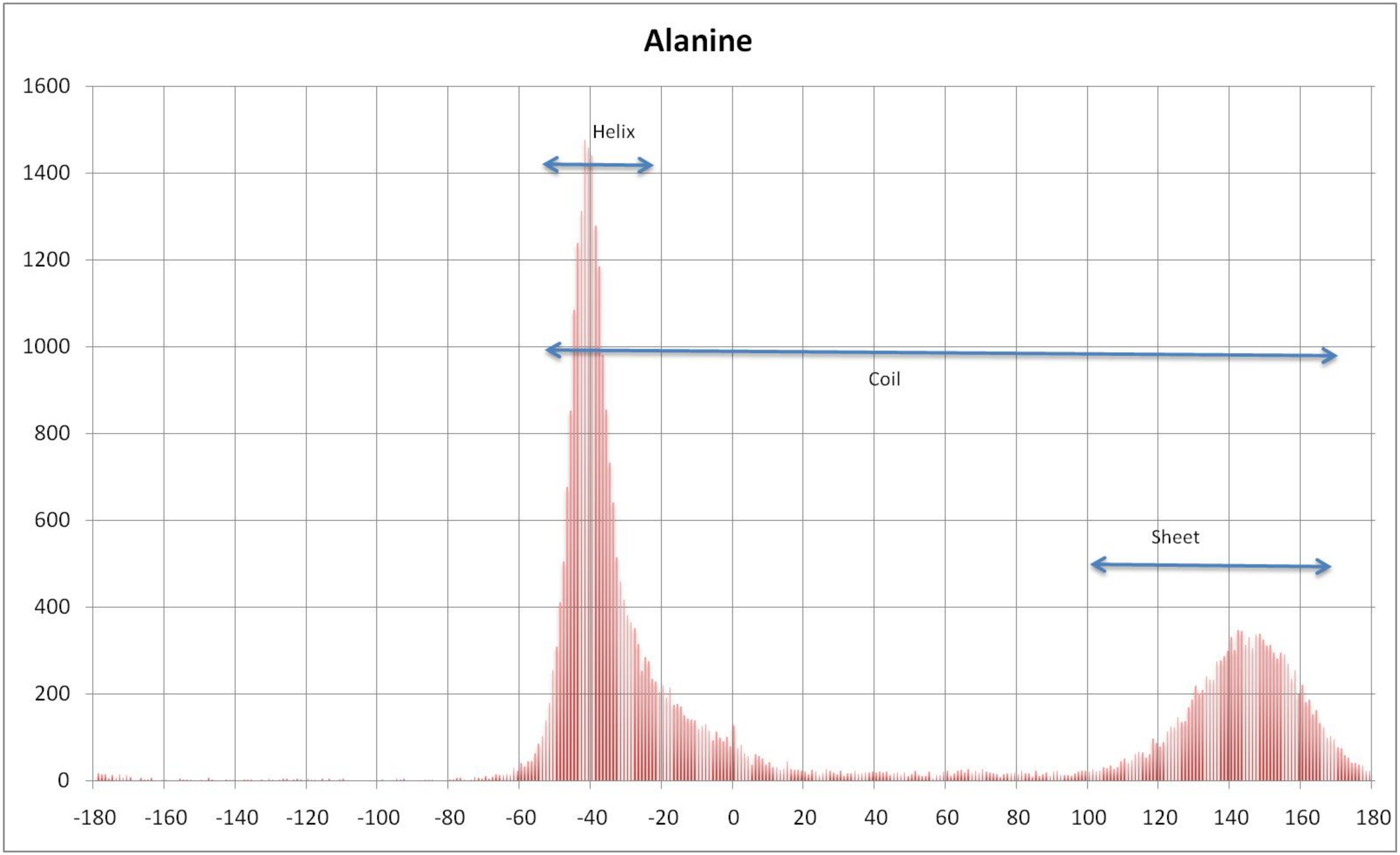

Alanine
Helix
Coil
Sheet
1600
1400
1200
1000
800
600
400
200
0
-180
-160
-140
-120
-100
-80
-60
-40
-20
0
20
40
60
80
100
120
140
160
180

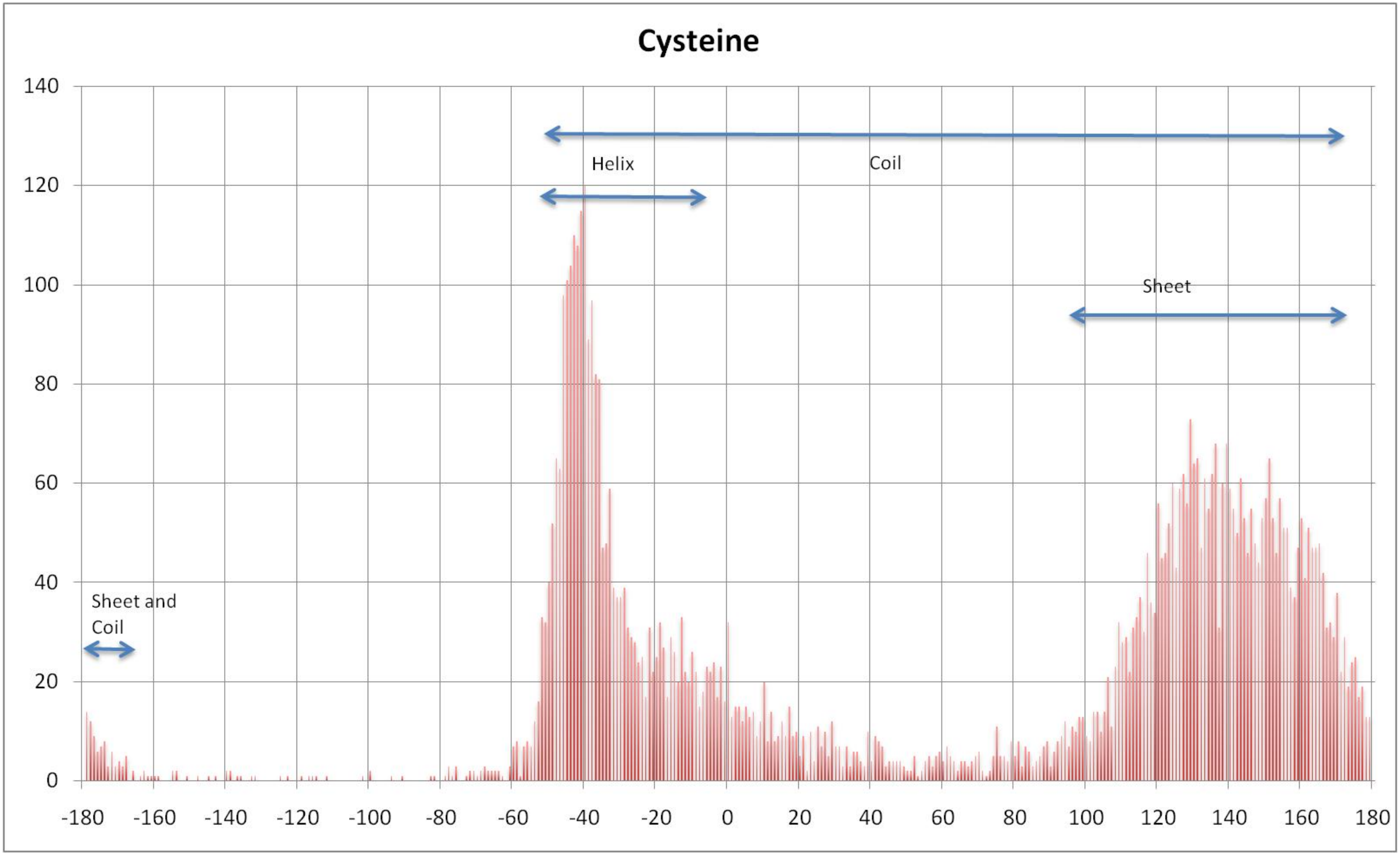
Cysteine
140
120
100
80
60
40
20
0
-180
-160
-140
-120
-100
-80
-60
-40
-20
0
20
40
60
80
100
120
140
160
180
Helix
Coil
Sheet
Sheet and
Coil

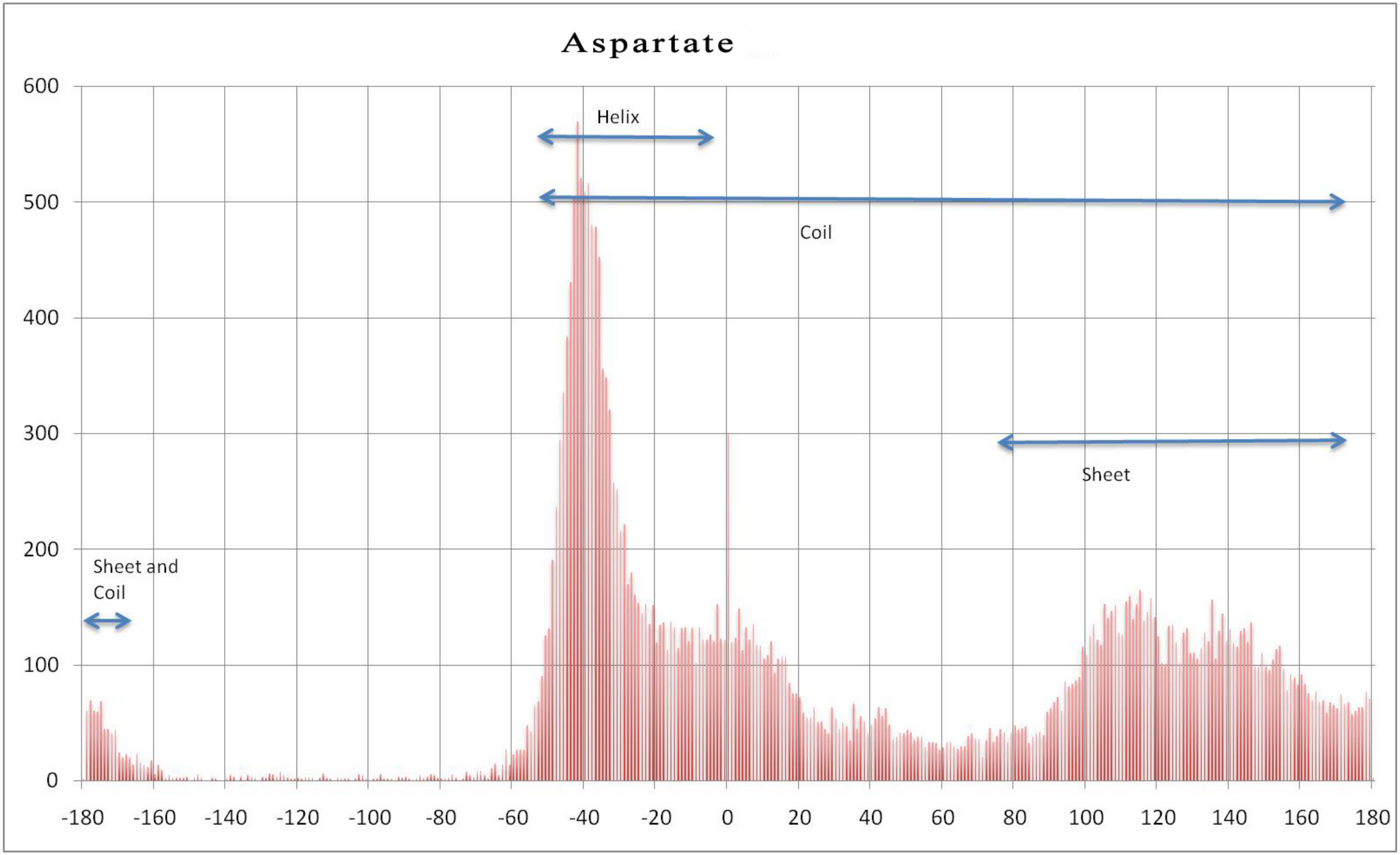
Aspartate
Helix
Coil
Sheet
Sheet and
Coil
600
500
400
300
200
100
0
-180
-160
-140
-120
-100
-80
-60
-40
-20
0
20
40
60
80
100
120
140
160
180

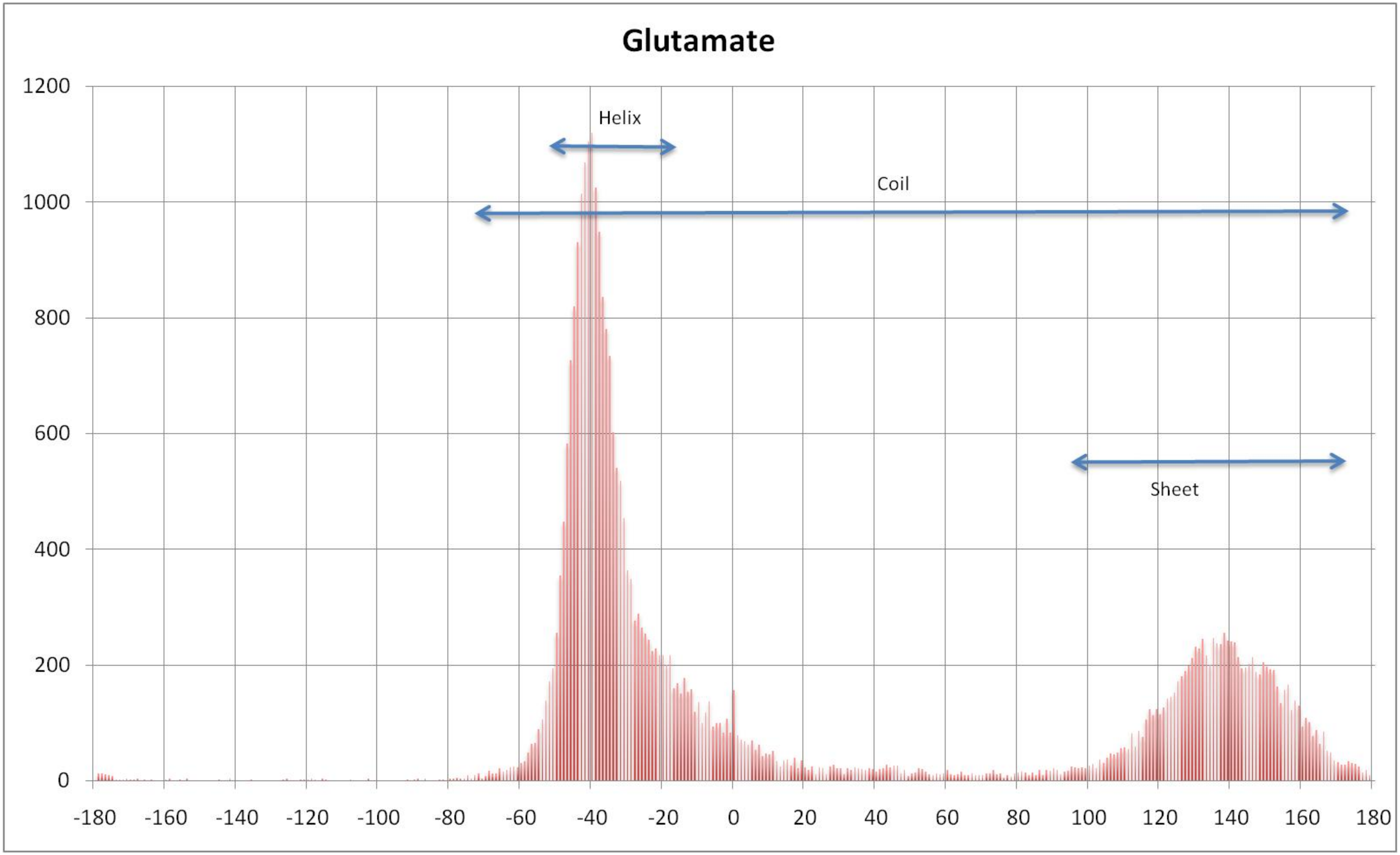
Glutamate
Helix
Coil
Sheet
1200
1000
800
600
400
200
0
-180
-160
-140
-120
-100
-80
-60
-40
-20
0
20
40
60
80
100
120
140
160
180

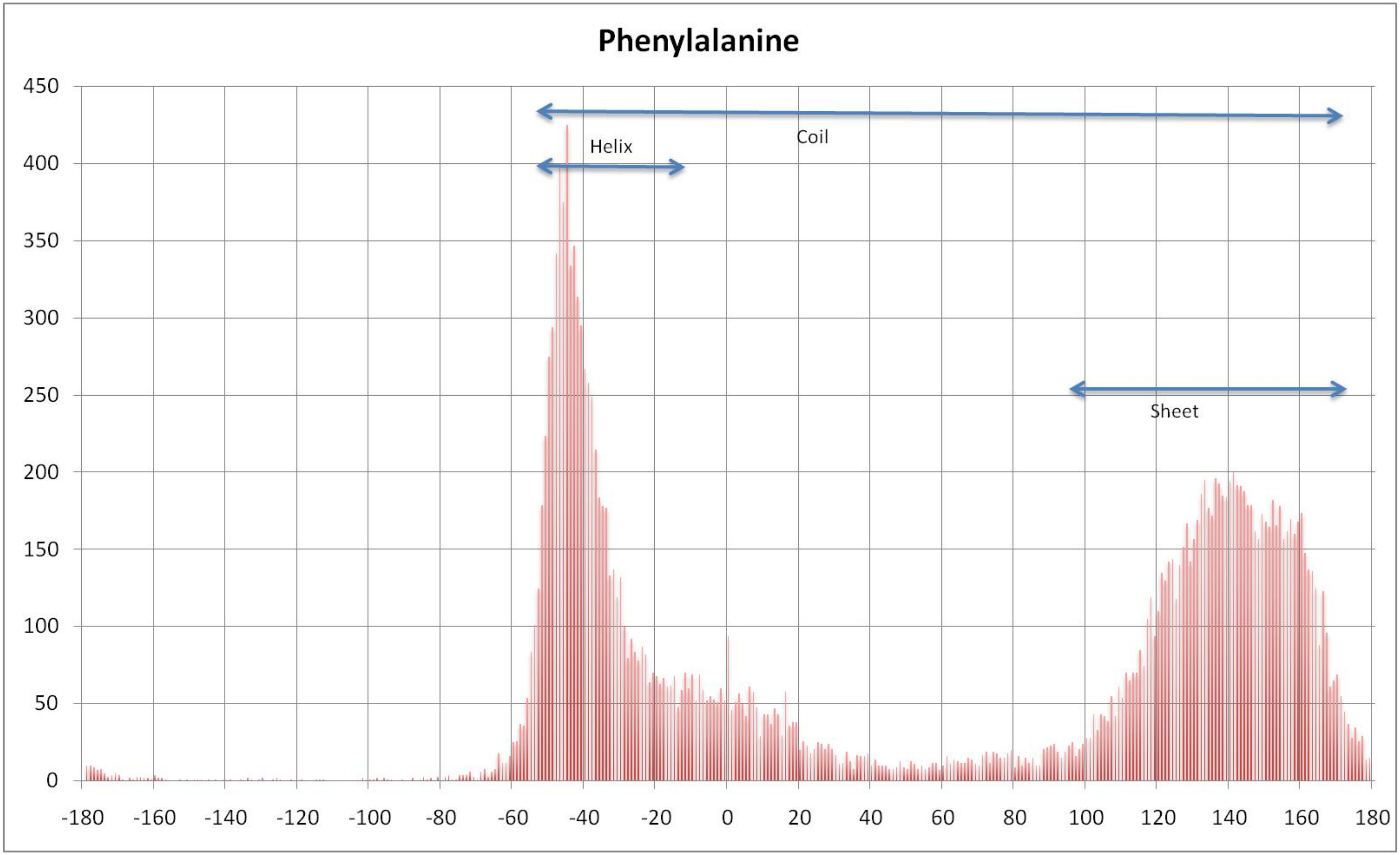

Phenylalanine
Helix
Coil
Sheet
450
400
350
300
250
200
150
100
50
0
-180
-160
-140
-120
-100
-80
-60
-40
-20
0
20
40
60
80
100
120
140
160
180

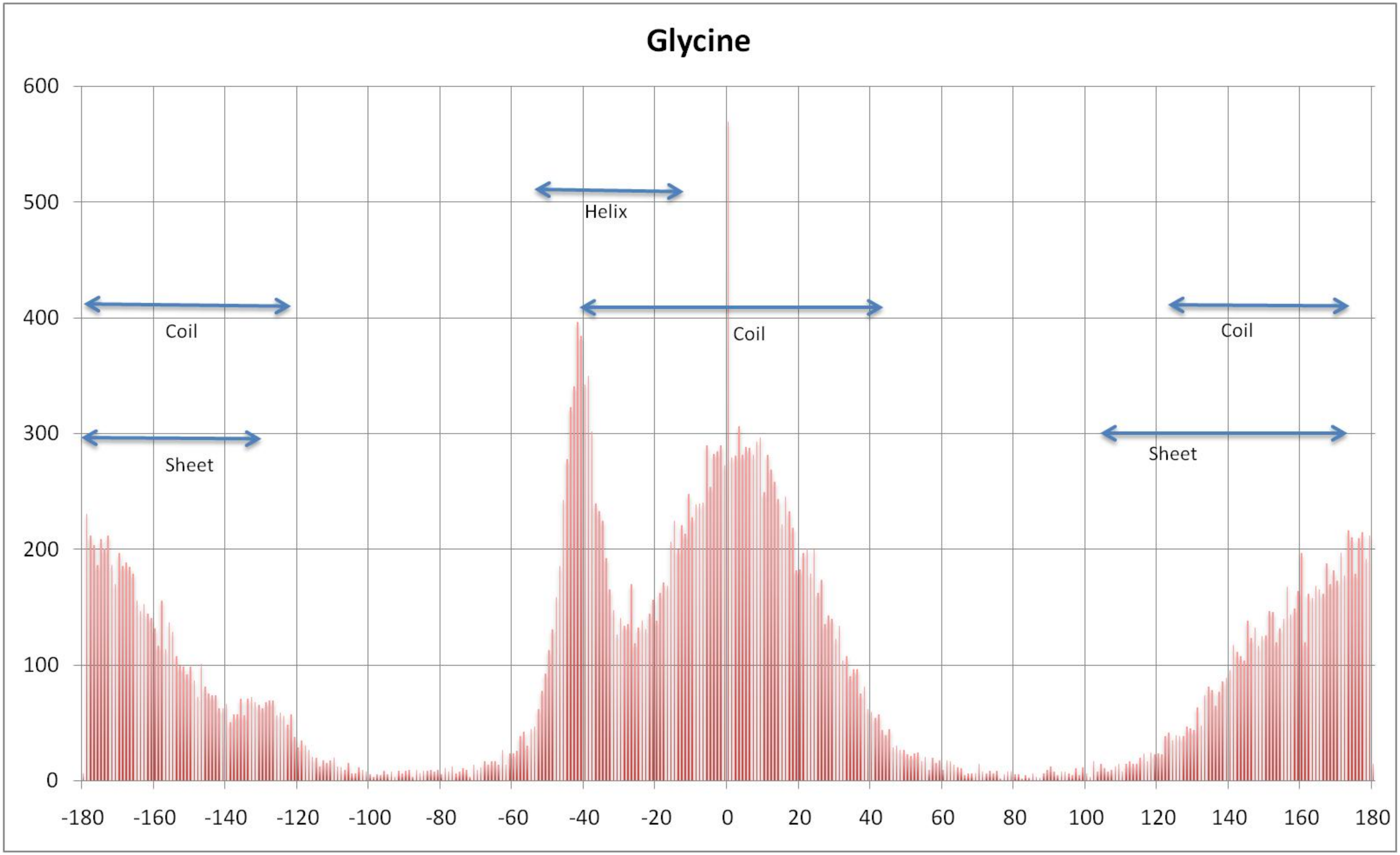
Glycine
600
500
400
300
200
100
0
-180
-160
-140
-120
-100
-80
-60
-40
-20
0
20
40
60
80
100
120
140
160
180
Helix
Coil
Coil
Coil
Sheet
Sheet

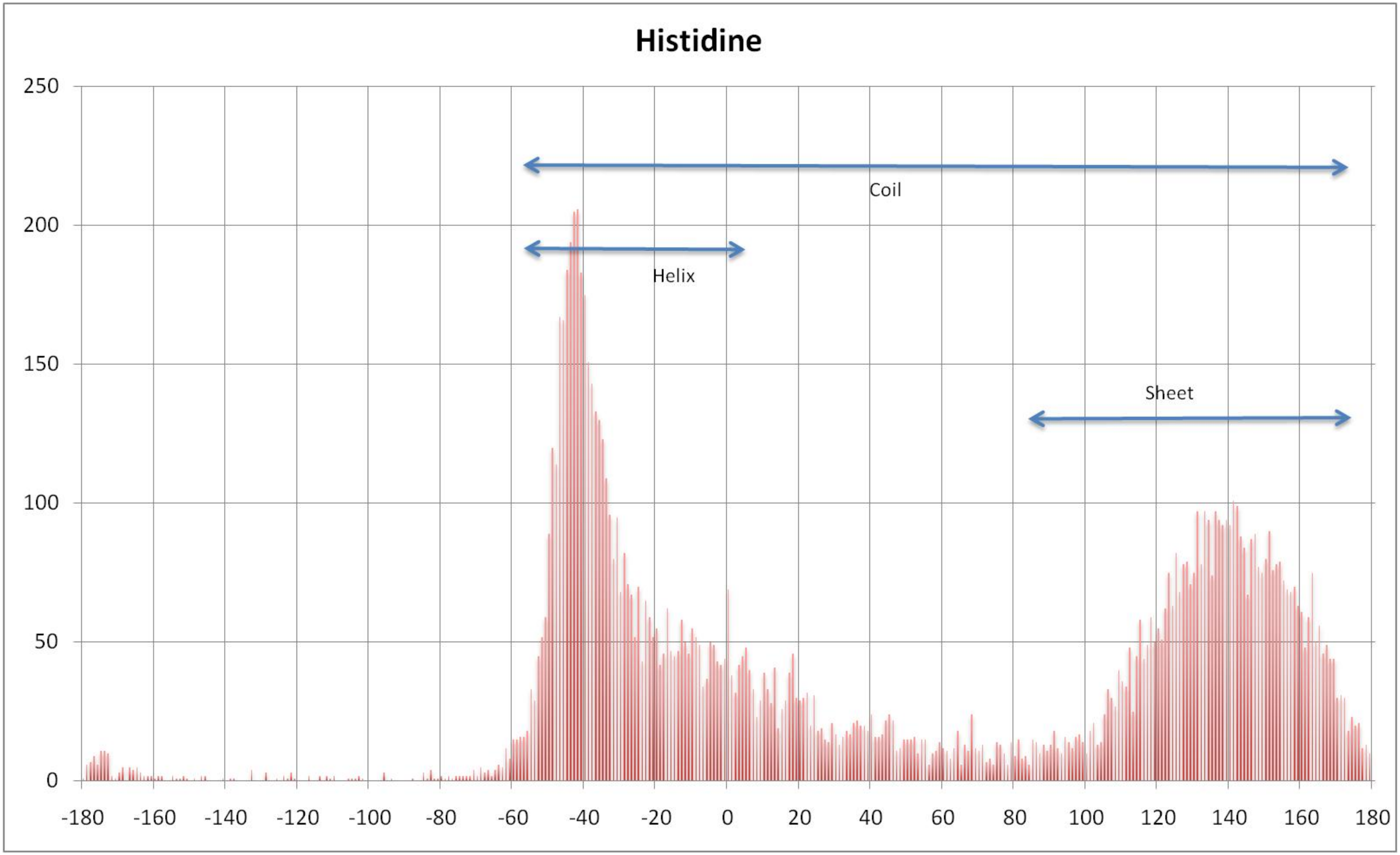
Histidine
Coil
Helix
Sheet
250
200
150
100
50
0
-180
-160
-140
-120
-100
-80
-60
-40
-20
0
20
40
60
80
100
120
140
160
180

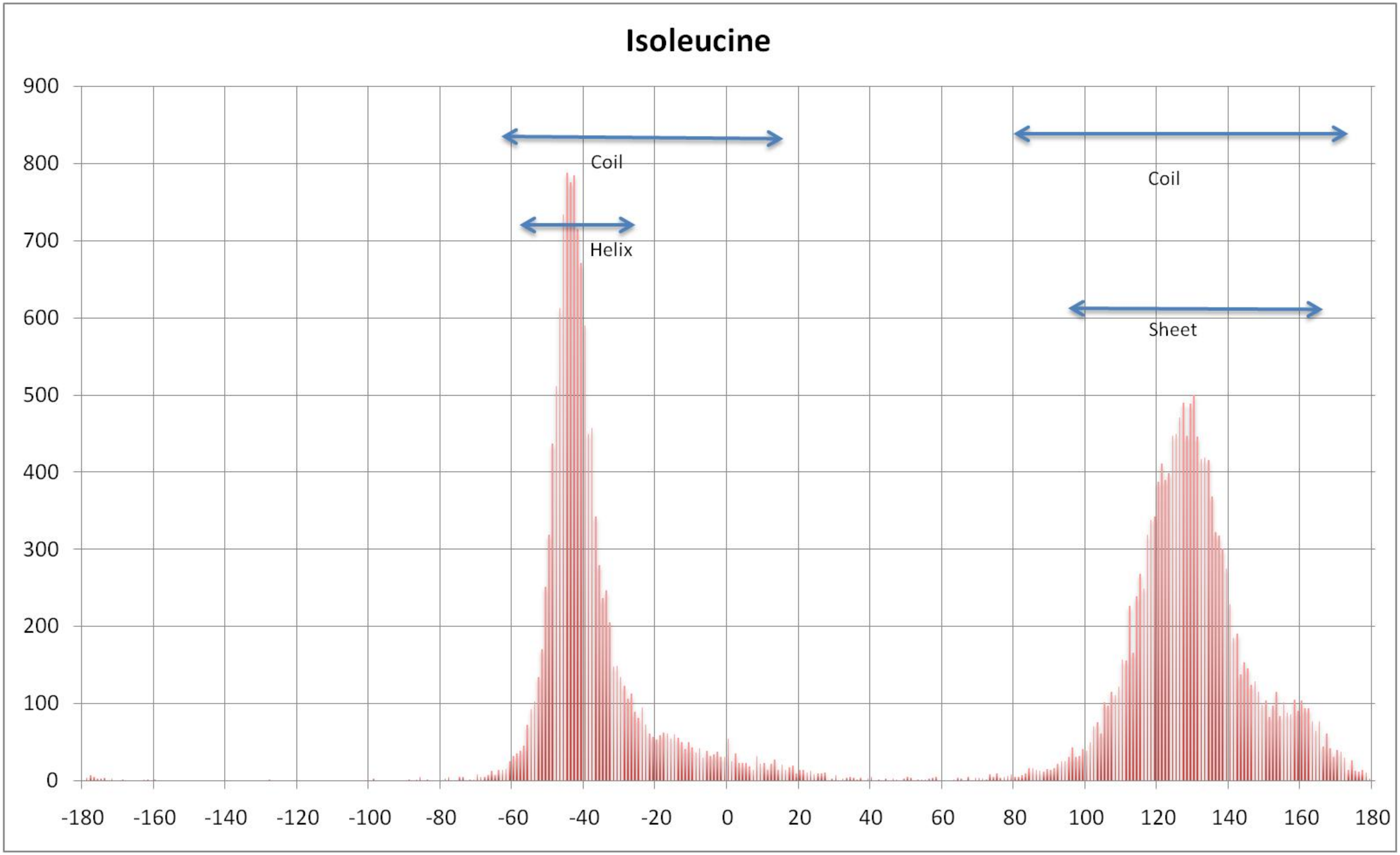

Isoleucine
Coil
Helix
Coil
Sheet
900
800
700
600
500
400
300
200
100
0
-180
-160
-140
-120
-100
-80
-60
-40
-20
0
20
40
60
80
100
120
140
160
180

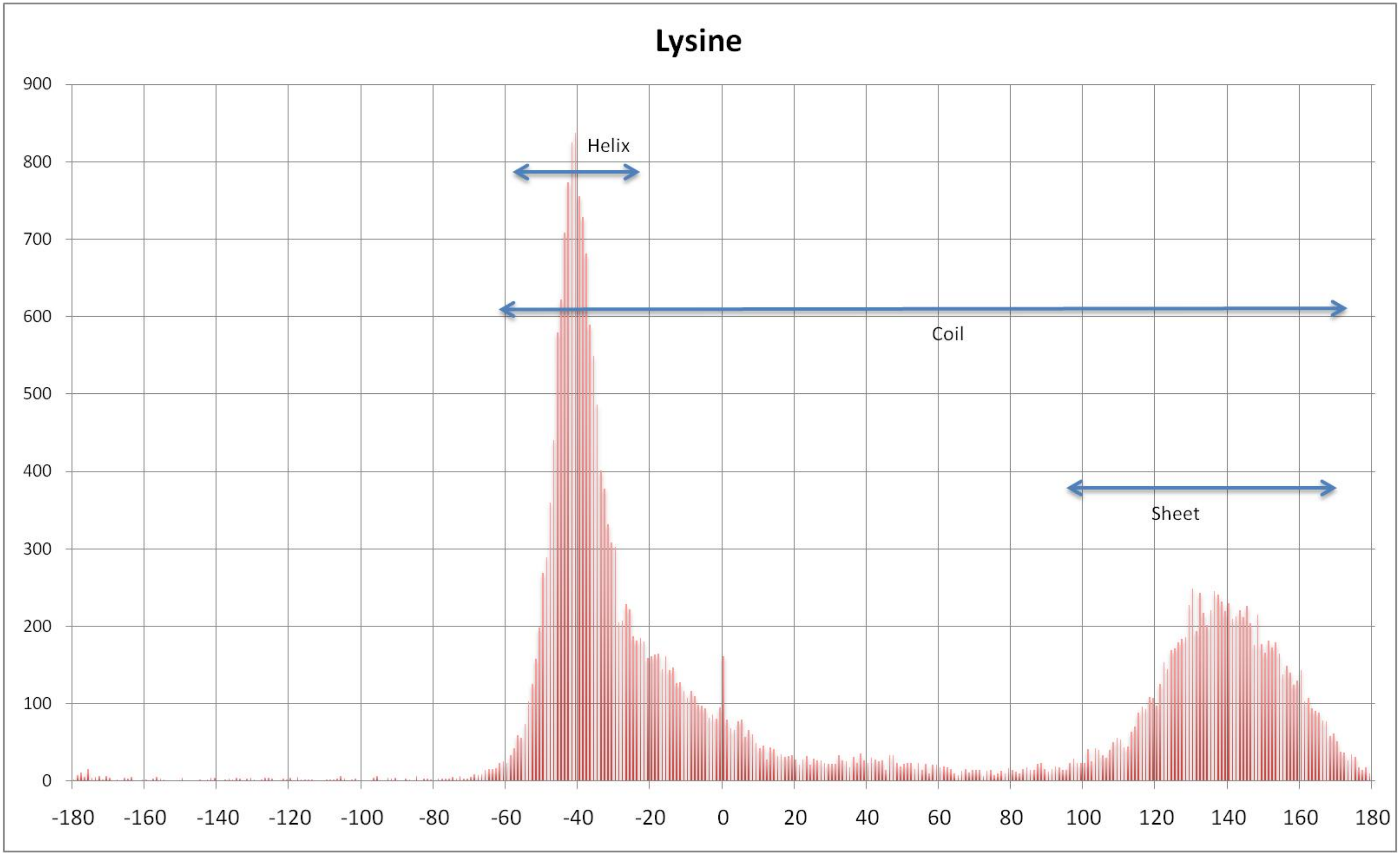
Lysine
Helix
Coil
Sheet
900
800
700
600
500
400
300
200
100
0
-180 -160 -140 -120 -100 -80 -60 -40 -20 0 20 40 60 80 100 120 140 160 180

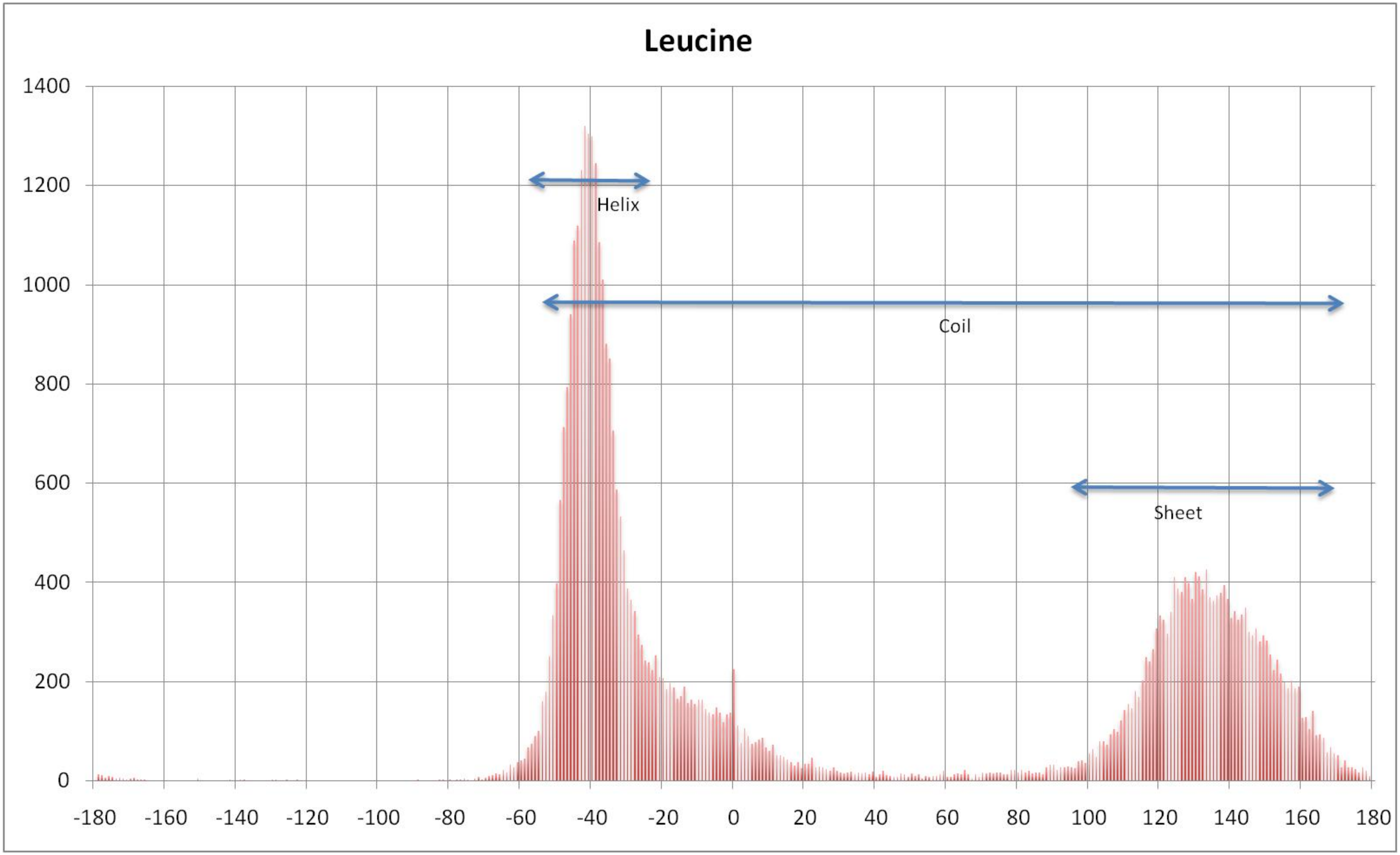

Leucine
Helix
Coil
Sheet
1400
1200
1000
800
600
400
200
0
-180 -160 -140 -120 -100 -80 -60 -40 -20 0 20 40 60 80 100 120 140 160 180

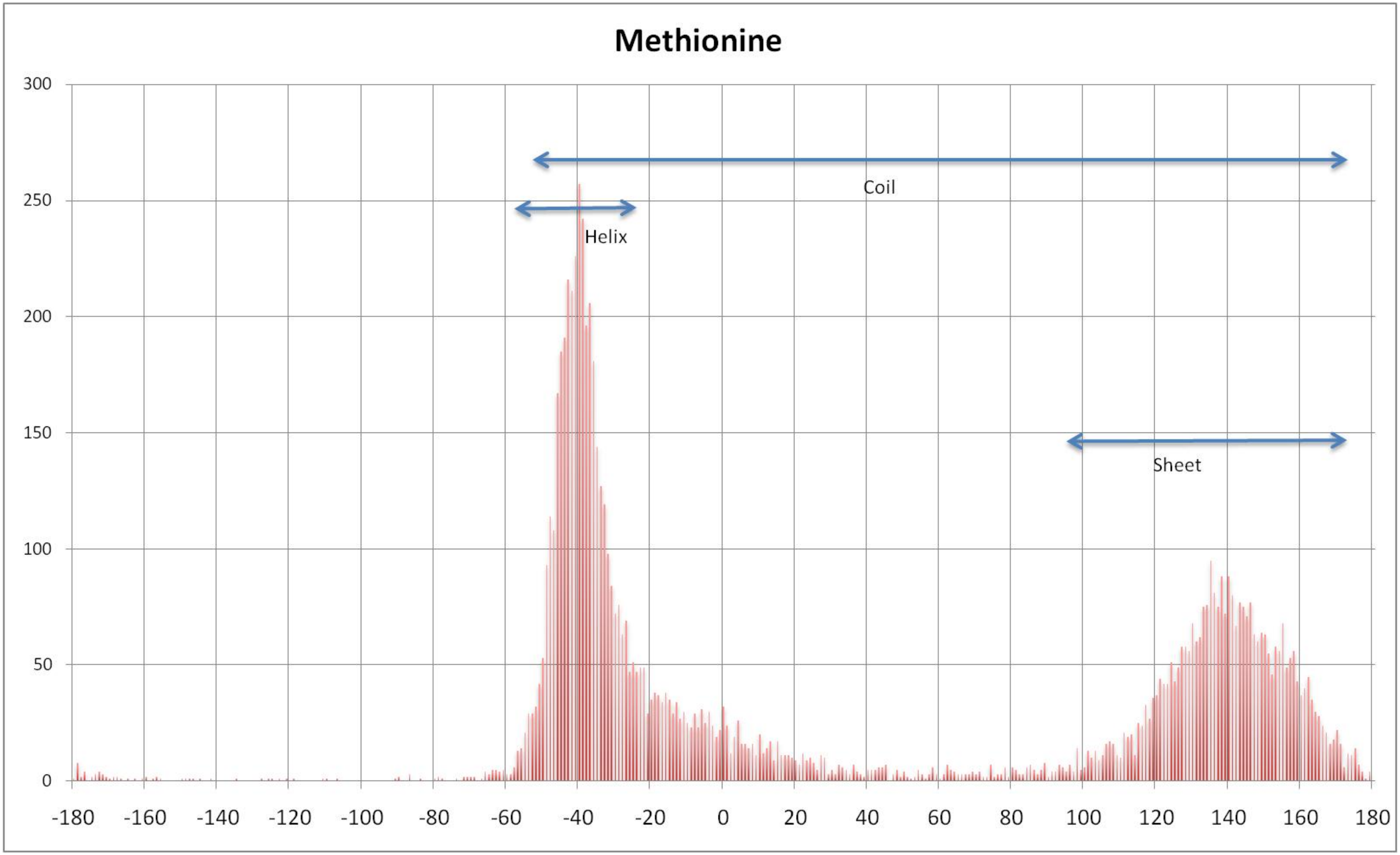

Methionine
Coil
Helix
Sheet
300
250
200
150
100
50
0
-180
-160
-140
-120
-100
-80
-60
-40
-20
0
20
40
60
80
100
120
140
160
180

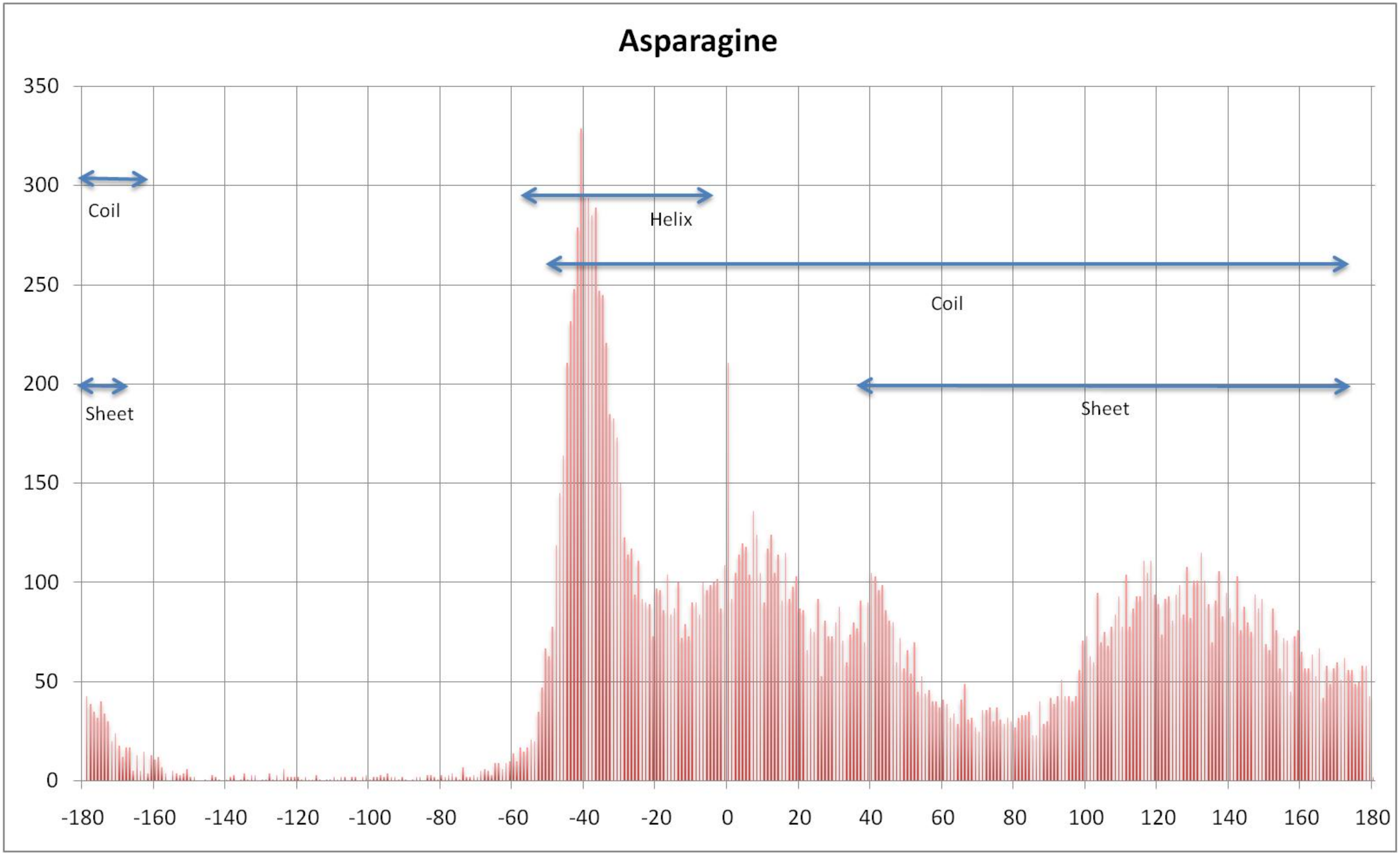

Asparagine
350
300
250
200
150
100
50
0
-180
-160
-140
-120
-100
-80
-60
-40
-20
0
20
40
60
80
100
120
140
160
180
Coil
Helix
Coil
Sheet
Sheet

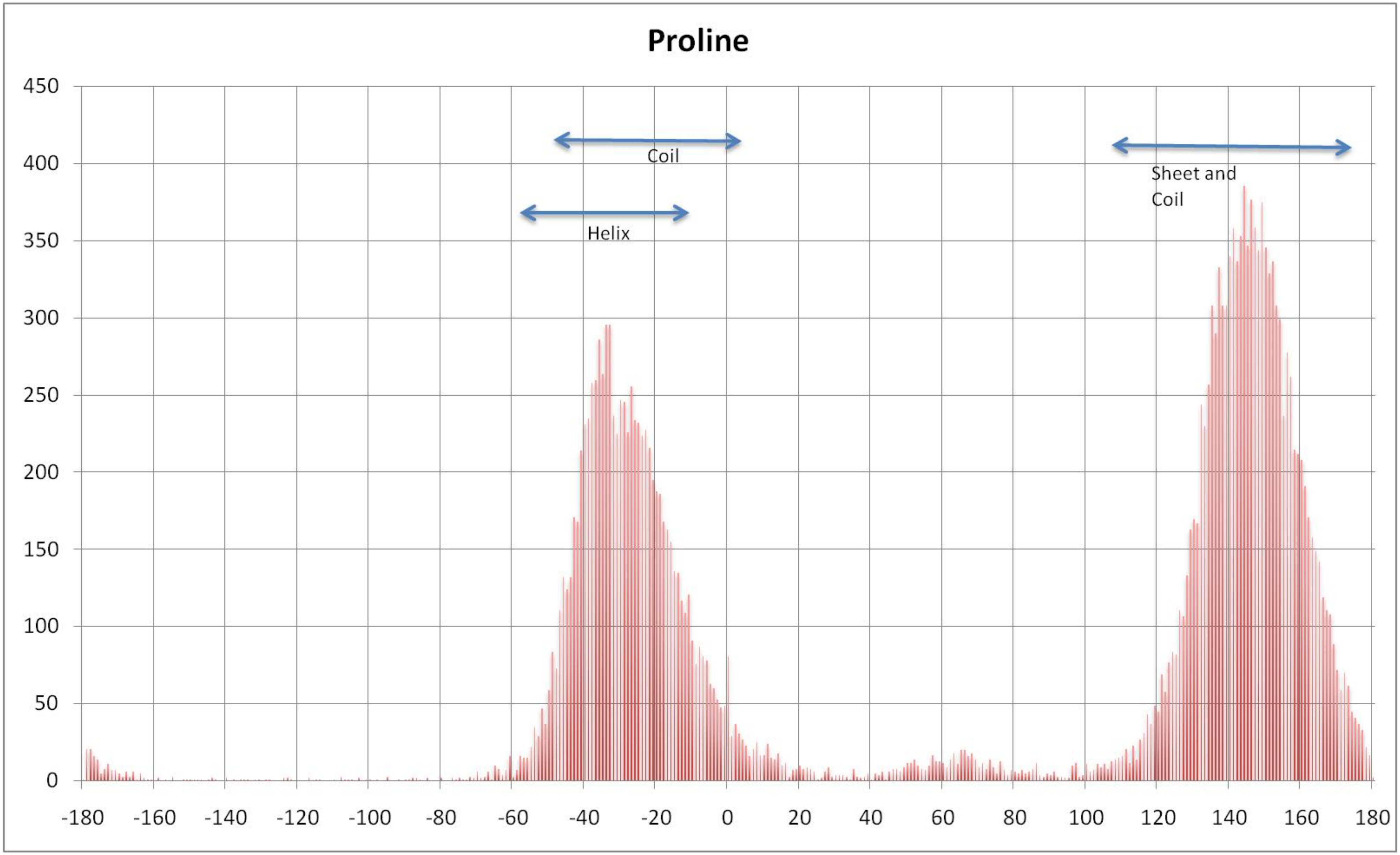

Proline
Coil
Helix
Sheet and
Coil
450
400
350
300
250
200
150
100
50
0
-180
-160
-140
-120
-100
-80
-60
-40
-20
0
20
40
60
80
100
120
140
160
180

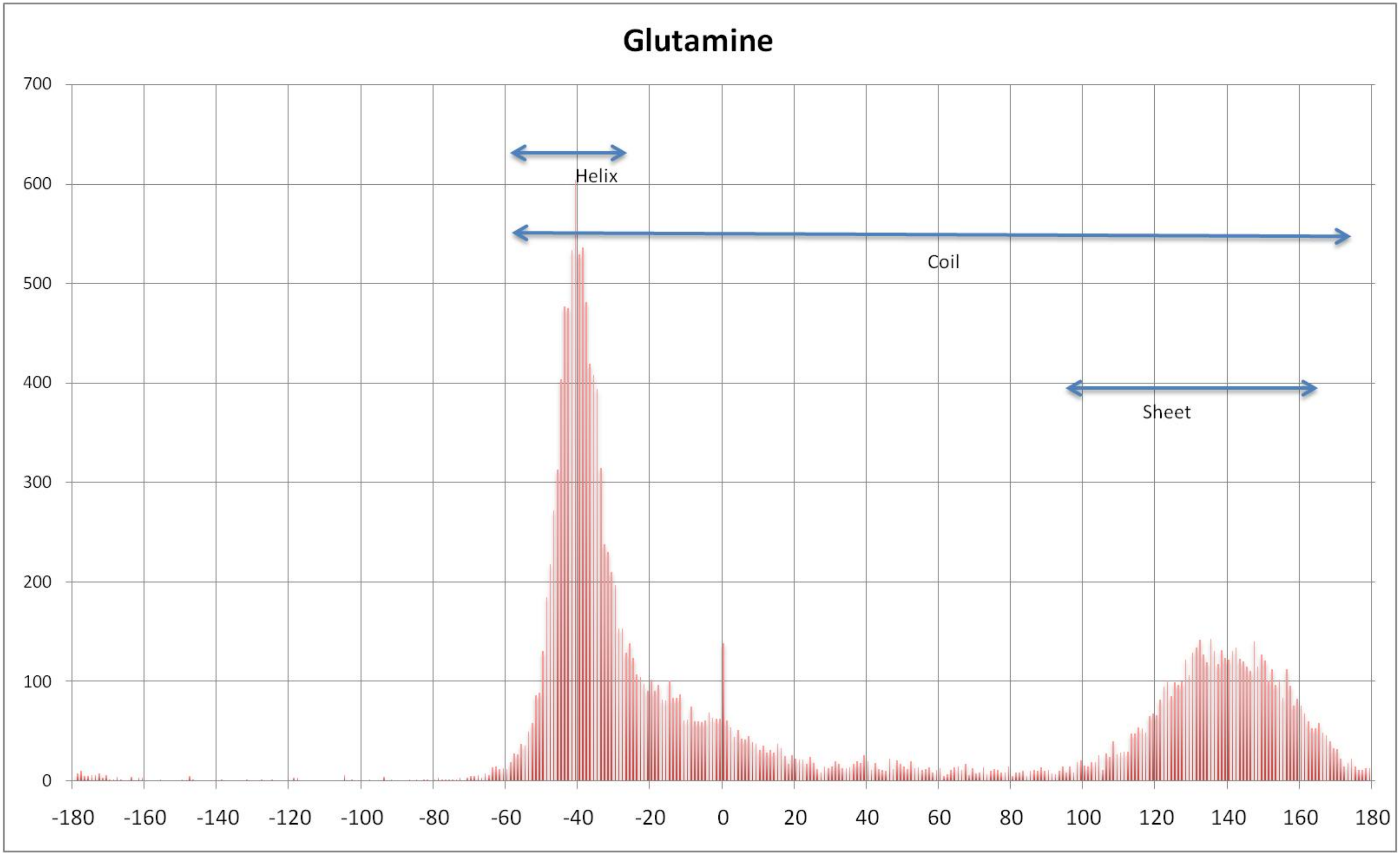
Glutamine
Helix
Coil
Sheet
700
600
500
400
300
200
100
0
-180
-160
-140
-120
-100
-80
-60
-40
-20
0
20
40
60
80
100
120
140
160
180

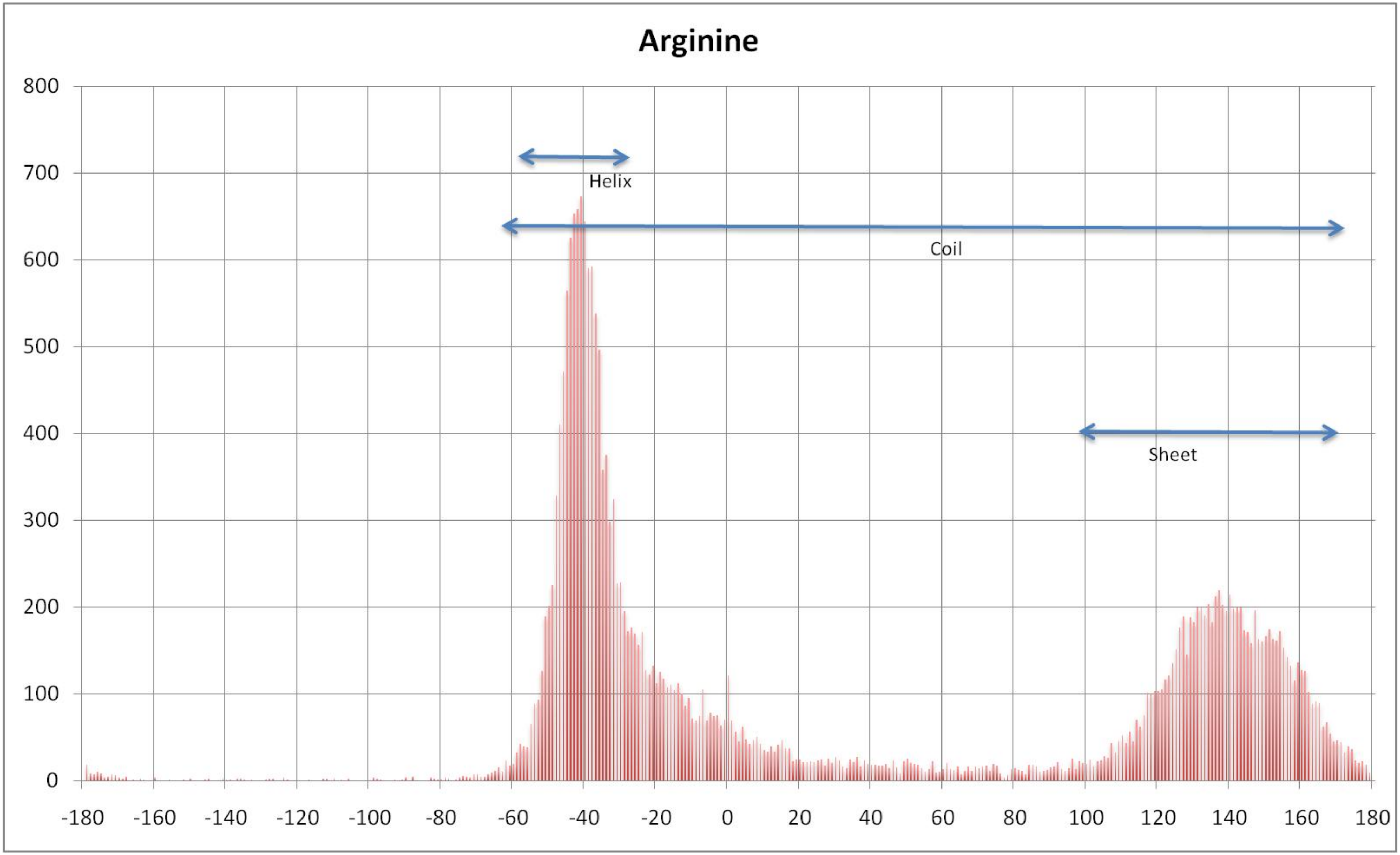
Arginine
Helix
Coil
Sheet
800
700
600
500
400
300
200
100
0
-180
-160
-140
-120
-100
-80
-60
-40
-20
0
20
40
60
80
100
120
140
160
180

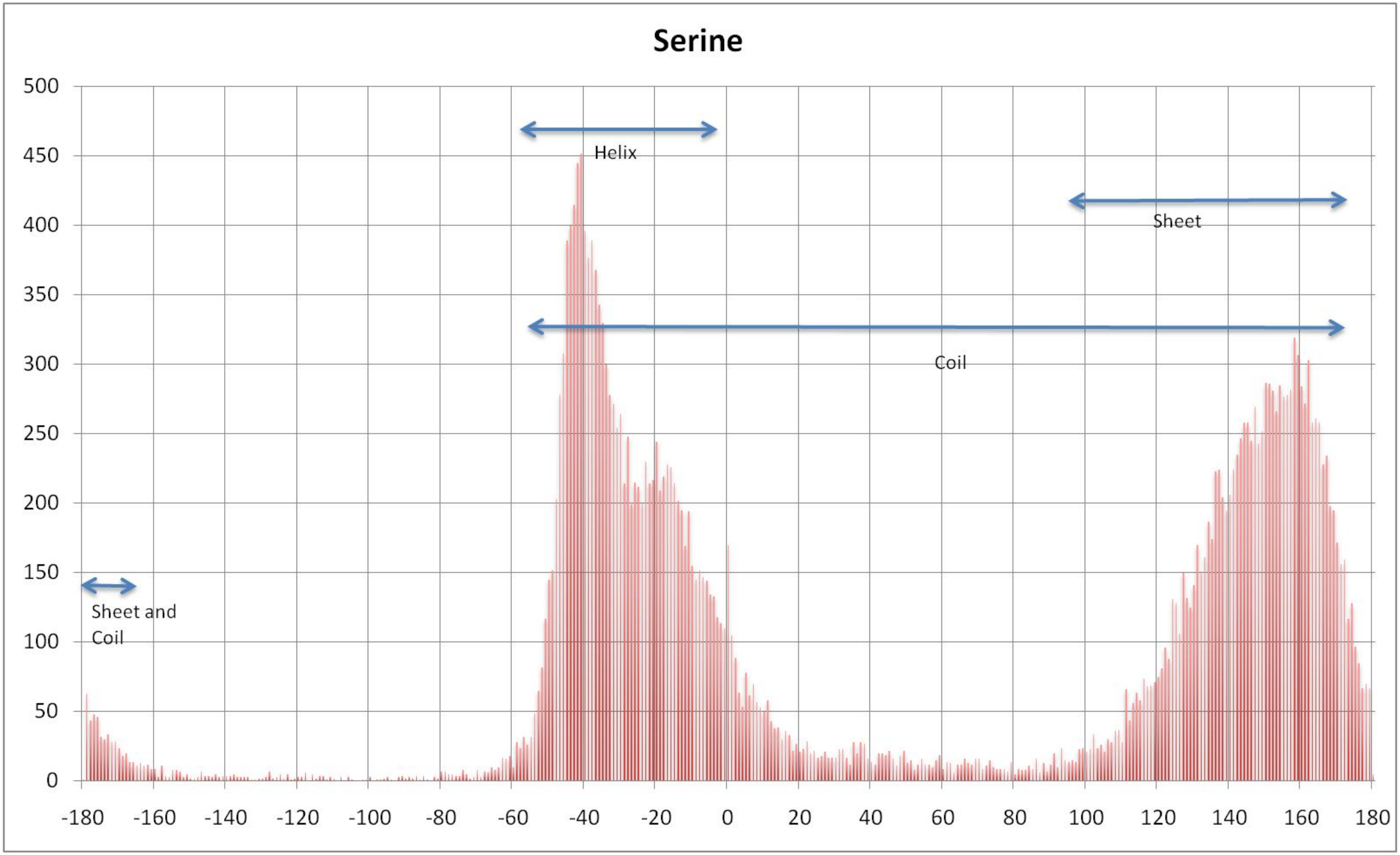
Serine
Helix
Sheet
Coil
Sheet and
Coil
500
450
400
350
300
250
200
150
100
50
0
-180
-160
-140
-120
-100
-80
-60
-40
-20
0
20
40
60
80
100
120
140
160
180

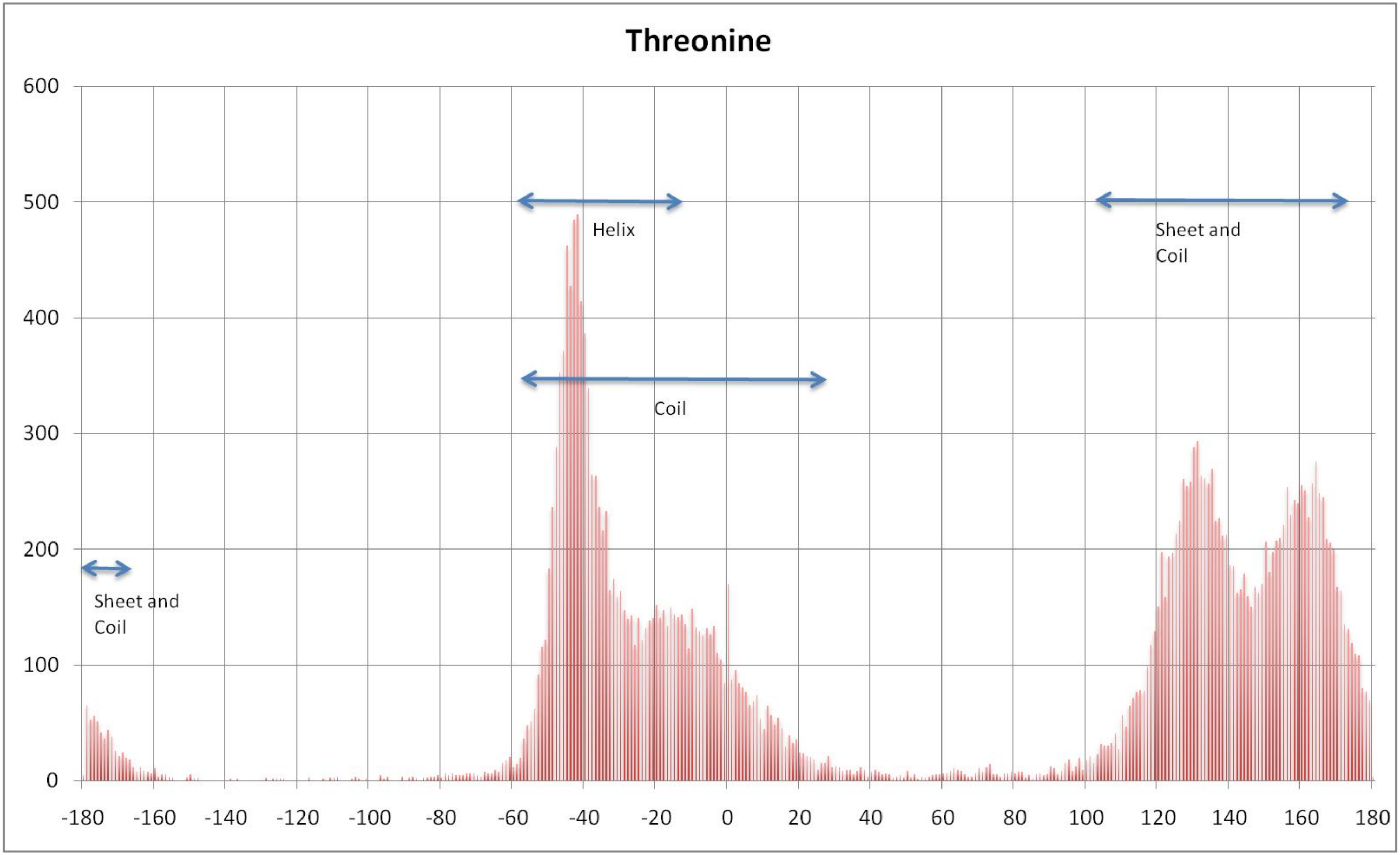

Threonine
600
500
400
300
200
100
0
Helix
Coil
Sheet and
Coil
Sheet and
Coil
-180
-160
-140
-120
-100
-80
-60
-40
-20
0
20
40
60
80
100
120
140
160
180

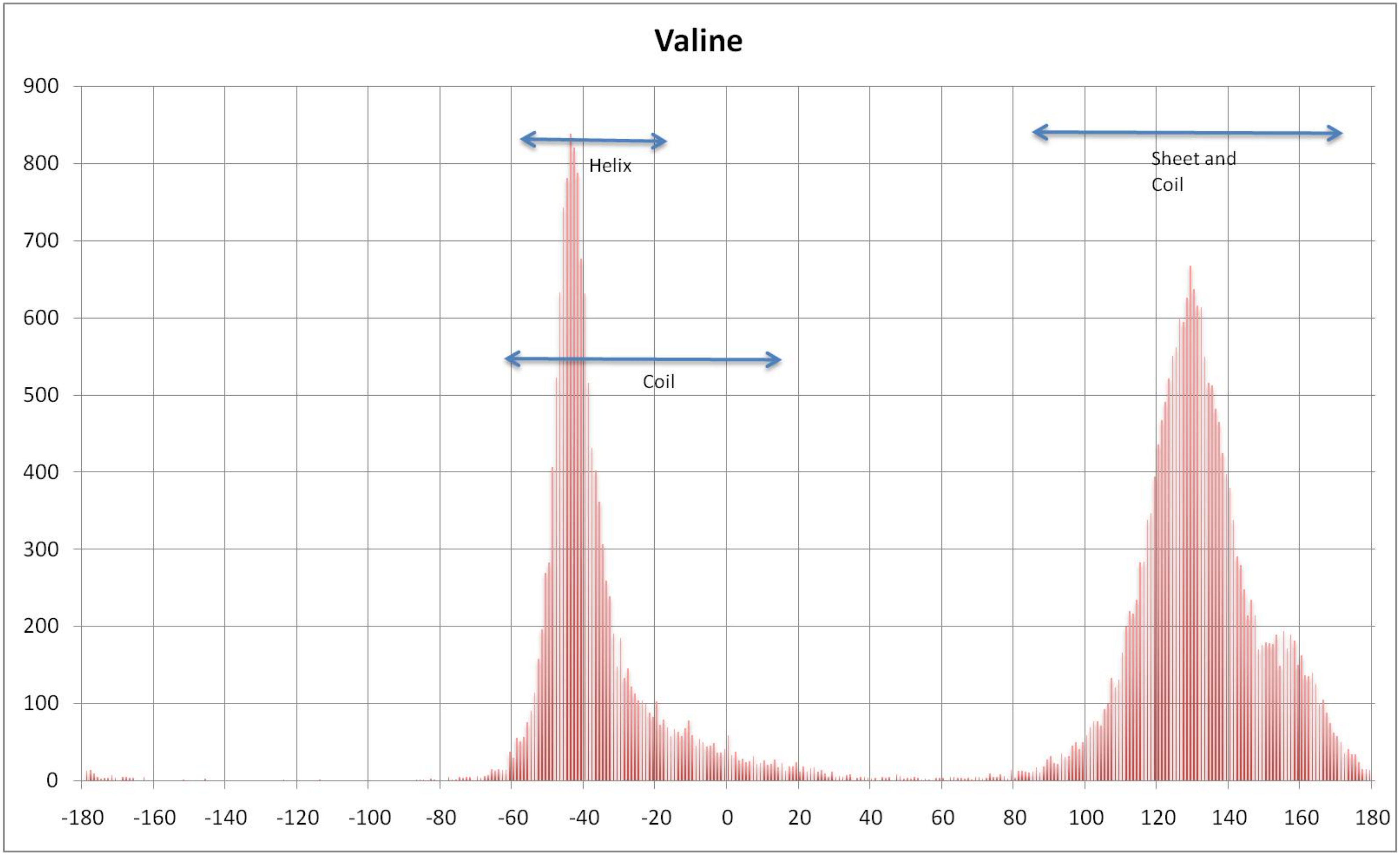

Valine
900
800
700
600
500
400
300
200
100
0
-180
-160
-140
-120
-100
-80
-60
-40
-20
0
20
40
60
80
100
120
140
160
180
Helix
Coil
Sheet and
Coil

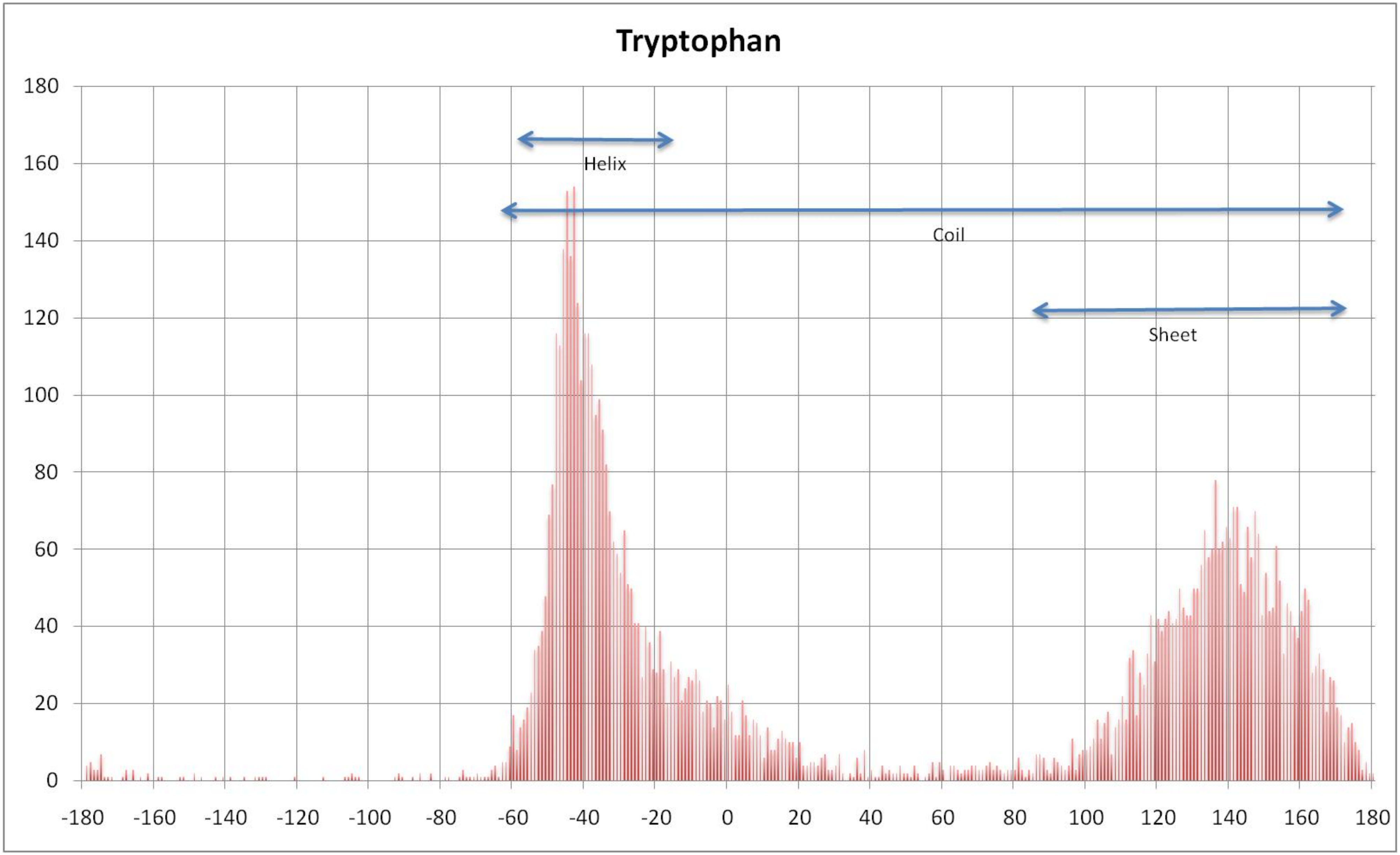

Tryptophan
Helix
Coil
Sheet
180
160
140
120
100
80
60
40
20
0
-180 -160 -140 -120 -100 -80 -60 -40 -20 0 20 40 60 80 100 120 140 160 180

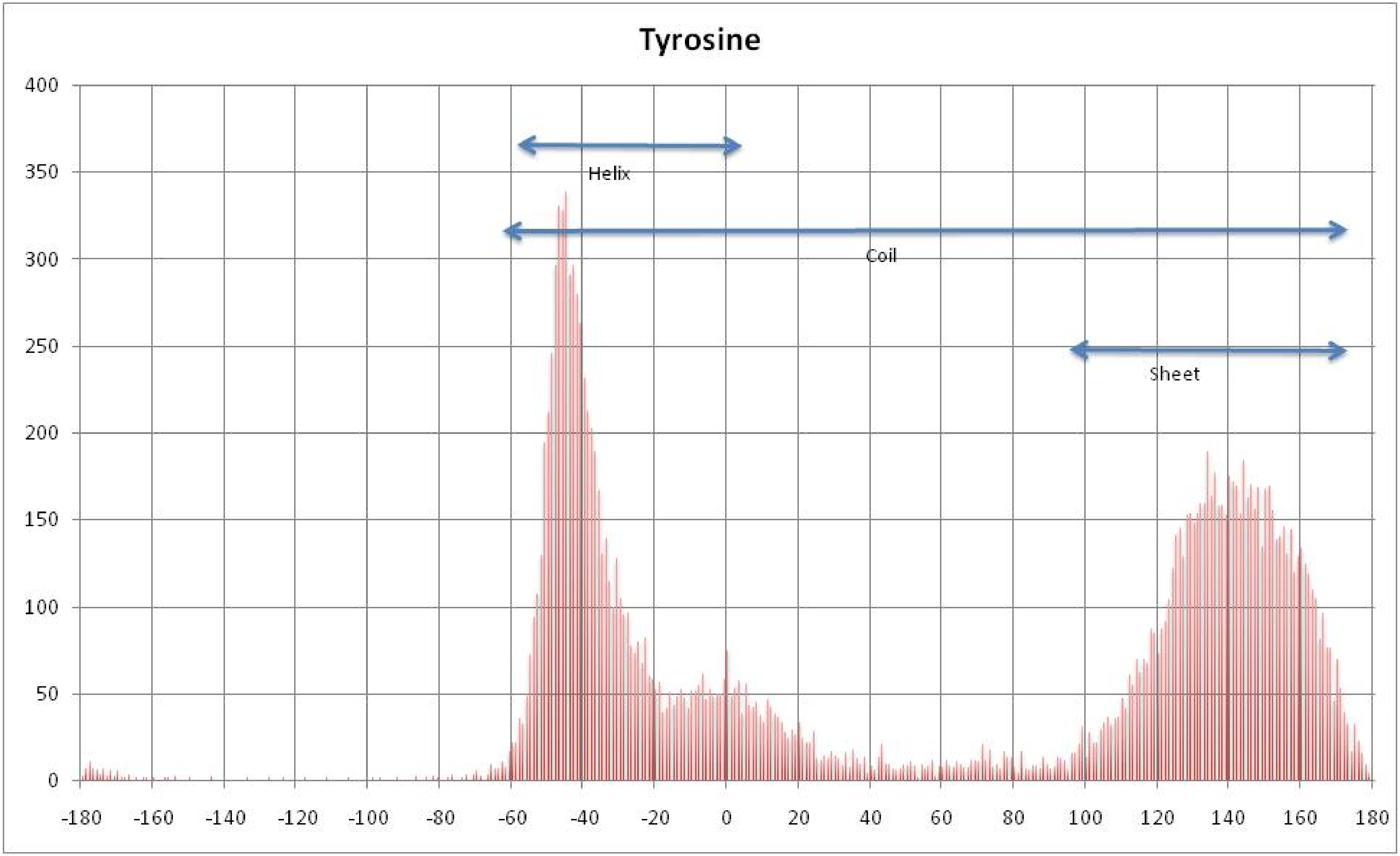
Tyrosine
Helix
Coil
Sheet
400
350
300
250
200
150
100
50
0
-180
-160
-140
-120
-100
-80
-60
-40
-20
0
20
40
60
80
100
120
140
160
180

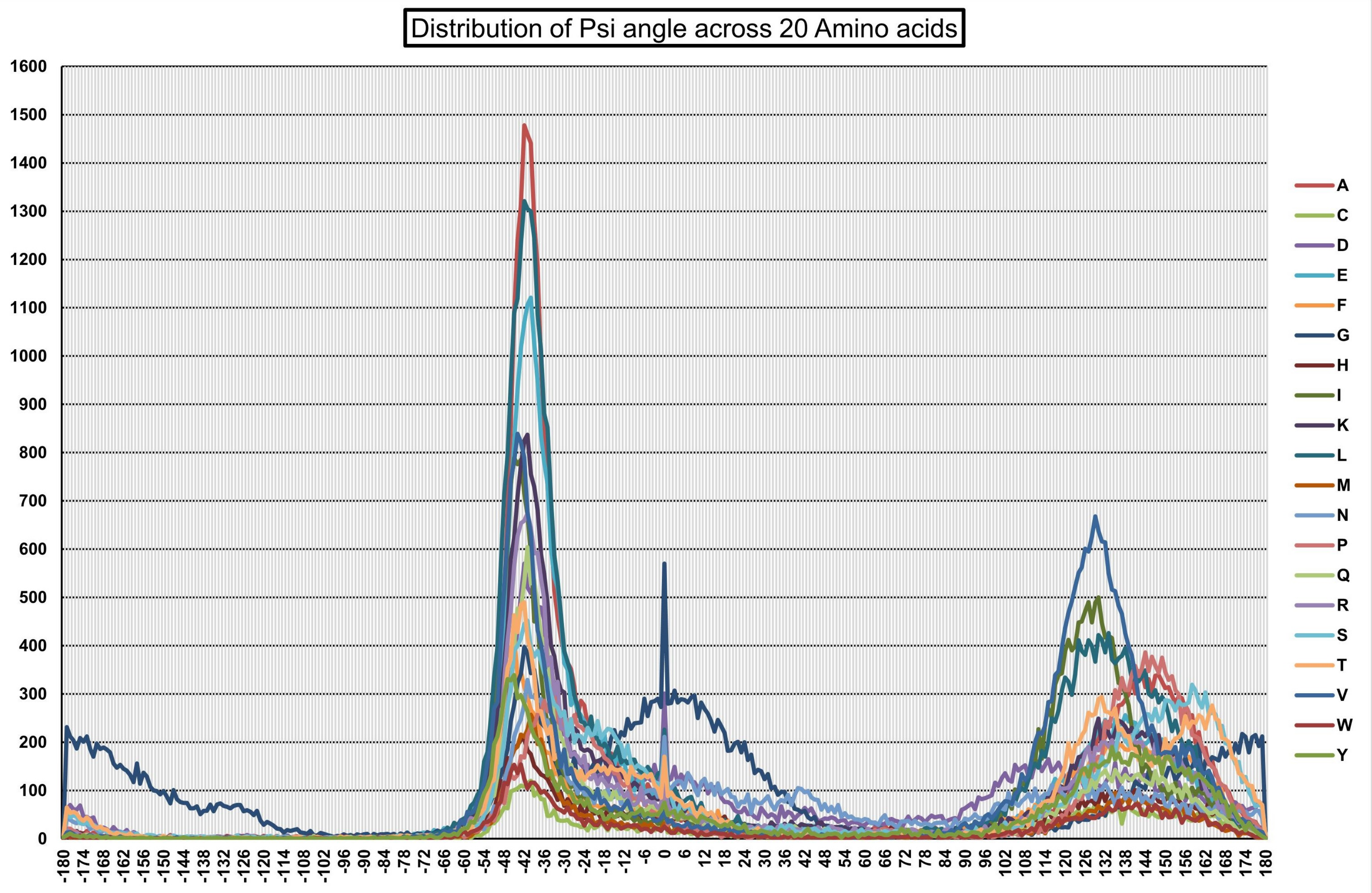

Distribution of Psi angle across 20 Amino acids
A
C
D
E
F
G
H
I
K
L
M
N
P
Q
R
S
T
V
W
Y
