# Supplementary material for: Evaluation of Protein Dihedral Angle Prediction Methods
Source: PLoS One. 2014 Aug 28;9(8):e105667. doi: 10.1371/journal.pone.0105667 (PMC4148315; doi:10.1371/journal.pone.0105667)
Supplement: File S2 — Contains the following files: Table S1. PDB IDs of 4682 PDB chains used in ccPDB30 dataset. Table S2. Pearson correlation coefficient (PCC) of methods on different datasets. Methods are represented by rows and datasets are represented by columns respectively. Table S3. Root-mean-square-error (RMSE) of methods on different datasets. Methods are represented by rows and datasets are represented by columns respectively. Table S4. Distribution of phi angle for 20 amino acids. Table S5. Distribution of psi angle for 20 amino acids. (DOC) [file pone.0105667.s002.doc]

Table S1: PDB IDs of 4682 PDB chains used in ccPDB30 dataset.

| 1a34A | 1a3aA | 1a3cA | 1a7dA | 1a82A | 1a95A | 1ae9A | 1agqA | 1ah7A | 1ajkA | 1ak0A | 1al3A | 1alqA |
| --- | --- | --- | --- | --- | --- | --- | --- | --- | --- | --- | --- | --- |
| 1amuA | 1amxA | 1aofA | 1aopA | 1apyA | 1aq6A | 1aqbA | 1asuA | 1atgA | 1ayxA | 1b0nA | 1b1cA | 1b25A |
| 1b2pA | 1b57A | 1b59A | 1b5fA | 1b5fB | 1b65A | 1b6aA | 1b71A | 1b7vA | 1b8dB | 1b93A | 1bf2A | 1bgfA |
| 1bheA | 1bk0A | 1bkrA | 1bs9A | 1bx7A | 1by2A | 1bz4A | 1c0iA | 1c3pA | 1c3qA | 1c44A | 1c5eA | 1c7kA |
| 1c7sA | 1cb0A | 1cb7B | 1cc3A | 1cc7A | 1cexA | 1cf9A | 1chdA | 1ci8A | 1cjcA | 1cp7A | 1cpoA | 1cqxA |
| 1cqyA | 1cvrA | 1cxyA | 1cy9A | 1czfA | 1czpA | 1d02A | 1d0cA | 1d0dA | 1d0qA | 1d2iA | 1d2zB | 1d3vA |
| 1d3yA | 1d6jA | 1d7cA | 1d8wA | 1danL | 1danT | 1danU | 1dbgA | 1deoA | 1deuA | 1dg6A | 1di6A | 1dkiA |
| 1dl5A | 1dlyA | 1dmgA | 1dokA | 1dowA | 1dp4A | 1dp7P | 1dqgA | 1dqnA | 1dqsA | 1dugA | 1dulA | 1dusA |
| 1dvoA | 1dw0A | 1dw9A | 1dypA | 1e15A | 1e1dA | 1e1hA | 1e25A | 1e2xA | 1e3aA | 1e3aB | 1e4fT | 1e5kA |
| 1e6iA | 1e8cA | 1eanA | 1earA | 1eazA | 1eb6A | 1ecsA | 1efzA | 1eg9B | 1eh6A | 1ei5A | 1ej2A | 1ej8A |
| 1ejbA | 1el6A | 1elqA | 1elvA | 1elwA | 1eokA | 1epfA | 1eq6A | 1eu8A | 1evkA | 1ew2A | 1ex2A | 1eyvA |
| 1ezgA | 1ezwA | 1f0kA | 1f0xA | 1f1eA | 1f1rA | 1f20A | 1f2tA | 1f2tB | 1f5mA | 1f60B | 1f7dA | 1f8rA |
| 1faoA | 1fazA | 1fc6A | 1fcqA | 1fe0A | 1fe6A | 1fhvA | 1fi2A | 1fiuA | 1fm0D | 1fm0E | 1fo6A | 1fo9A |
| 1fp3A | 1fqtA | 1fw9A | 1fx2A | 1fy2A | 1fzvA | 1g01A | 1g12A | 1g2bA | 1g2pA | 1g2rA | 1g60A | 1g6gA |
| 1g87A | 1g8qA | 1g8tA | 1g9rA | 1ga1A | 1gakA | 1gcuA | 1gheA | 1gk8I | 1gkkA | 1gl2A | 1gl2C | 1gl4A |
| 1gl4B | 1gmxA | 1gnyA | 1go3E | 1gotG | 1gouA | 1gpeA | 1gppA | 1gqgA | 1gqiA | 1gr0A | 1gr3A | 1gs9A |
| 1gugA | 1guiA | 1gv3A | 1gv8A | 1gvjA | 1gvnA | 1gvnB | 1gxmA | 1gxtA | 1gxyA | 1gy8A | 1gyhA | 1h0aA |
| 1h0bA | 1h0hB | 1h10A | 1h12A | 1h1nA | 1h2cA | 1h2eA | 1h3nA | 1h4iB | 1h4pA | 1h4xA | 1h6hA | 1h6kX |
| 1h75A | 1h7lA | 1h8pA | 1h98A | 1h99A | 1hdhA | 1hdoA | 1he1A | 1hfeL | 1hfeS | 1hh8A | 1hn0A | 1hq0A |
| 1hruA | 1hvxA | 1hxiA | 1hyuA | 1hz4A | 1hz5A | 1i0rA | 1i19A | 1i1dA | 1i1jA | 1i1qA | 1i24A | 1i27A |
| 1i2hA | 1i36A | 1i4uA | 1i60A | 1i6wA | 1i72A | 1i72B | 1i7kA | 1i7wB | 1i82A | 1i9dA | 1i9gA | 1i9sA |
| 1i9yA | 1iazA | 1ibyA | 1icfA | 1icfI | 1idpA | 1ie0A | 1ie7B | 1ie7C | 1ig3A | 1igqA | 1ihgA | 1ihjA |
| 1ihrA | 1ikoP | 1iktA | 1iooA | 1iq6A | 1iqzA | 1ireA | 1itwA | 1iu1A | 1iwmA | 1ix2A | 1ixkA | 1ixlA |
| 1iynA | 1iz6A | 1izcA | 1j1iA | 1j1tA | 1j1yA | 1j22A | 1j27A | 1j2tA | 1j30A | 1j58A | 1j5xA | 1j6pA |
| 1j75A | 1j7xA | 1j83A | 1j8rA | 1jaxA | 1jb3A | 1jb7A | 1jb7B | 1jb9A | 1jbkA | 1jdwA | 1jerA | 1jfrA |
| 1jfuA | 1jfxA | 1jg1A | 1jgtA | 1jhdA | 1jhjA | 1ji7A | 1jidA | 1jiwI | 1jjfA | 1jkgA | 1jkgB | 1jkmA |
| 1jkuA | 1jl3A | 1jm1A | 1jmkC | 1jmvA | 1jndA | 1jniA | 1jnrB | 1jp3A | 1jr8A | 1js2A | 1jtgB | 1ju2A |
| 1jvwA | 1jwqA | 1jy2O | 1jyhA | 1jykA | 1jysA | 1jztA | 1k0eA | 1k12A | 1k1aA | 1k30A | 1k4nA | 1k6zA |
| 1k75A | 1k7gA | 1k7kA | 1k87A | 1k8wA | 1k92A | 1k9yA | 1kcqA | 1kdgA | 1keaA | 1kfwA | 1kg2A | 1kgdA |
| 1kh0A | 1khbA | 1khiA | 1khxA | 1kidA | 1kl9A | 1kliL | 1klxA | 1kmoA | 1kngA | 1knlA | 1ko7A | 1kolA |
| 1kp6A | 1kptA | 1kq6A | 1kqfA | 1kqfB | 1kqfC | 1kqrA | 1kr7A | 1krhA | 1ktbA | 1ku1A | 1kytA | 1kzqA |
| 1l1dA | 1l2qA | 1l3kA | 1l3pA | 1l5xA | 1l8aA | 1l8jA | 1l9lA | 1l9xA | 1lauE | 1lc5A | 1lg7A | 1li1A |
| 1lj8A | 1ljoA | 1lk9A | 1llmC | 1lm5A | 1lm8B | 1lmiA | 1lmlA | 1ln4A | 1lq9A | 1lr0A | 1lr5A | 1lr7A |
| 1ltuA | 1lu9A | 1lxjA | 1ly1A | 1lyvA | 1lzkA | 1m0uA | 1m0zA | 1m1fA | 1m1pA | 1m21A | 1m33A | 1m38A |
| 1m44A | 1m4rA | 1m55A | 1m5q1 | 1m65A | 1m6yA | 1m93B | 1m9nA | 1mbyA | 1md6A | 1mg4A | 1mg5A | 1mg7A |
| 1mgrA | 1mgtA | 1mj4A | 1mk4A | 1mkiA | 1mkyA | 1mkzA | 1mn8A | 1mo9A | 1mofA | 1mrzA | 1msvA | 1mtpA |
| 1mu7A | 1mwpA | 1mwqA | 1n0qA | 1n1fA | 1n1jA | 1n1jB | 1n57A | 1n5wB | 1n5wC | 1n7zA | 1n93X | 1n97A |
| 1n99A | 1n9gA | 1na3A | 1nc5A | 1nc7A | 1ne7A | 1nepA | 1ng2A | 1ng6A | 1nh2C | 1ni9A | 1njkA | 1njrA |
| 1nkdA | 1nkgA | 1nkxA | 1nlrA | 1nnhA | 1nnwA | 1no5A | 1noxA | 1np3A | 1np8A | 1npuA | 1nqdA | 1nqeA |
| 1nriA | 1nrjA | 1nrjB | 1nrwA | 1nsfA | 1nteA | 1ntfA | 1nu3A | 1nupA | 1nvmA | 1nvmB | 1nwaA | 1nxjA |
| 1nxmA | 1nycA | 1nykA | 1nz0A | 1nzjA | 1o0wA | 1o13A | 1o1mA | 1o1yA | 1o22A | 1o2dA | 1o3uA | 1o4tA |
| 1o4vA | 1o4wA | 1o5eL | 1o5uA | 1o61A | 1o6aA | 1o6sA | 1o75A | 1o7iA | 1o8pA | 1o91A | 1o94C | 1o9gA |
| 1oa7A | 1oaiA | 1ocyA | 1odvA | 1oe4A | 1of1A | 1of3A | 1ofnA | 1ofsA | 1ofwA | 1oghA | 1ogmX | 1ogqA |
| 1ohtA | 1oi2A | 1oioA | 1oipA | 1oj7A | 1ojhA | 1okiA | 1oksA | 1on1A | 1onwA | 1oohA | 1oq1A | 1oqcA |
| 1oqjA | 1oqvA | 1oqwA | 1or0B | 1or7A | 1or7C | 1ornA | 1orrA | 1oruA | 1osyA | 1otmA | 1ouvA | 1ov8A |
| 1ow1A | 1ow3A | 1ow4A | 1ox3A | 1ox7A | 1oxjA | 1oyjA | 1oywA | 1oz9A | 1ozpA | 1p3cA | 1p5uA | 1p5uB |
| 1p99A | 1p9hA | 1pbjA | 1pbtA | 1pbwA | 1pc4A | 1pcfA | 1pfzA | 1pgvA | 1pj5A | 1pkoA | 1pm4A | 1pmhX |
| 1pmmA | 1pn0A | 1pprM | 1ppyA | 1pq4A | 1przA | 1ps3A | 1pswA | 1pt0A | 1pt6A | 1pu6A | 1puoA | 1pv5A |
| 1pvgA | 1pw9A | 1pwaA | 1px5A | 1pxzA | 1py9A | 1pyuB | 1pz4A | 1pz7A | 1pzsA | 1q08A | 1q0rA | 1q16A |
| 1q16B | 1q16C | 1q1cA | 1q33A | 1q40A | 1q44A | 1q4rA | 1q4sA | 1q5zA | 1q7lA | 1q7lB | 1q7tA | 1q8dA |
| 1q8rA | 1q9uA | 1qb5D | 1qb7A | 1qbzA | 1qcoA | 1qcsA | 1qd1A | 1qgeD | 1qgeE | 1qgiA | 1qgwA | 1qgwB |
| 1qh3A | 1qhdA | 1qkrA | 1qlmA | 1qlwA | 1qnoA | 1qo2A | 1qq0A | 1qrlA | 1qsaA | 1qtnA | 1qupA | 1qv9A |
| 1qw2A | 1qwdA | 1qwgA | 1qwiA | 1qwrA | 1qwyA | 1qwzA | 1qxmA | 1qy6A | 1qyuA | 1qz1A | 1qz9A | 1qzgA |
| 1r0uA | 1r1mA | 1r1tA | 1r3dA | 1r4pA | 1r4pB | 1r4vA | 1r4xA | 1r5mA | 1r5zA | 1r62A | 1r6vA | 1r75A |
| 1r77A | 1r7aA | 1r7jA | 1r8oA | 1r8oB | 1r9cA | 1r9fA | 1r9hA | 1r9lA | 1r9xA | 1rc9A | 1regX | 1rewC |
| 1rfsA | 1rfxA | 1rfyA | 1rgxA | 1ri6A | 1rifA | 1rj1A | 1rjdA | 1rjkA | 1rjpA | 1rkiA | 1rkuA | 1rlhA |
| 1rliA | 1rljA | 1rm6B | 1rmgA | 1rp0A | 1rqbA | 1rqpA | 1rsgA | 1rsyA | 1rtqA | 1ru4A | 1rwiA | 1rwjA |
| 1rwrA | 1rxqA | 1rz3A | 1s21A | 1s2oA | 1s2xA | 1s4kA | 1s5aA | 1s5uA | 1s66L | 1s68A | 1s69A | 1s7fA |
| 1s7zA | 1s8nA | 1s9rA | 1sauA | 1sbxA | 1sctB | 1sd4A | 1sdoA | 1sdwA | 1se8A | 1senA | 1sf9A | 1sfsA |
| 1sfxA | 1sgvA | 1sgwA | 1sh8A | 1sjyA | 1smbA | 1smoA | 1sn0A | 1sngA | 1sntA | 1soxA | 1spvA | 1sqsA |
| 1sr4C | 1sraA | 1ss4A | 1surA | 1suuA | 1svfA | 1sw1A | 1swfA | 1sxrA | 1sz7A | 1szhA | 1sznA | 1szwA |
| 1t06A | 1t0bA | 1t0fA | 1t0hA | 1t0hB | 1t0pB | 1t1eA | 1t1jA | 1t2aA | 1t3iA | 1t3mB | 1t3qA | 1t3tA |
| 1t44G | 1t6cA | 1t6t1 | 1t82A | 1t8hA | 1t9hA | 1ta8A | 1ta9A | 1tafA | 1tafB | 1tazA | 1tc5A | 1te2A |
| 1tfjA | 1tifA | 1tiqA | 1tjeA | 1tjmA | 1tk1A | 1tkiA | 1tl2A | 1toaA | 1tolA | 1tp6A | 1tpzA | 1tqiA |
| 1ts9A | 1tt0A | 1tu1A | 1tu9A | 1tuaA | 1tuhA | 1tuwA | 1tvfA | 1tvgA | 1tvnA | 1twdA | 1twuA | 1twyA |
| 1txgA | 1txnA | 1txqB | 1ty9A | 1tzbA | 1tzpA | 1u00A | 1u02A | 1u07A | 1u09A | 1u0kA | 1u17A | 1u1iA |
| 1u2bA | 1u2xA | 1u53A | 1u58A | 1u5dA | 1u5fA | 1u5kA | 1u5pA | 1u5uA | 1u69A | 1u6tA | 1u79A | 1u7hA |
| 1u7lA | 1u7oA | 1u8vA | 1u9dA | 1uaiA | 1uajA | 1ub4C | 1ucrA | 1ufbA | 1ufoA | 1ui0A | 1uiiA | 1uirA |
| 1uiuA | 1uj8A | 1ujmA | 1ujpA | 1ukfA | 1ukkA | 1ulkA | 1ulrA | 1umkA | 1umnA | 1unfX | 1unnC | 1uoyA |
| 1uozA | 1upiA | 1upkA | 1upsA | 1urqA | 1urqD | 1urrA | 1us2A | 1uscA | 1usmA | 1usqA | 1utxA | 1uujA |
| 1uuqA | 1uuzA | 1uv7A | 1uw4A | 1uw4B | 1uwkA | 1uwvA | 1ux6A | 1uxoA | 1uypA | 1v02A | 1v0aA | 1v10A |
| 1v2bA | 1v30A | 1v33A | 1v37A | 1v3zA | 1v54G | 1v57A | 1v5cA | 1v5vA | 1v6cA | 1v6tA | 1v6zA | 1v70A |
| 1v73A | 1v74A | 1v8cA | 1v8hA | 1v8iA | 1v9aA | 1v9yA | 1vajA | 1vbkA | 1vclA | 1vctA | 1vdhA | 1vfyA |
| 1vh4A | 1vheA | 1vhnA | 1vhuA | 1vi3A | 1vimA | 1vioA | 1vj0A | 1vjkA | 1vjnA | 1vjvA | 1vk1A | 1vk2A |
| 1vk4A | 1vk5A | 1vk8A | 1vkaA | 1vkbA | 1vkcA | 1vkfA | 1vkhA | 1vkiA | 1vkkA | 1vkmA | 1vkwA | 1vkyA |
| 1vl2A | 1vl4A | 1vl5A | 1vl7A | 1vlaA | 1vljA | 1vlrA | 1vlyA | 1vm0A | 1vmbA | 1vmeA | 1vmjA | 1vpbA |
| 1vpkA | 1vpmA | 1vpnA | 1vprA | 1vq3A | 1vqsA | 1vqzA | 1vr5A | 1vr6A | 1vr7A | 1vr8A | 1vraA | 1vraB |
| 1vrmA | 1vsrA | 1vtz0 | 1vyiA | 1vzgA | 1vzwA | 1w0hA | 1w0nA | 1w0oA | 1w0uA | 1w1dA | 1w1oA | 1w2lA |
| 1w2wA | 1w2wB | 1w2yA | 1w30A | 1w3oA | 1w44A | 1w4sA | 1w4tA | 1w4wA | 1w53A | 1w5fA | 1w66A | 1w79A |
| 1w7fA | 1w7oA | 1w80A | 1w94A | 1w9aA | 1w9hA | 1wakA | 1wawA | 1wc1A | 1wc2A | 1wchA | 1wckA | 1wcwA |
| 1wd3A | 1wd5A | 1wdeA | 1wdjA | 1wdyA | 1wehA | 1wgbA | 1whzA | 1wiwA | 1wk8A | 1wkaA | 1wkvA | 1wkyA |
| 1wleA | 1wlgA | 1wljA | 1wlzA | 1wmdA | 1wmhB | 1wmwA | 1wn5A | 1wn9A | 1wnhA | 1woqA | 1wpbA | 1wpnA |
| 1wr8A | 1wraA | 1wrjA | 1ws7A | 1wv3A | 1wvqA | 1wwiA | 1wwzA | 1wx2B | 1wybA | 1wyxA | 1wz3A | 1wzaA |
| 1wznA | 1wzzA | 1x0tA | 1x1nA | 1x1zA | 1x2iA | 1x3kA | 1x46A | 1x6iA | 1x6oA | 1x6vA | 1x74A | 1x7vA |
| 1x8bA | 1x99A | 1x9dA | 1xa1A | 1xa3A | 1xcrA | 1xdnA | 1xdzA | 1xe1A | 1xe7A | 1xedA | 1xerA | 1xfiA |
| 1xfkA | 1xhnA | 1xiwA | 1xiwB | 1xiyA | 1xjeA | 1xjtA | 1xjuA | 1xkfA | 1xkgA | 1xklA | 1xkpA | 1xkpB |
| 1xkpC | 1xkrA | 1xlyA | 1xmbA | 1xmtA | 1xnfA | 1xocA | 1xovA | 1xqaA | 1xqhA | 1xqoA | 1xrjA | 1xszA |
| 1xt5A | 1xt8A | 1xteA | 1xtpA | 1xuuA | 1xv2A | 1xvhA | 1xvwA | 1xvxA | 1xxqA | 1xy7A | 1xzzA | 1y02A |
| 1y07A | 1y08A | 1y0bA | 1y0hA | 1y0kA | 1y0uA | 1y12A | 1y1eX | 1y42X | 1y43B | 1y4cA | 1y4hA | 1y5yA |
| 1y63A | 1y65A | 1y66A | 1y7bA | 1y7rA | 1y7wA | 1y7yA | 1y81A | 1y88A | 1y89A | 1y8aA | 1y9aA | 1y9qA |
| 1yacA | 1yb3A | 1ybzA | 1yc9A | 1ycdA | 1ydgA | 1ydyA | 1yf9A | 1yggA | 1yhfA | 1yhtA | 1yj7A | 1ykdA |
| 1yleA | 1yliA | 1ylkA | 1yllA | 1ylmA | 1ylxA | 1ym3A | 1yn9A | 1ynsA | 1yo3A | 1yo5C | 1yocA | 1yp5A |
| 1ypyA | 1yq5A | 1yqhA | 1yqtA | 1yr2A | 1ys3A | 1yt3A | 1yt8A | 1ytlA | 1yu0A | 1yu6C | 1yulA | 1yuxA |
| 1yw5A | 1ywfA | 1ywmA | 1yx1A | 1yxmA | 1yxyA | 1yzfA | 1yzvA | 1z05A | 1z0bA | 1z0jB | 1z0mA | 1z0sA |
| 1z21A | 1z2nX | 1z3eA | 1z3xA | 1z45A | 1z4eA | 1z5gA | 1z67A | 1z6mA | 1z6oA | 1z6oM | 1z7xW | 1z84A |
| 1z8gA | 1z8kA | 1z9nA | 1za0A | 1zbfA | 1zchA | 1zcjA | 1zcuA | 1zdeA | 1ze3D | 1ze3H | 1zejA | 1zelA |
| 1zgxA | 1zh5A | 1zhhA | 1zhhB | 1zhqA | 1zhtA | 1zhvA | 1zjcA | 1zjrA | 1zkeA | 1zkiA | 1zkkA | 1zkpA |
| 1zkrA | 1zldA | 1zlhB | 1zljA | 1zmaA | 1zn6A | 1zp9A | 1zpsA | 1zpvA | 1zpwX | 1zr6A | 1zrsA | 1zs4A |
| 1zs7A | 1zsqA | 1zswA | 1zsyA | 1zuoA | 1zvaA | 1zvtA | 1zwyA | 1zxtA | 1zxuA | 1zy7A | 1zzgA | 2a0kA |
| 2a0mA | 2a10A | 2a13A | 2a15A | 2a1iA | 2a2mA | 2a35A | 2a3mA | 2a3nA | 2a4aA | 2a4dA | 2a4oA | 2a4wA |
| 2a50A | 2a50B | 2a65A | 2a67A | 2a6bA | 2a6cA | 2a6vA | 2a6yA | 2a75A | 2a78B | 2a9iA | 2a9sA | 2a9yA |
| 2aanA | 2ab0A | 2abkA | 2abwA | 2acfA | 2aeeA | 2aexA | 2agkA | 2aheA | 2ahnA | 2ahuA | 2aj6A | 2aj7A |
| 2amhA | 2amlA | 2amuA | 2an1A | 2anvA | 2ao9A | 2ap1A | 2apjA | 2apoA | 2aq5A | 2ar1A | 2ar5A | 2arzA |
| 2as0A | 2as9A | 2asbA | 2asfA | 2au3A | 2auwA | 2avkA | 2axcA | 2axiA | 2axoA | 2axqA | 2axwA | 2aydA |
| 2az4A | 2azwA | 2b06A | 2b0aA | 2b0jA | 2b0pA | 2b0vA | 2b1lA | 2b1xA | 2b1xB | 2b1yA | 2b3bA | 2b3hA |
| 2b3mA | 2b4hA | 2b4vA | 2b4wA | 2b5aA | 2b5vA | 2b61A | 2b7uA | 2b8iA | 2b8mA | 2b9eA | 2b9lA | 2b9wA |
| 2bayA | 2bbeA | 2bbhA | 2bbrA | 2bc0A | 2bcmA | 2bdrA | 2bezC | 2bfwA | 2bg1A | 2bgiA | 2bhuA | 2bi0A |
| 2bibA | 2biiA | 2bivA | 2bjbA | 2bjnA | 2bjrA | 2bjuA | 2bjvA | 2bk8A | 2bk9A | 2bkfA | 2blfB | 2bnlA |
| 2bo4A | 2bq4A | 2bryA | 2bs2A | 2bsjA | 2bswA | 2bv9A | 2bvfA | 2bw0A | 2bw3A | 2bw3B | 2bwmA | 2bydA |
| 2bzuA | 2c0hA | 2c0nA | 2c1dA | 2c1gA | 2c1lA | 2c1xA | 2c21A | 2c2fA | 2c2iA | 2c2nA | 2c2pA | 2c31A |
| 2c3fA | 2c3nA | 2c46A | 2c4bA | 2c4iA | 2c54A | 2c5lC | 2c5qA | 2c60A | 2c71A | 2c8sA | 2c9wA | 2cayA |
| 2cb2A | 2cb9A | 2cc0A | 2ccmA | 2ccvA | 2cd9A | 2ce0A | 2cgqA | 2ch5A | 2chcA | 2chlA | 2ci9A | 2cinA |
| 2ciqA | 2ciuA | 2cjpA | 2cjsA | 2cjtA | 2ckiA | 2ckkA | 2cklB | 2cl2A | 2cl3A | 2cm1A | 2cm5A | 2covD |
| 2cqsA | 2cs7A | 2cu3A | 2culA | 2cvbA | 2cveA | 2cviA | 2cvjA | 2cw9A | 2cwcA | 2cwlA | 2cwrA | 2cwsA |
| 2cwyA | 2cwzA | 2cxaA | 2cxcA | 2cxhA | 2cxiA | 2cxxA | 2cxyA | 2cy4A | 2cyeA | 2cyjA | 2cz4A | 2czqA |
| 2d0oA | 2d0oB | 2d1cA | 2d1gA | 2d1lA | 2d39A | 2d3yA | 2d4oA | 2d58A | 2d5wA | 2d68A | 2d73A | 2d80A |
| 2d8dA | 2db7A | 2dbbA | 2ddxA | 2de2A | 2debA | 2dfyC | 2dgaA | 2dh4A | 2dkaA | 2dkhA | 2dkvA | 2dlbA |
| 2dplA | 2dqaA | 2drvA | 2dskA | 2dstA | 2dsyA | 2dtcA | 2duyA | 2dvkA | 2dxaA | 2dxqA | 2dy0A | 2dyhA |
| 2dyjA | 2dyuA | 2e11A | 2e1nA | 2e26A | 2e2nA | 2e4lA | 2e4mC | 2e56A | 2e5bA | 2e7vA | 2e7zA | 2e8gA |
| 2eabA | 2eaqA | 2eb1A | 2ebbA | 2ebfX | 2eceA | 2eenA | 2eeyA | 2efvA | 2eg3A | 2eh3A | 2ehpA | 2ei9A |
| 2ej8A | 2ej9A | 2ejwA | 2ek8A | 2ekgA | 2ekyA | 2elfA | 2eo4A | 2epkX | 2eqaA | 2erbA | 2ervA | 2erwA |
| 2es4D | 2essA | 2etjA | 2etvA | 2etwA | 2ev1A | 2evaA | 2evrA | 2ew2A | 2ewfA | 2ewhA | 2ewrA | 2ewtA |
| 2ex2A | 2eyuA | 2f0cA | 2f1kA | 2f22A | 2f46A | 2f4mA | 2f4mB | 2f4pA | 2f4qA | 2f51A | 2f5tX | 2f6eA |
| 2f6jA | 2f6lA | 2f6rA | 2f7bA | 2f7fA | 2f7vA | 2f8yA | 2f93B | 2f9fA | 2f9hA | 2f9iB | 2fa1A | 2fa5A |
| 2faoA | 2fawA | 2fb5A | 2fb6A | 2fbhA | 2fbnA | 2fbqA | 2fckA | 2fd5A | 2fdrA | 2fe3A | 2feaA | 2fefA |
| 2fexA | 2fezA | 2ffcA | 2ffuA | 2fgzA | 2fhpA | 2fhzA | 2fi1A | 2fi9A | 2fimA | 2fjrA | 2fjzA | 2fk5A |
| 2fk9A | 2fkbA | 2fkkA | 2fl4A | 2fmmE | 2fnaA | 2fnoA | 2fozA | 2fp7B | 2fprA | 2fq3A | 2fqpA | 2fqwA |
| 2fr2A | 2freA | 2frgP | 2fs2A | 2fs5A | 2fsjA | 2fsqA | 2fsrA | 2fssA | 2fsuA | 2fsxA | 2ftrA | 2ftxB |
| 2fu4A | 2fukA | 2fulA | 2fupA | 2furA | 2fvhA | 2fvvA | 2fw5A | 2fwtA | 2fwvA | 2fyfA | 2fygA | 2fyqA |
| 2fyxA | 2fzpA | 2fzvA | 2g0wA | 2g16A | 2g1uA | 2g2sB | 2g3aA | 2g3bA | 2g40A | 2g5gX | 2g5xA | 2g62A |
| 2g7cA | 2g7oA | 2g7sA | 2g84A | 2g9gA | 2g9zA | 2ga1A | 2ga8A | 2gagA | 2gagB | 2gagC | 2gaiA | 2gakA |
| 2gcuA | 2gecA | 2genA | 2geyA | 2gfnA | 2gfoA | 2gfqA | 2ghsA | 2giaA | 2giaB | 2gibA | 2giyA | 2gj3A |
| 2gk4A | 2gkpA | 2glzA | 2gmqA | 2gmsA | 2gmwA | 2gmyA | 2gn4A | 2gnpA | 2gnqA | 2gokA | 2gpiA | 2gpyA |
| 2gq0A | 2gqtA | 2gr8A | 2gsdA | 2gsfA | 2gsnA | 2gu3A | 2guhA | 2guiA | 2gviA | 2gwgA | 2gwlA | 2gwnA |
| 2gx5A | 2gyqA | 2gz4A | 2h0uA | 2h12A | 2h1tA | 2h2kA | 2h2rA | 2h30A | 2h3hA | 2h5fA | 2h5yA | 2h6lA |
| 2h7oA | 2h7xA | 2h8gA | 2h8oA | 2h98A | 2h9uA | 2ha8A | 2hanA | 2hanB | 2hb6A | 2hboA | 2hcfA | 2hcmA |
| 2hcvA | 2hdoA | 2hdwA | 2hekA | 2hewF | 2hhcA | 2hhgA | 2hhzA | 2hi0A | 2himA | 2hinA | 2hiyA | 2hjhA |
| 2hjnA | 2hk0A | 2hkvA | 2hl7A | 2hljA | 2hlqA | 2hlsA | 2hlyA | 2hmcA | 2hnlA | 2hnuA | 2ho3A | 2hoqA |
| 2hp0A | 2hpgA | 2hplA | 2hpsA | 2hq4A | 2hq7A | 2hq9A | 2hqsC | 2hqyA | 2hrxA | 2hrzA | 2hsbA | 2hsiA |
| 2hsjA | 2hszA | 2ht9A | 2htaA | 2htdA | 2hu9A | 2huhA | 2hujA | 2hv8D | 2hw2A | 2hw4A | 2hx5A | 2hxiA |
| 2hy1A | 2hy5B | 2hy5C | 2hy7A | 2hytA | 2hyxA | 2hzkA | 2hzpA | 2i02A | 2i0zA | 2i13A | 2i2oA | 2i3dA |
| 2i3fA | 2i3yA | 2i47A | 2i48A | 2i4lA | 2i4sA | 2i51A | 2i5iA | 2i5rA | 2i5uA | 2i6dA | 2i6hA | 2i6vA |
| 2i71A | 2i7aA | 2i7fA | 2i7gA | 2i8bA | 2i8eA | 2i8tA | 2i9aA | 2i9cA | 2i9iA | 2i9wA | 2i9xA | 2ia4A |
| 2ia7A | 2iabA | 2iaiA | 2iayA | 2ib0A | 2ibaA | 2ibdA | 2iblA | 2ibnA | 2ic2A | 2ic6A | 2iccA | 2icgA |
| 2ichA | 2icpA | 2id3A | 2id4A | 2idlA | 2ieqA | 2if6A | 2ifxA | 2ig6A | 2ig8A | 2igpA | 2igtA | 2ihyA |
| 2ii1A | 2ii2A | 2ikbA | 2ikkA | 2iksA | 2ilrA | 2im9A | 2imdA | 2imgA | 2imhA | 2imjA | 2imrA | 2imzA |
| 2in3A | 2inbA | 2inuA | 2ionA | 2ip2A | 2ip6A | 2iplA | 2iqjA | 2iqyA | 2iruA | 2is9A | 2isbA | 2isyA |
| 2it9A | 2iu1A | 2iu5A | 2iucA | 2iumA | 2iuwA | 2iv7A | 2ivfA | 2ivfB | 2ivfC | 2iw0A | 2iw2A | 2ixdA |
| 2ixsA | 2iy2A | 2iy9A | 2iyfA | 2izzA | 2j0aA | 2j12B | 2j1aA | 2j43A | 2j4xA | 2j5cA | 2j5gA | 2j5yA |
| 2j6aA | 2j6bA | 2j6vA | 2j71A | 2j7qA | 2j7vA | 2j80A | 2j89A | 2j8bA | 2j8hA | 2j8kA | 2j91A | 2j9cA |
| 2j9fB | 2j9wA | 2jaeA | 2jbvA | 2jbzA | 2jcbA | 2jcnA | 2jd9A | 2jdjA | 2je2A | 2je6I | 2je8A | 2jekA |
| 2jemA | 2jepA | 2jerA | 2jffA | 2jfnA | 2jg1A | 2jgbA | 2jgpA | 2jh1A | 2jh3A | 2jhjA | 2jiiA | 2jisA |
| 2jjqA | 2jjsC | 2jlhA | 2jliA | 2jlpA | 2nmuA | 2nn5A | 2no0A | 2no4A | 2np5A | 2nptB | 2nq3A | 2nr5A |
| 2nr7A | 2nrkA | 2nrrA | 2ns0A | 2nsfA | 2nsqA | 2nt8A | 2nuhA | 2nujA | 2nukA | 2nv9A | 2nwhA | 2nx2A |
| 2nx4A | 2nx8A | 2nxfA | 2nxvA | 2nxwA | 2nyhA | 2nyiA | 2nzlA | 2nzwA | 2o0hA | 2o0mA | 2o0yA | 2o11A |
| 2o16A | 2o1aA | 2o1bA | 2o2cA | 2o2gA | 2o2kA | 2o2vB | 2o2xA | 2o37A | 2o3fA | 2o4dA | 2o4vA | 2o4xB |
| 2o57A | 2o5hA | 2o5vA | 2o62A | 2o66A | 2o6cA | 2o6lA | 2o6pA | 2o70A | 2o71A | 2o7aA | 2o7iA | 2o7rA |
| 2o8pA | 2o8qA | 2o95A | 2oa2A | 2oa9A | 2oafA | 2oaiA | 2ob0A | 2ob5A | 2oblA | 2obpA | 2oc5A | 2oc6A |
| 2ocgA | 2ochA | 2oczA | 2od0A | 2od4A | 2od5A | 2od6A | 2odaA | 2odfA | 2odhA | 2odkA | 2odlA | 2oebA |
| 2of3A | 2ofyA | 2og2A | 2ogfA | 2ogiA | 2ogxA | 2oh1A | 2oh3A | 2oh5A | 2ohwA | 2oikA | 2oiwA | 2ojhA |
| 2okgA | 2okmA | 2okqA | 2okuA | 2okvA | 2okxA | 2olmA | 2olnA | 2olpA | 2omdA | 2omkA | 2omlA | 2omoA |
| 2onfA | 2oo0A | 2oo3A | 2oo6A | 2oocA | 2oojA | 2ookA | 2opcA | 2opjA | 2oplA | 2opoA | 2oq6A | 2oqkA |
| 2oqmA | 2oqyA | 2oswA | 2ot4A | 2otmA | 2ou3A | 2ou5A | 2ou6A | 2ouwA | 2ov9A | 2ovjA | 2owaA | 2ownA |
| 2owpA | 2ox5A | 2ox6A | 2ox7A | 2oxlA | 2oxvA | 2oy9A | 2oycA | 2oynA | 2oyzA | 2ozeA | 2ozgA | 2ozhA |
| 2ozjA | 2oztA | 2ozvA | 2ozyA | 2p0aA | 2p0dA | 2p0eA | 2p0nA | 2p0sA | 2p0wA | 2p12A | 2p14A | 2p17A |
| 2p18A | 2p1gA | 2p1mB | 2p2rA | 2p35A | 2p39A | 2p3hA | 2p3pA | 2p3yA | 2p4fA | 2p4oA | 2p4xA | 2p51A |
| 2p57A | 2p58C | 2p5dA | 2p5kA | 2p5mA | 2p6vA | 2p6wA | 2p7hA | 2p8gA | 2p8jA | 2p8uA | 2p97A | 2p9bA |
| 2p9hA | 2p9oA | 2p9wA | 2p9xA | 2pa4A | 2pa7A | 2pa8D | 2pa8L | 2pagA | 2pb7A | 2pbiA | 2pc1A | 2pcxA |
| 2pd1A | 2pe4A | 2pebA | 2petA | 2pfiA | 2pfwA | 2pfyA | 2pgeA | 2pgnA | 2ph0A | 2phnA | 2pieA | 2pjsA |
| 2pjzA | 2pk8A | 2pkeA | 2pkfA | 2pliA | 2pljA | 2plqA | 2plwA | 2pmaA | 2pmrA | 2pmuA | 2pn0A | 2pn1A |
| 2pn2A | 2pnwA | 2pnyA | 2pokA | 2posA | 2ppqA | 2pptA | 2ppxA | 2pq7A | 2pqvA | 2pr5A | 2pr7A | 2prvA |
| 2prxA | 2pshA | 2pszA | 2pttA | 2pttB | 2puyA | 2pv7A | 2pvuA | 2pvzA | 2pxxA | 2pyqA | 2pytA | 2pywA |
| 2pyxA | 2pz0A | 2pzgA | 2pzjA | 2q03A | 2q0qA | 2q0tA | 2q0yA | 2q0zX | 2q2fA | 2q2hA | 2q30A | 2q3xA |
| 2q5cA | 2q5wD | 2q5xA | 2q62A | 2q73A | 2q79A | 2q7bA | 2q7sA | 2q82A | 2q86A | 2q88A | 2q8kA | 2q8nA |
| 2q8rE | 2q9fA | 2q9rA | 2q9uA | 2q9vA | 2qa1A | 2qacA | 2qapA | 2qb5A | 2qb6A | 2qbwA | 2qc1B | 2qc5A |
| 2qcuA | 2qe6A | 2qe8A | 2qe9A | 2qecA | 2qeeA | 2qeuA | 2qf4A | 2qf9A | 2qfaB | 2qfeA | 2qfnA | 2qg8A |
| 2qguA | 2qgyA | 2qh1A | 2qhkA | 2qhpA | 2qhqA | 2qhsA | 2qhtA | 2qibA | 2qikA | 2qipA | 2qiwA | 2qiyA |
| 2qj2A | 2qjvA | 2qjwA | 2qk0A | 2qk1A | 2qkdA | 2qkhA | 2qkpA | 2ql8A | 2qltA | 2qlwA | 2qlxA | 2qm0A |
| 2qmaA | 2qmlA | 2qmqA | 2qn4A | 2qngA | 2qniA | 2qnkA | 2qnlA | 2qntA | 2qp2A | 2qpxA | 2qq5A | 2qq8A |
| 2qqjA | 2qqyA | 2qqzA | 2qr6A | 2qruA | 2qs9A | 2qsaA | 2qsiA | 2qsxA | 2qt7A | 2qtfA | 2qtlA | 2qtqA |
| 2qtwB | 2quoA | 2qupA | 2qv5A | 2qv6A | 2qv8A | 2qvkA | 2qw5A | 2qwuA | 2qx2A | 2qycA | 2qzcA | 2qztA |
| 2qzuA | 2r01A | 2r09A | 2r0cA | 2r0fA | 2r0yA | 2r11A | 2r16A | 2r1bA | 2r1iA | 2r2aA | 2r2cA | 2r2dA |
| 2r2yA | 2r2zA | 2r32A | 2r37A | 2r3aA | 2r44A | 2r47A | 2r48A | 2r4gA | 2r4iA | 2r5oA | 2r6oA | 2r6qA |
| 2r6vA | 2r77A | 2r78A | 2r7dA | 2r7hA | 2r8qA | 2r8uA | 2ra8A | 2ra9A | 2rafA | 2rasA | 2rauA | 2rb7A |
| 2rbcA | 2rbdA | 2rbgA | 2rc3A | 2rc7A | 2rccA | 2rchA | 2rciA | 2rdcA | 2rdnA | 2re2A | 2reeA | 2regA |
| 2rekA | 2rerA | 2reuA | 2rf6A | 2rfaA | 2rffA | 2rfmA | 2rfrA | 2rgqA | 2rh0A | 2rh3A | 2rhfA | 2rhiA |
| 2rhkC | 2rhmA | 2rijA | 2rikA | 2rilA | 2riqA | 2rjiA | 2rk5A | 2rk9A | 2rklA | 2rldA | 2uurA | 2uuuA |
| 2uv4A | 2uvkA | 2uvpA | 2uwiA | 2uwjE | 2uxwA | 2uz0A | 2uz8A | 2v0uA | 2v1oA | 2v25A | 2v27A | 2v2fF |
| 2v2gA | 2v2kA | 2v33A | 2v36B | 2v3dA | 2v3sA | 2v40A | 2v4xA | 2v57A | 2v5jA | 2v5tA | 2v76A | 2v79A |
| 2v7iA | 2v7sA | 2v8iA | 2v9kA | 2v9vA | 2vbeA | 2vbfA | 2vc8A | 2vceA | 2vcgA | 2vckA | 2ve8A | 2vebA |
| 2vecA | 2vefA | 2vezA | 2vfkA | 2vfrA | 2vg2A | 2vgaA | 2vgxA | 2vh3A | 2vifA | 2vjiA | 2vjwA | 2vk2A |
| 2vk3A | 2vkjA | 2vkpA | 2vlaA | 2vlbA | 2vlgA | 2vliA | 2vm5A | 2vm9A | 2vn4A | 2vn5A | 2vn5B | 2vngA |
| 2vnsA | 2vnvA | 2vo9A | 2vovA | 2vozA | 2vpeA | 2vphA | 2vptA | 2vq2A | 2vqgA | 2vqpA | 2vqrA | 2vqxA |
| 2vsdA | 2vt1B | 2vtcA | 2vtvA | 2vtwA | 2vu4A | 2vunA | 2vveA | 2vvnA | 2vvwA | 2vwsA | 2vx4A | 2vxrA |
| 2vxzA | 2vy7A | 2vyoA | 2vyrA | 2vyrE | 2vywA | 2vz5A | 2vzpA | 2vzyA | 2w11A | 2w18A | 2w1pA | 2w1qA |
| 2w1rA | 2w2aA | 2w2jA | 2w2kA | 2w2rA | 2w31A | 2w38A | 2w3jA | 2w3oA | 2w3pA | 2w3qA | 2w3vA | 2w3zA |
| 2w4eA | 2w50A | 2w5fA | 2w5nA | 2w61A | 2w6aA | 2w7aA | 2w7nA | 2w7zA | 2w83C | 2w8jA | 2w8xA | 2w91A |
| 2w9xA | 2waaA | 2wabA | 2wagA | 2wanA | 2wawA | 2wb3A | 2wbfX | 2wbnA | 2wboA | 2wbxA | 2wciA | 2wcoA |
| 2wcpA | 2wcwA | 2wdcA | 2wefA | 2wfoA | 2wfpA | 2wfwA | 2wg7A | 2wgkA | 2wgpA | 2whjA | 2wivA | 2wj5A |
| 2wj9A | 2wjqA | 2wk1A | 2wkpA | 2wlaA | 2wlcA | 2wlrA | 2wm8A | 2wmiA | 2wmlA | 2wn4A | 2wnhA | 2wnkA |
| 2wnsA | 2wnwA | 2wnxA | 2wokA | 2wpvA | 2wq4A | 2wshA | 2wt9A | 2wteA | 2wtgA | 2wtmA | 2wtoA | 2wu9A |
| 2wuhA | 2wujA | 2wuqA | 2wuxA | 2wv3A | 2wvxA | 2ww5A | 2ww8A | 2wweA | 2wwxB | 2wx9A | 2wy3A | 2wy3B |
| 2wy7Q | 2wyhA | 2wz8A | 2wz9A | 2wzoA | 2wzvA | 2wzxA | 2x0kA | 2x1cA | 2x1zM | 2x26A | 2x2oA | 2x2sA |
| 2x2uA | 2x2zA | 2x35A | 2x36A | 2x3aA | 2x3gA | 2x3hA | 2x3mA | 2x3nA | 2x47A | 2x49A | 2x4jA | 2x4lA |
| 2x5cA | 2x5fA | 2x5nA | 2x5pA | 2x5rA | 2x5yA | 2x7mA | 2x8tA | 2x8xX | 2x98A | 2x9lA | 2x9oA | 2x9zA |
| 2xauA | 2xb1A | 2xbgA | 2xbuA | 2xc1A | 2xcyA | 2xdgA | 2xdhA | 2xdpA | 2xe4A | 2xepA | 2xetA | 2xeuA |
| 2xevA | 2xf1A | 2xffA | 2xfgA | 2xfgB | 2xfvA | 2xgrA | 2xhaA | 2xhgA | 2xhhA | 2xi9A | 2xigA | 2xijA |
| 2xitA | 2xiwA | 2xjpA | 2xmeA | 2xmoA | 2xmzA | 2xomA | 2xp0A | 2xqhA | 2xqoA | 2xsaA | 2xseA | 2xsqA |
| 2xsrA | 2xswA | 2xt2A | 2xtlA | 2xtpA | 2xtsA | 2xtsB | 2xu8A | 2xuaA | 2xveA | 2xvsA | 2xvxA | 2xw6A |
| 2xw7A | 2xwxA | 2xxlA | 2xxzA | 2xz2A | 2xz3A | 2xz7A | 2xz8A | 2y08A | 2y0oA | 2y1qA | 2y2zA | 2y32A |
| 2y33A | 2y39A | 2y3nB | 2y3vA | 2y3wA | 2y43A | 2y4oA | 2y4rA | 2y4zA | 2y51A | 2y5pA | 2y69D | 2y69E |
| 2y69F | 2y69G | 2y69H | 2y69I | 2y69J | 2y6uA | 2y75A | 2y78A | 2y8kA | 2y8wA | 2ya0A | 2yanA | 2yb1A |
| 2ybdA | 2yeuA | 2yfrA | 2yg8A | 2ygoA | 2yh5A | 2yh6A | 2yh9A | 2yhaA | 2yhgA | 2yhsA | 2yilA | 2yleA |
| 2yqzA | 2yr4A | 2yskA | 2yv9A | 2yveA | 2yvmA | 2yvsA | 2yvtA | 2yw3A | 2ywiA | 2yxbA | 2yxoA | 2yygA |
| 2yyhA | 2yyvA | 2yyyA | 2yzjA | 2yzkA | 2yzqA | 2z0aA | 2z0bA | 2z0jA | 2z0qA | 2z0zA | 2z13A | 2z1aA |
| 2z1cA | 2z38A | 2z51A | 2z66A | 2z6dA | 2z6iA | 2z7bA | 2z7fI | 2z83A | 2z84A | 2z8dA | 2z8xA | 2z9uA |
| 2za0A | 2zcaA | 2zcmA | 2zcoA | 2zcwA | 2zczA | 2zdpA | 2ze3A | 2zewA | 2zf9A | 2zfdA | 2zfdB | 2zfnA |
| 2zfuA | 2zfzA | 2zgdA | 2zggA | 2zgiA | 2zglA | 2zjdA | 2zonG | 2zooA | 2zouA | 2zplA | 2zq5A | 2zqeA |
| 2zqnA | 2zruA | 2zscA | 2zshB | 2ztjA | 2zumA | 2zuyA | 2zvbA | 2zvsA | 2zw2A | 2zwnA | 2zwsA | 2zx0A |
| 2zxjA | 2zxqA | 2zxyA | 2zycA | 2zyrA | 2zzjA | 2zzs1 | 2zzvA | 3a07A | 3a0jA | 3a0nA | 3a0sA | 3a0tA |
| 3a15A | 3a1cA | 3a21A | 3a2pA | 3a2yA | 3a35A | 3a3pB | 3a47A | 3a4lA | 3a4uB | 3a54A | 3a57A | 3a5pA |
| 3a5yA | 3a64A | 3a68A | 3a9fA | 3a9rA | 3aabA | 3aapA | 3abdA | 3acfA | 3adoA | 3adrA | 3adyA | 3aeiA |
| 3afgA | 3afoA | 3ag7A | 3ahcA | 3ai4A | 3ai5A | 3ai9X | 3aiiA | 3aj3A | 3ajvA | 3ajvB | 3akaA | 3akjA |
| 3al2A | 3anoA | 3anuA | 3apqA | 3apsA | 3atqA | 3atsA | 3au4A | 3av9A | 3awfA | 3axgA | 3azoA | 3b02A |
| 3b08B | 3b1bA | 3b33A | 3b42A | 3b49A | 3b4nA | 3b4oA | 3b4qA | 3b4uA | 3b4yA | 3b5eA | 3b5nB | 3b5nC |
| 3b5nD | 3b5oA | 3b64A | 3b6eA | 3b6hA | 3b6zA | 3b74A | 3b79A | 3b7cA | 3b7hA | 3b8bA | 3b8fA | 3b8lA |
| 3b9tA | 3b9wA | 3ba3A | 3balA | 3bb7A | 3bb9A | 3bbyA | 3bc1B | 3bc8A | 3bc9A | 3bciA | 3bcwA | 3bcyA |
| 3bd1A | 3bdeA | 3bdfA | 3bdiA | 3bdlA | 3bdvA | 3bedA | 3bemA | 3bf5A | 3bf7A | 3bfmA | 3bhdA | 3bhnA |
| 3bhqA | 3bioA | 3bipA | 3biyA | 3bjcA | 3bjdA | 3bjeA | 3bjnA | 3bk5A | 3bkbA | 3bkpA | 3bkrA | 3bkxA |
| 3blnA | 3blzA | 3bm3A | 3bm7A | 3bmxA | 3bmzA | 3bosA | 3bovA | 3bp1A | 3bpkA | 3bptA | 3bq3A | 3bqeA |
| 3bqxA | 3brcA | 3brqA | 3bs1A | 3bs3A | 3bs4A | 3bs5A | 3bs5B | 3buuA | 3bv6A | 3bv8A | 3bvjA | 3bwlA |
| 3bwsA | 3bwvA | 3bwxA | 3bx4B | 3by4A | 3by8A | 3by9A | 3bypA | 3bzhA | 3bzlC | 3bzpA | 3bzwA | 3c0fB |
| 3c18A | 3c1dA | 3c22A | 3c24A | 3c26A | 3c2qA | 3c3pA | 3c4bA | 3c4mA | 3c5hA | 3c5kA | 3c5vA | 3c5yA |
| 3c65A | 3c67A | 3c6aA | 3c6cA | 3c6vA | 3c6wA | 3c85A | 3c8cA | 3c8eA | 3c8iA | 3c8lA | 3c8mA | 3c8uA |
| 3c8wA | 3c8zA | 3c96A | 3c9aA | 3c9fA | 3c9hA | 3c9pA | 3c9qA | 3ca8A | 3caiA | 3canA | 3caoA | 3cawA |
| 3cbjA | 3cbnA | 3cbtA | 3cbwA | 3cc1A | 3cc8A | 3ccfA | 3ce7A | 3cecA | 3cexA | 3cf4A | 3cf4G | 3cggA |
| 3cgxA | 3ch4B | 3chjA | 3chmA | 3ci6A | 3cinA | 3citA | 3civA | 3ciwA | 3cj1A | 3cjdA | 3cjeA | 3cjmA |
| 3cjnA | 3cjpA | 3cjsA | 3cjsB | 3cjyA | 3ck1A | 3ck6A | 3ckmA | 3cmfA | 3cneA | 3cngA | 3cnhA | 3cnrA |
| 3cnvA | 3cnyA | 3co1A | 3co4A | 3cooA | 3cp0A | 3cp3A | 3cp5A | 3cp7A | 3cpgA | 3cpqA | 3cqbA | 3cqvA |
| 3csbA | 3cseA | 3csrA | 3ct6A | 3ctbA | 3ctpA | 3ctzA | 3cu0A | 3cu2A | 3cu3A | 3cuzA | 3cvjA | 3cvoA |
| 3cw3A | 3cwrA | 3cwvA | 3cxmA | 3cxnA | 3cypB | 3cz6A | 3czpA | 3czxA | 3d01A | 3d02A | 3d0fA | 3d0jA |
| 3d0kA | 3d0wA | 3d1bA | 3d1pA | 3d2lA | 3d2qA | 3d2wA | 3d33A | 3d34A | 3d3sA | 3d3yA | 3d3zA | 3d40A |
| 3d4eA | 3d4iA | 3d59A | 3d5pA | 3d6fB | 3d6iA | 3d6jA | 3d79A | 3d7iA | 3d7jA | 3d85C | 3d8bA | 3d8nA |
| 3d9iA | 3d9tA | 3d9xA | 3da0A | 3da8A | 3dalA | 3daoA | 3db0A | 3db2A | 3dboB | 3dcdA | 3dciA | 3dcmX |
| 3dcyA | 3dczA | 3dd7A | 3ddcB | 3ddhA | 3ddjA | 3deoA | 3dewA | 3df7A | 3df8A | 3dfgA | 3dgpA | 3dgpB |
| 3dgtA | 3dhaA | 3dhgA | 3dhhE | 3dhnA | 3di4A | 3dj9A | 3djdA | 3djlA | 3dkmA | 3dkrA | 3dlcA | 3dlmA |
| 3dloA | 3dlqR | 3dluA | 3dlzA | 3dm7A | 3dm8A | 3dmcA | 3dmeA | 3dmfA | 3dmnA | 3dn7A | 3dnfA | 3dnhA |
| 3dnjA | 3dnpA | 3dnxA | 3do8A | 3dp5A | 3dpgA | 3dpjA | 3dr2A | 3draA | 3draB | 3drfA | 3drzA | 3ds8A |
| 3dsbA | 3dshA | 3dsmA | 3dt5A | 3dttA | 3dtzA | 3du1X | 3dupA | 3dv9A | 3dwgC | 3dxlA | 3dz1A | 3dzaA |
| 3dzeA | 3e0xA | 3e0zA | 3e11A | 3e15A | 3e18A | 3e19A | 3e23A | 3e2dA | 3e2vA | 3e3mA | 3e3uA | 3e3xA |
| 3e46A | 3e48A | 3e4vA | 3e4wA | 3e57A | 3e58A | 3e6qA | 3e78A | 3e7hA | 3e7pA | 3e8oA | 3e8tA | 3e96A |
| 3e99A | 3eafA | 3ebbA | 3eblA | 3ebtA | 3ebyA | 3ec4A | 3ec6A | 3ec9A | 3ecfA | 3ecyA | 3ed4A | 3edgA |
| 3edoA | 3ee4A | 3eeaA | 3eehA | 3eerA | 3eetA | 3ef8A | 3efgA | 3efyA | 3eg4A | 3eggC | 3ehgA | 3ehmA |
| 3eifA | 3eikA | 3ejgA | 3ejkA | 3ejvA | 3ejxA | 3ek3A | 3el6A | 3elbA | 3elkA | 3eloA | 3elqA | 3elsA |
| 3emfA | 3emiA | 3emrA | 3en0A | 3en8A | 3enbA | 3eo5A | 3eo6A | 3eo7A | 3eo8A | 3eofA | 3eoiA | 3eqnA |
| 3eqxA | 3er6A | 3er7A | 3erbA | 3es1A | 3es4A | 3eslA | 3essA | 3etnA | 3eu3A | 3euoA | 3eupA | 3evpA |
| 3ew1A | 3ewnA | 3exeA | 3exnA | 3exrA | 3ey7A | 3eyeA | 3eyiA | 3eziA | 3ezuA | 3ezwA | 3f0hA | 3f14A |
| 3f2eA | 3f2gA | 3f2iA | 3f2vA | 3f40A | 3f42A | 3f44A | 3f4aA | 3f4mA | 3f4rA | 3f5bA | 3f5rA | 3f62A |
| 3f62B | 3f67A | 3f6kA | 3f6oA | 3f6qA | 3f6qB | 3f6vA | 3f6wA | 3f75P | 3f7cA | 3f7eA | 3f7tA | 3f7wA |
| 3f7xA | 3f8kA | 3f8lA | 3f8mA | 3f8xA | 3f95A | 3f9sA | 3fajA | 3fanA | 3fauA | 3fb9A | 3fblA | 3fbuA |
| 3fc3A | 3fcnA | 3fd3A | 3fd5A | 3fdbA | 3fdhA | 3fdjA | 3fdqA | 3feuA | 3ff0A | 3ff2A | 3ff5A | 3ffrA |
| 3ffvA | 3ffyA | 3fg7A | 3fg8A | 3fg9A | 3fgbA | 3fgeA | 3fgrA | 3fgrB | 3fgvA | 3fgyA | 3fh1A | 3fhdA |
| 3fidA | 3firA | 3fj1A | 3fjsA | 3fjuB | 3fjvA | 3fk8A | 3fkaA | 3fkeA | 3flaA | 3fljA | 3fm0A | 3fm2A |
| 3fm5A | 3fmbA | 3fmyA | 3fn5A | 3fncA | 3fnkA | 3fo8D | 3fobA | 3fotA | 3fovA | 3fp2A | 3fp5A | 3fpeA |
| 3fpnB | 3fpqA | 3fpzA | 3fqgA | 3fr7A | 3frqA | 3frrA | 3fryA | 3fs2A | 3fs5A | 3fs8A | 3fsdA | 3fseA |
| 3fsgA | 3ft1A | 3ftbA | 3fuyA | 3fvbA | 3fvyA | 3fwkA | 3fwyA | 3fwzA | 3fx7A | 3fxaA | 3fxhA | 3fxqA |
| 3fxyA | 3fy3A | 3fymA | 3fynA | 3fz4A | 3fzeA | 3fzgA | 3fznA | 3fzyA | 3g02A | 3g0kA | 3g0tA | 3g0vA |
| 3g13A | 3g14A | 3g16A | 3g1jA | 3g1pA | 3g20A | 3g23A | 3g2bA | 3g2eA | 3g36A | 3g3sA | 3g40A | 3g4eA |
| 3g5jA | 3g5oA | 3g5oB | 3g5tA | 3g7nA | 3g7pA | 3g7rA | 3g7uA | 3g85A | 3g88A | 3g8zA | 3g98A | 3g9kD |
| 3ga7A | 3ga8A | 3gaeA | 3gasA | 3gb5A | 3gbgA | 3gbhA | 3gbwA | 3gbyA | 3gceA | 3gd0A | 3gdbA | 3gdcA |
| 3gdhA | 3gdwA | 3ge4A | 3gf3A | 3gf6A | 3gfpA | 3gfvA | 3gg4A | 3gg7A | 3ggqA | 3ggyA | 3ghdA | 3ghjA |
| 3gi7A | 3giwA | 3gjyA | 3gk6A | 3gkhA | 3gkkA | 3gkrA | 3glvA | 3gm5A | 3gmfA | 3gmgA | 3gmiA | 3gmsA |
| 3gn6A | 3gnlA | 3go5A | 3go9A | 3goeA | 3gohA | 3gp4A | 3gpiA | 3gpkA | 3gqqA | 3gr3A | 3greA | 3grhA |
| 3grrA | 3grzA | 3gs9A | 3gt2A | 3guxA | 3guzA | 3gv1A | 3gveA | 3gvjA | 3gwaA | 3gwbA | 3gwhA | 3gwiA |
| 3gwnA | 3gwqA | 3gwzA | 3gx8A | 3gxgA | 3gxwA | 3gy9A | 3gycA | 3gydA | 3gyeA | 3gykA | 3gzbA | 3gzrA |
| 3h04A | 3h05A | 3h0nA | 3h0uA | 3h1dA | 3h1nA | 3h1zA | 3h20A | 3h2bA | 3h2gA | 3h2zA | 3h31A | 3h36A |
| 3h3hA | 3h41A | 3h49A | 3h4wA | 3h4yA | 3h50A | 3h51A | 3h5lA | 3h6pA | 3h6qA | 3h6rA | 3h6xA | 3h70A |
| 3h75A | 3h79A | 3h7aA | 3h7hA | 3h7hB | 3h7jA | 3h87A | 3h87C | 3h8hA | 3h8oA | 3h8tA | 3h8uA | 3h95A |
| 3h97A | 3h9mA | 3h9wA | 3ha1A | 3ha9A | 3hbmA | 3hc1A | 3hczA | 3hd4A | 3hdeA | 3hdfA | 3hdjA | 3hdsA |
| 3hdxA | 3hftA | 3hfwA | 3hgbA | 3hguA | 3hh1A | 3hhiA | 3hhsA | 3hi2B | 3hieA | 3hj1A | 3hj9A | 3hjrA |
| 3hkmA | 3hkxA | 3hl1A | 3hlzA | 3hmcA | 3hmzA | 3hn0A | 3hn5A | 3hn7A | 3ho6A | 3ho7A | 3hoiA | 3hp7A |
| 3hqxA | 3hr0A | 3hr6A | 3hraA | 3hrgA | 3hrlA | 3hrpA | 3hrqA | 3hs8A | 3hsaA | 3ht1A | 3htnA | 3htsB |
| 3htvA | 3htyA | 3hutA | 3hv8A | 3hvwA | 3hwpA | 3hwuA | 3hx3A | 3hx8A | 3hx9A | 3hxjA | 3hxsA | 3hy7A |
| 3hyfA | 3hynA | 3hz6A | 3hzpA | 3i0yA | 3i10A | 3i24A | 3i26A | 3i2vA | 3i31A | 3i36A | 3i3fA | 3i45A |
| 3i4gA | 3i4oA | 3i5xA | 3i6cA | 3i76A | 3i7mA | 3i7tA | 3i7uA | 3i84A | 3i8bA | 3i8sA | 3ianA | 3iarA |
| 3iayA | 3ib5A | 3ib7A | 3ic3A | 3ic4A | 3icvA | 3id4A | 3id7A | 3idfA | 3iduA | 3idvA | 3idwA | 3ie4A |
| 3ieeA | 3ieiA | 3ievA | 3ifeA | 3ig9A | 3igfA | 3igrA | 3ihsA | 3ihtA | 3ihuA | 3ihvA | 3ii7A | 3iibA |
| 3iijA | 3ij3A | 3ij5A | 3ijmA | 3ikbA | 3ilfA | 3ilsA | 3imkA | 3imnA | 3imoA | 3iosA | 3ioxA | 3ioyA |
| 3ip5A | 3ipjA | 3ipwA | 3iq1A | 3iq2A | 3iqtA | 3ir3A | 3ir4A | 3irbA | 3irsA | 3irvA | 3isaA | 3isrA |
| 3isuA | 3it0A | 3it4A | 3iteA | 3itfA | 3itqA | 3iu0A | 3iu5A | 3iu6A | 3iugA | 3iukA | 3iuoA | 3iupA |
| 3iuwA | 3iuzA | 3iv0A | 3iv4A | 3ivdA | 3iwfA | 3iwtA | 3ixcA | 3ixrA | 3jq1A | 3jquA | 3jqwA | 3jqyA |
| 3jr7A | 3jrnA | 3jrrA | 3js6A | 3jsrA | 3jsyA | 3jtwA | 3jtxA | 3ju0A | 3ju2A | 3ju3A | 3jugA | 3jveA |
| 3jwgA | 3jx9A | 3jxoA | 3jxsA | 3jygA | 3jysA | 3jyzA | 3jz9A | 3jzeA | 3jzyA | 3k01A | 3k0bA | 3k0zA |
| 3k11A | 3k13A | 3k1jA | 3k1tA | 3k1uA | 3k1zA | 3k2aA | 3k2oA | 3k3cA | 3k4iA | 3k50A | 3k5jA | 3k65A |
| 3k69A | 3k6gA | 3k6oA | 3k6qA | 3k6uA | 3k6yA | 3k7gB | 3k7mX | 3k7xA | 3k86A | 3k8uA | 3k9jA | 3k9wA |
| 3ka7A | 3kb9A | 3kbgA | 3kbqA | 3kbrA | 3kbyA | 3kccA | 3kciA | 3kcpA | 3kcwA | 3kczA | 3kd2A | 3kd3A |
| 3kd4A | 3kd6A | 3kdeC | 3kdfB | 3kdwA | 3ke7A | 3kebA | 3kepA | 3kf6A | 3kf6B | 3kflA | 3kfoA | 3kg0A |
| 3kg4A | 3kg9A | 3kgdA | 3kgkA | 3kgrA | 3kgyA | 3kgzA | 3kh1A | 3kh7A | 3kh8A | 3kizA | 3kjoA | 3kk4A |
| 3kkbA | 3kkfA | 3kkgA | 3kkwA | 3klbA | 3klqA | 3km5A | 3kmhA | 3kmiA | 3kmuA | 3kmvA | 3knvA | 3kogA |
| 3kojA | 3kolA | 3kopA | 3korA | 3kosA | 3kp8A | 3kpcA | 3kpxA | 3kq0A | 3kq5A | 3kqiA | 3kraB | 3kreA |
| 3ksmA | 3kstA | 3kt9A | 3ktaB | 3ktcA | 3ktoA | 3kv0A | 3kv1A | 3kvhA | 3kvtA | 3kwcA | 3kwkA | 3kwlA |
| 3kwrA | 3kwsA | 3kxwA | 3kyaA | 3kyzA | 3kz3A | 3kz7A | 3kzdA | 3kzpA | 3kzvA | 3kzxA | 3l12A | 3l15A |
| 3l18A | 3l1eA | 3l1nA | 3l1wA | 3l2cA | 3l2hA | 3l2iA | 3l39A | 3l3bA | 3l3fX | 3l40A | 3l46A | 3l4eA |
| 3l4hA | 3l4nA | 3l4rA | 3l51A | 3l51B | 3l5aA | 3l5iA | 3l5kA | 3l6bA | 3l7xA | 3l7yA | 3l80A | 3l8aA |
| 3l8dA | 3l8eA | 3l8hA | 3l8qA | 3l8uA | 3l9aX | 3l9bA | 3l9fA | 3l9qA | 3l9sA | 3l9uA | 3laeA | 3lagA |
| 3latA | 3laxA | 3lazA | 3lccA | 3lcmA | 3lcrA | 3ld3A | 3ldcA | 3ldyA | 3le0A | 3le4A | 3ledA | 3leqA |
| 3letA | 3lewA | 3lf5A | 3lfhA | 3lfjA | 3lfkA | 3lfpA | 3lfrA | 3lgdA | 3lgfA | 3lhnA | 3lhoA | 3lhqA |
| 3li8A | 3lidA | 3lizA | 3ljqA | 3lk5A | 3lk7A | 3lkeA | 3lkmA | 3ll7A | 3llbA | 3llcA | 3lloA | 3lluA |
| 3llvA | 3lm2A | 3lm3A | 3lm4A | 3lmaA | 3lmdA | 3lmzA | 3lncA | 3lopA | 3lp5A | 3lpwA | 3lpzA | 3lq0A |
| 3lq9A | 3lqbA | 3lqhA | 3lqyA | 3lrkA | 3ls0A | 3ltiA | 3ltjA | 3lufA | 3lulA | 3lurA | 3luuA | 3luyA |
| 3lv8A | 3lw8E | 3lw9A | 3lwaA | 3lwcA | 3lwkA | 3lwtX | 3lwxA | 3lx4A | 3lxqA | 3lxtA | 3lxyA | 3ly0A |
| 3ly1A | 3ly7A | 3lydA | 3lygA | 3lyhA | 3lyyA | 3lzaA | 3lzkA | 3m07A | 3m1dA | 3m1eA | 3m1iB | 3m1tA |
| 3m1uA | 3m3pA | 3m4rA | 3m5bA | 3m5lA | 3m6jA | 3m6uA | 3m6yA | 3m70A | 3m71A | 3m7aA | 3m7fB | 3m7oA |
| 3m7vA | 3m84A | 3m86A | 3m8jA | 3m8uA | 3m97X | 3m9zA | 3malA | 3maoA | 3mazA | 3mb8A | 3mbgA | 3mbhA |
| 3mbiA | 3mbrX | 3mc3A | 3mcbB | 3mcqA | 3mcwA | 3mcxA | 3mczA | 3md7A | 3mdpA | 3mdqA | 3me7A | 3me9A |
| 3mebA | 3mewA | 3mfdA | 3mg1A | 3mgdA | 3mggA | 3mgkA | 3mhxA | 3milA | 3mkoA | 3ml1B | 3ml3A | 3mm4A |
| 3mmyA | 3mmyB | 3mn2A | 3mnmA | 3mo4A | 3mosA | 3mpcA | 3mprA | 3mq0A | 3mq2A | 3mqgA | 3mqoA | 3mqzA |
| 3mr0A | 3mr2A | 3mstA | 3mswA | 3msxB | 3mt0A | 3mtqA | 3mtrA | 3mujA | 3muz1 | 3mvcA | 3mvgA | 3mvnA |
| 3mvuA | 3mwbA | 3mwqA | 3mx3A | 3mx7A | 3mxnA | 3mxnB | 3mxzA | 3myxA | 3mz1A | 3mz2A | 3mzoA | 3n07A |
| 3n0kA | 3n0rA | 3n0uA | 3n0wA | 3n0xA | 3n11A | 3n1sA | 3n29A | 3n3uA | 3n4fA | 3n4jA | 3n6wA | 3n6yA |
| 3n6zA | 3n72A | 3n77A | 3n8bA | 3n8uA | 3n9uC | 3na5A | 3nbmA | 3nd1A | 3ndhA | 3ndiA | 3ndqA | 3ne4A |
| 3ne8A | 3nehA | 3neuA | 3nfvA | 3nfwA | 3ng2A | 3ngfA | 3nghA | 3nh6A | 3ni0A | 3njcA | 3njeA | 3njhA |
| 3nkeA | 3nkgA | 3nklA | 3nl9A | 3no0A | 3no2A | 3no3A | 3no4A | 3no6A | 3no7A | 3nohA | 3noiA | 3nojA |
| 3nokA | 3nonA | 3npdA | 3nqiA | 3nqnA | 3nqpA | 3nr5A | 3nr6A | 3nreA | 3nrfA | 3nrhA | 3nrpA | 3nrvA |
| 3nrwA | 3ns6A | 3nswA | 3nufA | 3nuiA | 3nuqA | 3nurA | 3nv0A | 3nv0B | 3nvtA | 3nvvB | 3nwcA | 3nwoA |
| 3nxlA | 3nxqA | 3ny7A | 3nycA | 3nyiA | 3nymA | 3nyyA | 3nzeA | 3nzkA | 3nznA | 3nztA | 3o0dA | 3o0lA |
| 3o0yA | 3o10A | 3o12A | 3o14A | 3o1kA | 3o26A | 3o2rA | 3o2uA | 3o3fA | 3o3mA | 3o3mB | 3o3uN | 3o53A |
| 3o6cA | 3o6nA | 3o6pA | 3o70A | 3o7aA | 3o7bA | 3o7hA | 3o7mA | 3o8bA | 3o9oA | 3oa2A | 3oa3A | 3oa4A |
| 3oa8A | 3oa8B | 3oajA | 3obeA | 3oc2A | 3oc9A | 3ocjA | 3ocmA | 3ocrA | 3ocuA | 3od1A | 3od9A | 3odtA |
| 3oe3A | 3oepA | 3of4A | 3of5A | 3of7A | 3ofgA | 3og2A | 3og9A | 3ogaA | 3oghA | 3oh8A | 3ohgA | 3ohrA |
| 3oi8A | 3oioA | 3oirA | 3oisA | 3oizA | 3oj0A | 3oj6A | 3ojcA | 3okgA | 3okxA | 3ol3A | 3olqA | 3om0A |
| 3omcA | 3omdA | 3omsA | 3on1A | 3on2A | 3on4A | 3onjA | 3onpA | 3ooiA | 3oonA | 3oopA | 3oosA | 3oouA |
| 3ooxA | 3op6A | 3op7A | 3op9A | 3oq2A | 3oqgA | 3oqhA | 3oqpA | 3oqvA | 3or5A | 3orhA | 3oruA | 3oryA |
| 3os7A | 3osdA | 3oseA | 3osmA | 3ot1A | 3ot2A | 3otnA | 3ou2A | 3oufA | 3ougA | 3ouiA | 3ov5A | 3ovkA |
| 3owaA | 3owcA | 3owrA | 3owvA | 3oz2A | 3ozhA | 3p0kA | 3p1wA | 3p24A | 3p2eA | 3p2nA | 3p2qA | 3p3vA |
| 3p42A | 3p47A | 3p4gA | 3p4hA | 3p4lA | 3p53A | 3p6lA | 3p94A | 3p9vA | 3pa6A | 3pajA | 3pasA | 3pb4X |
| 3pc2A | 3pddA | 3pdtA | 3pesA | 3pf2A | 3pf6A | 3pfeA | 3pfgA | 3pfoA | 3pfyA | 3pg0A | 3pg6A | 3pgpA |
| 3phsA | 3pi7A | 3picA | 3pigA | 3piwA | 3pj0A | 3pjlA | 3pjvD | 3pjyA | 3pkvA | 3pkzA | 3pl0A | 3plwA |
| 3pm2A | 3pmdA | 3pmmA | 3pmsA | 3pmtA | 3pnnA | 3pnxA | 3pnzA | 3pohA | 3pp5A | 3pp9A | 3pplA | 3pr6A |
| 3ps0A | 3psaA | 3psxA | 3pt1A | 3pt5A | 3ptyA | 3pu9A | 3pubA | 3putA | 3pveA | 3pycA | 3pywA | 3pz9A |
| 3pzfA | 3pzjA | 3q0hA | 3q13A | 3q1cA | 3q1nA | 3q1pA | 3q20A | 3q2eA | 3q34A | 3q3qA | 3q47B | 3q4oA |
| 3q62A | 3q6sA | 3q7rA | 3q8tA | 3q93A | 3q9dA | 3q9vA | 3qa9A | 3qaoA | 3qayA | 3qb8A | 3qbcA | 3qbtB |
| 3qc7A | 3qdlA | 3qguA | 3qh4A | 3qhpA | 3qi7A | 3qitA | 3qleA | 3qliA | 3qljA | 3qooA | 3qp8A | 3qq8B |
| 3qqqA | 3qraA | 3qsjA | 3qslA | 3qsqA | 3qt5A | 3qtaA | 3qu3A | 3qufA | 3quvA | 3qvmA | 3qw3A | 3qw9A |
| 3qxbA | 3qxfA | 3qy3A | 3qy8A | 3qyfA | 3qyyA | 3qzsA | 3r0nA | 3r0vA | 3r0xA | 3r14A | 3r1kA | 3r1wA |
| 3r3uA | 3r5aA | 3r5gA | 3r5lA | 3r5yA | 3r6fA | 3r6hA | 3r6oA | 3r77A | 3r7aA | 3r89A | 3r8eA | 3r8yA |
| 3r90A | 3rayA | 3rbuA | 3rbvA | 3rd5A | 3rd7A | 3rdmA | 3rdyA | 3re2A | 3reeA | 3renA | 3rf0A | 3rf6A |
| 3rfeA | 3rgoA | 3rh0A | 3rhbA | 3rhgA | 3rhtA | 3rhzA | 3rivA | 3rjtA | 3rjvA | 3rk6A | 3rlbA | 3rleA |
| 3rlhA | 3rlsA | 3rmhA | 3rmqA | 3rnlA | 3rnrA | 3robA | 3rpcA | 3rpeA | 3rpfA | 3rpfC | 3rphA | 3rpnA |
| 3rpwA | 3rqiA | 3rqsA | 3rqzA | 3rrxA | 3ru6A | 3ruxA | 3rv1A | 3rwnA | 3rx9A | 3rxyA | 3rykA | 3rzsA |
| 3s25A | 3s3lA | 3s3tA | 3s3zA | 3s4mA | 3s5qA | 3s5vA | 3s5wA | 3s6bA | 3s6eA | 3s6fA | 3s8iA | 3s8mA |
| 3s8pA | 3s8sA | 3s9jA | 3s9xA | 3saeA | 3sb1A | 3sb4A | 3sd2A | 3sd4A | 3sdwA | 3se5A | 3seeA | 3sg0A |
| 3sggA | 3sh4A | 3shgA | 3shgB | 3shtA | 3sk9A | 3sl7A | 3slrA | 3smpA | 3snyA | 3so5A | 3soeA | 3sojA |
| 3solA | 3st1A | 3sxkA | 3sxwA | 3sxyA | 3szaA | 3szyA | 3t49A | 3t54A | 3t5nA | 3t6pA | 3t7vA | 3t95A |
| 3tedA | 3thcA | 3thkA | 3towA | 3tu8A | 3txsA | 3ty1A | 3u0gA | 3u0vA | 3u13A | 3u1dA | 3u1lA | 3u1wA |
| 3u26A | 3u28A | 3u28C | 3u4vA | 3u7iA | 3u7vA | 3u7zA | 3u8vA | 3u9rB | 3uamA | 3ubwA | 3ucjA | 3ucqA |
| 3udfA | 3ugfA | 3uh9A | 3uj6A | 3uksA | 3umvA | 3uo3A | 3uotA | 3upiA | 3upsA | 3urrA | 3us6A | 3utmA |
| 3uv0A | 3uv2A | 3uw3A | 3uzuA | 3v2iA | 3v4cA | 3v4gA | 3v5xA | 3v6tA | 3v7bA | 3v7pA | 3v9oA | 3vaaA |
| 3vbiA | 3vcxA | 3vdiA | 3venA | 3vgzA | 3vi6A | 3vk6A | 3vluA | 3zqkA | 3zqmA | 3zqsA | 3zquA | 3zr8X |
| 3zrgA | 3zriA | 3zrvA | 3ztvA | 3zvlA | 3zvzB | 3zx1A | 3zzpA | 3zzyA | 4a4yA | 4a57A | 4a94C | 4a9eA |
| 4acoA | 4d8lA |  |  |  |  |  |  |  |  |  |  |  |

Table S2: Pearson correlation coefficient (PCC) of methods on different datasets. Methods are represented by rows and datasets are represented by columns respectively.

|  | PHI | | | PSI | | |
| --- | --- | --- | --- | --- | --- | --- |
| Dataset  Method | ANGLOR | SPINE X | ccPDB30 | ANGLOR | SPINE X | ccPDB30 |
| ANGLOR | 0.758 | 0.754 | 0.762 | 0.766 | 0.771 | 0.765 |
| TANGLE (as reported) | 0.529 | NA | NA | 0.654 | NA | NA |
| SPINE X | 0.791 | 0.799 | 0.800 | 0.811 | 0.819 | 0.804 |
| RANDOM | 0.639 | 0.603 | 0.615 | 0.614 | 0.591 | 0.584 |

Table S3: Root-mean-square-error (RMSE) of methods on different datasets. Methods are represented by rows and datasets are represented by columns respectively.

|  | PHI | | | PSI | | |
| --- | --- | --- | --- | --- | --- | --- |
| Dataset  Method | ANGLOR | SPINE X | ccPDB30 | ANGLOR | SPINE X | ccPDB30 |
| ANGLOR | 43.65 | 39.21 | 39.37 | 62.24 | 59.58 | 60.53 |
| TANGLE (as reported) | 46.72 | NA | NA | 69.45 | NA | NA |
| SPINE X | 40.98 | 35.93 | 36.62 | 65.93 | 61.45 | 64.73 |
| RANDOM | 61.10 | 56.68 | 56.55 | 108.90 | 109.77 | 110.31 |

Table S4: Distribution of phi angle for 20 amino acids.

| Phi Angle | A | C | D | E | F | G | H | I | K | L | M | N | P | Q | R | S | T | V | W | Y |
| --- | --- | --- | --- | --- | --- | --- | --- | --- | --- | --- | --- | --- | --- | --- | --- | --- | --- | --- | --- | --- |
| -180 | 0 | 0 | 0 | 0 | 1 | 1 | 1 | 0 | 0 | 0 | 0 | 0 | 0 | 0 | 1 | 0 | 0 | 0 | 0 | 0 |
| -179 | 7 | 3 | 4 | 0 | 1 | 50 | 1 | 1 | 3 | 0 | 0 | 4 | 0 | 1 | 7 | 7 | 5 | 3 | 1 | 2 |
| -178 | 12 | 2 | 2 | 3 | 3 | 73 | 0 | 0 | 4 | 2 | 0 | 2 | 0 | 2 | 6 | 7 | 3 | 3 | 0 | 3 |
| -177 | 6 | 3 | 10 | 3 | 6 | 61 | 4 | 1 | 3 | 2 | 0 | 3 | 0 | 0 | 5 | 11 | 4 | 1 | 1 | 2 |
| -176 | 7 | 1 | 7 | 4 | 13 | 70 | 3 | 1 | 5 | 1 | 5 | 6 | 0 | 3 | 7 | 18 | 4 | 1 | 2 | 6 |
| -175 | 23 | 2 | 13 | 7 | 4 | 58 | 4 | 2 | 3 | 4 | 1 | 7 | 1 | 4 | 8 | 15 | 4 | 1 | 3 | 7 |
| -174 | 14 | 3 | 10 | 7 | 4 | 64 | 4 | 1 | 6 | 3 | 2 | 4 | 0 | 0 | 8 | 17 | 3 | 1 | 1 | 11 |
| -173 | 18 | 3 | 15 | 7 | 8 | 53 | 8 | 0 | 7 | 2 | 2 | 9 | 0 | 4 | 7 | 32 | 5 | 3 | 3 | 4 |
| -172 | 29 | 3 | 12 | 5 | 11 | 75 | 9 | 5 | 8 | 3 | 3 | 8 | 0 | 4 | 10 | 31 | 2 | 1 | 2 | 4 |
| -171 | 24 | 7 | 17 | 14 | 17 | 75 | 9 | 5 | 7 | 4 | 4 | 12 | 0 | 11 | 17 | 34 | 10 | 0 | 0 | 14 |
| -170 | 24 | 10 | 14 | 5 | 10 | 68 | 13 | 0 | 14 | 5 | 11 | 9 | 0 | 5 | 17 | 39 | 11 | 2 | 2 | 13 |
| -169 | 59 | 17 | 25 | 12 | 16 | 60 | 8 | 2 | 20 | 10 | 6 | 20 | 0 | 10 | 17 | 32 | 7 | 3 | 7 | 10 |
| -168 | 43 | 9 | 27 | 13 | 23 | 59 | 18 | 3 | 14 | 7 | 5 | 20 | 0 | 10 | 14 | 60 | 16 | 3 | 7 | 18 |
| -167 | 43 | 9 | 27 | 16 | 24 | 65 | 14 | 4 | 18 | 8 | 6 | 15 | 0 | 15 | 23 | 70 | 17 | 5 | 9 | 16 |
| -166 | 54 | 16 | 36 | 15 | 19 | 60 | 18 | 6 | 21 | 11 | 8 | 21 | 0 | 12 | 23 | 77 | 13 | 8 | 9 | 17 |
| -165 | 70 | 18 | 33 | 15 | 33 | 63 | 24 | 0 | 19 | 10 | 11 | 33 | 0 | 19 | 26 | 60 | 17 | 5 | 8 | 26 |
| -164 | 79 | 17 | 30 | 25 | 33 | 69 | 23 | 8 | 31 | 16 | 11 | 22 | 0 | 18 | 24 | 101 | 27 | 4 | 11 | 41 |
| -163 | 93 | 20 | 41 | 33 | 34 | 59 | 26 | 8 | 29 | 20 | 13 | 33 | 0 | 19 | 28 | 85 | 30 | 11 | 13 | 16 |
| -162 | 96 | 29 | 54 | 24 | 48 | 65 | 21 | 7 | 31 | 27 | 11 | 40 | 0 | 18 | 32 | 115 | 21 | 9 | 13 | 35 |
| -161 | 99 | 27 | 54 | 21 | 59 | 69 | 26 | 3 | 22 | 26 | 14 | 38 | 0 | 22 | 37 | 109 | 40 | 16 | 18 | 46 |
| -160 | 109 | 21 | 49 | 34 | 48 | 56 | 33 | 11 | 34 | 29 | 14 | 36 | 0 | 21 | 37 | 105 | 42 | 10 | 20 | 38 |
| -159 | 97 | 27 | 56 | 41 | 48 | 42 | 25 | 13 | 35 | 34 | 10 | 43 | 0 | 27 | 51 | 115 | 36 | 23 | 18 | 56 |
| -158 | 103 | 27 | 60 | 41 | 69 | 55 | 32 | 21 | 35 | 41 | 20 | 48 | 0 | 21 | 52 | 133 | 32 | 28 | 24 | 47 |
| -157 | 124 | 31 | 63 | 34 | 72 | 57 | 43 | 12 | 41 | 30 | 16 | 48 | 0 | 25 | 42 | 125 | 39 | 17 | 28 | 49 |
| -156 | 109 | 26 | 53 | 43 | 66 | 62 | 36 | 31 | 38 | 30 | 22 | 46 | 1 | 36 | 52 | 128 | 57 | 21 | 20 | 63 |
| -155 | 135 | 27 | 54 | 50 | 74 | 54 | 44 | 33 | 54 | 41 | 24 | 66 | 0 | 42 | 55 | 127 | 51 | 32 | 30 | 61 |
| -154 | 122 | 41 | 57 | 50 | 88 | 56 | 35 | 29 | 49 | 32 | 21 | 46 | 1 | 36 | 48 | 128 | 65 | 32 | 24 | 66 |
| -153 | 135 | 24 | 53 | 41 | 73 | 56 | 39 | 18 | 44 | 50 | 23 | 57 | 0 | 34 | 57 | 136 | 64 | 38 | 22 | 67 |
| -152 | 140 | 31 | 73 | 50 | 85 | 61 | 41 | 20 | 44 | 48 | 19 | 63 | 0 | 31 | 71 | 132 | 62 | 39 | 21 | 69 |
| -151 | 131 | 28 | 67 | 52 | 82 | 57 | 52 | 23 | 56 | 47 | 20 | 53 | 0 | 46 | 64 | 116 | 63 | 50 | 35 | 78 |
| -150 | 140 | 35 | 65 | 81 | 79 | 45 | 44 | 28 | 47 | 54 | 29 | 50 | 1 | 36 | 69 | 122 | 87 | 53 | 22 | 76 |
| -149 | 132 | 19 | 64 | 66 | 84 | 50 | 45 | 43 | 66 | 57 | 22 | 68 | 0 | 47 | 65 | 127 | 72 | 57 | 22 | 83 |
| -148 | 134 | 37 | 65 | 90 | 95 | 52 | 54 | 33 | 59 | 53 | 23 | 49 | 0 | 52 | 54 | 133 | 62 | 61 | 27 | 70 |
| -147 | 166 | 26 | 69 | 86 | 87 | 47 | 49 | 35 | 70 | 69 | 27 | 51 | 1 | 68 | 87 | 134 | 76 | 60 | 24 | 72 |
| -146 | 135 | 24 | 74 | 74 | 72 | 45 | 47 | 50 | 87 | 82 | 38 | 61 | 0 | 46 | 72 | 154 | 81 | 89 | 21 | 70 |
| -145 | 132 | 28 | 65 | 91 | 82 | 62 | 38 | 60 | 83 | 63 | 27 | 65 | 0 | 53 | 81 | 136 | 104 | 87 | 21 | 59 |
| -144 | 127 | 21 | 69 | 94 | 85 | 51 | 52 | 61 | 80 | 82 | 31 | 62 | 0 | 59 | 79 | 155 | 114 | 113 | 22 | 91 |
| -143 | 135 | 25 | 70 | 93 | 71 | 51 | 55 | 86 | 66 | 83 | 29 | 78 | 0 | 53 | 111 | 136 | 121 | 146 | 39 | 68 |
| -142 | 124 | 32 | 76 | 92 | 84 | 58 | 65 | 75 | 96 | 93 | 29 | 54 | 0 | 71 | 110 | 146 | 150 | 175 | 25 | 91 |
| -141 | 147 | 36 | 72 | 100 | 86 | 55 | 53 | 90 | 101 | 87 | 37 | 73 | 0 | 51 | 93 | 153 | 143 | 153 | 25 | 95 |
| -140 | 125 | 38 | 85 | 95 | 96 | 54 | 51 | 94 | 92 | 85 | 37 | 68 | 0 | 65 | 116 | 145 | 157 | 178 | 30 | 87 |
| -139 | 107 | 37 | 74 | 97 | 109 | 58 | 60 | 127 | 116 | 84 | 44 | 87 | 0 | 59 | 118 | 143 | 173 | 212 | 24 | 97 |
| -138 | 115 | 33 | 71 | 121 | 106 | 54 | 57 | 140 | 104 | 83 | 43 | 83 | 0 | 67 | 108 | 152 | 192 | 247 | 29 | 107 |
| -137 | 100 | 34 | 73 | 118 | 87 | 58 | 70 | 169 | 99 | 123 | 36 | 81 | 0 | 70 | 108 | 141 | 208 | 303 | 30 | 112 |
| -136 | 114 | 41 | 77 | 119 | 126 | 45 | 87 | 142 | 109 | 101 | 43 | 74 | 0 | 69 | 145 | 150 | 217 | 305 | 32 | 113 |
| -135 | 125 | 42 | 71 | 96 | 124 | 55 | 71 | 192 | 118 | 153 | 49 | 93 | 0 | 92 | 118 | 144 | 222 | 373 | 30 | 129 |
| -134 | 102 | 49 | 67 | 126 | 127 | 53 | 62 | 217 | 118 | 151 | 42 | 84 | 2 | 79 | 129 | 150 | 224 | 358 | 32 | 123 |
| -133 | 134 | 33 | 82 | 123 | 145 | 74 | 76 | 200 | 150 | 171 | 39 | 92 | 0 | 86 | 129 | 134 | 265 | 402 | 28 | 145 |
| -132 | 113 | 39 | 73 | 126 | 130 | 49 | 86 | 246 | 123 | 157 | 46 | 93 | 0 | 96 | 132 | 135 | 276 | 420 | 29 | 140 |
| -131 | 104 | 45 | 82 | 135 | 144 | 61 | 85 | 257 | 124 | 166 | 45 | 92 | 1 | 72 | 144 | 145 | 275 | 425 | 36 | 121 |
| -130 | 113 | 52 | 75 | 108 | 148 | 64 | 81 | 234 | 129 | 167 | 51 | 94 | 0 | 82 | 133 | 139 | 289 | 457 | 37 | 114 |
| -129 | 103 | 48 | 85 | 144 | 165 | 64 | 60 | 273 | 145 | 192 | 41 | 96 | 1 | 71 | 143 | 156 | 287 | 452 | 55 | 131 |
| -128 | 114 | 41 | 98 | 115 | 169 | 64 | 69 | 291 | 131 | 211 | 45 | 92 | 0 | 84 | 140 | 161 | 289 | 454 | 40 | 133 |
| -127 | 104 | 56 | 105 | 135 | 199 | 65 | 81 | 302 | 149 | 207 | 51 | 91 | 0 | 82 | 147 | 114 | 289 | 469 | 54 | 135 |
| -126 | 121 | 38 | 81 | 149 | 168 | 57 | 76 | 313 | 136 | 233 | 55 | 95 | 0 | 89 | 157 | 126 | 279 | 481 | 37 | 152 |
| -125 | 110 | 40 | 96 | 129 | 157 | 55 | 97 | 338 | 135 | 233 | 48 | 95 | 2 | 83 | 149 | 142 | 314 | 456 | 39 | 163 |
| -124 | 98 | 55 | 104 | 139 | 182 | 84 | 78 | 331 | 137 | 226 | 48 | 114 | 1 | 83 | 137 | 125 | 314 | 458 | 43 | 154 |
| -123 | 101 | 53 | 102 | 139 | 159 | 60 | 85 | 342 | 144 | 265 | 44 | 112 | 1 | 88 | 147 | 142 | 295 | 475 | 44 | 154 |
| -122 | 110 | 55 | 96 | 137 | 164 | 53 | 74 | 335 | 144 | 259 | 44 | 112 | 2 | 98 | 146 | 118 | 314 | 479 | 35 | 132 |
| -121 | 114 | 59 | 99 | 129 | 180 | 63 | 65 | 334 | 140 | 259 | 46 | 114 | 1 | 85 | 112 | 138 | 294 | 464 | 44 | 153 |
| -120 | 117 | 65 | 99 | 133 | 187 | 74 | 93 | 339 | 156 | 269 | 44 | 108 | 1 | 75 | 151 | 124 | 268 | 460 | 34 | 150 |
| -119 | 90 | 42 | 106 | 124 | 170 | 66 | 80 | 314 | 152 | 331 | 50 | 101 | 0 | 105 | 123 | 156 | 290 | 466 | 55 | 134 |
| -118 | 121 | 63 | 85 | 133 | 182 | 65 | 74 | 330 | 127 | 259 | 51 | 117 | 1 | 72 | 123 | 136 | 271 | 455 | 50 | 153 |
| -117 | 111 | 42 | 114 | 131 | 177 | 70 | 72 | 341 | 149 | 302 | 41 | 124 | 0 | 93 | 116 | 123 | 296 | 441 | 54 | 149 |
| -116 | 111 | 49 | 118 | 143 | 181 | 75 | 82 | 366 | 154 | 318 | 46 | 133 | 1 | 84 | 126 | 126 | 260 | 403 | 43 | 142 |
| -115 | 118 | 61 | 131 | 141 | 180 | 59 | 81 | 316 | 146 | 290 | 44 | 133 | 2 | 92 | 129 | 150 | 249 | 410 | 50 | 127 |
| -114 | 97 | 53 | 121 | 112 | 173 | 58 | 85 | 332 | 162 | 314 | 45 | 122 | 1 | 97 | 132 | 139 | 252 | 397 | 54 | 168 |
| -113 | 112 | 46 | 144 | 134 | 167 | 73 | 91 | 310 | 147 | 297 | 41 | 143 | 3 | 108 | 130 | 112 | 244 | 426 | 40 | 175 |
| -112 | 115 | 66 | 121 | 129 | 165 | 73 | 91 | 294 | 167 | 357 | 38 | 132 | 3 | 84 | 120 | 118 | 274 | 366 | 39 | 152 |
| -111 | 116 | 52 | 126 | 139 | 197 | 69 | 94 | 281 | 152 | 334 | 40 | 101 | 1 | 94 | 123 | 126 | 269 | 350 | 41 | 135 |
| -110 | 115 | 47 | 158 | 151 | 161 | 72 | 89 | 309 | 142 | 353 | 53 | 128 | 3 | 93 | 130 | 124 | 229 | 349 | 47 | 175 |
| -109 | 102 | 65 | 164 | 133 | 190 | 85 | 82 | 310 | 171 | 341 | 62 | 137 | 1 | 109 | 136 | 118 | 224 | 318 | 46 | 120 |
| -108 | 115 | 48 | 175 | 135 | 156 | 80 | 86 | 269 | 159 | 363 | 59 | 148 | 6 | 93 | 134 | 129 | 251 | 336 | 38 | 141 |
| -107 | 115 | 45 | 157 | 137 | 177 | 84 | 105 | 285 | 144 | 360 | 43 | 133 | 2 | 85 | 131 | 121 | 209 | 318 | 43 | 133 |
| -106 | 125 | 41 | 172 | 132 | 174 | 93 | 87 | 257 | 161 | 366 | 46 | 170 | 2 | 107 | 120 | 137 | 209 | 297 | 34 | 143 |
| -105 | 102 | 55 | 183 | 147 | 170 | 109 | 74 | 256 | 168 | 340 | 44 | 167 | 10 | 108 | 140 | 132 | 237 | 290 | 44 | 168 |
| -104 | 118 | 43 | 192 | 158 | 161 | 100 | 82 | 237 | 166 | 350 | 46 | 168 | 6 | 118 | 146 | 130 | 210 | 282 | 47 | 143 |
| -103 | 111 | 36 | 190 | 153 | 174 | 112 | 89 | 246 | 175 | 359 | 35 | 185 | 9 | 100 | 143 | 142 | 235 | 286 | 50 | 158 |
| -102 | 139 | 49 | 204 | 160 | 150 | 102 | 85 | 260 | 171 | 351 | 40 | 193 | 6 | 101 | 131 | 159 | 210 | 286 | 58 | 142 |
| -101 | 92 | 51 | 235 | 177 | 161 | 112 | 94 | 216 | 191 | 399 | 51 | 181 | 14 | 105 | 134 | 149 | 223 | 233 | 39 | 122 |
| -100 | 138 | 49 | 231 | 169 | 149 | 114 | 96 | 226 | 156 | 365 | 41 | 180 | 13 | 115 | 165 | 152 | 196 | 265 | 44 | 132 |
| -99 | 122 | 50 | 259 | 148 | 149 | 116 | 77 | 234 | 180 | 379 | 58 | 175 | 15 | 126 | 154 | 144 | 193 | 257 | 57 | 128 |
| -98 | 139 | 42 | 229 | 191 | 148 | 114 | 84 | 184 | 168 | 345 | 44 | 204 | 18 | 116 | 146 | 156 | 205 | 233 | 47 | 133 |
| -97 | 140 | 50 | 216 | 171 | 154 | 97 | 78 | 220 | 199 | 365 | 35 | 199 | 19 | 107 | 152 | 192 | 209 | 233 | 41 | 138 |
| -96 | 178 | 34 | 264 | 176 | 138 | 133 | 79 | 205 | 200 | 369 | 55 | 189 | 31 | 135 | 156 | 197 | 222 | 199 | 41 | 148 |
| -95 | 155 | 54 | 278 | 184 | 140 | 119 | 90 | 173 | 210 | 356 | 59 | 194 | 30 | 112 | 130 | 184 | 213 | 217 | 48 | 122 |
| -94 | 165 | 37 | 278 | 178 | 132 | 134 | 77 | 215 | 189 | 387 | 49 | 200 | 25 | 124 | 131 | 185 | 213 | 231 | 45 | 108 |
| -93 | 170 | 37 | 267 | 175 | 132 | 120 | 93 | 198 | 193 | 373 | 58 | 219 | 36 | 129 | 133 | 194 | 221 | 244 | 51 | 101 |
| -92 | 158 | 59 | 295 | 192 | 146 | 137 | 85 | 182 | 185 | 356 | 51 | 191 | 53 | 121 | 168 | 191 | 187 | 210 | 39 | 102 |
| -91 | 201 | 45 | 285 | 217 | 135 | 149 | 92 | 203 | 209 | 372 | 68 | 214 | 63 | 151 | 140 | 191 | 208 | 203 | 53 | 120 |
| -90 | 187 | 43 | 317 | 201 | 155 | 150 | 69 | 198 | 213 | 370 | 49 | 226 | 70 | 119 | 169 | 192 | 208 | 181 | 48 | 130 |
| -89 | 216 | 51 | 346 | 234 | 153 | 150 | 99 | 207 | 191 | 338 | 57 | 228 | 81 | 136 | 152 | 232 | 211 | 216 | 53 | 106 |
| -88 | 194 | 56 | 310 | 236 | 125 | 153 | 67 | 165 | 240 | 368 | 67 | 202 | 89 | 127 | 161 | 239 | 200 | 194 | 55 | 100 |
| -87 | 215 | 46 | 351 | 223 | 130 | 136 | 69 | 180 | 260 | 362 | 54 | 206 | 110 | 154 | 157 | 222 | 212 | 199 | 58 | 95 |
| -86 | 243 | 55 | 357 | 245 | 118 | 140 | 91 | 179 | 218 | 376 | 56 | 209 | 132 | 146 | 173 | 215 | 207 | 208 | 48 | 111 |
| -85 | 227 | 52 | 313 | 257 | 142 | 158 | 78 | 182 | 252 | 349 | 70 | 206 | 151 | 130 | 160 | 229 | 205 | 200 | 51 | 107 |
| -84 | 251 | 45 | 384 | 250 | 117 | 159 | 87 | 169 | 249 | 389 | 65 | 225 | 154 | 152 | 195 | 264 | 226 | 197 | 49 | 115 |
| -83 | 231 | 47 | 353 | 237 | 140 | 156 | 92 | 170 | 215 | 378 | 69 | 219 | 177 | 127 | 177 | 273 | 214 | 195 | 47 | 93 |
| -82 | 249 | 45 | 362 | 273 | 123 | 162 | 81 | 180 | 225 | 380 | 66 | 185 | 196 | 139 | 193 | 260 | 221 | 192 | 48 | 106 |
| -81 | 264 | 61 | 315 | 269 | 146 | 145 | 73 | 183 | 261 | 390 | 76 | 196 | 237 | 185 | 176 | 269 | 232 | 237 | 60 | 101 |
| -80 | 315 | 48 | 347 | 269 | 130 | 159 | 77 | 179 | 229 | 387 | 71 | 193 | 255 | 163 | 198 | 280 | 237 | 202 | 52 | 98 |
| -79 | 328 | 51 | 324 | 291 | 157 | 166 | 95 | 178 | 255 | 369 | 72 | 199 | 314 | 160 | 180 | 289 | 224 | 214 | 51 | 121 |
| -78 | 311 | 50 | 331 | 302 | 141 | 169 | 80 | 198 | 265 | 395 | 55 | 179 | 325 | 163 | 233 | 324 | 221 | 204 | 64 | 129 |
| -77 | 334 | 49 | 320 | 304 | 120 | 179 | 85 | 195 | 245 | 445 | 64 | 188 | 363 | 153 | 235 | 300 | 260 | 213 | 68 | 119 |
| -76 | 374 | 51 | 349 | 304 | 142 | 186 | 113 | 219 | 248 | 426 | 71 | 219 | 382 | 181 | 238 | 316 | 244 | 263 | 61 | 123 |
| -75 | 422 | 63 | 312 | 379 | 170 | 170 | 87 | 219 | 285 | 495 | 84 | 201 | 388 | 199 | 240 | 372 | 242 | 289 | 62 | 135 |
| -74 | 442 | 49 | 325 | 399 | 175 | 195 | 127 | 257 | 283 | 500 | 102 | 227 | 429 | 223 | 267 | 349 | 256 | 308 | 67 | 138 |
| -73 | 479 | 84 | 363 | 457 | 176 | 188 | 126 | 228 | 351 | 552 | 104 | 222 | 452 | 231 | 284 | 414 | 276 | 306 | 82 | 151 |
| -72 | 480 | 75 | 382 | 493 | 222 | 198 | 108 | 289 | 342 | 621 | 135 | 239 | 476 | 261 | 328 | 456 | 279 | 374 | 91 | 160 |
| -71 | 571 | 79 | 427 | 561 | 242 | 220 | 173 | 338 | 462 | 686 | 140 | 272 | 517 | 306 | 324 | 443 | 284 | 430 | 102 | 178 |
| -70 | 664 | 88 | 464 | 703 | 211 | 252 | 168 | 392 | 513 | 807 | 150 | 306 | 509 | 328 | 412 | 503 | 375 | 443 | 100 | 197 |
| -69 | 801 | 94 | 518 | 809 | 278 | 279 | 157 | 440 | 529 | 1002 | 176 | 306 | 552 | 385 | 445 | 557 | 417 | 532 | 109 | 228 |
| -68 | 956 | 122 | 543 | 920 | 297 | 342 | 195 | 550 | 623 | 1108 | 220 | 331 | 589 | 460 | 537 | 571 | 427 | 584 | 135 | 267 |
| -67 | 1135 | 114 | 593 | 1084 | 348 | 347 | 224 | 604 | 730 | 1317 | 244 | 362 | 589 | 523 | 652 | 680 | 477 | 692 | 127 | 293 |
| -66 | 1373 | 145 | 716 | 1226 | 409 | 446 | 225 | 744 | 819 | 1535 | 253 | 412 | 713 | 590 | 676 | 795 | 529 | 749 | 171 | 326 |
| -65 | 1660 | 150 | 713 | 1281 | 404 | 445 | 248 | 727 | 965 | 1619 | 320 | 450 | 742 | 634 | 807 | 757 | 541 | 834 | 200 | 337 |
| -64 | 1752 | 157 | 728 | 1372 | 498 | 516 | 266 | 847 | 1048 | 1654 | 294 | 471 | 731 | 675 | 806 | 819 | 588 | 917 | 190 | 387 |
| -63 | 2038 | 179 | 791 | 1490 | 452 | 499 | 251 | 879 | 1037 | 1718 | 314 | 429 | 790 | 686 | 853 | 778 | 602 | 945 | 192 | 421 |
| -62 | 1932 | 170 | 786 | 1392 | 513 | 545 | 284 | 823 | 1029 | 1690 | 310 | 420 | 863 | 672 | 885 | 789 | 564 | 891 | 204 | 429 |
| -61 | 1905 | 154 | 755 | 1289 | 510 | 505 | 264 | 773 | 950 | 1576 | 267 | 381 | 878 | 618 | 803 | 780 | 527 | 869 | 212 | 425 |
| -60 | 1741 | 138 | 721 | 1162 | 454 | 470 | 249 | 780 | 899 | 1400 | 311 | 394 | 918 | 573 | 827 | 703 | 498 | 769 | 183 | 398 |
| -59 | 1439 | 119 | 590 | 1049 | 455 | 413 | 247 | 668 | 775 | 1289 | 229 | 334 | 913 | 470 | 719 | 622 | 410 | 663 | 168 | 417 |
| -58 | 1268 | 92 | 522 | 903 | 398 | 365 | 204 | 532 | 726 | 1007 | 163 | 265 | 892 | 451 | 614 | 495 | 357 | 587 | 157 | 313 |
| -57 | 1001 | 93 | 486 | 764 | 358 | 241 | 183 | 407 | 604 | 808 | 150 | 205 | 842 | 342 | 548 | 403 | 313 | 433 | 134 | 309 |
| -56 | 863 | 81 | 359 | 629 | 315 | 263 | 135 | 323 | 512 | 648 | 115 | 192 | 822 | 298 | 404 | 349 | 272 | 377 | 94 | 250 |
| -55 | 642 | 51 | 339 | 448 | 237 | 175 | 126 | 281 | 414 | 522 | 104 | 155 | 813 | 232 | 318 | 273 | 188 | 280 | 91 | 252 |
| -54 | 509 | 40 | 263 | 423 | 187 | 127 | 80 | 213 | 331 | 439 | 95 | 121 | 694 | 170 | 290 | 250 | 167 | 221 | 82 | 157 |
| -53 | 403 | 41 | 221 | 288 | 160 | 153 | 89 | 164 | 260 | 359 | 58 | 100 | 605 | 145 | 228 | 184 | 159 | 206 | 57 | 139 |
| -52 | 332 | 26 | 157 | 276 | 126 | 103 | 58 | 119 | 206 | 271 | 34 | 76 | 531 | 115 | 167 | 149 | 119 | 164 | 48 | 84 |
| -51 | 239 | 23 | 121 | 229 | 95 | 71 | 45 | 92 | 193 | 221 | 33 | 76 | 458 | 108 | 171 | 124 | 86 | 100 | 37 | 91 |
| -50 | 219 | 19 | 97 | 191 | 81 | 77 | 36 | 69 | 148 | 163 | 26 | 43 | 406 | 72 | 108 | 78 | 75 | 78 | 23 | 86 |
| -49 | 162 | 19 | 91 | 141 | 46 | 58 | 28 | 58 | 126 | 137 | 27 | 39 | 311 | 47 | 89 | 75 | 60 | 64 | 16 | 50 |
| -48 | 140 | 8 | 61 | 88 | 49 | 45 | 21 | 32 | 96 | 101 | 19 | 31 | 251 | 60 | 76 | 90 | 53 | 58 | 13 | 39 |
| -47 | 114 | 8 | 70 | 114 | 39 | 52 | 26 | 39 | 75 | 95 | 12 | 42 | 207 | 46 | 54 | 58 | 40 | 46 | 18 | 37 |
| -46 | 84 | 6 | 45 | 59 | 31 | 31 | 20 | 36 | 67 | 76 | 10 | 23 | 160 | 29 | 38 | 44 | 38 | 30 | 8 | 16 |
| -45 | 82 | 6 | 36 | 44 | 24 | 25 | 11 | 15 | 49 | 57 | 9 | 15 | 138 | 24 | 59 | 44 | 24 | 36 | 15 | 22 |
| -44 | 48 | 5 | 38 | 41 | 19 | 21 | 10 | 12 | 43 | 43 | 6 | 15 | 83 | 19 | 41 | 28 | 24 | 22 | 10 | 19 |
| -43 | 46 | 4 | 26 | 37 | 12 | 19 | 8 | 14 | 39 | 37 | 1 | 11 | 79 | 10 | 21 | 20 | 21 | 20 | 5 | 10 |
| -42 | 41 | 5 | 19 | 33 | 13 | 11 | 12 | 13 | 27 | 25 | 7 | 15 | 44 | 12 | 34 | 36 | 14 | 14 | 3 | 12 |
| -41 | 29 | 4 | 17 | 29 | 7 | 13 | 4 | 7 | 30 | 28 | 5 | 6 | 45 | 16 | 20 | 18 | 19 | 9 | 5 | 9 |
| -40 | 19 | 6 | 15 | 26 | 6 | 9 | 6 | 7 | 19 | 14 | 3 | 8 | 47 | 7 | 11 | 20 | 9 | 6 | 7 | 14 |
| -39 | 15 | 2 | 5 | 17 | 9 | 9 | 3 | 7 | 11 | 13 | 4 | 6 | 24 | 6 | 12 | 11 | 10 | 6 | 4 | 6 |
| -38 | 21 | 2 | 7 | 18 | 5 | 10 | 1 | 3 | 18 | 13 | 3 | 8 | 22 | 2 | 10 | 13 | 8 | 6 | 2 | 7 |
| -37 | 27 | 0 | 13 | 17 | 4 | 8 | 1 | 3 | 11 | 10 | 3 | 4 | 23 | 6 | 5 | 7 | 9 | 6 | 2 | 7 |
| -36 | 12 | 2 | 6 | 11 | 3 | 4 | 2 | 3 | 10 | 14 | 1 | 3 | 19 | 5 | 8 | 6 | 11 | 6 | 0 | 1 |
| -35 | 13 | 0 | 10 | 8 | 5 | 9 | 2 | 0 | 6 | 6 | 2 | 4 | 24 | 4 | 8 | 4 | 6 | 4 | 3 | 1 |
| -34 | 10 | 3 | 5 | 5 | 1 | 5 | 1 | 4 | 5 | 2 | 2 | 2 | 10 | 1 | 5 | 4 | 3 | 4 | 2 | 2 |
| -33 | 8 | 0 | 7 | 10 | 5 | 3 | 0 | 1 | 5 | 8 | 0 | 2 | 9 | 0 | 4 | 4 | 4 | 2 | 2 | 3 |
| -32 | 10 | 0 | 10 | 4 | 1 | 8 | 1 | 0 | 6 | 2 | 1 | 2 | 9 | 1 | 2 | 5 | 1 | 2 | 0 | 1 |
| -31 | 3 | 3 | 2 | 3 | 0 | 5 | 0 | 1 | 2 | 4 | 1 | 0 | 7 | 2 | 3 | 4 | 2 | 2 | 1 | 2 |
| -30 | 6 | 0 | 4 | 3 | 1 | 2 | 1 | 4 | 4 | 2 | 0 | 1 | 4 | 1 | 2 | 3 | 2 | 2 | 0 | 1 |
| -29 | 5 | 2 | 2 | 6 | 2 | 1 | 2 | 0 | 2 | 1 | 1 | 4 | 2 | 1 | 2 | 4 | 0 | 2 | 0 | 0 |
| -28 | 7 | 2 | 1 | 3 | 0 | 1 | 1 | 3 | 2 | 1 | 1 | 0 | 6 | 0 | 1 | 1 | 1 | 1 | 0 | 1 |
| -27 | 1 | 1 | 2 | 3 | 1 | 2 | 1 | 1 | 6 | 0 | 0 | 0 | 3 | 2 | 3 | 2 | 4 | 2 | 1 | 1 |
| -26 | 3 | 0 | 2 | 3 | 1 | 1 | 0 | 0 | 2 | 3 | 0 | 0 | 3 | 1 | 1 | 2 | 1 | 2 | 0 | 1 |
| -25 | 0 | 0 | 2 | 1 | 0 | 1 | 1 | 0 | 2 | 1 | 0 | 0 | 3 | 0 | 2 | 3 | 0 | 0 | 0 | 1 |
| -24 | 0 | 0 | 2 | 4 | 0 | 1 | 1 | 0 | 3 | 0 | 1 | 2 | 3 | 2 | 2 | 2 | 0 | 0 | 0 | 0 |
| -23 | 3 | 0 | 3 | 2 | 0 | 0 | 0 | 1 | 1 | 1 | 0 | 0 | 5 | 1 | 4 | 0 | 0 | 0 | 0 | 0 |
| -22 | 3 | 0 | 0 | 2 | 0 | 0 | 0 | 1 | 4 | 0 | 1 | 0 | 0 | 0 | 0 | 0 | 0 | 0 | 0 | 0 |
| -21 | 1 | 0 | 1 | 0 | 2 | 0 | 0 | 1 | 1 | 0 | 1 | 2 | 2 | 1 | 1 | 0 | 0 | 4 | 1 | 0 |
| -20 | 1 | 0 | 3 | 1 | 0 | 1 | 0 | 1 | 2 | 0 | 0 | 0 | 1 | 1 | 1 | 1 | 0 | 0 | 1 | 1 |
| -19 | 1 | 0 | 1 | 1 | 0 | 1 | 0 | 0 | 1 | 0 | 1 | 1 | 2 | 1 | 1 | 0 | 1 | 0 | 0 | 1 |
| -18 | 2 | 0 | 1 | 0 | 0 | 1 | 0 | 1 | 2 | 0 | 0 | 1 | 1 | 0 | 0 | 1 | 1 | 0 | 0 | 0 |
| -17 | 1 | 1 | 0 | 0 | 0 | 1 | 0 | 0 | 1 | 0 | 0 | 3 | 1 | 1 | 0 | 2 | 1 | 0 | 0 | 1 |
| -16 | 0 | 0 | 3 | 1 | 0 | 3 | 0 | 0 | 1 | 0 | 0 | 2 | 1 | 0 | 0 | 1 | 2 | 1 | 1 | 1 |
| -15 | 1 | 0 | 0 | 1 | 1 | 1 | 1 | 1 | 1 | 0 | 0 | 0 | 2 | 0 | 0 | 1 | 2 | 1 | 0 | 0 |
| -14 | 0 | 2 | 0 | 1 | 0 | 2 | 0 | 0 | 0 | 0 | 0 | 0 | 0 | 0 | 0 | 2 | 0 | 0 | 0 | 0 |
| -13 | 0 | 0 | 0 | 1 | 0 | 1 | 0 | 0 | 2 | 1 | 0 | 0 | 1 | 0 | 1 | 1 | 0 | 1 | 0 | 0 |
| -12 | 0 | 0 | 0 | 0 | 0 | 2 | 1 | 0 | 0 | 1 | 1 | 1 | 0 | 0 | 0 | 0 | 0 | 1 | 1 | 0 |
| -11 | 1 | 0 | 1 | 2 | 0 | 1 | 1 | 0 | 0 | 0 | 2 | 1 | 1 | 0 | 1 | 0 | 0 | 1 | 0 | 0 |
| -10 | 1 | 0 | 1 | 0 | 0 | 1 | 0 | 0 | 0 | 0 | 0 | 1 | 1 | 0 | 0 | 1 | 2 | 0 | 0 | 0 |
| -9 | 0 | 0 | 2 | 0 | 0 | 0 | 0 | 1 | 0 | 0 | 0 | 1 | 1 | 0 | 0 | 1 | 2 | 1 | 0 | 0 |
| -8 | 1 | 0 | 1 | 0 | 0 | 0 | 0 | 1 | 2 | 1 | 1 | 0 | 1 | 1 | 1 | 1 | 1 | 0 | 0 | 0 |
| -7 | 0 | 0 | 0 | 1 | 0 | 2 | 0 | 0 | 0 | 0 | 0 | 0 | 1 | 0 | 1 | 2 | 0 | 2 | 0 | 1 |
| -6 | 1 | 0 | 1 | 0 | 0 | 2 | 0 | 0 | 0 | 2 | 0 | 0 | 3 | 2 | 1 | 2 | 0 | 0 | 0 | 0 |
| -5 | 2 | 0 | 2 | 0 | 0 | 0 | 1 | 1 | 0 | 1 | 0 | 1 | 0 | 1 | 0 | 0 | 0 | 1 | 0 | 0 |
| -4 | 2 | 0 | 2 | 2 | 0 | 2 | 0 | 0 | 1 | 0 | 0 | 2 | 0 | 0 | 2 | 0 | 1 | 0 | 0 | 0 |
| -3 | 0 | 0 | 0 | 1 | 2 | 1 | 0 | 0 | 0 | 0 | 0 | 0 | 0 | 0 | 0 | 0 | 0 | 1 | 0 | 0 |
| -2 | 0 | 0 | 0 | 3 | 0 | 0 | 0 | 1 | 0 | 0 | 1 | 1 | 0 | 0 | 0 | 1 | 0 | 0 | 0 | 0 |
| -1 | 0 | 0 | 0 | 0 | 0 | 1 | 1 | 0 | 1 | 1 | 0 | 0 | 1 | 1 | 1 | 1 | 0 | 0 | 0 | 0 |
| 0 | 0 | 0 | 1 | 0 | 0 | 4 | 0 | 1 | 2 | 0 | 0 | 0 | 0 | 0 | 1 | 2 | 0 | 1 | 0 | 0 |
| 1 | 0 | 0 | 0 | 0 | 0 | 0 | 0 | 0 | 0 | 0 | 1 | 0 | 0 | 0 | 0 | 1 | 0 | 0 | 0 | 1 |
| 2 | 1 | 0 | 0 | 0 | 0 | 0 | 0 | 0 | 1 | 1 | 0 | 0 | 0 | 0 | 0 | 0 | 1 | 0 | 0 | 0 |
| 3 | 1 | 0 | 0 | 0 | 0 | 1 | 0 | 0 | 0 | 0 | 0 | 1 | 0 | 1 | 0 | 0 | 1 | 0 | 0 | 1 |
| 4 | 0 | 0 | 1 | 0 | 0 | 0 | 1 | 0 | 0 | 0 | 0 | 2 | 3 | 0 | 1 | 0 | 0 | 0 | 0 | 0 |
| 5 | 0 | 0 | 0 | 1 | 0 | 1 | 0 | 0 | 0 | 2 | 0 | 0 | 0 | 0 | 0 | 0 | 1 | 0 | 0 | 1 |
| 6 | 0 | 0 | 1 | 0 | 0 | 1 | 0 | 0 | 0 | 0 | 0 | 0 | 1 | 0 | 0 | 0 | 0 | 0 | 0 | 0 |
| 7 | 0 | 0 | 0 | 1 | 0 | 0 | 1 | 0 | 1 | 0 | 0 | 1 | 0 | 0 | 0 | 0 | 0 | 0 | 0 | 0 |
| 8 | 0 | 0 | 0 | 2 | 0 | 2 | 0 | 0 | 0 | 0 | 0 | 0 | 1 | 0 | 0 | 0 | 1 | 0 | 0 | 0 |
| 9 | 0 | 0 | 1 | 0 | 0 | 1 | 1 | 0 | 0 | 0 | 0 | 0 | 1 | 1 | 0 | 0 | 0 | 0 | 0 | 0 |
| 10 | 0 | 0 | 2 | 1 | 0 | 1 | 0 | 0 | 1 | 0 | 0 | 3 | 0 | 0 | 0 | 0 | 0 | 0 | 0 | 0 |
| 11 | 1 | 0 | 1 | 1 | 0 | 1 | 0 | 0 | 0 | 1 | 0 | 0 | 0 | 0 | 0 | 0 | 0 | 0 | 0 | 0 |
| 12 | 0 | 0 | 0 | 0 | 0 | 1 | 0 | 1 | 0 | 0 | 0 | 0 | 0 | 0 | 0 | 0 | 0 | 0 | 0 | 1 |
| 13 | 3 | 0 | 1 | 0 | 0 | 0 | 0 | 0 | 0 | 1 | 0 | 0 | 0 | 0 | 0 | 0 | 0 | 0 | 0 | 0 |
| 14 | 0 | 0 | 1 | 0 | 0 | 3 | 0 | 1 | 2 | 0 | 0 | 1 | 0 | 0 | 0 | 1 | 0 | 1 | 0 | 0 |
| 15 | 2 | 0 | 0 | 0 | 0 | 1 | 0 | 0 | 1 | 0 | 0 | 0 | 0 | 0 | 0 | 1 | 1 | 0 | 0 | 0 |
| 16 | 0 | 0 | 0 | 1 | 0 | 3 | 1 | 0 | 1 | 0 | 0 | 0 | 0 | 0 | 0 | 2 | 1 | 0 | 0 | 0 |
| 17 | 0 | 0 | 0 | 0 | 1 | 2 | 1 | 0 | 0 | 1 | 0 | 1 | 0 | 0 | 0 | 0 | 0 | 0 | 0 | 0 |
| 18 | 1 | 0 | 1 | 0 | 0 | 1 | 0 | 1 | 0 | 2 | 0 | 1 | 0 | 0 | 0 | 0 | 0 | 0 | 0 | 0 |
| 19 | 0 | 0 | 0 | 0 | 0 | 0 | 2 | 0 | 1 | 1 | 0 | 0 | 0 | 0 | 0 | 1 | 0 | 1 | 0 | 1 |
| 20 | 1 | 0 | 0 | 0 | 0 | 0 | 3 | 0 | 0 | 1 | 0 | 1 | 0 | 0 | 0 | 1 | 1 | 0 | 0 | 0 |
| 21 | 0 | 0 | 0 | 0 | 0 | 3 | 0 | 0 | 0 | 0 | 0 | 0 | 0 | 1 | 0 | 0 | 0 | 0 | 0 | 0 |
| 22 | 0 | 1 | 1 | 0 | 0 | 2 | 0 | 0 | 0 | 1 | 0 | 0 | 0 | 0 | 0 | 2 | 0 | 0 | 0 | 0 |
| 23 | 0 | 0 | 0 | 0 | 0 | 0 | 1 | 0 | 2 | 0 | 0 | 0 | 1 | 0 | 1 | 0 | 0 | 1 | 0 | 0 |
| 24 | 1 | 0 | 2 | 1 | 0 | 2 | 0 | 0 | 1 | 0 | 0 | 2 | 3 | 2 | 1 | 2 | 0 | 1 | 0 | 1 |
| 25 | 1 | 0 | 0 | 0 | 1 | 2 | 0 | 0 | 1 | 1 | 0 | 0 | 0 | 0 | 0 | 0 | 0 | 0 | 0 | 1 |
| 26 | 0 | 0 | 2 | 0 | 0 | 1 | 0 | 1 | 1 | 0 | 0 | 0 | 0 | 0 | 0 | 1 | 0 | 1 | 1 | 0 |
| 27 | 0 | 0 | 2 | 1 | 0 | 2 | 0 | 0 | 1 | 2 | 0 | 2 | 0 | 0 | 2 | 1 | 0 | 1 | 0 | 1 |
| 28 | 3 | 0 | 0 | 1 | 0 | 2 | 1 | 0 | 2 | 0 | 0 | 1 | 0 | 1 | 1 | 0 | 1 | 0 | 0 | 0 |
| 29 | 1 | 0 | 2 | 2 | 0 | 1 | 0 | 0 | 2 | 1 | 0 | 1 | 0 | 0 | 1 | 3 | 0 | 0 | 0 | 0 |
| 30 | 0 | 1 | 0 | 1 | 0 | 6 | 1 | 1 | 0 | 0 | 0 | 0 | 0 | 1 | 0 | 1 | 0 | 1 | 0 | 0 |
| 31 | 1 | 0 | 4 | 3 | 0 | 4 | 1 | 0 | 2 | 0 | 0 | 3 | 0 | 1 | 0 | 1 | 0 | 0 | 0 | 0 |
| 32 | 2 | 0 | 3 | 4 | 0 | 2 | 1 | 1 | 1 | 0 | 0 | 5 | 0 | 2 | 2 | 3 | 0 | 0 | 0 | 3 |
| 33 | 1 | 0 | 1 | 1 | 1 | 1 | 4 | 1 | 1 | 0 | 0 | 5 | 0 | 1 | 2 | 1 | 1 | 0 | 0 | 0 |
| 34 | 0 | 1 | 3 | 1 | 1 | 5 | 0 | 0 | 3 | 0 | 1 | 3 | 0 | 0 | 2 | 3 | 2 | 1 | 0 | 1 |
| 35 | 2 | 0 | 6 | 0 | 1 | 4 | 0 | 0 | 3 | 0 | 1 | 2 | 0 | 3 | 1 | 2 | 3 | 0 | 0 | 0 |
| 36 | 1 | 1 | 4 | 3 | 0 | 7 | 0 | 1 | 4 | 0 | 1 | 5 | 0 | 0 | 1 | 1 | 2 | 1 | 0 | 0 |
| 37 | 3 | 0 | 6 | 3 | 0 | 6 | 2 | 0 | 1 | 0 | 1 | 6 | 0 | 3 | 4 | 5 | 1 | 4 | 1 | 0 |
| 38 | 5 | 0 | 11 | 6 | 3 | 6 | 2 | 2 | 2 | 3 | 0 | 9 | 0 | 1 | 1 | 6 | 5 | 2 | 0 | 2 |
| 39 | 3 | 1 | 9 | 11 | 1 | 12 | 1 | 0 | 4 | 1 | 0 | 11 | 0 | 1 | 2 | 2 | 4 | 2 | 2 | 2 |
| 40 | 4 | 0 | 12 | 7 | 2 | 6 | 4 | 0 | 2 | 2 | 0 | 18 | 0 | 3 | 3 | 3 | 1 | 2 | 0 | 1 |
| 41 | 5 | 1 | 13 | 8 | 2 | 6 | 4 | 2 | 9 | 7 | 1 | 19 | 0 | 6 | 1 | 4 | 2 | 1 | 1 | 3 |
| 42 | 6 | 1 | 16 | 9 | 3 | 8 | 5 | 0 | 8 | 4 | 0 | 19 | 0 | 5 | 6 | 11 | 3 | 0 | 0 | 3 |
| 43 | 13 | 1 | 18 | 15 | 3 | 16 | 4 | 0 | 8 | 1 | 1 | 33 | 0 | 7 | 10 | 8 | 4 | 1 | 1 | 3 |
| 44 | 10 | 2 | 23 | 10 | 2 | 17 | 9 | 0 | 9 | 2 | 2 | 36 | 0 | 6 | 9 | 7 | 2 | 0 | 0 | 0 |
| 45 | 9 | 2 | 37 | 14 | 7 | 20 | 7 | 1 | 22 | 4 | 1 | 40 | 0 | 12 | 14 | 16 | 6 | 1 | 2 | 11 |
| 46 | 9 | 4 | 40 | 10 | 4 | 22 | 7 | 0 | 13 | 7 | 2 | 51 | 0 | 10 | 11 | 11 | 2 | 5 | 1 | 4 |
| 47 | 9 | 3 | 47 | 23 | 4 | 32 | 12 | 1 | 20 | 10 | 2 | 53 | 0 | 11 | 24 | 19 | 2 | 3 | 1 | 6 |
| 48 | 10 | 3 | 48 | 15 | 2 | 37 | 13 | 1 | 14 | 6 | 3 | 69 | 0 | 19 | 16 | 17 | 5 | 3 | 2 | 11 |
| 49 | 19 | 4 | 41 | 28 | 7 | 41 | 8 | 2 | 29 | 12 | 2 | 81 | 0 | 22 | 21 | 11 | 2 | 1 | 2 | 3 |
| 50 | 22 | 5 | 61 | 21 | 10 | 43 | 20 | 1 | 37 | 16 | 5 | 91 | 0 | 16 | 21 | 17 | 4 | 2 | 1 | 8 |
| 51 | 34 | 7 | 56 | 29 | 9 | 55 | 15 | 1 | 27 | 10 | 9 | 107 | 0 | 23 | 33 | 23 | 2 | 3 | 4 | 14 |
| 52 | 27 | 4 | 68 | 29 | 7 | 70 | 19 | 0 | 34 | 12 | 5 | 95 | 0 | 29 | 29 | 32 | 2 | 3 | 3 | 7 |
| 53 | 29 | 9 | 62 | 27 | 8 | 73 | 26 | 0 | 39 | 15 | 6 | 104 | 1 | 22 | 31 | 34 | 7 | 2 | 3 | 9 |
| 54 | 21 | 3 | 77 | 30 | 16 | 78 | 24 | 2 | 53 | 17 | 4 | 138 | 0 | 29 | 35 | 27 | 3 | 3 | 3 | 9 |
| 55 | 35 | 12 | 68 | 26 | 20 | 93 | 23 | 1 | 46 | 18 | 7 | 101 | 0 | 32 | 35 | 23 | 2 | 2 | 4 | 13 |
| 56 | 32 | 5 | 72 | 25 | 13 | 108 | 14 | 1 | 50 | 17 | 4 | 97 | 0 | 17 | 26 | 23 | 4 | 1 | 4 | 20 |
| 57 | 20 | 5 | 70 | 33 | 15 | 132 | 27 | 0 | 43 | 18 | 7 | 124 | 0 | 27 | 32 | 25 | 2 | 1 | 4 | 13 |
| 58 | 13 | 7 | 72 | 25 | 14 | 140 | 24 | 1 | 33 | 10 | 3 | 122 | 0 | 25 | 24 | 24 | 5 | 1 | 6 | 18 |
| 59 | 22 | 8 | 65 | 31 | 16 | 133 | 18 | 2 | 35 | 12 | 4 | 103 | 0 | 21 | 31 | 14 | 1 | 1 | 5 | 14 |
| 60 | 31 | 4 | 57 | 24 | 15 | 147 | 22 | 4 | 28 | 10 | 4 | 102 | 0 | 12 | 16 | 24 | 6 | 4 | 5 | 10 |
| 61 | 12 | 3 | 53 | 18 | 11 | 156 | 19 | 2 | 37 | 14 | 3 | 105 | 0 | 16 | 21 | 19 | 1 | 2 | 5 | 11 |
| 62 | 12 | 5 | 47 | 21 | 17 | 183 | 18 | 1 | 35 | 25 | 4 | 92 | 0 | 20 | 27 | 16 | 1 | 2 | 7 | 12 |
| 63 | 11 | 8 | 44 | 15 | 10 | 220 | 14 | 1 | 42 | 13 | 7 | 77 | 0 | 15 | 20 | 12 | 4 | 3 | 3 | 10 |
| 64 | 13 | 4 | 41 | 19 | 11 | 215 | 18 | 2 | 32 | 11 | 3 | 59 | 0 | 12 | 24 | 12 | 1 | 0 | 0 | 11 |
| 65 | 11 | 5 | 33 | 19 | 11 | 209 | 13 | 1 | 24 | 13 | 5 | 65 | 0 | 17 | 16 | 15 | 0 | 4 | 1 | 8 |
| 66 | 15 | 7 | 26 | 19 | 5 | 262 | 17 | 0 | 17 | 7 | 4 | 59 | 0 | 17 | 20 | 14 | 1 | 2 | 1 | 11 |
| 67 | 12 | 5 | 18 | 13 | 4 | 265 | 8 | 0 | 22 | 13 | 0 | 35 | 0 | 10 | 19 | 10 | 7 | 4 | 2 | 12 |
| 68 | 11 | 2 | 34 | 16 | 7 | 270 | 15 | 1 | 17 | 6 | 2 | 50 | 1 | 9 | 16 | 7 | 4 | 2 | 1 | 8 |
| 69 | 8 | 2 | 28 | 14 | 11 | 280 | 8 | 0 | 14 | 13 | 3 | 43 | 0 | 8 | 10 | 9 | 4 | 1 | 2 | 5 |
| 70 | 5 | 2 | 21 | 7 | 6 | 318 | 11 | 3 | 12 | 7 | 6 | 35 | 0 | 5 | 10 | 9 | 2 | 1 | 2 | 5 |
| 71 | 7 | 0 | 16 | 12 | 2 | 302 | 11 | 1 | 10 | 7 | 6 | 34 | 0 | 13 | 10 | 13 | 4 | 1 | 3 | 7 |
| 72 | 6 | 2 | 23 | 10 | 3 | 317 | 11 | 0 | 13 | 6 | 2 | 35 | 0 | 5 | 11 | 15 | 5 | 2 | 1 | 1 |
| 73 | 7 | 2 | 17 | 7 | 5 | 342 | 14 | 2 | 11 | 5 | 2 | 25 | 0 | 8 | 6 | 7 | 3 | 2 | 0 | 15 |
| 74 | 5 | 0 | 13 | 6 | 5 | 348 | 8 | 0 | 12 | 11 | 2 | 18 | 0 | 4 | 6 | 1 | 4 | 0 | 1 | 6 |
| 75 | 4 | 0 | 13 | 9 | 6 | 384 | 7 | 0 | 10 | 4 | 1 | 12 | 0 | 4 | 5 | 8 | 5 | 2 | 1 | 6 |
| 76 | 1 | 1 | 7 | 8 | 5 | 361 | 4 | 1 | 3 | 2 | 1 | 20 | 0 | 4 | 8 | 5 | 5 | 1 | 1 | 2 |
| 77 | 3 | 1 | 10 | 6 | 3 | 377 | 0 | 2 | 6 | 4 | 4 | 12 | 0 | 2 | 2 | 4 | 6 | 0 | 4 | 4 |
| 78 | 4 | 0 | 5 | 8 | 2 | 384 | 2 | 2 | 2 | 5 | 0 | 11 | 0 | 4 | 4 | 5 | 6 | 1 | 0 | 1 |
| 79 | 3 | 1 | 3 | 3 | 4 | 403 | 1 | 2 | 5 | 3 | 0 | 9 | 0 | 0 | 2 | 6 | 0 | 1 | 2 | 1 |
| 80 | 3 | 0 | 5 | 6 | 4 | 384 | 4 | 1 | 5 | 1 | 2 | 5 | 0 | 0 | 3 | 4 | 4 | 0 | 0 | 3 |
| 81 | 1 | 0 | 4 | 6 | 1 | 411 | 1 | 1 | 3 | 4 | 0 | 8 | 0 | 1 | 3 | 4 | 1 | 0 | 0 | 1 |
| 82 | 3 | 2 | 5 | 2 | 2 | 421 | 1 | 2 | 5 | 2 | 1 | 13 | 0 | 2 | 1 | 3 | 1 | 0 | 0 | 3 |
| 83 | 2 | 1 | 6 | 3 | 3 | 406 | 1 | 2 | 4 | 3 | 1 | 5 | 0 | 0 | 1 | 5 | 0 | 0 | 0 | 3 |
| 84 | 1 | 1 | 3 | 0 | 1 | 420 | 0 | 0 | 7 | 1 | 2 | 5 | 0 | 1 | 4 | 1 | 3 | 1 | 0 | 0 |
| 85 | 2 | 3 | 2 | 0 | 1 | 371 | 1 | 0 | 2 | 0 | 0 | 1 | 0 | 1 | 0 | 4 | 0 | 1 | 1 | 0 |
| 86 | 0 | 0 | 0 | 0 | 3 | 438 | 0 | 0 | 0 | 2 | 1 | 3 | 0 | 0 | 1 | 1 | 2 | 1 | 1 | 0 |
| 87 | 0 | 0 | 1 | 2 | 1 | 375 | 1 | 0 | 1 | 0 | 1 | 1 | 0 | 0 | 2 | 1 | 2 | 0 | 0 | 1 |
| 88 | 1 | 1 | 4 | 2 | 0 | 372 | 1 | 0 | 0 | 2 | 0 | 0 | 0 | 1 | 0 | 2 | 3 | 0 | 0 | 1 |
| 89 | 0 | 0 | 1 | 1 | 0 | 376 | 0 | 0 | 0 | 1 | 0 | 0 | 0 | 1 | 0 | 2 | 2 | 1 | 0 | 1 |
| 90 | 0 | 0 | 2 | 1 | 0 | 313 | 0 | 0 | 2 | 0 | 0 | 0 | 0 | 3 | 0 | 0 | 0 | 0 | 0 | 1 |
| 91 | 0 | 0 | 2 | 1 | 1 | 329 | 1 | 0 | 1 | 2 | 0 | 1 | 0 | 0 | 2 | 0 | 1 | 1 | 0 | 0 |
| 92 | 1 | 0 | 0 | 3 | 0 | 291 | 0 | 0 | 1 | 0 | 1 | 0 | 0 | 0 | 0 | 1 | 1 | 1 | 2 | 0 |
| 93 | 1 | 2 | 2 | 1 | 1 | 341 | 0 | 1 | 0 | 0 | 0 | 1 | 0 | 1 | 2 | 2 | 0 | 1 | 0 | 0 |
| 94 | 0 | 0 | 0 | 1 | 0 | 324 | 1 | 0 | 0 | 2 | 0 | 0 | 0 | 0 | 0 | 2 | 0 | 1 | 0 | 0 |
| 95 | 0 | 0 | 0 | 1 | 0 | 300 | 1 | 0 | 0 | 1 | 0 | 0 | 0 | 1 | 0 | 3 | 0 | 0 | 0 | 1 |
| 96 | 2 | 0 | 0 | 1 | 0 | 287 | 0 | 0 | 0 | 1 | 0 | 1 | 0 | 0 | 2 | 1 | 0 | 0 | 0 | 0 |
| 97 | 1 | 0 | 1 | 1 | 0 | 267 | 0 | 0 | 2 | 0 | 0 | 1 | 0 | 0 | 0 | 0 | 0 | 0 | 0 | 0 |
| 98 | 0 | 0 | 0 | 1 | 0 | 251 | 0 | 0 | 1 | 0 | 0 | 0 | 0 | 0 | 0 | 1 | 2 | 0 | 0 | 1 |
| 99 | 0 | 0 | 0 | 0 | 0 | 230 | 0 | 0 | 0 | 0 | 0 | 1 | 0 | 0 | 1 | 2 | 0 | 0 | 0 | 0 |
| 100 | 0 | 0 | 0 | 1 | 0 | 193 | 0 | 0 | 0 | 0 | 0 | 0 | 0 | 0 | 0 | 0 | 1 | 0 | 0 | 0 |
| 101 | 1 | 0 | 0 | 1 | 1 | 199 | 0 | 0 | 0 | 0 | 0 | 1 | 0 | 0 | 1 | 0 | 1 | 0 | 0 | 0 |
| 102 | 0 | 0 | 0 | 1 | 0 | 206 | 0 | 0 | 0 | 0 | 0 | 2 | 0 | 1 | 0 | 1 | 0 | 0 | 0 | 1 |
| 103 | 0 | 0 | 0 | 0 | 0 | 185 | 0 | 0 | 1 | 0 | 0 | 2 | 0 | 0 | 0 | 1 | 0 | 0 | 0 | 0 |
| 104 | 1 | 0 | 0 | 0 | 0 | 151 | 0 | 1 | 1 | 0 | 0 | 1 | 0 | 0 | 0 | 1 | 0 | 0 | 0 | 0 |
| 105 | 1 | 0 | 3 | 0 | 0 | 136 | 0 | 0 | 0 | 0 | 0 | 1 | 0 | 0 | 1 | 0 | 0 | 1 | 0 | 0 |
| 106 | 1 | 0 | 0 | 0 | 0 | 135 | 0 | 0 | 0 | 0 | 0 | 0 | 0 | 0 | 0 | 1 | 0 | 0 | 0 | 0 |
| 107 | 1 | 0 | 1 | 0 | 0 | 126 | 0 | 0 | 0 | 0 | 0 | 0 | 0 | 0 | 0 | 0 | 1 | 0 | 0 | 0 |
| 108 | 1 | 0 | 1 | 0 | 0 | 121 | 0 | 0 | 0 | 0 | 0 | 1 | 0 | 0 | 0 | 0 | 0 | 0 | 0 | 0 |
| 109 | 0 | 0 | 1 | 0 | 0 | 128 | 1 | 1 | 0 | 0 | 0 | 0 | 0 | 0 | 0 | 0 | 0 | 0 | 0 | 0 |
| 110 | 0 | 0 | 0 | 1 | 1 | 106 | 1 | 0 | 0 | 0 | 0 | 0 | 0 | 0 | 1 | 0 | 0 | 0 | 0 | 0 |
| 111 | 0 | 0 | 1 | 0 | 0 | 87 | 0 | 1 | 2 | 0 | 0 | 0 | 0 | 1 | 0 | 0 | 0 | 0 | 0 | 0 |
| 112 | 0 | 0 | 0 | 2 | 0 | 87 | 0 | 1 | 0 | 0 | 0 | 0 | 0 | 0 | 0 | 0 | 0 | 0 | 0 | 0 |
| 113 | 0 | 0 | 0 | 0 | 0 | 95 | 0 | 0 | 0 | 0 | 0 | 0 | 0 | 0 | 0 | 0 | 1 | 0 | 0 | 0 |
| 114 | 0 | 0 | 0 | 0 | 0 | 86 | 0 | 0 | 0 | 0 | 0 | 0 | 0 | 0 | 0 | 0 | 0 | 0 | 0 | 0 |
| 115 | 0 | 0 | 0 | 0 | 0 | 73 | 0 | 0 | 0 | 0 | 0 | 0 | 0 | 0 | 0 | 1 | 1 | 0 | 0 | 1 |
| 116 | 2 | 0 | 0 | 0 | 0 | 63 | 0 | 0 | 1 | 0 | 1 | 1 | 0 | 0 | 0 | 0 | 0 | 0 | 0 | 0 |
| 117 | 0 | 0 | 1 | 0 | 0 | 61 | 0 | 0 | 0 | 0 | 0 | 0 | 0 | 0 | 0 | 0 | 0 | 0 | 0 | 0 |
| 118 | 1 | 0 | 0 | 0 | 0 | 61 | 0 | 0 | 0 | 0 | 0 | 1 | 0 | 0 | 0 | 0 | 0 | 0 | 0 | 0 |
| 119 | 0 | 0 | 0 | 1 | 0 | 68 | 0 | 0 | 0 | 0 | 0 | 0 | 0 | 0 | 0 | 0 | 0 | 0 | 0 | 0 |
| 120 | 0 | 0 | 0 | 0 | 0 | 42 | 0 | 0 | 0 | 0 | 1 | 0 | 0 | 0 | 0 | 1 | 0 | 0 | 0 | 0 |
| 121 | 1 | 0 | 0 | 0 | 0 | 61 | 0 | 1 | 0 | 2 | 0 | 0 | 0 | 0 | 1 | 0 | 0 | 0 | 0 | 0 |
| 122 | 0 | 0 | 0 | 0 | 0 | 63 | 0 | 0 | 0 | 0 | 0 | 0 | 0 | 0 | 0 | 1 | 0 | 0 | 0 | 0 |
| 123 | 0 | 0 | 0 | 0 | 0 | 59 | 0 | 0 | 0 | 0 | 0 | 0 | 0 | 0 | 0 | 0 | 0 | 0 | 0 | 0 |
| 124 | 1 | 0 | 1 | 0 | 0 | 39 | 1 | 0 | 0 | 0 | 0 | 0 | 0 | 0 | 0 | 0 | 0 | 0 | 0 | 0 |
| 125 | 0 | 0 | 1 | 0 | 0 | 55 | 0 | 0 | 0 | 0 | 0 | 0 | 0 | 1 | 0 | 0 | 0 | 0 | 0 | 0 |
| 126 | 0 | 0 | 0 | 0 | 0 | 47 | 1 | 0 | 0 | 0 | 0 | 0 | 0 | 1 | 0 | 0 | 0 | 0 | 0 | 0 |
| 127 | 0 | 0 | 1 | 0 | 0 | 49 | 0 | 0 | 0 | 0 | 1 | 0 | 0 | 0 | 0 | 0 | 0 | 0 | 0 | 0 |
| 128 | 0 | 0 | 0 | 1 | 0 | 52 | 0 | 0 | 0 | 0 | 0 | 1 | 0 | 0 | 0 | 0 | 0 | 0 | 0 | 0 |
| 129 | 0 | 0 | 0 | 0 | 0 | 44 | 0 | 0 | 0 | 1 | 0 | 0 | 0 | 0 | 0 | 0 | 0 | 0 | 0 | 0 |
| 130 | 0 | 0 | 0 | 0 | 0 | 45 | 0 | 0 | 1 | 0 | 0 | 0 | 0 | 0 | 0 | 1 | 0 | 0 | 0 | 0 |
| 131 | 0 | 0 | 0 | 0 | 0 | 32 | 0 | 0 | 0 | 0 | 0 | 0 | 0 | 0 | 0 | 0 | 0 | 0 | 0 | 0 |
| 132 | 0 | 0 | 0 | 0 | 0 | 35 | 0 | 0 | 0 | 0 | 0 | 0 | 0 | 0 | 0 | 0 | 0 | 0 | 0 | 1 |
| 133 | 0 | 0 | 0 | 0 | 0 | 31 | 0 | 0 | 1 | 0 | 0 | 0 | 0 | 0 | 0 | 0 | 0 | 0 | 0 | 0 |
| 134 | 1 | 0 | 2 | 0 | 0 | 38 | 0 | 0 | 1 | 0 | 0 | 0 | 0 | 0 | 0 | 0 | 0 | 0 | 0 | 0 |
| 135 | 0 | 0 | 1 | 1 | 0 | 36 | 0 | 0 | 0 | 0 | 0 | 0 | 0 | 0 | 0 | 1 | 0 | 0 | 0 | 0 |
| 136 | 0 | 0 | 0 | 0 | 0 | 51 | 0 | 0 | 0 | 0 | 0 | 0 | 0 | 0 | 0 | 0 | 1 | 0 | 0 | 0 |
| 137 | 0 | 0 | 0 | 1 | 0 | 36 | 0 | 0 | 0 | 0 | 0 | 0 | 0 | 1 | 0 | 1 | 0 | 0 | 0 | 0 |
| 138 | 0 | 0 | 0 | 0 | 0 | 26 | 1 | 0 | 1 | 0 | 0 | 0 | 0 | 0 | 0 | 0 | 0 | 0 | 0 | 0 |
| 139 | 0 | 0 | 0 | 1 | 1 | 35 | 0 | 0 | 0 | 0 | 0 | 2 | 0 | 0 | 0 | 0 | 0 | 0 | 0 | 0 |
| 140 | 0 | 0 | 0 | 0 | 0 | 32 | 0 | 0 | 1 | 1 | 0 | 0 | 0 | 0 | 0 | 0 | 1 | 0 | 0 | 0 |
| 141 | 0 | 0 | 0 | 0 | 0 | 26 | 0 | 0 | 0 | 1 | 0 | 0 | 0 | 0 | 0 | 0 | 0 | 0 | 0 | 0 |
| 142 | 0 | 0 | 0 | 0 | 0 | 35 | 0 | 0 | 0 | 0 | 0 | 0 | 0 | 0 | 0 | 0 | 0 | 0 | 0 | 0 |
| 143 | 1 | 0 | 1 | 0 | 0 | 47 | 0 | 0 | 0 | 0 | 0 | 0 | 0 | 0 | 0 | 0 | 0 | 0 | 0 | 0 |
| 144 | 0 | 0 | 0 | 1 | 0 | 47 | 0 | 0 | 0 | 0 | 0 | 0 | 0 | 0 | 1 | 1 | 1 | 0 | 0 | 0 |
| 145 | 0 | 0 | 0 | 0 | 0 | 33 | 0 | 0 | 0 | 0 | 0 | 0 | 0 | 0 | 0 | 2 | 0 | 0 | 0 | 0 |
| 146 | 0 | 0 | 1 | 0 | 0 | 36 | 1 | 0 | 0 | 1 | 0 | 0 | 0 | 0 | 0 | 0 | 0 | 0 | 0 | 0 |
| 147 | 0 | 0 | 1 | 0 | 0 | 44 | 0 | 0 | 0 | 0 | 0 | 0 | 0 | 0 | 0 | 2 | 0 | 0 | 0 | 0 |
| 148 | 1 | 0 | 0 | 0 | 0 | 53 | 0 | 0 | 0 | 0 | 0 | 0 | 0 | 0 | 0 | 1 | 0 | 0 | 0 | 0 |
| 149 | 1 | 0 | 1 | 0 | 0 | 38 | 0 | 0 | 0 | 0 | 0 | 0 | 0 | 0 | 0 | 0 | 0 | 0 | 0 | 0 |
| 150 | 0 | 0 | 0 | 0 | 0 | 43 | 0 | 0 | 0 | 0 | 1 | 0 | 0 | 0 | 1 | 0 | 0 | 0 | 0 | 0 |
| 151 | 1 | 0 | 0 | 0 | 0 | 45 | 1 | 0 | 0 | 1 | 0 | 0 | 0 | 0 | 1 | 0 | 1 | 0 | 0 | 0 |
| 152 | 0 | 0 | 0 | 0 | 1 | 48 | 0 | 0 | 0 | 0 | 0 | 0 | 0 | 0 | 1 | 0 | 0 | 0 | 0 | 0 |
| 153 | 2 | 1 | 2 | 0 | 0 | 47 | 0 | 1 | 0 | 0 | 0 | 1 | 0 | 0 | 0 | 0 | 0 | 0 | 0 | 0 |
| 154 | 0 | 0 | 1 | 2 | 0 | 40 | 1 | 0 | 0 | 0 | 0 | 0 | 0 | 0 | 0 | 1 | 1 | 0 | 0 | 0 |
| 155 | 1 | 0 | 0 | 0 | 0 | 32 | 0 | 0 | 0 | 2 | 0 | 0 | 0 | 0 | 0 | 0 | 0 | 0 | 0 | 0 |
| 156 | 0 | 0 | 1 | 0 | 0 | 38 | 0 | 0 | 0 | 1 | 0 | 0 | 0 | 0 | 0 | 1 | 0 | 0 | 0 | 0 |
| 157 | 0 | 0 | 1 | 0 | 0 | 34 | 0 | 0 | 1 | 1 | 0 | 1 | 0 | 0 | 0 | 2 | 1 | 0 | 0 | 0 |
| 158 | 0 | 0 | 0 | 0 | 0 | 43 | 0 | 0 | 0 | 0 | 0 | 1 | 0 | 0 | 0 | 2 | 2 | 0 | 0 | 0 |
| 159 | 0 | 0 | 0 | 0 | 0 | 42 | 0 | 0 | 0 | 0 | 0 | 1 | 0 | 1 | 1 | 2 | 1 | 0 | 0 | 0 |
| 160 | 1 | 0 | 0 | 0 | 0 | 50 | 0 | 0 | 0 | 1 | 0 | 0 | 0 | 0 | 0 | 2 | 0 | 0 | 0 | 0 |
| 161 | 2 | 0 | 0 | 1 | 1 | 40 | 0 | 0 | 0 | 0 | 0 | 1 | 0 | 0 | 1 | 1 | 0 | 1 | 0 | 0 |
| 162 | 1 | 0 | 1 | 2 | 0 | 44 | 1 | 0 | 0 | 0 | 0 | 1 | 0 | 0 | 0 | 1 | 0 | 0 | 0 | 1 |
| 163 | 2 | 0 | 1 | 1 | 0 | 59 | 0 | 0 | 0 | 0 | 0 | 0 | 0 | 0 | 0 | 1 | 0 | 0 | 0 | 1 |
| 164 | 1 | 0 | 0 | 2 | 1 | 39 | 0 | 0 | 0 | 0 | 0 | 0 | 0 | 0 | 0 | 2 | 0 | 0 | 0 | 0 |
| 165 | 4 | 0 | 3 | 1 | 0 | 54 | 1 | 0 | 1 | 0 | 0 | 0 | 0 | 0 | 1 | 1 | 0 | 0 | 0 | 0 |
| 166 | 1 | 0 | 1 | 1 | 0 | 51 | 0 | 0 | 2 | 0 | 1 | 0 | 0 | 0 | 0 | 1 | 1 | 1 | 1 | 0 |
| 167 | 0 | 0 | 1 | 0 | 0 | 52 | 0 | 0 | 1 | 0 | 0 | 0 | 1 | 1 | 0 | 2 | 0 | 0 | 0 | 0 |
| 168 | 1 | 0 | 3 | 1 | 1 | 46 | 0 | 0 | 2 | 1 | 0 | 0 | 0 | 0 | 0 | 2 | 1 | 2 | 0 | 0 |
| 169 | 1 | 0 | 1 | 2 | 0 | 62 | 0 | 0 | 1 | 0 | 0 | 0 | 0 | 1 | 0 | 2 | 0 | 0 | 0 | 0 |
| 170 | 1 | 0 | 0 | 0 | 0 | 50 | 2 | 0 | 1 | 2 | 0 | 0 | 0 | 0 | 2 | 3 | 3 | 1 | 0 | 1 |
| 171 | 0 | 0 | 2 | 3 | 0 | 44 | 0 | 1 | 2 | 1 | 1 | 2 | 0 | 3 | 4 | 3 | 0 | 0 | 0 | 1 |
| 172 | 2 | 2 | 0 | 0 | 0 | 67 | 1 | 0 | 2 | 1 | 0 | 3 | 0 | 1 | 1 | 4 | 2 | 1 | 0 | 0 |
| 173 | 1 | 0 | 2 | 0 | 2 | 58 | 0 | 0 | 0 | 0 | 0 | 0 | 0 | 1 | 1 | 5 | 0 | 0 | 0 | 1 |
| 174 | 0 | 1 | 3 | 2 | 0 | 55 | 0 | 1 | 1 | 1 | 0 | 3 | 0 | 0 | 0 | 4 | 1 | 0 | 1 | 1 |
| 175 | 2 | 0 | 0 | 1 | 0 | 67 | 0 | 0 | 1 | 2 | 0 | 2 | 0 | 1 | 1 | 6 | 0 | 0 | 0 | 1 |
| 176 | 6 | 0 | 6 | 3 | 1 | 60 | 4 | 1 | 0 | 1 | 0 | 4 | 0 | 1 | 0 | 7 | 1 | 2 | 0 | 0 |
| 177 | 6 | 0 | 4 | 2 | 1 | 50 | 1 | 0 | 0 | 0 | 0 | 2 | 0 | 1 | 1 | 6 | 1 | 0 | 0 | 1 |
| 178 | 5 | 0 | 3 | 1 | 0 | 56 | 3 | 0 | 1 | 0 | 1 | 3 | 0 | 4 | 1 | 5 | 1 | 2 | 0 | 3 |
| 179 | 7 | 2 | 2 | 2 | 2 | 46 | 2 | 0 | 3 | 1 | 0 | 3 | 0 | 1 | 1 | 7 | 1 | 0 | 0 | 3 |
| 180 | 0 | 0 | 0 | 0 | 0 | 3 | 1 | 0 | 0 | 0 | 0 | 0 | 0 | 0 | 0 | 0 | 0 | 0 | 0 | 0 |

Table S5: Distribution of psi angle for 20 amino acids.

| Psi Angle | A | C | D | E | F | G | H | I | K | L | M | N | P | Q | R | S | T | V | W | Y |
| --- | --- | --- | --- | --- | --- | --- | --- | --- | --- | --- | --- | --- | --- | --- | --- | --- | --- | --- | --- | --- |
| -180 | 0 | 0 | 2 | 0 | 0 | 7 | 1 | 0 | 2 | 0 | 1 | 0 | 1 | 1 | 0 | 1 | 5 | 0 | 0 | 3 |
| -179 | 18 | 14 | 61 | 14 | 10 | 231 | 6 | 4 | 8 | 14 | 8 | 43 | 21 | 8 | 19 | 63 | 65 | 13 | 4 | 7 |
| -178 | 15 | 12 | 70 | 13 | 10 | 212 | 7 | 8 | 11 | 12 | 2 | 39 | 21 | 10 | 9 | 44 | 53 | 14 | 5 | 11 |
| -177 | 16 | 9 | 61 | 12 | 9 | 204 | 9 | 6 | 5 | 6 | 4 | 35 | 16 | 5 | 8 | 48 | 56 | 10 | 3 | 7 |
| -176 | 6 | 6 | 60 | 11 | 7 | 187 | 6 | 3 | 16 | 11 | 0 | 32 | 14 | 5 | 11 | 46 | 52 | 5 | 3 | 6 |
| -175 | 13 | 7 | 69 | 9 | 8 | 209 | 11 | 3 | 4 | 8 | 2 | 40 | 5 | 6 | 9 | 32 | 42 | 3 | 7 | 4 |
| -174 | 8 | 8 | 45 | 3 | 5 | 201 | 11 | 4 | 6 | 5 | 3 | 34 | 8 | 6 | 4 | 30 | 37 | 4 | 1 | 7 |
| -173 | 16 | 3 | 45 | 3 | 3 | 212 | 10 | 1 | 7 | 7 | 4 | 30 | 11 | 8 | 5 | 34 | 44 | 4 | 1 | 3 |
| -172 | 7 | 6 | 41 | 3 | 4 | 187 | 2 | 3 | 2 | 5 | 3 | 20 | 7 | 3 | 8 | 28 | 38 | 8 | 1 | 6 |
| -171 | 13 | 3 | 44 | 5 | 5 | 170 | 1 | 1 | 7 | 3 | 2 | 24 | 7 | 6 | 7 | 29 | 26 | 4 | 0 | 3 |
| -170 | 10 | 4 | 25 | 3 | 4 | 197 | 3 | 0 | 4 | 5 | 1 | 18 | 5 | 2 | 4 | 24 | 22 | 0 | 0 | 6 |
| -169 | 1 | 3 | 20 | 3 | 1 | 186 | 5 | 2 | 0 | 7 | 2 | 12 | 3 | 2 | 3 | 18 | 25 | 6 | 1 | 2 |
| -168 | 2 | 5 | 23 | 4 | 0 | 189 | 0 | 1 | 2 | 3 | 2 | 17 | 6 | 4 | 5 | 20 | 20 | 5 | 3 | 2 |
| -167 | 8 | 0 | 20 | 1 | 2 | 185 | 5 | 0 | 1 | 3 | 1 | 17 | 3 | 2 | 1 | 14 | 19 | 4 | 0 | 4 |
| -166 | 4 | 2 | 14 | 3 | 1 | 179 | 4 | 0 | 4 | 3 | 0 | 5 | 6 | 0 | 2 | 14 | 12 | 4 | 3 | 0 |
| -165 | 3 | 0 | 24 | 2 | 3 | 156 | 5 | 1 | 3 | 1 | 1 | 13 | 1 | 1 | 1 | 11 | 8 | 1 | 0 | 2 |
| -164 | 8 | 1 | 16 | 3 | 3 | 147 | 3 | 0 | 6 | 2 | 0 | 5 | 5 | 4 | 3 | 13 | 12 | 0 | 1 | 1 |
| -163 | 0 | 2 | 14 | 0 | 2 | 153 | 2 | 2 | 0 | 2 | 1 | 15 | 2 | 1 | 2 | 11 | 9 | 5 | 0 | 2 |
| -162 | 1 | 1 | 12 | 1 | 2 | 145 | 2 | 2 | 0 | 2 | 0 | 4 | 1 | 3 | 0 | 12 | 8 | 1 | 2 | 2 |
| -161 | 3 | 1 | 18 | 2 | 1 | 141 | 2 | 1 | 2 | 1 | 1 | 13 | 1 | 3 | 1 | 9 | 7 | 1 | 0 | 0 |
| -160 | 3 | 1 | 6 | 3 | 4 | 132 | 1 | 2 | 2 | 2 | 2 | 11 | 0 | 0 | 4 | 9 | 11 | 1 | 0 | 2 |
| -159 | 2 | 1 | 14 | 5 | 2 | 117 | 2 | 1 | 1 | 2 | 0 | 12 | 2 | 0 | 1 | 3 | 4 | 1 | 1 | 0 |
| -158 | 2 | 0 | 10 | 1 | 2 | 156 | 2 | 1 | 3 | 1 | 1 | 7 | 0 | 0 | 1 | 11 | 6 | 1 | 1 | 1 |
| -157 | 2 | 0 | 3 | 0 | 1 | 114 | 0 | 0 | 5 | 1 | 2 | 4 | 1 | 1 | 1 | 4 | 6 | 1 | 0 | 2 |
| -156 | 5 | 0 | 5 | 3 | 0 | 137 | 0 | 0 | 3 | 0 | 1 | 0 | 0 | 2 | 2 | 3 | 4 | 0 | 0 | 2 |
| -155 | 4 | 2 | 3 | 1 | 0 | 129 | 2 | 1 | 2 | 1 | 0 | 5 | 3 | 1 | 1 | 8 | 3 | 0 | 0 | 0 |
| -154 | 4 | 2 | 3 | 4 | 0 | 108 | 1 | 0 | 1 | 0 | 0 | 4 | 0 | 0 | 1 | 8 | 1 | 1 | 0 | 3 |
| -153 | 3 | 0 | 3 | 0 | 1 | 100 | 1 | 0 | 0 | 1 | 0 | 3 | 0 | 1 | 1 | 7 | 1 | 0 | 1 | 0 |
| -152 | 2 | 0 | 3 | 2 | 0 | 99 | 2 | 0 | 1 | 0 | 0 | 4 | 1 | 0 | 2 | 3 | 1 | 2 | 1 | 0 |
| -151 | 2 | 1 | 4 | 0 | 1 | 92 | 1 | 0 | 0 | 5 | 0 | 6 | 1 | 1 | 0 | 5 | 3 | 0 | 0 | 0 |
| -150 | 3 | 0 | 1 | 2 | 0 | 99 | 0 | 0 | 4 | 1 | 1 | 2 | 1 | 2 | 3 | 2 | 6 | 0 | 0 | 2 |
| -149 | 2 | 0 | 3 | 2 | 1 | 87 | 1 | 1 | 1 | 1 | 1 | 2 | 1 | 1 | 0 | 2 | 2 | 0 | 2 | 1 |
| -148 | 8 | 1 | 6 | 1 | 1 | 73 | 0 | 0 | 1 | 1 | 1 | 0 | 1 | 5 | 1 | 3 | 3 | 1 | 0 | 1 |
| -147 | 3 | 0 | 3 | 2 | 1 | 101 | 2 | 0 | 1 | 1 | 1 | 0 | 1 | 2 | 0 | 7 | 1 | 0 | 1 | 0 |
| -146 | 0 | 0 | 1 | 1 | 0 | 82 | 2 | 0 | 0 | 0 | 0 | 1 | 0 | 1 | 2 | 4 | 1 | 3 | 0 | 0 |
| -145 | 0 | 1 | 1 | 3 | 1 | 76 | 0 | 0 | 2 | 1 | 1 | 0 | 1 | 0 | 3 | 4 | 0 | 0 | 0 | 1 |
| -144 | 2 | 0 | 3 | 2 | 0 | 74 | 0 | 0 | 1 | 0 | 0 | 3 | 2 | 0 | 0 | 3 | 1 | 0 | 0 | 3 |
| -143 | 4 | 1 | 2 | 0 | 1 | 74 | 0 | 0 | 2 | 0 | 0 | 2 | 1 | 0 | 1 | 5 | 0 | 0 | 1 | 1 |
| -142 | 3 | 0 | 0 | 4 | 0 | 63 | 0 | 0 | 4 | 4 | 1 | 1 | 0 | 1 | 0 | 3 | 1 | 1 | 0 | 0 |
| -141 | 3 | 0 | 1 | 2 | 1 | 63 | 1 | 0 | 4 | 0 | 0 | 0 | 0 | 1 | 3 | 4 | 0 | 1 | 1 | 0 |
| -140 | 0 | 2 | 2 | 2 | 1 | 67 | 0 | 0 | 1 | 1 | 0 | 1 | 2 | 1 | 2 | 4 | 1 | 1 | 0 | 1 |
| -139 | 0 | 2 | 5 | 0 | 1 | 51 | 1 | 1 | 1 | 3 | 0 | 2 | 0 | 2 | 2 | 4 | 2 | 0 | 1 | 0 |
| -138 | 3 | 0 | 4 | 2 | 0 | 58 | 1 | 1 | 2 | 4 | 0 | 3 | 1 | 1 | 1 | 5 | 0 | 0 | 0 | 0 |
| -137 | 3 | 1 | 1 | 0 | 0 | 58 | 0 | 0 | 3 | 2 | 0 | 0 | 0 | 0 | 3 | 3 | 2 | 0 | 0 | 1 |
| -136 | 3 | 1 | 4 | 3 | 1 | 71 | 0 | 0 | 2 | 0 | 0 | 1 | 1 | 0 | 3 | 3 | 1 | 1 | 0 | 1 |
| -135 | 1 | 0 | 1 | 0 | 0 | 57 | 0 | 0 | 4 | 2 | 1 | 4 | 1 | 1 | 2 | 3 | 1 | 0 | 1 | 1 |
| -134 | 0 | 0 | 5 | 1 | 2 | 71 | 0 | 0 | 3 | 1 | 0 | 1 | 1 | 0 | 1 | 3 | 1 | 0 | 0 | 2 |
| -133 | 3 | 1 | 4 | 0 | 1 | 73 | 4 | 0 | 1 | 0 | 0 | 3 | 0 | 1 | 2 | 1 | 0 | 1 | 0 | 1 |
| -132 | 2 | 1 | 3 | 2 | 0 | 68 | 0 | 1 | 3 | 2 | 0 | 3 | 1 | 2 | 1 | 1 | 0 | 0 | 1 | 0 |
| -131 | 6 | 0 | 1 | 1 | 1 | 66 | 0 | 0 | 4 | 1 | 0 | 0 | 1 | 0 | 0 | 2 | 0 | 0 | 1 | 0 |
| -130 | 4 | 0 | 4 | 2 | 2 | 63 | 0 | 0 | 2 | 3 | 0 | 1 | 0 | 1 | 1 | 2 | 1 | 0 | 1 | 1 |
| -129 | 2 | 0 | 3 | 2 | 0 | 68 | 3 | 0 | 1 | 3 | 0 | 1 | 1 | 1 | 2 | 3 | 3 | 0 | 1 | 1 |
| -128 | 3 | 0 | 7 | 2 | 0 | 70 | 0 | 2 | 2 | 0 | 1 | 4 | 1 | 2 | 3 | 7 | 1 | 1 | 0 | 2 |
| -127 | 5 | 0 | 6 | 3 | 1 | 70 | 0 | 0 | 4 | 0 | 0 | 1 | 0 | 1 | 3 | 2 | 2 | 1 | 0 | 1 |
| -126 | 6 | 0 | 4 | 4 | 2 | 57 | 1 | 0 | 4 | 3 | 1 | 3 | 0 | 1 | 0 | 3 | 2 | 0 | 0 | 1 |
| -125 | 1 | 1 | 8 | 2 | 1 | 59 | 0 | 0 | 3 | 0 | 1 | 1 | 0 | 2 | 1 | 5 | 2 | 0 | 0 | 1 |
| -124 | 4 | 0 | 5 | 1 | 0 | 56 | 2 | 0 | 0 | 2 | 0 | 6 | 2 | 1 | 4 | 2 | 2 | 2 | 0 | 2 |
| -123 | 6 | 1 | 3 | 2 | 0 | 49 | 1 | 1 | 1 | 3 | 1 | 2 | 2 | 0 | 2 | 5 | 0 | 0 | 0 | 0 |
| -122 | 4 | 0 | 2 | 3 | 1 | 58 | 3 | 0 | 3 | 1 | 0 | 2 | 1 | 1 | 1 | 1 | 1 | 0 | 0 | 1 |
| -121 | 2 | 0 | 2 | 3 | 0 | 38 | 1 | 0 | 2 | 2 | 1 | 2 | 0 | 0 | 0 | 2 | 1 | 1 | 1 | 1 |
| -120 | 6 | 0 | 3 | 3 | 0 | 29 | 0 | 0 | 4 | 2 | 0 | 2 | 0 | 0 | 0 | 3 | 1 | 0 | 0 | 1 |
| -119 | 4 | 1 | 2 | 5 | 1 | 35 | 0 | 0 | 0 | 2 | 1 | 1 | 0 | 3 | 1 | 3 | 1 | 1 | 0 | 0 |
| -118 | 4 | 0 | 3 | 3 | 0 | 31 | 0 | 1 | 5 | 1 | 0 | 2 | 0 | 3 | 1 | 6 | 0 | 0 | 0 | 2 |
| -117 | 2 | 1 | 3 | 0 | 0 | 27 | 2 | 0 | 2 | 2 | 0 | 1 | 2 | 1 | 2 | 0 | 3 | 1 | 0 | 1 |
| -116 | 6 | 1 | 3 | 4 | 0 | 21 | 0 | 0 | 2 | 2 | 0 | 1 | 0 | 1 | 0 | 5 | 0 | 1 | 0 | 0 |
| -115 | 1 | 1 | 1 | 3 | 1 | 20 | 0 | 0 | 2 | 0 | 0 | 3 | 1 | 1 | 1 | 2 | 1 | 0 | 0 | 0 |
| -114 | 6 | 0 | 3 | 1 | 1 | 13 | 2 | 0 | 2 | 2 | 0 | 1 | 1 | 0 | 1 | 4 | 1 | 2 | 0 | 0 |
| -113 | 0 | 0 | 7 | 2 | 1 | 18 | 0 | 0 | 1 | 0 | 0 | 0 | 0 | 0 | 3 | 4 | 2 | 1 | 1 | 1 |
| -112 | 1 | 1 | 3 | 2 | 0 | 16 | 2 | 0 | 1 | 1 | 0 | 1 | 0 | 1 | 3 | 1 | 0 | 0 | 0 | 2 |
| -111 | 4 | 0 | 2 | 1 | 0 | 18 | 1 | 0 | 1 | 0 | 1 | 1 | 0 | 1 | 0 | 3 | 3 | 0 | 0 | 0 |
| -110 | 5 | 0 | 1 | 0 | 0 | 20 | 2 | 0 | 2 | 0 | 1 | 2 | 1 | 0 | 3 | 2 | 3 | 0 | 0 | 0 |
| -109 | 1 | 0 | 3 | 1 | 0 | 13 | 0 | 0 | 2 | 0 | 0 | 1 | 0 | 1 | 0 | 0 | 4 | 0 | 0 | 0 |
| -108 | 0 | 0 | 2 | 3 | 0 | 12 | 0 | 0 | 2 | 1 | 0 | 2 | 3 | 0 | 2 | 3 | 0 | 1 | 0 | 0 |
| -107 | 1 | 0 | 2 | 0 | 0 | 10 | 0 | 0 | 3 | 0 | 1 | 2 | 1 | 1 | 1 | 0 | 1 | 1 | 1 | 0 |
| -106 | 0 | 0 | 2 | 1 | 0 | 16 | 1 | 0 | 7 | 0 | 0 | 0 | 1 | 0 | 3 | 3 | 1 | 0 | 1 | 2 |
| -105 | 0 | 0 | 0 | 1 | 0 | 7 | 1 | 0 | 3 | 2 | 0 | 2 | 1 | 6 | 1 | 1 | 2 | 0 | 2 | 1 |
| -104 | 1 | 0 | 2 | 2 | 0 | 7 | 1 | 0 | 0 | 1 | 0 | 2 | 0 | 0 | 0 | 0 | 4 | 0 | 1 | 0 |
| -103 | 0 | 0 | 6 | 4 | 0 | 12 | 2 | 0 | 2 | 0 | 0 | 0 | 2 | 2 | 0 | 0 | 2 | 0 | 1 | 0 |
| -102 | 2 | 1 | 5 | 2 | 2 | 10 | 1 | 0 | 3 | 0 | 0 | 2 | 0 | 1 | 0 | 1 | 1 | 1 | 0 | 0 |
| -101 | 2 | 0 | 2 | 0 | 1 | 8 | 0 | 0 | 0 | 2 | 0 | 3 | 1 | 0 | 1 | 1 | 2 | 1 | 0 | 0 |
| -100 | 2 | 2 | 0 | 0 | 1 | 6 | 0 | 0 | 0 | 1 | 0 | 1 | 1 | 0 | 1 | 3 | 0 | 0 | 0 | 0 |
| -99 | 3 | 0 | 2 | 2 | 1 | 4 | 0 | 3 | 1 | 1 | 0 | 2 | 0 | 1 | 4 | 0 | 0 | 0 | 0 | 2 |
| -98 | 2 | 0 | 2 | 1 | 2 | 6 | 0 | 1 | 0 | 0 | 0 | 2 | 1 | 2 | 3 | 1 | 1 | 0 | 0 | 0 |
| -97 | 2 | 0 | 6 | 1 | 0 | 5 | 0 | 1 | 4 | 1 | 0 | 3 | 0 | 1 | 2 | 1 | 5 | 1 | 0 | 2 |
| -96 | 2 | 0 | 2 | 0 | 2 | 9 | 3 | 0 | 7 | 0 | 0 | 2 | 0 | 0 | 0 | 2 | 2 | 0 | 0 | 0 |
| -95 | 5 | 0 | 2 | 1 | 1 | 6 | 0 | 0 | 0 | 1 | 0 | 4 | 3 | 1 | 1 | 3 | 4 | 0 | 0 | 1 |
| -94 | 4 | 1 | 2 | 0 | 1 | 8 | 1 | 0 | 0 | 1 | 0 | 2 | 0 | 4 | 0 | 1 | 1 | 0 | 0 | 1 |
| -93 | 6 | 0 | 0 | 1 | 0 | 4 | 0 | 1 | 4 | 0 | 0 | 2 | 0 | 0 | 2 | 0 | 1 | 0 | 1 | 1 |
| -92 | 2 | 0 | 4 | 3 | 0 | 9 | 0 | 0 | 3 | 0 | 0 | 1 | 1 | 2 | 0 | 3 | 1 | 1 | 2 | 2 |
| -91 | 2 | 1 | 3 | 2 | 1 | 7 | 0 | 1 | 4 | 0 | 1 | 2 | 0 | 0 | 2 | 4 | 4 | 1 | 1 | 0 |
| -90 | 2 | 0 | 4 | 3 | 0 | 9 | 0 | 0 | 0 | 1 | 2 | 1 | 1 | 1 | 4 | 2 | 1 | 0 | 0 | 1 |
| -89 | 0 | 0 | 2 | 4 | 0 | 10 | 0 | 2 | 0 | 4 | 0 | 0 | 1 | 0 | 1 | 3 | 3 | 0 | 0 | 0 |
| -88 | 0 | 0 | 1 | 0 | 2 | 4 | 1 | 0 | 3 | 0 | 0 | 1 | 2 | 0 | 5 | 2 | 4 | 0 | 1 | 1 |
| -87 | 3 | 0 | 2 | 4 | 0 | 10 | 0 | 2 | 2 | 1 | 3 | 2 | 2 | 2 | 1 | 4 | 2 | 2 | 0 | 3 |
| -86 | 3 | 0 | 5 | 2 | 1 | 7 | 0 | 5 | 1 | 2 | 0 | 2 | 1 | 1 | 0 | 1 | 1 | 2 | 2 | 0 |
| -85 | 3 | 0 | 3 | 1 | 3 | 9 | 3 | 0 | 7 | 1 | 0 | 0 | 0 | 2 | 0 | 4 | 3 | 2 | 0 | 1 |
| -84 | 2 | 0 | 4 | 2 | 1 | 9 | 1 | 2 | 0 | 2 | 1 | 3 | 2 | 1 | 1 | 1 | 3 | 1 | 0 | 2 |
| -83 | 2 | 1 | 6 | 3 | 2 | 10 | 4 | 0 | 3 | 3 | 0 | 3 | 0 | 2 | 4 | 0 | 4 | 3 | 2 | 1 |
| -82 | 0 | 1 | 5 | 3 | 0 | 8 | 1 | 0 | 3 | 3 | 0 | 2 | 0 | 2 | 3 | 3 | 4 | 2 | 0 | 3 |
| -81 | 1 | 0 | 3 | 2 | 3 | 10 | 1 | 0 | 2 | 2 | 0 | 1 | 0 | 0 | 2 | 2 | 5 | 1 | 0 | 2 |
| -80 | 3 | 0 | 2 | 4 | 0 | 6 | 2 | 1 | 1 | 3 | 1 | 3 | 2 | 2 | 2 | 7 | 3 | 0 | 0 | 1 |
| -79 | 4 | 1 | 2 | 4 | 3 | 11 | 1 | 3 | 3 | 1 | 2 | 2 | 0 | 3 | 4 | 7 | 7 | 0 | 1 | 1 |
| -78 | 8 | 3 | 3 | 6 | 4 | 8 | 2 | 6 | 3 | 4 | 1 | 3 | 0 | 2 | 3 | 5 | 5 | 5 | 1 | 2 |
| -77 | 8 | 1 | 6 | 5 | 1 | 13 | 1 | 1 | 3 | 3 | 0 | 4 | 2 | 2 | 1 | 5 | 7 | 2 | 0 | 4 |
| -76 | 3 | 3 | 3 | 5 | 1 | 7 | 2 | 1 | 3 | 5 | 0 | 2 | 1 | 2 | 2 | 4 | 5 | 2 | 0 | 1 |
| -75 | 2 | 0 | 1 | 10 | 4 | 8 | 2 | 5 | 5 | 3 | 0 | 1 | 2 | 2 | 4 | 4 | 5 | 6 | 1 | 1 |
| -74 | 4 | 0 | 2 | 5 | 4 | 11 | 2 | 5 | 3 | 2 | 1 | 7 | 1 | 2 | 6 | 8 | 5 | 4 | 3 | 2 |
| -73 | 10 | 1 | 8 | 11 | 4 | 10 | 2 | 1 | 7 | 5 | 0 | 2 | 1 | 3 | 5 | 5 | 7 | 5 | 1 | 4 |
| -72 | 8 | 2 | 5 | 13 | 6 | 4 | 2 | 2 | 3 | 8 | 2 | 2 | 3 | 0 | 4 | 2 | 7 | 6 | 1 | 0 |
| -71 | 6 | 2 | 4 | 5 | 3 | 14 | 4 | 2 | 3 | 4 | 2 | 3 | 2 | 3 | 8 | 3 | 7 | 1 | 1 | 4 |
| -70 | 12 | 1 | 8 | 9 | 1 | 10 | 2 | 9 | 5 | 7 | 2 | 2 | 6 | 5 | 8 | 8 | 5 | 7 | 2 | 6 |
| -69 | 6 | 2 | 9 | 18 | 6 | 12 | 5 | 5 | 9 | 11 | 2 | 5 | 2 | 5 | 5 | 3 | 4 | 4 | 1 | 3 |
| -68 | 10 | 3 | 5 | 14 | 8 | 17 | 3 | 6 | 8 | 12 | 0 | 6 | 3 | 6 | 5 | 7 | 8 | 7 | 1 | 1 |
| -67 | 15 | 2 | 2 | 13 | 3 | 14 | 4 | 8 | 9 | 15 | 1 | 5 | 6 | 3 | 8 | 6 | 7 | 8 | 1 | 4 |
| -66 | 13 | 2 | 11 | 22 | 6 | 17 | 2 | 13 | 15 | 14 | 4 | 3 | 1 | 8 | 10 | 10 | 7 | 16 | 3 | 10 |
| -65 | 13 | 2 | 15 | 17 | 8 | 17 | 4 | 8 | 16 | 23 | 3 | 9 | 10 | 6 | 12 | 8 | 10 | 13 | 4 | 7 |
| -64 | 15 | 2 | 5 | 20 | 18 | 14 | 6 | 14 | 16 | 18 | 5 | 9 | 8 | 14 | 16 | 10 | 8 | 16 | 1 | 7 |
| -63 | 13 | 1 | 14 | 24 | 12 | 27 | 5 | 15 | 17 | 33 | 5 | 6 | 4 | 15 | 11 | 17 | 16 | 15 | 5 | 11 |
| -62 | 29 | 0 | 28 | 26 | 12 | 14 | 12 | 16 | 23 | 28 | 4 | 9 | 7 | 12 | 24 | 16 | 18 | 15 | 5 | 8 |
| -61 | 24 | 3 | 14 | 24 | 16 | 24 | 8 | 26 | 26 | 38 | 5 | 10 | 16 | 13 | 18 | 18 | 21 | 38 | 9 | 17 |
| -60 | 42 | 7 | 23 | 31 | 25 | 24 | 15 | 33 | 23 | 42 | 3 | 14 | 3 | 12 | 20 | 10 | 12 | 30 | 17 | 22 |
| -59 | 34 | 8 | 27 | 34 | 26 | 26 | 15 | 36 | 34 | 46 | 3 | 10 | 7 | 19 | 33 | 28 | 15 | 56 | 8 | 22 |
| -58 | 46 | 1 | 27 | 49 | 37 | 39 | 16 | 39 | 43 | 68 | 6 | 17 | 16 | 28 | 43 | 24 | 20 | 52 | 14 | 36 |
| -57 | 46 | 7 | 27 | 64 | 36 | 43 | 16 | 46 | 59 | 75 | 13 | 15 | 15 | 27 | 40 | 32 | 37 | 57 | 16 | 33 |
| -56 | 64 | 8 | 48 | 66 | 54 | 31 | 18 | 73 | 56 | 91 | 14 | 17 | 15 | 37 | 39 | 26 | 48 | 76 | 19 | 49 |
| -55 | 85 | 7 | 43 | 90 | 84 | 45 | 33 | 93 | 74 | 102 | 21 | 21 | 22 | 36 | 66 | 32 | 52 | 91 | 23 | 73 |
| -54 | 103 | 12 | 65 | 106 | 101 | 47 | 29 | 103 | 103 | 160 | 29 | 20 | 35 | 50 | 89 | 49 | 62 | 115 | 34 | 94 |
| -53 | 139 | 16 | 69 | 140 | 125 | 62 | 45 | 135 | 126 | 180 | 29 | 35 | 29 | 58 | 94 | 65 | 92 | 158 | 35 | 108 |
| -52 | 179 | 33 | 91 | 172 | 179 | 78 | 52 | 171 | 158 | 251 | 32 | 47 | 47 | 86 | 127 | 82 | 116 | 197 | 39 | 130 |
| -51 | 256 | 32 | 126 | 195 | 224 | 93 | 59 | 252 | 198 | 334 | 42 | 67 | 37 | 89 | 190 | 117 | 122 | 270 | 48 | 195 |
| -50 | 310 | 40 | 132 | 257 | 275 | 113 | 89 | 319 | 269 | 398 | 53 | 63 | 59 | 131 | 202 | 145 | 184 | 283 | 69 | 212 |
| -49 | 411 | 52 | 191 | 355 | 294 | 131 | 120 | 437 | 289 | 567 | 93 | 78 | 84 | 185 | 226 | 152 | 237 | 407 | 77 | 246 |
| -48 | 505 | 65 | 237 | 449 | 342 | 159 | 114 | 512 | 360 | 714 | 114 | 119 | 73 | 218 | 329 | 203 | 289 | 523 | 116 | 297 |
| -47 | 678 | 63 | 295 | 583 | 399 | 186 | 167 | 613 | 441 | 795 | 108 | 145 | 111 | 272 | 411 | 278 | 353 | 633 | 113 | 331 |
| -46 | 854 | 98 | 336 | 727 | 375 | 243 | 166 | 734 | 580 | 942 | 167 | 164 | 132 | 313 | 472 | 308 | 372 | 743 | 138 | 328 |
| -45 | 1086 | 101 | 384 | 820 | 425 | 278 | 184 | 789 | 622 | 1090 | 185 | 211 | 124 | 404 | 565 | 389 | 463 | 782 | 153 | 339 |
| -44 | 1239 | 104 | 431 | 931 | 334 | 323 | 194 | 776 | 709 | 1119 | 191 | 232 | 132 | 477 | 626 | 400 | 428 | 839 | 136 | 291 |
| -43 | 1313 | 110 | 505 | 1016 | 347 | 341 | 205 | 785 | 774 | 1231 | 216 | 248 | 171 | 475 | 654 | 415 | 485 | 821 | 154 | 297 |
| -42 | 1478 | 108 | 570 | 1070 | 314 | 397 | 206 | 715 | 825 | 1321 | 211 | 279 | 168 | 534 | 659 | 445 | 490 | 789 | 124 | 280 |
| -41 | 1459 | 115 | 521 | 1106 | 295 | 385 | 183 | 671 | 837 | 1305 | 226 | 329 | 214 | 604 | 674 | 452 | 415 | 677 | 104 | 264 |
| -40 | 1441 | 120 | 509 | 1121 | 267 | 343 | 175 | 591 | 755 | 1300 | 257 | 294 | 231 | 529 | 645 | 396 | 387 | 632 | 116 | 232 |
| -39 | 1280 | 89 | 517 | 1026 | 258 | 350 | 151 | 450 | 729 | 1245 | 242 | 294 | 235 | 536 | 591 | 377 | 340 | 516 | 116 | 213 |
| -38 | 1185 | 97 | 481 | 949 | 249 | 302 | 143 | 458 | 681 | 1086 | 196 | 285 | 258 | 481 | 593 | 389 | 265 | 432 | 108 | 203 |
| -37 | 982 | 82 | 479 | 837 | 215 | 240 | 133 | 343 | 590 | 1012 | 206 | 289 | 260 | 419 | 539 | 368 | 264 | 403 | 95 | 190 |
| -36 | 855 | 81 | 453 | 781 | 184 | 233 | 130 | 280 | 549 | 881 | 181 | 247 | 286 | 408 | 497 | 343 | 237 | 362 | 99 | 167 |
| -35 | 734 | 47 | 356 | 735 | 178 | 225 | 123 | 237 | 487 | 852 | 144 | 245 | 264 | 394 | 359 | 330 | 217 | 307 | 91 | 131 |
| -34 | 641 | 48 | 349 | 603 | 177 | 193 | 109 | 247 | 401 | 706 | 127 | 221 | 296 | 315 | 376 | 300 | 233 | 260 | 82 | 140 |
| -33 | 515 | 59 | 321 | 542 | 133 | 166 | 96 | 206 | 378 | 588 | 119 | 185 | 296 | 238 | 299 | 278 | 165 | 240 | 70 | 115 |
| -32 | 459 | 39 | 258 | 519 | 137 | 148 | 80 | 148 | 332 | 533 | 98 | 183 | 237 | 230 | 325 | 272 | 175 | 191 | 62 | 100 |
| -31 | 418 | 37 | 252 | 455 | 119 | 127 | 95 | 149 | 308 | 466 | 84 | 173 | 225 | 210 | 228 | 254 | 159 | 148 | 59 | 128 |
| -30 | 382 | 37 | 216 | 364 | 132 | 141 | 68 | 135 | 303 | 389 | 72 | 150 | 247 | 197 | 229 | 264 | 164 | 185 | 54 | 105 |
| -29 | 365 | 39 | 222 | 350 | 100 | 134 | 82 | 124 | 205 | 366 | 76 | 123 | 246 | 153 | 196 | 214 | 148 | 134 | 65 | 96 |
| -28 | 352 | 31 | 170 | 277 | 80 | 136 | 71 | 107 | 207 | 343 | 63 | 114 | 226 | 153 | 173 | 248 | 140 | 146 | 51 | 97 |
| -27 | 315 | 29 | 180 | 289 | 92 | 170 | 67 | 113 | 229 | 296 | 69 | 117 | 256 | 129 | 177 | 199 | 143 | 123 | 50 | 78 |
| -26 | 254 | 28 | 161 | 266 | 84 | 119 | 52 | 90 | 222 | 275 | 47 | 94 | 234 | 139 | 170 | 215 | 118 | 113 | 41 | 74 |
| -25 | 286 | 24 | 154 | 255 | 78 | 133 | 70 | 82 | 187 | 243 | 51 | 111 | 232 | 124 | 157 | 212 | 141 | 104 | 41 | 80 |
| -24 | 276 | 25 | 145 | 244 | 87 | 139 | 43 | 96 | 182 | 240 | 47 | 92 | 224 | 107 | 172 | 200 | 122 | 105 | 27 | 68 |
| -23 | 236 | 17 | 153 | 225 | 82 | 131 | 65 | 73 | 185 | 223 | 49 | 90 | 228 | 105 | 128 | 230 | 132 | 100 | 40 | 83 |
| -22 | 230 | 31 | 136 | 229 | 64 | 145 | 59 | 62 | 180 | 253 | 49 | 89 | 216 | 98 | 123 | 214 | 139 | 89 | 36 | 60 |
| -21 | 206 | 22 | 152 | 218 | 70 | 157 | 52 | 57 | 159 | 210 | 29 | 73 | 195 | 91 | 133 | 217 | 141 | 83 | 29 | 59 |
| -20 | 219 | 25 | 120 | 218 | 68 | 139 | 55 | 54 | 161 | 208 | 35 | 97 | 188 | 102 | 113 | 244 | 152 | 103 | 28 | 53 |
| -19 | 192 | 32 | 135 | 201 | 63 | 163 | 42 | 59 | 164 | 185 | 38 | 96 | 186 | 91 | 126 | 209 | 141 | 73 | 39 | 57 |
| -18 | 216 | 27 | 137 | 217 | 67 | 172 | 46 | 63 | 165 | 197 | 37 | 86 | 168 | 97 | 118 | 219 | 148 | 80 | 29 | 40 |
| -17 | 175 | 17 | 113 | 161 | 61 | 169 | 62 | 62 | 144 | 188 | 34 | 104 | 163 | 82 | 108 | 228 | 134 | 70 | 20 | 42 |
| -16 | 177 | 29 | 138 | 170 | 62 | 207 | 47 | 55 | 161 | 166 | 38 | 84 | 155 | 81 | 111 | 226 | 150 | 58 | 31 | 51 |
| -15 | 171 | 26 | 133 | 151 | 68 | 225 | 45 | 61 | 143 | 171 | 35 | 87 | 136 | 101 | 105 | 214 | 144 | 67 | 27 | 44 |
| -14 | 151 | 20 | 115 | 178 | 48 | 200 | 47 | 56 | 147 | 191 | 29 | 100 | 135 | 84 | 113 | 202 | 142 | 64 | 29 | 49 |
| -13 | 143 | 33 | 132 | 154 | 59 | 221 | 58 | 50 | 127 | 158 | 34 | 72 | 117 | 84 | 101 | 195 | 144 | 58 | 21 | 53 |
| -12 | 141 | 22 | 133 | 159 | 70 | 214 | 50 | 41 | 128 | 165 | 27 | 79 | 109 | 87 | 87 | 169 | 136 | 68 | 24 | 48 |
| -11 | 140 | 20 | 121 | 120 | 60 | 248 | 46 | 51 | 117 | 156 | 30 | 73 | 121 | 61 | 96 | 194 | 115 | 79 | 27 | 42 |
| -10 | 120 | 26 | 132 | 136 | 69 | 228 | 55 | 44 | 108 | 164 | 25 | 90 | 91 | 62 | 72 | 155 | 149 | 60 | 26 | 52 |
| -9 | 126 | 22 | 102 | 100 | 52 | 239 | 52 | 37 | 116 | 164 | 23 | 90 | 76 | 75 | 70 | 145 | 133 | 46 | 29 | 52 |
| -8 | 131 | 15 | 133 | 119 | 69 | 240 | 49 | 43 | 110 | 145 | 29 | 84 | 87 | 60 | 75 | 152 | 130 | 55 | 26 | 55 |
| -7 | 115 | 18 | 122 | 138 | 59 | 241 | 34 | 30 | 99 | 138 | 23 | 100 | 81 | 60 | 106 | 147 | 126 | 50 | 18 | 62 |
| -6 | 93 | 23 | 122 | 94 | 52 | 290 | 37 | 39 | 97 | 134 | 31 | 96 | 78 | 59 | 70 | 144 | 132 | 45 | 21 | 47 |
| -5 | 113 | 22 | 127 | 100 | 55 | 254 | 50 | 32 | 94 | 149 | 25 | 99 | 63 | 61 | 79 | 134 | 127 | 46 | 20 | 53 |
| -4 | 99 | 24 | 121 | 101 | 53 | 283 | 49 | 35 | 82 | 138 | 30 | 100 | 60 | 69 | 75 | 133 | 134 | 49 | 14 | 49 |
| -3 | 91 | 17 | 153 | 84 | 51 | 285 | 43 | 38 | 86 | 118 | 24 | 102 | 53 | 64 | 76 | 118 | 111 | 37 | 22 | 50 |
| -2 | 101 | 23 | 123 | 108 | 60 | 290 | 42 | 31 | 81 | 135 | 19 | 87 | 48 | 63 | 64 | 114 | 105 | 37 | 21 | 50 |
| -1 | 79 | 16 | 122 | 84 | 52 | 273 | 44 | 31 | 95 | 138 | 22 | 109 | 49 | 63 | 71 | 110 | 85 | 42 | 16 | 59 |
| 0 | 127 | 32 | 301 | 158 | 94 | 570 | 69 | 55 | 161 | 226 | 32 | 211 | 81 | 139 | 122 | 170 | 170 | 60 | 25 | 75 |
| 1 | 76 | 13 | 120 | 79 | 46 | 280 | 38 | 26 | 79 | 111 | 24 | 92 | 29 | 61 | 70 | 105 | 88 | 34 | 18 | 49 |
| 2 | 84 | 15 | 124 | 72 | 51 | 281 | 32 | 36 | 68 | 77 | 12 | 105 | 37 | 54 | 57 | 89 | 96 | 38 | 12 | 54 |
| 3 | 64 | 15 | 149 | 69 | 57 | 307 | 42 | 24 | 66 | 106 | 19 | 114 | 31 | 44 | 46 | 64 | 85 | 27 | 12 | 58 |
| 4 | 57 | 12 | 113 | 63 | 50 | 282 | 45 | 23 | 77 | 90 | 26 | 120 | 27 | 51 | 63 | 54 | 81 | 29 | 21 | 39 |
| 5 | 37 | 15 | 133 | 71 | 42 | 289 | 48 | 24 | 79 | 75 | 16 | 118 | 23 | 43 | 48 | 78 | 77 | 25 | 17 | 56 |
| 6 | 61 | 13 | 122 | 54 | 61 | 288 | 40 | 19 | 57 | 78 | 16 | 104 | 16 | 42 | 43 | 62 | 66 | 26 | 12 | 44 |
| 7 | 58 | 14 | 136 | 63 | 58 | 282 | 33 | 14 | 66 | 84 | 14 | 136 | 21 | 45 | 47 | 70 | 69 | 33 | 16 | 43 |
| 8 | 52 | 9 | 118 | 43 | 48 | 293 | 23 | 33 | 61 | 87 | 16 | 124 | 25 | 39 | 51 | 57 | 74 | 22 | 15 | 45 |
| 9 | 38 | 12 | 117 | 48 | 29 | 297 | 29 | 22 | 49 | 68 | 11 | 105 | 17 | 37 | 43 | 53 | 54 | 24 | 12 | 38 |
| 10 | 44 | 20 | 106 | 46 | 43 | 250 | 39 | 24 | 43 | 61 | 20 | 90 | 17 | 31 | 36 | 50 | 45 | 27 | 6 | 34 |
| 11 | 31 | 8 | 109 | 53 | 43 | 282 | 33 | 15 | 46 | 74 | 12 | 117 | 24 | 36 | 34 | 58 | 65 | 21 | 14 | 47 |
| 12 | 31 | 14 | 121 | 34 | 37 | 269 | 28 | 22 | 28 | 52 | 14 | 124 | 15 | 29 | 40 | 43 | 57 | 22 | 8 | 43 |
| 13 | 25 | 8 | 94 | 25 | 47 | 259 | 41 | 28 | 44 | 52 | 17 | 105 | 14 | 31 | 34 | 38 | 49 | 28 | 8 | 39 |
| 14 | 27 | 9 | 106 | 36 | 43 | 244 | 19 | 14 | 41 | 48 | 9 | 114 | 18 | 29 | 42 | 39 | 55 | 18 | 11 | 37 |
| 15 | 45 | 12 | 107 | 37 | 30 | 222 | 26 | 21 | 32 | 43 | 17 | 91 | 10 | 37 | 47 | 30 | 46 | 24 | 13 | 34 |
| 16 | 28 | 9 | 107 | 27 | 58 | 246 | 29 | 15 | 35 | 38 | 11 | 115 | 12 | 33 | 38 | 36 | 30 | 13 | 11 | 28 |
| 17 | 25 | 15 | 85 | 40 | 36 | 233 | 39 | 18 | 31 | 32 | 11 | 92 | 3 | 25 | 38 | 33 | 40 | 19 | 10 | 25 |
| 18 | 25 | 9 | 76 | 23 | 38 | 219 | 46 | 20 | 32 | 39 | 11 | 98 | 7 | 17 | 23 | 22 | 33 | 19 | 10 | 30 |
| 19 | 24 | 10 | 76 | 36 | 38 | 182 | 30 | 10 | 33 | 26 | 10 | 103 | 8 | 26 | 25 | 27 | 36 | 25 | 6 | 26 |
| 20 | 22 | 5 | 72 | 24 | 20 | 183 | 29 | 14 | 28 | 34 | 9 | 87 | 10 | 23 | 25 | 21 | 25 | 12 | 10 | 34 |
| 21 | 16 | 9 | 59 | 19 | 26 | 197 | 30 | 15 | 21 | 35 | 7 | 86 | 8 | 22 | 22 | 23 | 24 | 19 | 4 | 25 |
| 22 | 26 | 2 | 55 | 25 | 23 | 201 | 32 | 11 | 28 | 47 | 12 | 66 | 9 | 22 | 22 | 29 | 22 | 12 | 4 | 22 |
| 23 | 21 | 10 | 55 | 12 | 18 | 179 | 20 | 13 | 32 | 28 | 9 | 77 | 8 | 17 | 23 | 20 | 21 | 17 | 5 | 22 |
| 24 | 14 | 4 | 64 | 24 | 21 | 200 | 31 | 9 | 21 | 30 | 10 | 75 | 6 | 24 | 22 | 22 | 19 | 18 | 5 | 29 |
| 25 | 18 | 11 | 51 | 22 | 25 | 163 | 18 | 10 | 29 | 27 | 8 | 92 | 1 | 18 | 24 | 17 | 10 | 12 | 4 | 13 |
| 26 | 27 | 7 | 52 | 12 | 24 | 174 | 19 | 10 | 27 | 25 | 5 | 53 | 2 | 12 | 25 | 18 | 16 | 15 | 6 | 12 |
| 27 | 23 | 10 | 45 | 25 | 21 | 136 | 15 | 11 | 27 | 20 | 11 | 81 | 6 | 9 | 18 | 21 | 16 | 10 | 7 | 15 |
| 28 | 20 | 5 | 41 | 28 | 24 | 143 | 14 | 1 | 24 | 27 | 10 | 73 | 9 | 15 | 26 | 17 | 22 | 5 | 3 | 13 |
| 29 | 15 | 12 | 64 | 23 | 21 | 140 | 21 | 3 | 22 | 21 | 3 | 73 | 3 | 13 | 21 | 17 | 12 | 12 | 3 | 17 |
| 30 | 23 | 7 | 54 | 22 | 17 | 123 | 17 | 8 | 22 | 17 | 5 | 80 | 4 | 15 | 28 | 17 | 13 | 5 | 4 | 15 |
| 31 | 12 | 7 | 45 | 12 | 11 | 134 | 13 | 1 | 22 | 15 | 3 | 88 | 4 | 20 | 24 | 23 | 12 | 7 | 7 | 13 |
| 32 | 17 | 3 | 51 | 23 | 12 | 104 | 16 | 3 | 34 | 18 | 7 | 71 | 4 | 17 | 17 | 23 | 9 | 5 | 2 | 9 |
| 33 | 18 | 7 | 47 | 19 | 19 | 108 | 18 | 4 | 27 | 19 | 6 | 60 | 3 | 13 | 15 | 17 | 10 | 8 | 0 | 16 |
| 34 | 23 | 3 | 35 | 24 | 13 | 91 | 17 | 6 | 26 | 14 | 5 | 74 | 1 | 13 | 25 | 12 | 10 | 9 | 2 | 8 |
| 35 | 16 | 6 | 67 | 23 | 8 | 97 | 21 | 4 | 18 | 17 | 3 | 80 | 8 | 14 | 22 | 28 | 6 | 1 | 1 | 18 |
| 36 | 17 | 6 | 45 | 21 | 17 | 97 | 22 | 2 | 31 | 15 | 7 | 77 | 3 | 17 | 28 | 20 | 9 | 4 | 6 | 13 |
| 37 | 19 | 4 | 56 | 20 | 16 | 76 | 20 | 4 | 24 | 17 | 4 | 91 | 2 | 20 | 17 | 28 | 12 | 6 | 2 | 10 |
| 38 | 20 | 3 | 52 | 22 | 16 | 82 | 20 | 1 | 36 | 13 | 3 | 70 | 3 | 18 | 24 | 27 | 10 | 6 | 8 | 14 |
| 39 | 21 | 10 | 41 | 21 | 18 | 62 | 18 | 3 | 27 | 19 | 2 | 90 | 5 | 26 | 20 | 16 | 4 | 4 | 1 | 5 |
| 40 | 19 | 4 | 49 | 16 | 10 | 60 | 24 | 5 | 24 | 9 | 5 | 105 | 1 | 20 | 19 | 14 | 8 | 3 | 3 | 9 |
| 41 | 21 | 9 | 54 | 21 | 14 | 55 | 16 | 1 | 30 | 13 | 5 | 103 | 5 | 11 | 19 | 12 | 10 | 4 | 1 | 6 |
| 42 | 20 | 8 | 64 | 22 | 10 | 58 | 16 | 2 | 28 | 20 | 5 | 96 | 4 | 18 | 18 | 20 | 8 | 1 | 1 | 14 |
| 43 | 22 | 7 | 56 | 28 | 10 | 44 | 17 | 1 | 26 | 12 | 6 | 99 | 6 | 12 | 18 | 20 | 6 | 5 | 4 | 21 |
| 44 | 13 | 3 | 63 | 24 | 10 | 40 | 22 | 3 | 27 | 11 | 6 | 86 | 1 | 11 | 20 | 19 | 7 | 4 | 2 | 10 |
| 45 | 18 | 4 | 49 | 27 | 8 | 45 | 24 | 0 | 14 | 9 | 7 | 81 | 6 | 10 | 15 | 22 | 6 | 6 | 3 | 10 |
| 46 | 20 | 4 | 36 | 27 | 8 | 29 | 22 | 3 | 33 | 9 | 0 | 80 | 8 | 23 | 24 | 16 | 3 | 1 | 2 | 7 |
| 47 | 14 | 4 | 38 | 14 | 9 | 31 | 11 | 2 | 34 | 15 | 3 | 60 | 8 | 12 | 16 | 11 | 4 | 9 | 2 | 6 |
| 48 | 20 | 4 | 42 | 19 | 13 | 27 | 12 | 1 | 23 | 13 | 5 | 72 | 6 | 21 | 9 | 18 | 5 | 7 | 4 | 7 |
| 49 | 11 | 3 | 41 | 9 | 9 | 27 | 15 | 3 | 19 | 9 | 2 | 57 | 9 | 17 | 23 | 22 | 3 | 3 | 2 | 10 |
| 50 | 16 | 2 | 44 | 13 | 8 | 23 | 15 | 6 | 22 | 15 | 4 | 66 | 12 | 15 | 26 | 13 | 9 | 4 | 2 | 9 |
| 51 | 24 | 2 | 42 | 15 | 13 | 22 | 15 | 4 | 24 | 11 | 2 | 54 | 12 | 12 | 21 | 15 | 3 | 3 | 1 | 11 |
| 52 | 14 | 5 | 35 | 22 | 11 | 24 | 16 | 1 | 24 | 13 | 1 | 70 | 14 | 20 | 20 | 8 | 6 | 6 | 4 | 9 |
| 53 | 12 | 1 | 38 | 21 | 8 | 25 | 10 | 2 | 16 | 5 | 2 | 45 | 10 | 13 | 19 | 12 | 2 | 4 | 2 | 2 |
| 54 | 12 | 2 | 38 | 16 | 10 | 17 | 15 | 2 | 24 | 10 | 5 | 53 | 8 | 14 | 15 | 16 | 4 | 2 | 0 | 10 |
| 55 | 21 | 4 | 29 | 12 | 8 | 21 | 15 | 2 | 14 | 9 | 3 | 44 | 7 | 11 | 11 | 12 | 3 | 3 | 1 | 7 |
| 56 | 6 | 5 | 34 | 11 | 9 | 10 | 6 | 3 | 22 | 11 | 1 | 46 | 10 | 12 | 14 | 12 | 4 | 2 | 2 | 9 |
| 57 | 13 | 3 | 34 | 13 | 12 | 20 | 10 | 4 | 10 | 11 | 3 | 40 | 17 | 14 | 23 | 10 | 5 | 1 | 5 | 12 |
| 58 | 13 | 5 | 33 | 12 | 12 | 16 | 11 | 6 | 21 | 13 | 6 | 40 | 13 | 9 | 10 | 15 | 6 | 4 | 1 | 3 |
| 59 | 22 | 6 | 27 | 14 | 7 | 18 | 14 | 0 | 21 | 21 | 3 | 37 | 13 | 11 | 11 | 19 | 6 | 4 | 5 | 9 |
| 60 | 23 | 4 | 29 | 20 | 10 | 10 | 12 | 1 | 18 | 8 | 1 | 41 | 12 | 13 | 14 | 9 | 7 | 4 | 3 | 8 |
| 61 | 12 | 7 | 34 | 12 | 16 | 18 | 11 | 1 | 19 | 9 | 3 | 39 | 9 | 5 | 21 | 14 | 7 | 1 | 0 | 12 |
| 62 | 18 | 5 | 34 | 10 | 11 | 17 | 8 | 1 | 18 | 13 | 7 | 32 | 14 | 7 | 14 | 13 | 9 | 6 | 4 | 9 |
| 63 | 23 | 4 | 30 | 12 | 14 | 14 | 12 | 2 | 16 | 16 | 5 | 34 | 18 | 11 | 13 | 6 | 11 | 6 | 4 | 8 |
| 64 | 25 | 2 | 28 | 17 | 13 | 12 | 18 | 4 | 10 | 14 | 4 | 29 | 11 | 14 | 17 | 12 | 10 | 4 | 3 | 11 |
| 65 | 19 | 4 | 30 | 11 | 12 | 11 | 6 | 3 | 8 | 22 | 3 | 41 | 20 | 15 | 8 | 12 | 9 | 4 | 2 | 10 |
| 66 | 28 | 4 | 30 | 10 | 12 | 5 | 13 | 0 | 13 | 13 | 3 | 49 | 20 | 11 | 12 | 16 | 6 | 3 | 3 | 8 |
| 67 | 17 | 3 | 39 | 13 | 15 | 7 | 11 | 5 | 16 | 5 | 3 | 31 | 16 | 17 | 17 | 13 | 4 | 4 | 3 | 8 |
| 68 | 24 | 4 | 41 | 10 | 14 | 7 | 24 | 0 | 11 | 13 | 3 | 32 | 18 | 6 | 12 | 12 | 4 | 2 | 4 | 12 |
| 69 | 18 | 5 | 37 | 11 | 11 | 7 | 12 | 4 | 15 | 8 | 4 | 27 | 14 | 13 | 17 | 16 | 7 | 5 | 4 | 12 |
| 70 | 27 | 6 | 36 | 11 | 10 | 15 | 11 | 4 | 14 | 18 | 3 | 25 | 9 | 8 | 15 | 16 | 11 | 6 | 3 | 11 |
| 71 | 21 | 2 | 21 | 14 | 17 | 8 | 13 | 3 | 15 | 16 | 4 | 36 | 12 | 9 | 17 | 9 | 8 | 3 | 3 | 21 |
| 72 | 19 | 1 | 35 | 13 | 19 | 7 | 7 | 2 | 7 | 18 | 2 | 36 | 8 | 14 | 18 | 11 | 12 | 4 | 4 | 12 |
| 73 | 18 | 2 | 46 | 19 | 12 | 9 | 8 | 9 | 13 | 16 | 2 | 37 | 14 | 7 | 12 | 15 | 15 | 10 | 5 | 18 |
| 74 | 16 | 5 | 34 | 12 | 19 | 7 | 6 | 5 | 15 | 18 | 7 | 30 | 9 | 10 | 20 | 10 | 6 | 5 | 3 | 8 |
| 75 | 10 | 11 | 39 | 14 | 18 | 9 | 14 | 10 | 8 | 17 | 2 | 37 | 5 | 12 | 18 | 9 | 6 | 6 | 4 | 10 |
| 76 | 15 | 5 | 45 | 6 | 15 | 2 | 13 | 4 | 10 | 13 | 3 | 31 | 13 | 11 | 9 | 9 | 4 | 9 | 3 | 7 |
| 77 | 18 | 5 | 42 | 10 | 15 | 6 | 10 | 5 | 13 | 14 | 3 | 29 | 7 | 9 | 4 | 9 | 7 | 7 | 2 | 17 |
| 78 | 17 | 4 | 34 | 8 | 18 | 8 | 6 | 7 | 10 | 22 | 6 | 32 | 4 | 9 | 7 | 7 | 7 | 3 | 3 | 14 |
| 79 | 19 | 8 | 42 | 14 | 20 | 8 | 14 | 9 | 17 | 23 | 2 | 30 | 7 | 15 | 14 | 14 | 8 | 15 | 3 | 14 |
| 80 | 16 | 5 | 48 | 15 | 9 | 6 | 9 | 5 | 14 | 17 | 6 | 27 | 6 | 5 | 15 | 5 | 7 | 4 | 3 | 8 |
| 81 | 23 | 8 | 44 | 17 | 16 | 6 | 15 | 6 | 12 | 22 | 5 | 32 | 5 | 9 | 13 | 9 | 8 | 13 | 6 | 5 |
| 82 | 18 | 3 | 46 | 15 | 10 | 2 | 8 | 8 | 10 | 18 | 3 | 33 | 7 | 9 | 12 | 8 | 8 | 13 | 3 | 17 |
| 83 | 17 | 7 | 47 | 11 | 15 | 5 | 9 | 10 | 16 | 21 | 3 | 33 | 5 | 10 | 8 | 8 | 3 | 12 | 1 | 7 |
| 84 | 9 | 6 | 33 | 15 | 12 | 3 | 6 | 17 | 18 | 15 | 6 | 35 | 6 | 5 | 19 | 11 | 5 | 11 | 3 | 6 |
| 85 | 23 | 3 | 38 | 10 | 15 | 7 | 15 | 17 | 14 | 17 | 7 | 23 | 8 | 10 | 19 | 9 | 2 | 12 | 2 | 9 |
| 86 | 12 | 4 | 41 | 19 | 10 | 4 | 14 | 14 | 15 | 17 | 5 | 23 | 12 | 11 | 17 | 16 | 6 | 18 | 7 | 9 |
| 87 | 15 | 5 | 43 | 11 | 10 | 3 | 10 | 13 | 22 | 13 | 3 | 40 | 4 | 10 | 11 | 6 | 7 | 11 | 7 | 7 |
| 88 | 20 | 7 | 40 | 19 | 21 | 7 | 13 | 12 | 24 | 27 | 5 | 29 | 3 | 14 | 12 | 13 | 5 | 19 | 6 | 14 |
| 89 | 12 | 8 | 60 | 18 | 22 | 10 | 11 | 16 | 16 | 33 | 8 | 30 | 5 | 10 | 13 | 7 | 6 | 28 | 3 | 10 |
| 90 | 22 | 3 | 63 | 23 | 23 | 13 | 13 | 15 | 12 | 33 | 2 | 42 | 4 | 11 | 16 | 12 | 13 | 32 | 2 | 7 |
| 91 | 23 | 6 | 68 | 19 | 24 | 8 | 18 | 17 | 16 | 23 | 4 | 39 | 6 | 8 | 17 | 20 | 11 | 24 | 6 | 8 |
| 92 | 14 | 8 | 73 | 12 | 19 | 5 | 12 | 22 | 19 | 28 | 4 | 43 | 3 | 7 | 22 | 10 | 6 | 22 | 5 | 14 |
| 93 | 13 | 9 | 61 | 17 | 15 | 8 | 10 | 26 | 18 | 28 | 7 | 51 | 3 | 10 | 14 | 24 | 9 | 36 | 4 | 13 |
| 94 | 13 | 12 | 86 | 18 | 19 | 8 | 16 | 26 | 15 | 30 | 6 | 43 | 2 | 15 | 12 | 15 | 16 | 31 | 3 | 12 |
| 95 | 12 | 7 | 82 | 26 | 23 | 7 | 14 | 31 | 15 | 28 | 4 | 43 | 2 | 9 | 15 | 14 | 19 | 32 | 4 | 6 |
| 96 | 17 | 11 | 84 | 24 | 25 | 5 | 12 | 44 | 23 | 26 | 7 | 40 | 10 | 15 | 26 | 15 | 9 | 46 | 11 | 16 |
| 97 | 22 | 10 | 87 | 23 | 16 | 11 | 16 | 31 | 29 | 40 | 4 | 43 | 12 | 9 | 14 | 14 | 13 | 51 | 3 | 16 |
| 98 | 24 | 13 | 90 | 24 | 21 | 5 | 17 | 32 | 24 | 41 | 14 | 56 | 3 | 19 | 23 | 23 | 20 | 42 | 7 | 21 |
| 99 | 24 | 13 | 116 | 22 | 24 | 12 | 14 | 42 | 23 | 37 | 5 | 71 | 4 | 21 | 21 | 24 | 10 | 51 | 8 | 31 |
| 100 | 22 | 9 | 109 | 27 | 28 | 7 | 10 | 39 | 24 | 55 | 6 | 73 | 11 | 16 | 20 | 21 | 18 | 60 | 8 | 14 |
| 101 | 27 | 8 | 125 | 30 | 28 | 4 | 18 | 50 | 41 | 65 | 13 | 63 | 6 | 15 | 25 | 23 | 22 | 70 | 9 | 28 |
| 102 | 21 | 14 | 136 | 22 | 43 | 17 | 21 | 71 | 26 | 49 | 10 | 60 | 7 | 19 | 17 | 34 | 16 | 78 | 11 | 22 |
| 103 | 23 | 14 | 122 | 38 | 33 | 8 | 13 | 76 | 43 | 81 | 13 | 95 | 11 | 19 | 23 | 24 | 23 | 77 | 16 | 22 |
| 104 | 32 | 10 | 118 | 31 | 43 | 15 | 14 | 62 | 40 | 81 | 9 | 70 | 9 | 26 | 24 | 26 | 32 | 72 | 11 | 30 |
| 105 | 29 | 14 | 153 | 40 | 42 | 11 | 24 | 102 | 34 | 73 | 11 | 75 | 11 | 11 | 29 | 21 | 31 | 93 | 15 | 34 |
| 106 | 35 | 21 | 141 | 48 | 39 | 8 | 33 | 98 | 30 | 95 | 16 | 68 | 8 | 28 | 27 | 30 | 31 | 100 | 18 | 37 |
| 107 | 27 | 11 | 147 | 46 | 55 | 10 | 30 | 116 | 40 | 105 | 17 | 78 | 13 | 24 | 44 | 28 | 33 | 134 | 7 | 32 |
| 108 | 32 | 23 | 152 | 50 | 42 | 13 | 27 | 111 | 50 | 99 | 16 | 84 | 14 | 40 | 33 | 36 | 41 | 121 | 14 | 36 |
| 109 | 46 | 32 | 128 | 57 | 61 | 15 | 40 | 122 | 56 | 123 | 11 | 93 | 15 | 27 | 46 | 37 | 28 | 132 | 16 | 37 |
| 110 | 52 | 28 | 127 | 58 | 54 | 8 | 36 | 157 | 54 | 143 | 10 | 78 | 16 | 29 | 53 | 28 | 57 | 166 | 22 | 48 |
| 111 | 42 | 29 | 155 | 56 | 70 | 15 | 34 | 156 | 44 | 156 | 21 | 104 | 21 | 30 | 44 | 66 | 47 | 200 | 16 | 42 |
| 112 | 47 | 22 | 160 | 82 | 65 | 17 | 48 | 227 | 45 | 147 | 19 | 78 | 12 | 30 | 57 | 44 | 65 | 220 | 32 | 61 |
| 113 | 61 | 31 | 140 | 60 | 70 | 15 | 25 | 166 | 64 | 182 | 20 | 87 | 23 | 48 | 46 | 56 | 72 | 217 | 34 | 55 |
| 114 | 67 | 33 | 153 | 87 | 70 | 15 | 45 | 240 | 70 | 170 | 11 | 93 | 14 | 48 | 71 | 64 | 77 | 235 | 17 | 70 |
| 115 | 66 | 37 | 165 | 76 | 85 | 20 | 58 | 269 | 89 | 202 | 25 | 93 | 27 | 54 | 63 | 58 | 79 | 283 | 28 | 63 |
| 116 | 53 | 30 | 139 | 107 | 75 | 24 | 44 | 250 | 96 | 250 | 24 | 111 | 31 | 53 | 76 | 74 | 78 | 285 | 25 | 70 |
| 117 | 61 | 46 | 146 | 124 | 105 | 17 | 49 | 319 | 93 | 242 | 33 | 105 | 43 | 49 | 102 | 68 | 99 | 339 | 33 | 68 |
| 118 | 98 | 36 | 158 | 114 | 119 | 25 | 59 | 339 | 109 | 266 | 27 | 111 | 37 | 65 | 100 | 69 | 118 | 347 | 43 | 88 |
| 119 | 88 | 34 | 142 | 125 | 94 | 23 | 50 | 343 | 108 | 307 | 36 | 94 | 49 | 68 | 104 | 71 | 130 | 395 | 31 | 85 |
| 120 | 80 | 56 | 125 | 115 | 110 | 24 | 55 | 388 | 98 | 334 | 37 | 89 | 45 | 66 | 104 | 75 | 151 | 436 | 42 | 74 |
| 121 | 90 | 45 | 102 | 128 | 135 | 23 | 51 | 412 | 125 | 325 | 44 | 74 | 69 | 82 | 106 | 81 | 198 | 468 | 39 | 88 |
| 122 | 113 | 46 | 101 | 142 | 130 | 39 | 62 | 390 | 154 | 298 | 42 | 92 | 58 | 95 | 117 | 96 | 159 | 492 | 42 | 92 |
| 123 | 125 | 52 | 134 | 146 | 142 | 42 | 75 | 399 | 145 | 341 | 42 | 93 | 77 | 101 | 122 | 88 | 194 | 522 | 44 | 104 |
| 124 | 124 | 60 | 135 | 153 | 144 | 35 | 63 | 448 | 169 | 411 | 51 | 81 | 84 | 85 | 136 | 131 | 197 | 551 | 41 | 123 |
| 125 | 147 | 43 | 120 | 173 | 118 | 40 | 82 | 450 | 171 | 389 | 43 | 94 | 82 | 99 | 152 | 128 | 214 | 562 | 42 | 142 |
| 126 | 135 | 59 | 102 | 182 | 140 | 39 | 68 | 471 | 179 | 381 | 49 | 99 | 111 | 97 | 177 | 106 | 225 | 601 | 50 | 146 |
| 127 | 138 | 62 | 128 | 190 | 152 | 39 | 78 | 490 | 184 | 411 | 58 | 84 | 107 | 100 | 190 | 150 | 261 | 595 | 45 | 129 |
| 128 | 170 | 56 | 132 | 199 | 167 | 47 | 79 | 448 | 186 | 399 | 58 | 108 | 133 | 122 | 146 | 132 | 255 | 626 | 43 | 153 |
| 129 | 187 | 73 | 111 | 213 | 142 | 46 | 71 | 489 | 227 | 367 | 56 | 82 | 163 | 106 | 189 | 125 | 259 | 668 | 43 | 154 |
| 130 | 220 | 64 | 111 | 232 | 157 | 44 | 75 | 500 | 249 | 422 | 68 | 101 | 170 | 129 | 183 | 141 | 289 | 638 | 50 | 148 |
| 131 | 201 | 65 | 106 | 229 | 169 | 64 | 97 | 447 | 194 | 412 | 60 | 101 | 167 | 134 | 200 | 170 | 294 | 616 | 50 | 154 |
| 132 | 210 | 47 | 115 | 246 | 186 | 48 | 78 | 417 | 243 | 386 | 62 | 115 | 244 | 142 | 200 | 151 | 264 | 614 | 56 | 160 |
| 133 | 242 | 61 | 128 | 218 | 195 | 74 | 97 | 420 | 217 | 426 | 75 | 101 | 230 | 127 | 191 | 161 | 262 | 550 | 65 | 160 |
| 134 | 233 | 55 | 121 | 201 | 177 | 82 | 94 | 416 | 202 | 371 | 76 | 89 | 257 | 119 | 204 | 187 | 257 | 516 | 58 | 190 |
| 135 | 234 | 62 | 157 | 247 | 172 | 79 | 74 | 369 | 221 | 363 | 95 | 70 | 308 | 143 | 183 | 174 | 270 | 513 | 60 | 164 |
| 136 | 276 | 68 | 106 | 238 | 196 | 65 | 97 | 323 | 245 | 374 | 81 | 91 | 290 | 131 | 213 | 223 | 225 | 483 | 78 | 177 |
| 137 | 278 | 31 | 130 | 237 | 193 | 77 | 94 | 318 | 241 | 379 | 75 | 106 | 333 | 118 | 220 | 224 | 227 | 466 | 60 | 158 |
| 138 | 288 | 60 | 145 | 256 | 185 | 86 | 92 | 301 | 232 | 395 | 88 | 83 | 308 | 132 | 203 | 204 | 212 | 425 | 62 | 159 |
| 139 | 299 | 68 | 121 | 243 | 184 | 89 | 94 | 276 | 220 | 367 | 72 | 95 | 308 | 124 | 196 | 194 | 213 | 398 | 66 | 153 |
| 140 | 332 | 59 | 131 | 242 | 194 | 96 | 92 | 229 | 230 | 329 | 88 | 87 | 340 | 122 | 215 | 206 | 187 | 380 | 63 | 176 |
| 141 | 301 | 55 | 119 | 240 | 200 | 118 | 101 | 185 | 209 | 342 | 80 | 80 | 358 | 131 | 199 | 224 | 186 | 339 | 71 | 172 |
| 142 | 347 | 50 | 116 | 215 | 192 | 112 | 99 | 191 | 213 | 326 | 67 | 103 | 337 | 134 | 200 | 235 | 163 | 291 | 71 | 170 |
| 143 | 345 | 61 | 130 | 195 | 191 | 108 | 88 | 138 | 221 | 336 | 77 | 76 | 353 | 123 | 200 | 247 | 166 | 280 | 51 | 154 |
| 144 | 314 | 53 | 132 | 196 | 188 | 104 | 84 | 154 | 212 | 349 | 75 | 88 | 386 | 120 | 174 | 258 | 179 | 249 | 49 | 185 |
| 145 | 331 | 46 | 120 | 202 | 179 | 139 | 67 | 146 | 226 | 301 | 71 | 80 | 347 | 115 | 172 | 258 | 160 | 215 | 66 | 163 |
| 146 | 305 | 55 | 137 | 215 | 179 | 124 | 87 | 125 | 204 | 294 | 77 | 75 | 377 | 111 | 159 | 245 | 151 | 235 | 58 | 171 |
| 147 | 338 | 48 | 99 | 189 | 162 | 133 | 89 | 129 | 176 | 308 | 63 | 94 | 359 | 140 | 197 | 269 | 168 | 215 | 70 | 157 |
| 148 | 339 | 44 | 99 | 184 | 157 | 117 | 77 | 116 | 215 | 282 | 60 | 87 | 344 | 115 | 164 | 243 | 163 | 171 | 64 | 169 |
| 149 | 325 | 53 | 111 | 206 | 173 | 125 | 75 | 99 | 177 | 294 | 64 | 92 | 375 | 127 | 161 | 252 | 170 | 176 | 43 | 135 |
| 150 | 312 | 57 | 99 | 198 | 168 | 126 | 80 | 104 | 166 | 284 | 63 | 69 | 346 | 121 | 167 | 287 | 207 | 180 | 54 | 168 |
| 151 | 314 | 65 | 96 | 193 | 165 | 147 | 90 | 83 | 182 | 256 | 55 | 66 | 329 | 101 | 175 | 286 | 181 | 179 | 44 | 170 |
| 152 | 296 | 53 | 105 | 192 | 182 | 146 | 76 | 98 | 173 | 223 | 46 | 87 | 337 | 112 | 164 | 281 | 198 | 178 | 45 | 156 |
| 153 | 281 | 46 | 114 | 164 | 166 | 120 | 78 | 116 | 179 | 245 | 58 | 76 | 308 | 97 | 162 | 266 | 208 | 190 | 61 | 139 |
| 154 | 295 | 57 | 117 | 135 | 178 | 132 | 79 | 84 | 165 | 217 | 56 | 57 | 299 | 102 | 173 | 285 | 210 | 149 | 52 | 141 |
| 155 | 291 | 51 | 97 | 158 | 157 | 140 | 72 | 102 | 138 | 199 | 68 | 72 | 237 | 84 | 154 | 277 | 221 | 195 | 33 | 147 |
| 156 | 269 | 51 | 79 | 166 | 162 | 168 | 69 | 89 | 149 | 187 | 49 | 71 | 278 | 112 | 143 | 278 | 254 | 172 | 46 | 131 |
| 157 | 236 | 39 | 92 | 123 | 170 | 144 | 68 | 87 | 140 | 202 | 53 | 45 | 262 | 96 | 133 | 282 | 230 | 190 | 44 | 145 |
| 158 | 256 | 37 | 89 | 139 | 160 | 149 | 70 | 106 | 124 | 187 | 56 | 73 | 215 | 76 | 116 | 319 | 243 | 182 | 40 | 120 |
| 159 | 199 | 47 | 83 | 130 | 168 | 164 | 63 | 91 | 130 | 191 | 43 | 76 | 212 | 83 | 137 | 307 | 240 | 151 | 37 | 129 |
| 160 | 221 | 53 | 92 | 95 | 174 | 197 | 61 | 104 | 143 | 128 | 37 | 65 | 208 | 76 | 128 | 284 | 256 | 163 | 44 | 134 |
| 161 | 182 | 41 | 84 | 109 | 148 | 120 | 48 | 94 | 103 | 129 | 40 | 57 | 191 | 68 | 127 | 272 | 251 | 137 | 50 | 125 |
| 162 | 188 | 51 | 76 | 102 | 137 | 162 | 59 | 94 | 107 | 104 | 45 | 57 | 171 | 60 | 103 | 303 | 228 | 136 | 47 | 119 |
| 163 | 154 | 47 | 70 | 78 | 136 | 158 | 75 | 77 | 94 | 141 | 35 | 64 | 158 | 53 | 89 | 258 | 257 | 140 | 28 | 110 |
| 164 | 163 | 47 | 77 | 88 | 125 | 169 | 50 | 65 | 91 | 92 | 30 | 53 | 149 | 53 | 92 | 261 | 276 | 126 | 30 | 105 |
| 165 | 133 | 48 | 68 | 64 | 88 | 166 | 56 | 77 | 88 | 95 | 28 | 67 | 142 | 58 | 90 | 258 | 249 | 101 | 33 | 82 |
| 166 | 123 | 42 | 70 | 86 | 123 | 162 | 46 | 45 | 78 | 87 | 24 | 42 | 119 | 49 | 63 | 228 | 245 | 106 | 29 | 97 |
| 167 | 100 | 31 | 59 | 53 | 96 | 188 | 49 | 62 | 77 | 58 | 21 | 58 | 111 | 46 | 68 | 234 | 209 | 89 | 18 | 77 |
| 168 | 104 | 32 | 68 | 50 | 61 | 170 | 44 | 43 | 58 | 68 | 16 | 49 | 108 | 40 | 55 | 198 | 206 | 75 | 27 | 77 |
| 169 | 96 | 29 | 66 | 36 | 65 | 182 | 44 | 31 | 62 | 55 | 18 | 57 | 89 | 33 | 46 | 195 | 200 | 63 | 26 | 46 |
| 170 | 77 | 38 | 63 | 33 | 69 | 173 | 30 | 40 | 52 | 53 | 22 | 60 | 72 | 32 | 47 | 172 | 168 | 58 | 19 | 70 |
| 171 | 75 | 22 | 75 | 29 | 55 | 197 | 31 | 38 | 38 | 27 | 16 | 51 | 59 | 23 | 45 | 156 | 164 | 50 | 17 | 54 |
| 172 | 60 | 29 | 67 | 28 | 45 | 178 | 30 | 30 | 37 | 42 | 6 | 62 | 70 | 15 | 33 | 159 | 136 | 36 | 10 | 40 |
| 173 | 54 | 19 | 68 | 34 | 37 | 217 | 18 | 15 | 27 | 28 | 12 | 56 | 62 | 18 | 40 | 117 | 131 | 41 | 14 | 33 |
| 174 | 42 | 24 | 58 | 31 | 28 | 211 | 23 | 27 | 35 | 28 | 11 | 56 | 45 | 23 | 37 | 128 | 119 | 35 | 15 | 17 |
| 175 | 41 | 25 | 61 | 30 | 35 | 179 | 20 | 13 | 31 | 24 | 14 | 49 | 41 | 15 | 24 | 97 | 110 | 35 | 10 | 33 |
| 176 | 37 | 17 | 64 | 25 | 26 | 210 | 21 | 12 | 18 | 17 | 7 | 50 | 37 | 11 | 21 | 85 | 109 | 25 | 8 | 23 |
| 177 | 35 | 19 | 64 | 15 | 29 | 215 | 12 | 15 | 15 | 28 | 4 | 58 | 33 | 11 | 23 | 67 | 80 | 16 | 3 | 16 |
| 178 | 23 | 13 | 77 | 20 | 14 | 192 | 13 | 11 | 18 | 21 | 1 | 58 | 22 | 13 | 19 | 70 | 77 | 16 | 5 | 10 |
| 179 | 23 | 13 | 71 | 11 | 15 | 212 | 10 | 3 | 10 | 11 | 4 | 43 | 17 | 13 | 10 | 67 | 70 | 14 | 2 | 5 |
| 180 | 2 | 0 | 3 | 1 | 0 | 15 | 0 | 0 | 1 | 0 | 0 | 2 | 3 | 1 | 1 | 5 | 4 | 0 | 2 | 1 |
